# Supplementary material for: Synthesis and Biological Evaluation of Sclareolide-Indole Conjugates and Their Derivatives
Source: Molecules. 2023 Feb 11;28(4):1737. doi: 10.3390/molecules28041737 (PMC9961340; doi:10.3390/molecules28041737)
Supplement: Supplementary file 1 [file molecules-28-01737-s001.zip › molecules-2213111-supplementary.pdf]

Supporting information for the article entitled:

Synthesis and Biological Evaluation of Sclareolide-Indole Conjugates and Their Derivatives

Table of Content

Table S1: Limitation of substrates of the coupling reaction[a] S3

Table S2: The X-ray data of compounds 3 .... S4

Table S3: X-ray structure of compound 8ab................................ S5

Figure S1: Cell cycle distribution figures of Decitabine............. S6

Figure S2: Cell cycle distribution figures of 10 .... S6

Figure S3: Cell apoptosis figures of Decitabine.......................... S7

Figure S4: Cell apoptosis figures of 10 .... S7

1. General chemistry methods S8

2. Procedures for synthesis of Sclareolide derivatives S8

3. ^{1}H NMR and ^{13}C NMR spectrum of intermediates and final compounds S31

4. Original HRMS spectra of final compounds S71

5. UPLC purity reports of final compounds S92

Table S1: Limitation of substrates of the coupling reaction[a]

Entry	Substitution Position	Yield	Entry	Substitution Position	Yield
**1**	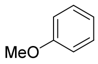	No reaction	**11**	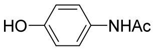	No reaction
**2**	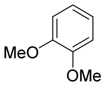	No reaction	**12**	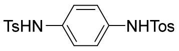	No reaction
**3**	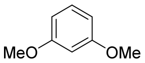	No reaction	**13**	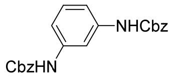	No reaction
**4**	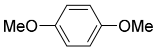	No reaction	**14**	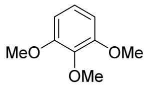	No reaction
**5**	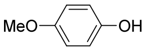	No reaction	**15**	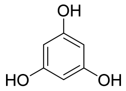	No reaction
**6**	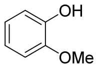	No reaction	**16**	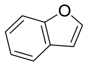	No reaction
**7**	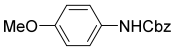	No reaction	**17**	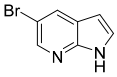	No reaction
**8**	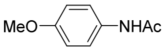	No reaction	**18**	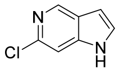	No reaction
**9**	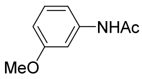	No reaction	**19**	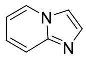	No reaction
**10**	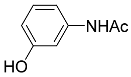	No reaction	**20**	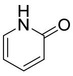	No reaction

[a] Compounds that have been tried but had no formation of expected product in the standard condition.

Table S2: The X-ray data of compounds 3

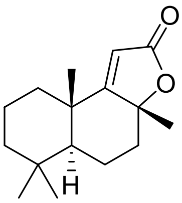

3

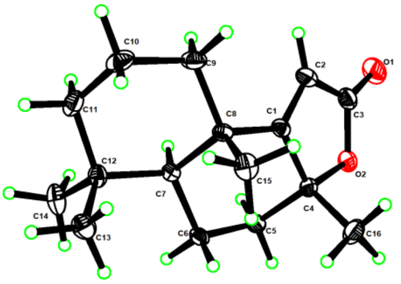

CCDC Deposition Number: 2238307.

Datablock: cu_2022563_0m

Bond precision:	C-C = 0.0028 A		Wavelength=1.54178
Cell:	a=7.3391(2)	b=10.7059(3)	c=9.5244(3)
	alpha=90	beta=111.776(2)	gamma=90
Temperature:	170 K		

	Calculated	Reported
Volume	694.95(4)	694.95(4)
Space group	P 21	P 1 21 1
Hall group	P 2yb	P 2yb
Moiety formula	C16 H24 O2	C16 H24 O2
Sum formula	C16 H24 O2	C16 H24 O2
Mr	248.35	248.35
Dx,g cm-3	1.187	1.187
Z	2	2
Mu (mm-1)	0.593	0.593
F000	272.0	272.0
F000'	272.75	
h,k,lmax	9,13,11	9,13,11
Nref	2835[1497]	2721
Tmin,Tmax	0.945,0.971	0.636,0.754
Tmin'	0.915	

Correction method= # Reported T Limits: Tmin=0.636 Tmax=0.754
AbsCorr = MULTI-SCAN

Data completeness= 1.82/0.96 Theta(max)= 74.496

R(reflections)= 0.0312(2608) wR2(reflections)= 0.0800(2721)

S = 1.054 Npar= 167

Table S3: X-ray structure of compound 8ab

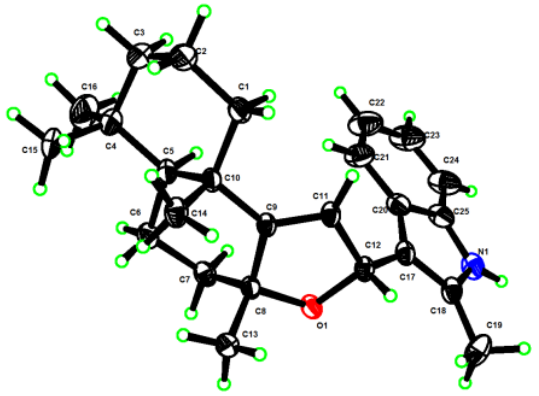

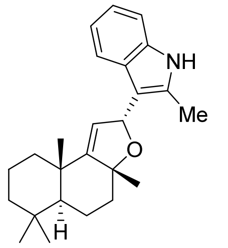

Major isomer of **8ab**

CCDC Deposition Number: 2236373.

Datablock: cu_20221527_0m

Bond precision: C–C = 0.0071 A   Wavelength=1.54178

Cell:	a=11.2980(14)	b=7.7424(10)	c=14.2186(18)
	alpha=90	beta=101.685(6)	gamma=90
Temperature:	170 K		

	Calculated	Reported
Volume	1218.0(3)	1218.0(3)
Space group	P 21	P 1 21 1
Hall group	P 2yb	P 2yb
Moiety formula	C25 H33 N O, C H2 Cl2	C H2 Cl2, C25 H33 N O
Sum formula	C26 H35 Cl2 N O	C26 H35 Cl2 N O
Mr	448.45	448.45
Dx,g cm-3	1.223	1.223
Z	2	2
Mu (mm-1)	2.515	2.515
F000	480.0	480.0
F000’	482.51	
h,k,lmax	13,9,17	13,9,17
Nref	4657[2513]	4315
Tmin,Tmax	0.785,0.882	0.545,0.753
Tmin’	0.739	

Correction method= # Reported T Limits: Tmin=0.545 Tmax=0.753
AbsCorr = MULTI-SCAN

Data completeness= 1.72/0.93   Theta(max)= 70.362

R(reflections)= 0.0716(3939)   wR2(reflections)= 0.2055(4315)

S = 1.041   Npar= 294

Figure S1: Cell cycle distribution figures of Decitabine

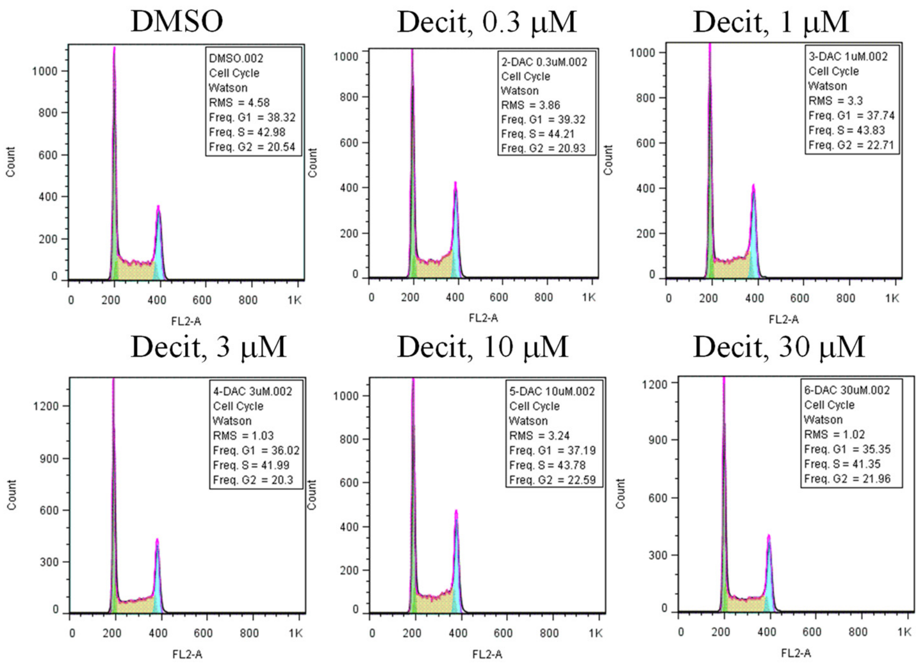

Cytometric flow analysis of cell cycle distribution of MV4-11 cells in the presence of Decitabine at the indicated concentrations for 48 h. The experiments were repeated twice.

Figure S2: Cell cycle distribution figures of 10

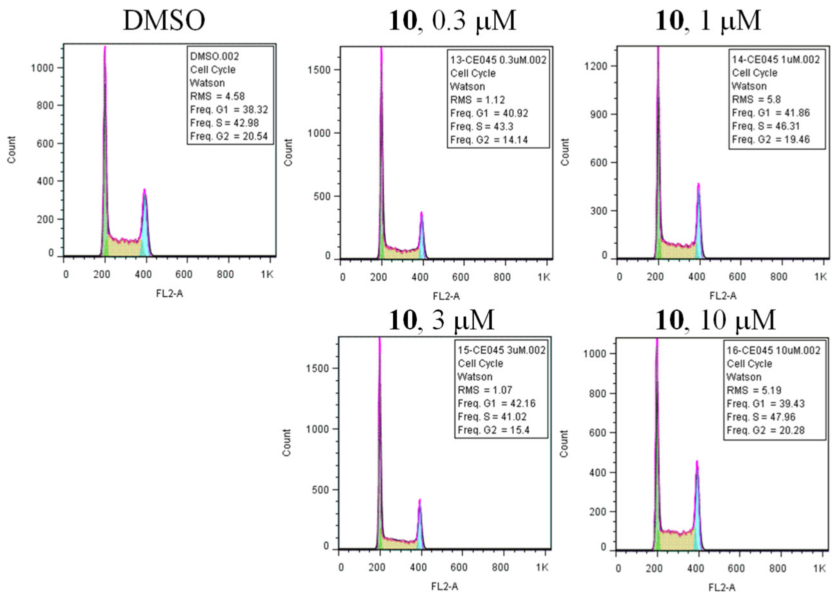

Cytometric flow analysis of cell cycle distribution of MV4-11 cells in the presence of **10** at the indicated concentrations for 48 h. The experiments were repeated twice.

Figure S3: Cell apoptosis figures of Decitabine

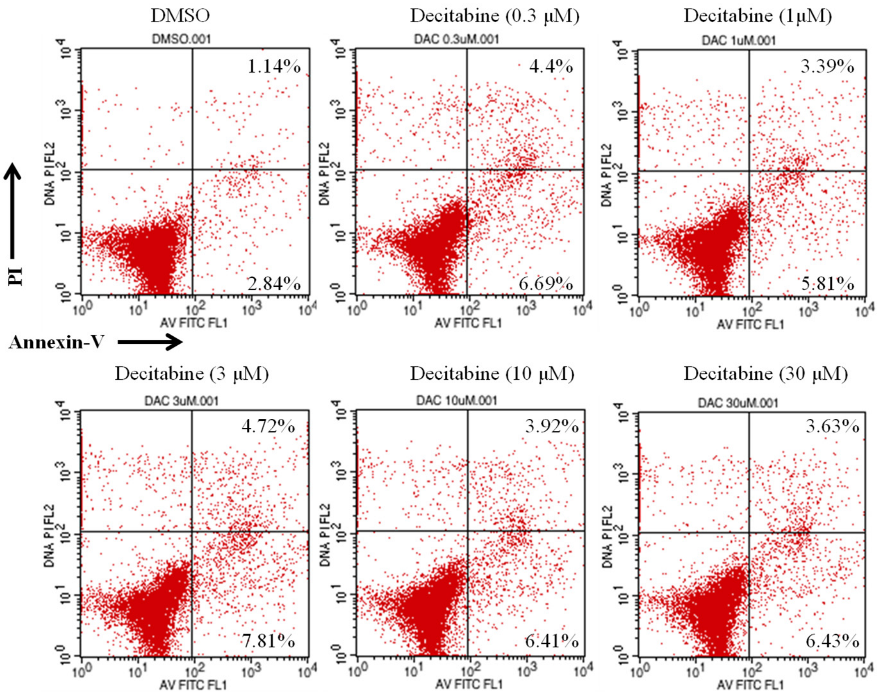

Cytometric flow analysis of cell apoptosis distribution of MV4-11 cells in the presence of Decitabine at the indicated concentrations for 48 h. The experiments were repeated twice.

Figure S4: Cell apoptosis figures of 10

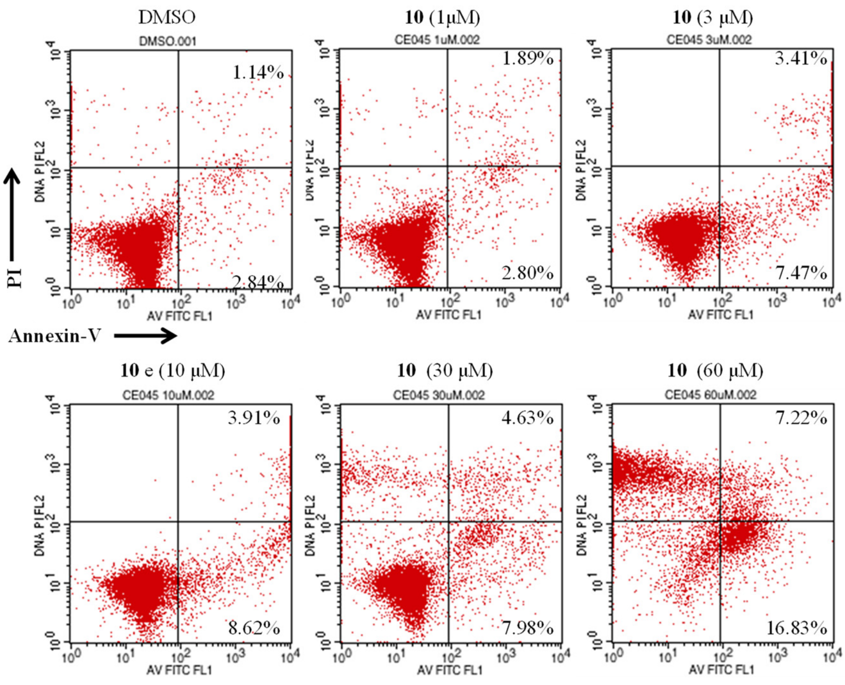

Cytometric flow analysis of cell apoptosis distribution of MV4-11 cells in the presence of **10** at the indicated concentrations for 48 h. The experiments were repeated twice.

1. General chemistry methods

All reactions were conducted in a round-bottomed flask equipped with a Teflon-coated magnet stirring bar. Experiments involving moisture and/or air sensitive components were performed under an N_2 atmosphere. Commercial reagents and anhydrous solvents were used without further purification. The crude reaction products were purified by flash column chromatography using silica gel. Proton nuclear magnetic resonance (^{1}H NMR) was performed in Bruker Advance 400 NMR spectrometers. Carbon nuclear magnetic resonance (^{13}C NMR) spectroscopy was performed in Bruker Advance 500 NMR spectrometers. High resolution ESI mass spectrum analysis was performed on Agilent Q-TOF mass spectrometer (G6520). The analytical UPLC model was Waters Acquity H class (UV detection at 230 nm and 254 nm) and the reverse phase column used was the Acquity UPLC® BEH (C18-1.7 μm, 2.1 × 50 mm). Further purification of final compounds for biological testing was performed on a preparative HPLC (Waters 2545) with a C18 reverse phase column. The mobile phase used here was a gradient flow of solvent A (water) and solvent B (CH_3CN) at a flow rate of 10 mL/min. All final compounds were purified to ≥ 95% purity as determined by analytical UPLC analysis. Isomer ratio was determined by integration of corresponding vinyl proton or O-CH peaks in ^{1}H NMR spectrum.

2. Procedures for synthesis of Sclareolide derivatives

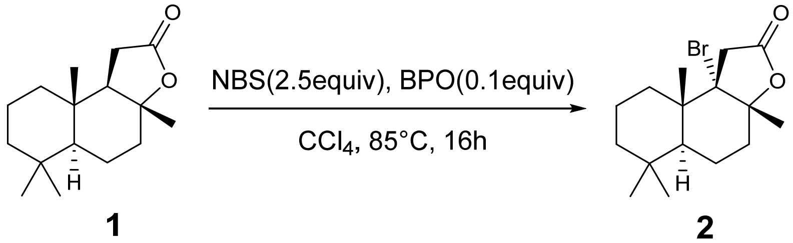

1
Sclareolide
2

A 250 ml round-bottom flask equipped with a magnetic stirring bar was charged with **1** (5.0 g, 19.97 mmol, 1.0 equiv), N-Bromosuccinimide (8.89 g, 49.92 mmol, 2.5 equiv), dibenzoyl peroxide (483.7 mg, 1.20 mmol, 0.1 equiv) and CCl_4 (60 mL). The mixture was stirred at 85°C for 16 h. The resulting mixture was filtered, and the filtrate was concentrated. The crude was purified by flash column chromatography to afford the title compound **2** as a white solid (3.48 g, 53%).

(3aR,5aS,9aS,9bS)-9b-bromo-3a,6,6,9a-tetramethyldecahydronaphtho[2,1-b]furan-2(1H)-one (2): white solid (3.48 g, 53%). ^{1}H NMR (500 MHz, $CDCl_3$) δ 3.54 – 3.46 (d, J = 18.0Hz, 1H), 3.14 (d, J = 18.0 Hz, 1H), 2.25 – 2.18 (m, 1H), 1.91 (dtd, J = 12.9, 3.3, 1.6 Hz, 1H), 1.75 (s, 3H), 1.71 – 1.61 (m, 2H), 1.57 – 1.43 (m, 3H), 1.43 – 1.32 (m, 2H), 1.29 (s, 3H), 1.19 – 1.10 (m, 2H), 0.96 (s, 3H), 0.89 (s, 3H). ^{13}C NMR (126 MHz, $CDCl_3$) δ 175.60, 94.65, 88.77, 49.55, 45.47, 43.87, 41.30, 38.50, 36.48, 33.82, 33.52, 26.18, 21.72, 18.88, 18.83, 17.98. HRMS (EI): *m/z* calculated for $C_{16}H_{25}{}^{79}BrO_2$ [M^+]: 328.1032, Found: 328.1033. Specific Rotation: $[\alpha]^{20}$ = -46.95° (c = 1.0 g/100 mL, in $CHCl_3$).

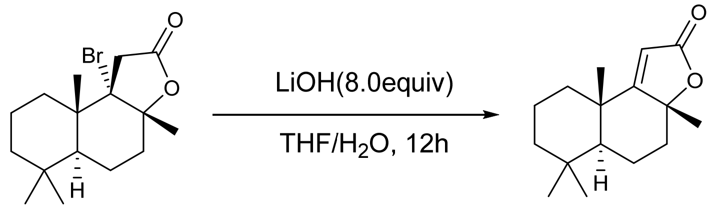

2 **3**

Dissolve **2** (3.48 g, 10.58 mmol, 1.0 equiv) in H_2O and THF (20/20 mL) then LiOH·H_2O (3.55 g, 84.62 mmol, 8.0 equiv) was added. The mixture was stirred at ambient temperature for 12 h. When the reaction was completed, adjust the pH of the solution to 6 with 1N HCl. The aqueous layer was extracted with EA (3 × 50 mL), and the combined organic layers were washed with brine (50 mL), dried over anhydrous Na_2SO_4, and concentrated in vacuo. The residual crude product was purified by flash column chromatography to afford the title compound **3** as a white solid (2.24 g, 85%).

(3aR,5aS,9aS)-3a,6,6,9a-tetramethyl-4,5,5a,6,7,8,9,9a-octahydronaphtho[2,1-b]furan-2(3aH)-one (3): white solid (2.24 g, 85%). ^{1}H NMR (500 MHz, $CDCl_3$) δ 5.51 (s, 1H), 2.29 (dd, J = 8.9, 2.9 Hz, 1H), 1.83 – 1.68 (m, 3H), 1.63-1.57 (m, 1H), 1.56-1.53 (m, 4H), 1.51-1.50 (m, 1H), 1.49 – 1.44 (m, 2H), 1.22 – 1.15 (m, 4H), 0.96 – 0.93 (m, 1H), 0.90 (d, J = 7.8 Hz, 6H). ^{13}C NMR (126 MHz, $CDCl_3$) δ 197.98, 173.72, 107.09, 84.78, 57.61, 41.49, 40.71, 39.66, 37.09, 34.73, 33.24, 25.22, 22.36, 19.36, 18.07, 17.91. HRMS (EI): m/z calculated for $C_{16}H_{24}O_2$ [M^+]: 248.1771, Found: 248.1758. Specific Rotation: $[\alpha]^{20}$ = -157.13° (C=1.0 g/100 mL, in $CHCl_3$).

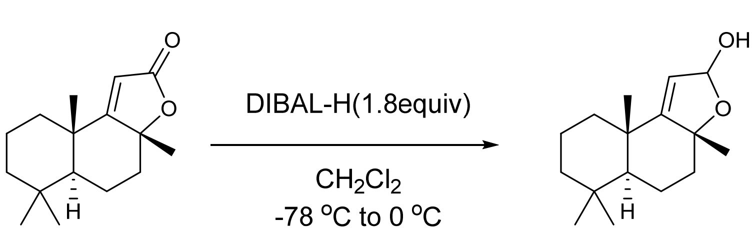

3 **4**

A 100 ml dry round-bottom flask equipped with a magnetic stirring bar was charged with **3** (1.2 g, 4.83 mmol, 1.0 equiv) and anhydrous CH_2Cl_2 (40 mL), DIBAL-H (1.0 M in Hexane, 8.7 mL, 8.7 mmol, 1.8 equiv) was added at -78°C. The resulting solution was allowed to stir from -78°C to 0°C for 12h. Quench the reaction mixture with water (30 mL) then add 2N NaOH (10 mL). The aqueous layer was extracted with CH_2Cl_2 (3 ×40 mL), and the combined organic layers were washed with brine (50 mL) , dried over anhydrous Na_2SO_4, filtered and concentrated in vacuo to

afford the crude product **4** as a white solid (1.18 g, 98%). The crude product **4** was used for next step without further purification.

(3aR,5aS,9aS)-3a,6,6,9a-tetramethyl-2,3a,4,5,5a,6,7,8,9,9a-decahydronaphtho[2,1-b]furan-2-ol (4): white solid (1.18 g, 98%). ^{1}H NMR (500 MHz, $CDCl_3$) δ 5.91 (d, J = 1.5 Hz, 1H), 5.19 (d, J = 1.5 Hz, 1H), 2.10 – 1.95 (m, 1H), 1.72 – 1.55 (m, 4H), 1.49 – 1.44 (m, 4H), 1.40 – 1.34 (m, 2H), 1.15 – 1.03 (m, 6H), 0.82 (d, J = 2.0 Hz, 6H). ^{13}C NMR (126 MHz, $CDCl_3$) δ 161.79, 113.90, 102.55, 86.94, 55.52, 42.41, 42.05, 37.86, 37.65, 33.71, 33.47, 28.70, 21.58, 19.98, 18.99, 18.55.

1. Procedure for coupling reactions of sclareolide-indole and sclareolide-aromatic compounds

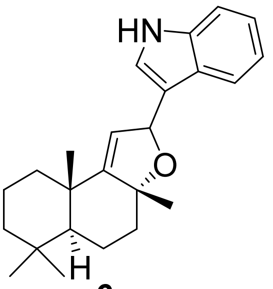

6

3-((3aR,5aS,9aS)-3a,6,6,9a-tetramethyl-2,3a,4,5,5a,6,7,8,9,9a-decahydronaphtho[2,1-b]furan-2-yl)-1H-indole (6):

General method A for the syntheses of **6**, **8a-8y**, **8ab-8af**, and **9-14:**

To a 100 mL dry round-bottom flask equipped with a magnetic stirring bar, **4** (0.20 mmol, 1.0 equiv), indole (0.16 mmol, 0.8 equiv) and anhydrous CH_2Cl_2 (10 mL) were added. The solution was cooled to -78°C then $TiCl_4$ (1.0 M in CH_2Cl_2, 0.16 mmol, 0.8 equiv) was added dropwise via a syringe, The reaction mixture was allowed to stirred at -78°C for 2.5 h before quenching with $NaHCO_3$ saturated aqueous solution (15 mL). The aqueous layer was extracted with CH_2Cl_2 (3 × 30 mL), and the combined organic layers were washed with brine (50 mL), dried over anhydrous sodium sulfate, and concentrated in vacuo. The residual crude product was purified by flash column chromatography to afford the desired product **6** (white solid, 20.4 mg, 58% yield, Isomer ratio: 69:31). ^{1}H NMR (500 MHz, $CDCl_3$) δ 8.07 (s, 1H), 7.74 (ddd, *J* = 8.0, 5.6, 1.0 Hz, 1H), 7.35 (dt, *J* = 8.2, 1.0 Hz, 1H), 7.21 – 7.16 (m, 2H), 7.14 – 7.07 (m, 1H), 6.05 (d, *J* = 1.3 Hz, 0.69H), 6.02 (d, J = 1.3Hz, 0.31H), 5.50(d, J = 1.2Hz, 0.31H), 5.40 (d, *J* = 1.2 Hz, 0.69H), 2.07 (ddt, *J* = 12.7, 6.6, 2.8 Hz, 1H), 1.81 – 1.64 (m, 4H), 1.55 – 1.38 (m, 7H), 1.34 – 1.14 (m, 5H), 1.08 (ddd, *J* = 50.3, 12.2, 2.3 Hz, 1H), 0.94 – 0.88 (m, 6H). ^{13}C NMR (126 MHz, $CDCl_3$) δ 157.03, 156.15, 136.97, 136.78, 126.21, 126.09, 123.30, 122.81, 121.93, 119.74, 119.61, 119.45, 119.42, 118.09, 117.42, 117.05, 115.77, 111.47, 111.39, 87.67, 87.20, 78.79, 78.30, 56.06, 55.22, 53.55, 42.79, 42.33, 42.28, 42.20, 38.21, 38.18, 38.14, 37.87, 33.83, 33.77, 33.63, 33.54, 28.68, 26.63, 21.63, 21.58, 20.52, 20.33, 20.15, 19.80, 18.74, 18.68. HRMS (ESI): *m*/*z* calculated for $C_{24}H_{32}NO$ $[M+H]^+$: 350.2484, Found $[M+H]^+$: 350.2484.

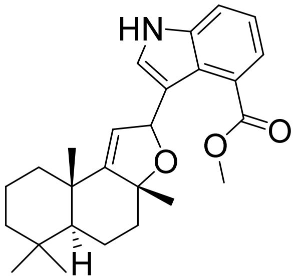

8a

Methyl 3-((3aR,5aS,9aS)-3a,6,6,9a-tetramethyl-2,3a,4,5,5a,6,7,8,9,9a decahydronaphtho [2,1-b]furan-2-yl)-1H-indole-4-carboxylate (8a). Method A: **4** (50 mg, 0.20 mmol), methyl indole-4-carboxylate (28.0 mg, 0.16 mmol), $TiCl_4$ (0.16 mL, 0.16 mmol) and CH_2Cl_2 (10 mL) were used to afford the desired product **8a** as yellow oil (33.8 mg, 52% yield, Isomer ratio: 51:49). ^{1}H NMR (500 MHz, $CDCl_3$) δ 7.91 (ddd, J = 7.3, 6.2, 0.9 Hz, 1H), 7.77 (dd, J = 12.2, 8.2 Hz, 1H), 7.36 (d, J = 3.4 Hz, 0.49H), 7.32 (d, J = 3.4 Hz, 0.51H), 7.18 (d, J = 3.3Hz, 0.49H), 7.14 (d, J = 3.3 Hz, 0.51H), 6.79 (d, J = 1.8 Hz, 1H), 5.44 (d, J = 1.8 Hz, 1H), 5.34 (d, J = 1.1 Hz, 1H), 3.97 (s, 3H), 2.07 (ddt, J = 40.6, 12.2, 3.1 Hz, 1H), 1.89 – 1.70 (m, 4H), 1.55 – 1.33 (m, 6H), 1.31 – 1.19 (m, 5H), 1.12 (dd, J = 12.2, 2.4 Hz, 0.49H), 1.00 (dd, J = 12.2, 2.5 Hz, 0.51H), 0.96 – 0.89 (m, 6H). ^{13}C NMR (126 MHz, $CDCl_3$) δ 168.17, 163.61, 163.05, 137.32, 137.13, 129.06, 126.87, 123.69, 123.63, 121.75, 121.66, 121.20, 121.12, 114.96, 114.69, 112.72, 111.03, 104.08, 103.44, 89.09, 88.32, 88.19, 87.49, 55.99, 55.81, 51.82, 42.86, 42.35, 42.12, 38.58, 38.38, 38.11, 34.00, 33.93, 33.56, 27.86, 27.59, 21.64, 20.39, 20.18, 19.69, 19.42, 18.63. HRMS (ESI): *m/z* calculated for $C_{26}H_{33}NNaO_3$ $[M+Na]^+$: 430.2358, Found $[M+Na]^+$: 430.2356.

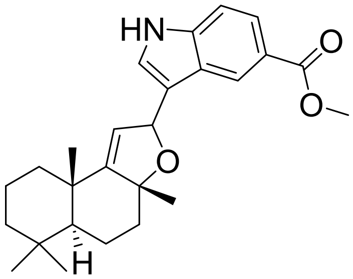

8b

methyl 3-((3aR,5aS,9aS)-3a,6,6,9a-tetramethyl-2,3a,4,5,5a,6,7,8,9,9a-decahydronaphtho [2,1-b]furan-2-yl)-1H-indole-5-carboxylate (8b): Method A: **4** (50 mg, 0.20 mmol), methyl indole-5-carboxylate (28.0 mg, 0.16 mmol), $TiCl_4$ (0.16 mL, 0.16 mmol) and CH_2Cl_2 (10 mL) were used to afford the desired product **8b** as yellow oil (60.3 mg, 92% yield, Isomer ratio: 76:24). ^{1}H NMR (500 MHz, Methanol-d_4) δ 8.53 (d, J = 1.6 Hz, 1H), 7.80 (dt, J = 8.6, 2.1 Hz, 1H), 7.39 (dd, J = 8.7, 1.8 Hz, 1H), 7.34 (d, J = 2.0 Hz, 0.76H), 7.25 (d, J = 2.0 Hz, 0.24H), 6.00-5.98 (m, 7 Hz, 1H), 5.52 (d, J = 3.7 Hz, 0.24H), 5.39 (d, J = 3.6 Hz, 0.76H), 3.89 (d, J = 2.4 Hz, 3H), 2.02 (ddd, J = 11.9, 4.5, 2.8 Hz, 1H), 1.89 – 1.76 (m, 4H), 1.63 – 1.53 (m, 4H), 1.51 – 1.34 (m, 4H), 1.29-1.22 (m, 4H), 1.04 – 0.90 (m, 6H). ^{13}C NMR (126 MHz, $CDCl_3$) δ 168.33, 157.64, 156.47, 139.66, 139.49, 125.70, 124.64, 124.13, 123.59, 123.40, 122.98, 122.88, 121.57, 118.51, 117.07, 115.53, 111.15, 111.07, 87.98, 87.50, 78.58, 78.04, 56.14, 55.02, 52.01, 51.92, 42.89, 42.32, 42.17, 38.33, 38.27, 38.18, 37.96, 33.87, 33.82, 33.56, 33.53, 28.44, 26.68, 21.69, 21.59, 20.57, 20.34,

20.15, 19.55, 18.73. HRMS (ESI): *m/z* calculated for $C_{26}H_{34}NO_3$ $[M+H]^+$: 408.2539, Found $[M+H]^+$: 408.2533.

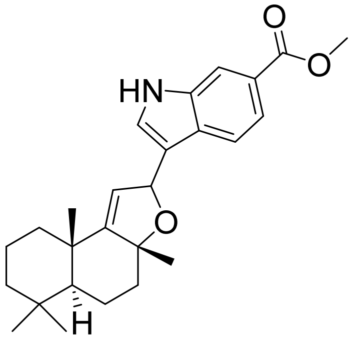

8c

Methyl 3-((3aR,5aS,9aS)-3a,6,6,9a-tetramethyl-2,3a,4,5,5a,6,7,8,9,9a decahydronaphtho [2,1-b]furan-2-yl)-1H-indole-6-carboxylate (8c): Method A: **4** (50 mg, 0.20 mmol), methyl indole-6-carboxylate (28.0 mg, 0.16 mmol), $TiCl_4$ (0.16 mL, 0.16 mmol) and CH_2Cl_2 (10 mL) were used to afford the desired product **8c** as yellow oil (47.4 mg, 73% yield, Isomer ratio: 69:31). ^1H NMR (500 MHz, Methanol-d_4) δ 8.10 (dd, J = 4.7, 1.6 Hz, 1H), 7.74 (dd, J = 8.4, 2.2 Hz, 1H), 7.69 (td, J = 8.2, 1.5 Hz, 1H), 7.44 (s, 0.69H), 7.35 (s, 0.31H), 6.04 (d, J = 1.2 Hz, 0.69H), 6.00 (d,J = 1.3 Hz, 0.31H), 5.40 (d, J = 1.3 Hz, 1H), 3.91 (d, J = 1.1 Hz, 3H), 2.04-2.01(m, 1H), 1.87 – 1.72 (m, 4H), 1.63 (td, J = 12.3, 10.7, 2.7 Hz, 1H), 1.58 – 1.55 (m, 1H), 1.53-1.44 (m, 4H), 1.35 – 1.21 (m, 5H), 1.19 (d, J = 2.4 Hz, 0.69H), 1.06 – 1.01 (m, 0.36H), 0.99 – 0.91 (m, 6H). ^{13}C NMR (126 MHz, $CDCl_3$) δ 168.33, 157.58, 156.76, 136.30, 136.10, 129.82, 126.45, 125.86, 123.75, 120.70, 120.67, 119.42, 119.25, 118.13, 117.06, 115.40, 113.80, 87.84, 87.40, 78.37, 77.92, 56.06, 55.36, 52.08, 42.73, 42.41, 42.29, 42.21, 38.28, 38.18, 37.95, 33.86, 33.82, 33.64, 33.56, 28.74, 26.59, 21.65, 21.59, 20.52, 20.35, 20.13, 19.82, 18.75, 18.69. HRMS (ESI): *m/z* calculated for $C_{26}H_{34}NO_3$ $[M+H]^+$: 408.2539, Found $[M+H]^+$: 408.2529.

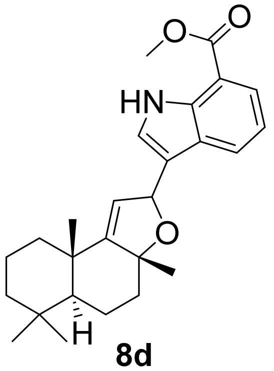

8d

Methyl 3-((3aR,5aS,9aS)-3a,6,6,9a-tetramethyl-2,3a,4,5,5a,6,7,8,9,9a decahydronaphtho [2,1-b]furan-2-yl)-1H-indole-7-carboxylate (8d): Method A: **4** (50 mg, 0.20 mmol), methyl indole-7-carboxylate (28.0 mg, 0.16 mmol), $TiCl_4$ (0.16 mL, 0.16 mmol) and CH_2Cl_2 (10 mL) were used to afford the desired product **8d** as yellow oil (38.9 mg, 60% yield, Isomer ratio: 82:18). ^1H NMR (500 MHz, $CDCl_3$) δ 9.74 (s, 1H), 7.97 (dt, J = 8.0, 0.8 Hz, 1H), 7.88 (dd, J = 7.6, 1.1 Hz, 1H), 7.29 (d, J = 2.4 Hz, 0.82H), 7.22 (d, J = 1.3Hz, 0.18H), 7.14 (td, J = 7.7, 1.6 Hz, 1H), 6.04

(dd, J = 14.5, 1.6 Hz, 1H), 5.49 (d, J = 1.3Hz, 0.18H), 5.38 (d, J = 1.1 Hz, 0.82H), 3.97 (d, J = 1.4 Hz, 3H), 2.13 – 2.02 (m, 1H), 1.75 (qt, J = 14.2, 2.5 Hz, 4H), 1.55 – 1.43 (m, 6H), 1.34 – 1.00 (m, 6H), 0.95 – 0.88 (m, 6H). ^{13}C NMR (126 MHz, $CDCl_3$) δ 168.04, 157.56, 136.88, 127.47, 125.63, 125.48, 124.50, 123.98, 123.36, 118.94, 117.92, 117.19, 115.46, 112.61, 87.72, 87.28, 78.28, 77.91, 56.04, 55.42, 52.00, 42.76, 42.50, 42.22, 38.28, 38.20, 37.93, 33.86, 33.82, 33.65, 33.57, 28.81, 26.52, 21.66, 21.60, 20.51, 20.37, 20.14, 19.89, 18.70. HRMS (ESI): *m/z* calculated for $C_{26}H_{34}NO_3$ $[M+H]^+$: 408.2539, Found $[M+H]^+$: 408.2528.

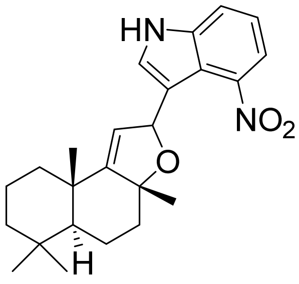

8e

4-nitro-3-((3aR,5aS,9aS)-3a,6,6,9a-tetramethyl-2,3a,4,5,5a,6,7,8,9,9a-decahydronaphtho[2,1-b]furan-2-yl)-1H-indole (8e): Method A: **4** (50 mg, 0.20 mmol), 4-nitroindole (25.9 mg, 0.16 mmol), $TiCl_4$ (0.16 mL, 0.16 mmol) and CH_2Cl_2 (10 mL) were used to afford major isomer **8e** (13.0 mg, 21% yield)and minor isomer **8e'** (6.0 mg, 10% yield) both as yellow oil (Isomer ratio: 68:32). **8e:** ^{1}H NMR (500 MHz, $CDCl_3$) δ 8.16 (d, J = 7.8Hz, 1H), 7.91 (d, J = 8.1 Hz, 1H), 7.43 (d, J = 3.3 Hz, 1H), 7.30 (t, J = 8.1 Hz, 1H), 7.24 (d, J = 3.3 Hz, 1H), 6.79 (d, J = 1.7 Hz, 1H), 5.45 (d, J = 1.8 Hz, 1H), 2.12 (dt, J = 12.0, 3.1 Hz, 1H), 1.91 – 1.73 (m, 4H), 1.57 – 1.40 (m, 3H), 1.34 (s, 3H), 1.27 – 1.18 (m, 5H), 1.00 (dd, J = 12.3, 2.5 Hz, 1H), 0.91 (d, J = 3.2 Hz, 6H). ^{13}C NMR (126 MHz, $CDCl_3$) δ 164.39, 140.46, 138.59, 129.17, 123.64, 120.92, 118.04, 117.02, 110.50, 102.74, 88.66, 87.71, 55.78, 42.77, 42.07, 38.65, 38.09, 33.94, 33.56, 27.82, 21.63, 20.14, 19.66, 18.61. HRMS (ESI): *m/z* calculated for Found $C_{24}H_{30}N_2NaO_3$ $[M+Na]^+$: 417.2154, Found $[M+Na]^+$: 417.2150. Specific Rotation: $[\alpha]^{20}$ = +40.52° (c = 1.0 g/100 mL, in $CHCl_3$). **8e':** ^{1}H NMR (500 MHz, $CDCl_3$) δ 8.14 (dd, *J* = 8.0, 0.8 Hz, 1H), 7.88 (d, *J* = 8.2 Hz, 1H), 7.47 (d, *J* = 3.4 Hz, 1H), 7.31 – 7.27 (m, 2H), 6.97 (d, *J* = 1.1 Hz, 1H), 5.35 (d, *J* = 1.1 Hz, 1H), 2.05 (dt, *J* = 11.9, 3.0 Hz, 1H), 1.85 – 1.71 (m, 4H), 1.58 – 1.55 (m, 1H), 1.54 (s, 3H), 1.52 – 1.44 (m, 2H), 1.38 – 1.16 (m, 5H), 1.13 – 1.01 (m, 1H), 0.93 (d, *J* = 18.9 Hz, 6H). ^{13}C NMR (126 MHz, $CDCl_3$) δ 163.78, 140.50, 138.40, 129.16, 123.58, 120.90, 118.02, 116.66, 112.12, 103.40, 89.60, 88.49, 56.06, 42.35, 42.04, 38.65, 38.38, 34.03, 33.55, 27.58, 21.65, 20.36, 19.37, 18.59. HRMS (ESI): *m/z* calculated for Found $C_{24}H_{30}N_2NaO_3$ $[M+Na]^+$: 417.2154, Found $[M+Na]^+$: 417.2153. Specific Rotation: $[\alpha]^{20}$ = -53.38° (c = 1.0 g/100 mL, in $CHCl_3$).

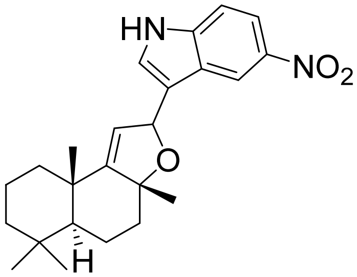

8f

5-nitro-3-((3aR,5aS,9aS)-3a,6,6,9a-tetramethyl-2,3a,4,5,5a,6,7,8,9,9a-decahydronaphtho[2,1-b]furan-2-yl)-1H-indole (8f): Method A: **4** (50 mg, 0.20 mmol), 5-nitroindole (25.9 mg, 0.16 mmol), $TiCl_4$ (0.16 mL, 0.16 mmol) and CH_2Cl_2 (10 mL) were used to afford the desired product **8f** as yellow oil (42.0 mg, 67% yield, Isomer ratio: 85:15). ^{1}H NMR (500 MHz, Methanol-d_4) δ 8.80 (d, J = 2.3 Hz, 0.85H), 8.74 (d, J = 2.3Hz, 0.15H), 8.05 (dd, J = 9.0, 2.2 Hz, 1H), 7.50 – 7.36 (m, 2H), 5.99 (d, J = 1.3 Hz, 1H), 5.54 (d, J = 1.4Hz, 0.15H), 5.39 (d, J = 1.2 Hz, 0.85H), 2.07 – 2.00 (m, 1H), 1.90 – 1.76 (m, 4H), 1.64 (dt, J = 11.8, 5.7 Hz, 1H), 1.58 (dt, J = 14.8, 3.2 Hz, 1H), 1.54-1.43 (m, 6H), 1.42 – 1.22 (m, 4H), 1.06 – 0.92 (m, 6H). ^{13}C NMR (126 MHz, $CDCl_3$) δ 158.31, 141.50, 140.12, 126.42, 125.70, 125.58, 119.50, 117.79, 117.72, 116.60, 111.37, 87.96, 77.81, 54.79, 42.12, 41.94, 38.41, 38.11, 33.88, 33.55, 33.44, 28.49, 27.00, 21.70, 21.59, 20.31, 20.03, 19.65, 18.71. HRMS (ESI): *m/z* calculated for Found $C_{24}H_{31}N_2O_3$ $[M+H]^+$: 395.2335, Found $[M+H]^+$: 395.2332.

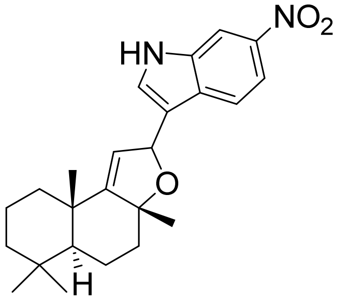

8g

6-nitro-3-((3aR,5aS,9aS)-3a,6,6,9a-tetramethyl-2,3a,4,5,5a,6,7,8,9,9a-decahydronaphtho[2,1-b]furan-2-yl)-1H-indole (8g): Method A: **4** (50 mg, 0.20 mmol), 6-nitroindole (25.9 mg, 0.16 mmol), $TiCl_4$ (0.16 mL, 0.16 mmol) and CH_2Cl_2 (10 mL) were used to afford the desired product **8g** as yellow oil (34.9 mg, 55% yield, Isomer ratio: 69:31). ^{1}H NMR (500 MHz, $CDCl_3$) δ 8.76 (d, J = 13.1 Hz, 1H), 8.28 (dd, J = 3.5, 2.0 Hz, 1H), 8.00 (ddd, J = 8.8, 4.5, 2.0 Hz, 1H), 7.78 (d, J = 8.8 Hz, 1H), 7.44 (d, J = 2.4 Hz, 0.69H), 7.37 (d, J = 2.3Hz, 0.31H), 6.04 (d, J = 1.2 Hz, 0.69H), 6.00 (d, J = 1.2Hz, 0.31H), 5.46 (d, J = 1.3Hz, 0.31H), 5.35 (d, J = 1.2 Hz, 0.69H), 2.08 (ddt, J = 15.9, 12.0, 3.1 Hz, 1H), 1.82 – 1.70 (m, 4H), 1.58 – 1.44 (m, 6H), 1.36 – 1.14 (m, 5H), 1.10 – 1.00 (m, 1H), 0.95 – 0.88 (m, 6H). ^{13}C NMR (126 MHz, $CDCl_3$) δ 158.14, 157.33, 143.35, 135.39, 135.23, 130.95, 128.84, 128.27, 119.65, 119.53, 118.71, 116.65, 115.17, 114.97, 108.37, 88.17, 87.68, 78.03, 77.61, 56.06, 55.49, 42.67, 42.46, 42.26, 42.19, 38.36, 38.32, 38.19, 38.02, 33.88, 33.84, 33.64, 33.55, 28.75, 26.52, 21.65, 21.59, 20.49, 20.32, 20.11, 19.83, 18.73, 18.67. HRMS (ESI): *m/z* calculated for Found $C_{24}H_{31}N_2O_3$ $[M+H]^+$: 395.2335, Found $[M+H]^+$: 395.2328.

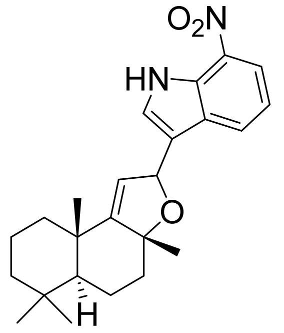

8h

7-nitro-3-((3aR,5aS,9aS)-3a,6,6,9a-tetramethyl-2,3a,4,5,5a,6,7,8,9,9a-decahydronaphtho[2,1-b]furan-2-yl)-1H-indole (8h): Method A: **4** (50 mg, 0.20 mmol), 7-nitroindole (25.9 mg, 0.16 mmol), $TiCl_4$ (0.16 mL, 0.16 mmol) and CH_2Cl_2 (10 mL) were used to afford the desired product **8h** as yellow oil (61.5 mg, 87% yield, Isomer ratio: 68:32). ^{1}H NMR (500 MHz, $CDCl_3$) δ 9.81 (s, 1H), 8.16 (d, J = 8.1 Hz, 1H), 8.11 (dd, J = 11.0, 7.7 Hz, 1H), 7.37 (d, J = Hz, 0.32H), 7.29 (d, J = 2.4 Hz, 0.68H), 7.20 (td, J = 7.9, 1.3 Hz, 1H), 6.06 (d, J = 1.9Hz, 0.31H), 6.01 (d, J = 1.9Hz, 0.68H), 5.48 (d, J = 1.9 Hz, 0.68H), 5.31 (d, J = 1.8Hz, 0.31H), 2.08 (ddt, J = 15.1, 12.0, 3.2 Hz, 1H), 1.81 – 1.69 (m, 4H), 1.57 – 1.42 (m, 6H), 1.29 – 1.15 (m, 5H), 1.05 (ddd, J = 26.4, 12.3, 2.4 Hz, 1H), 0.94 – 0.88 (m, 6H). ^{13}C NMR (126 MHz, $CDCl_3$) δ 158.13, 157.54, 133.00, 130.30, 130.12, 128.07, 127.98, 125.12, 124.44, 120.75, 119.53, 119.43, 119.11, 116.63, 114.88, 88.02, 87.56, 77.82, 77.49, 55.97, 55.50, 42.66, 42.49, 42.24, 42.17, 38.28, 38.18, 37.96, 33.85, 33.81, 33.62, 33.54, 28.79, 26.44, 21.63, 21.57, 20.44, 20.31, 20.08, 19.86, 18.71, 18.65. HRMS (ESI): *m/z* calculated for Found $C_{24}H_{31}N_2O_3$ $[M+H]^+$: 395.2335, Found $[M+H]^+$: 395.2329.

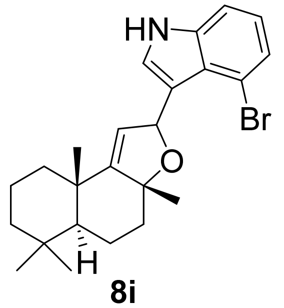

8i

4-bromo-3-((3aR,5aS,9aS)-3a,6,6,9a-tetramethyl-2,3a,4,5,5a,6,7,8,9,9a-decahydronaphtho[2,1-b]furan-2-yl)-1H-indole (8i): Method A: **4** (50 mg, 0.20 mmol), 4-bromo-1H-indole (31.4 mg, 0.16 mmol), $TiCl_4$ (0.16 mL, 0.16 mmol) and CH_2Cl_2 (10 mL) were used to afford the desired product **8i** as yellow oil (29.6 mg, 43% yield, Isomer ratio: 62:38). ^{1}H NMR (500 MHz, Methanol-d_4) δ 7.34 (ddd, J = 8.1, 3.7, 0.9 Hz, 1H), 7.26 (d, J = 0.9 Hz, 0.62H), 7.23 (d, J = 0.9Hz, 0.38H), 7.19 (ddd, J = 7.6, 2.6, 0.9 Hz, 1H), 6.96 (t, J = 7.8 Hz, 1H), 6.71 (t, J = 1.1 Hz, 0.62H), 6.63 (d, J = 1.2Hz, 0.38H), 5.55 (d, J = 1.3Hz, 0.38H), 5.49 (d, J = 1.2 Hz, 0.62H), 2.07 (ddt, J = 24.2, 11.8, 2.9 Hz, 1H), 1.81 – 1.70 (m, 3H), 1.69 – 1.54 (m, 2H), 1.53 – 1.45 (m, 5H), 1.45 – 1.39 (m, 1H), 1.29 – 1.16 (m, 4H), 1.03 (dt, J = 12.2, 2.6 Hz, 1H), 0.92 – 0.88 (m, 6H). ^{13}C NMR (126 MHz, $CDCl_3$) δ 155.13, 155.02, 137.74, 124.74, 123.94, 123.83, 123.16, 122.73, 122.66, 120.39, 120.24, 119.12, 117.88, 113.77, 113.66, 110.61, 87.95, 87.48, 79.01, 78.42, 55.92, 55.26, 43.20, 42.75, 42.24, 42.10, 38.05, 37.99, 37.69, 33.81, 33.72, 33.52, 33.49, 29.82, 29.25, 26.92, 21.60,

21.56, 20.53, 20.36, 20.03, 19.76, 18.72, 18.63. HRMS (ESI): *m/z* calculated for $C_{24}H_{31}{}^{79}BrNO$ $[M+H]^+$: 428.1589, $C_{24}H_{31}{}^{81}BrNO$ $[M+H]^+$: 430.1569, Found $[M+H]^+$: 428.1585, 430.1576.

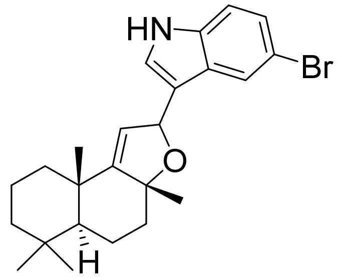

8j

5-bromo-3-((3aR,5aS,9aS)-3a,6,6,9a-tetramethyl-2,3a,4,5,5a,6,7,8,9,9a-decahydronaphtho[2,1-b]furan-2-yl)-1H-indole (8j): Method A: **4** (50 mg, 0.20 mmol), 5-bromine indole (31.4 mg, 0.16 mmol), $TiCl_4$ (0.16 mL, 0.16 mmol) and CH_2Cl_2 (10 mL) were used to afford the desired product **8j** as yellow oil (50.0 mg, 73% yield, Isomer ratio: 76:24). ^1H NMR (500 MHz, Methanol-d_4) δ 7.92 (d, J = 1.9 Hz, 0.76H), 7.84 (d, J = 1.9Hz, 0.24H), 7.31 – 7.15 (m, 3H), 5.97 – 5.91 (m, 1H), 5.50 (d, J = 2.2Hz, 0.24H), 5.36 (d, J = 2.1Hz, 0.76H), 2.02 (dt, J = 12.1, 3.0 Hz, 1H), 1.86 – 1.76 (m, 4H), 1.65 – 1.55 (m, 3H), 1.52-1.43 (m, 3H), 1.37 – 1.30 (m, 2H), 1.25-1.21 (m, 3H), 1.13 – 0.89 (m, 7H). ^{13}C NMR (126 MHz, $CDCl_3$) δ 157.34, 156.67, 135.64, 135.40, 127.88, 124.84, 124.76, 124.54, 123.86, 122.88, 122.35, 118.03, 117.09, 116.77, 115.36, 112.86, 112.78, 112.73, 87.87, 87.57, 78.41, 78.09, 56.08, 55.04, 42.79, 42.29, 42.22, 42.11, 38.37, 38.30, 38.18, 38.03, 33.86, 33.56, 28.62, 27.03, 21.62, 21.59, 20.52, 20.35, 20.05, 19.77, 18.75, 18.70. HRMS (ESI): *m/z* calculated for $C_{24}H_{31}{}^{79}BrNO$ $[M+H]^+$: 428.1589, $C_{24}H_{31}{}^{81}BrNO$ $[M+H]^+$: 430.1569, Found $[M+H]^+$: 428.1578, 430.1577.

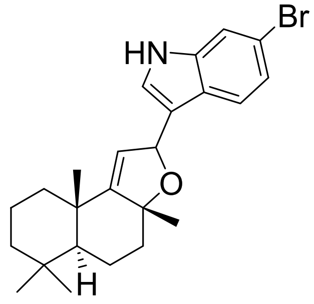

8k

6-bromo-3-((3aR,5aS,9aS)-3a,6,6,9a-tetramethyl-2,3a,4,5,5a,6,7,8,9,9a-decahydronaphtho[2,1-b]furan-2-yl)-1H-indole (8k): Method A: **4** (50 mg, 0.20 mmol), 6-bromine indole (31.4 mg, 0.16 mmol), $TiCl_4$ (0.16 mL, 0.16 mmol) and CH_2Cl_2 (10 mL) were used to afford the desired product **8j** as yellow oil (41.9 mg, 61% yield, Isomer ratio: 71:29). ^1H NMR (500 MHz, $CDCl_3$) δ 8.11 (s, 1H), 7.60 (dd, *J* = 8.5, 3.7 Hz, 1H), 7.49 (d, *J* = 1.7 Hz, 1H), 7.22 – 7.18 (m, 1H),7.15 (d, *J* = 2.4 Hz, 0.71H),7.08 (d, J = 2.4Hz, 0.29H) 6.00 (d, *J* = 1.2 Hz, 0.71H), 5.96 (d, J = 1.2Hz, 0.29H), 5.46 (d, J = 1.3Hz, 0.29H), 5.35 (d, *J* = 1.2 Hz, 0.71H), 2.11 – 2.03 (m, 1H), 1.78 – 1.66 (m, 4H), 1.53 – 1.43 (m, 6H), 1.31 – 1.14 (m, 5H), 1.05 (ddd, *J* = 38.5, 12.2, 2.2 Hz, 1H), 0.93 – 0.88 (m, 6H). ^{13}C NMR (126 MHz, $CDCl_3$) δ 157.54, 156.74, 137.74, 137.55, 125.11, 123.61, 123.05, 122.93, 121.07, 120.95, 117.83, 117.10, 115.73, 115.42, 114.30, 114.23, 87.79, 87.32,

78.40, 77.96, 56.06, 55.38, 42.74, 42.40, 42.29, 42.22, 38.27, 38.19, 37.94, 33.87, 33.82, 33.65, 33.56, 28.72, 26.56, 21.65, 21.60, 20.52, 20.34, 20.14, 19.84, 18.75, 18.69. HRMS (ESI): *m/z* calculated for $C_{24}H_{31}{}^{79}BrNO$ $[M+H]^+$: 428.1589, $C_{24}H_{31}{}^{81}BrNO$ $[M+H]^+$: 430.1569, Found $[M+H]^+$: 428.1580, 430.1578.

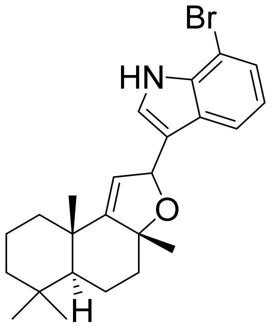

8l

7-bromo-3-((3aR,5aS,9aS)-3a,6,6,9a-tetramethyl-2,3a,4,5,5a,6,7,8,9,9a-decahydronaphtho[2,1-b]furan-2-yl)-1H-indole (8l): Method A: **4** (50 mg, 0.20 mmol), 7-bromine indole (31.4 mg, 0.16 mmol), $TiCl_4$ (0.16 mL, 0.16 mmol) and CH_2Cl_2 (10 mL) were used to afford the desired product **8j** as yellow oil (46.5 mg, 68% yield, Isomer ratio: 70:30). ^{1}H NMR (500 MHz, $CDCl_3$) δ 8.23 (s, 1H), 7.68 (t, *J* = 8.1 Hz, 1H), 7.34 (dd, *J* = 7.6, 0.9 Hz, 1H), 7.22 (dd, *J* = 30.3, 2.5 Hz, 1H), 6.99 (td, *J* = 7.8, 1.4 Hz, 1H), 6.02 (d, J = 1.2Hz, 0.7H), 5.98 (d, J = 1.3Hz, 0.3H), 5.48 (d, J = 1.7Hz, 0.3H), 5.37 (d, J = 1.6Hz, 0.7H), 2.11 – 2.04 (m, 1H), 1.79 – 1.64 (m, 4H), 1.53 – 1.42 (m, 6H), 1.32 – 1.14 (m, 5H), 1.05 (ddd, *J* = 37.3, 12.2, 2.2 Hz, 1H), 0.93 – 0.88 (m, 5H). ^{13}C NMR (126 MHz, $CDCl_3$) δ 157.51, 135.60, 127.38, 124.51, 123.34, 122.76, 120.84, 119.31, 119.12, 119.00, 117.00, 115.34, 104.89, 87.78, 87.34, 78.58, 78.16, 56.03, 55.36, 42.74, 42.47, 42.29, 42.21, 38.24, 38.18, 37.93, 33.86, 33.81, 33.64, 33.57, 28.78, 26.56, 21.65, 21.60, 20.51, 20.36, 20.13, 19.84, 18.75, 18.69. HRMS (ESI): *m/z* calculated for $C_{24}H_{31}{}^{79}BrNO$ $[M+H]^+$: 428.1589, $C_{24}H_{31}{}^{81}BrNO$ $[M+H]^+$: 430.1569, Found $[M+H]^+$: 428.1586, 430.1576.

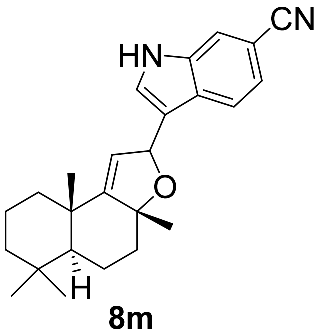

8m

3-((3aR,5aS,9aS)-3a,6,6,9a-tetramethyl-2,3a,4,5,5a,6,7,8,9,9a-decahydronaphtho[2,1-b]furan-2-yl)-1H-indole-6-carbonitrile (8m): Method A: **4** (50 mg, 0.20 mmol), 6-cyanoindole (22.7 mg, 0.16 mmol), $TiCl_4$ (0.16 mL, 0.16 mmol) and CH_2Cl_2 (10 mL) were used to afford the desired product **8m** as yellow oil (50.4 mg, 84% yield, Isomer ratio: 67:33). ^{1}H NMR (500 MHz, Methanol-d_4) δ 7.85 – 7.79 (m, 1H), 7.77 (dd, *J* = 5.4, 1.3 Hz, 1H), 7.49 (s, 0.67H), 7.40 (s, 0.33H), 7.29 (dt, *J* = 8.3, 1.3 Hz, 1H), 6.03 (d, *J* = 1.2 Hz, 0.67H), 5.98 (d, J = 1.3Hz, 0.33H), 5.52 (d, J = 1.3Hz, 0.33H), 5.38 (d, *J* = 1.3 Hz, 0.67H), 2.03 (ddt, *J* = 12.4, 9.5, 3.0 Hz, 1H), 1.84 –

1.69 (m, 4H), 1.65 – 1.55 (m, 2H), 1.53 – 1.41 (m, 4H), 1.30 – 0.99 (m, 6H), 0.97 – 0.91 (m, 6H).^{13}C NMR (126 MHz, $CDCl_3$) δ 157.98, 157.17, 135.71, 135.55, 129.35, 126.90, 126.35, 122.55, 120.76, 120.55, 120.44, 119.55, 118.43, 116.71, 116.32, 115.04, 104.47, 88.08, 87.59, 78.06, 77.65, 56.05, 55.47, 42.67, 42.45, 42.25, 42.18, 38.32, 38.18, 37.98, 33.87, 33.82, 33.64, 33.54, 28.75, 26.50, 21.64, 20.48, 20.31, 20.11, 19.84, 18.66. HRMS (ESI): *m/z* calculated for $C_{25}H_{31}N_2O$ $[M+H]^+$: 375.2436, Found $[M+H]^+$: 375.2431.

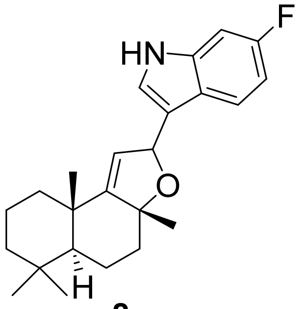

8n

6-fluoro-3-((3aR,5aS,9aS)-3a,6,6,9a-tetramethyl-2,3a,4,5,5a,6,7,8,9,9a-decahydronaphtho[2,1-b]furan-2-yl)-1H-indole (8n): Method A: **4** (50 mg, 0.20 mmol), 6-fluoroindole (21.6 mg, 0.16 mmol), $TiCl_4$ (0.16 mL, 0.16 mmol) and CH_2Cl_2 (10 mL) were used to afford the desired product **8n** as yellow oil (45.4 mg, 77% yield, Isomer ratio: 70:30). ^{1}H NMR (500 MHz, Methanol-d_4) δ 7.62 (ddd, *J* = 8.6, 5.4, 3.0 Hz, 1H), 7.21 (s, 0.7H), 7.12 (s, 0.3H), 7.04 (ddd, *J* = 10.1, 6.6, 2.3 Hz, 1H), 6.79 (ddt, *J* = 9.8, 8.8, 2.4 Hz, 1H), 5.99 (d, *J* = 1.2 Hz, 1H), 5.95 (d, J = 1.3Hz, 0.3H), 5.50 (d, J = 1.4Hz, 0.3H), 5.38 (d, *J* = 1.2 Hz, 1H), 2.03 (ddt, *J* = 11.7, 5.6, 2.9 Hz, 1H), 1.86 – 1.71 (m, 4H), 1.65 – 1.59 (m, 1H), 1.57 – 1.54 (m, 1H), 1.52-1.44 (m, 4H), 1.35 – 1.18 (m, 5H), 1.18 – 1.02 (m, 1H), 0.97 – 0.90 (m, 6H). ^{13}C NMR (126 MHz, $CDCl_3$) δ 157.51, 156.63, 136.97, 136.87, 123.37, 122.80, 120.55, 120.47, 117.58, 117.19, 115.53, 108.45, 108.26, 97.75, 97.55, 87.78, 87.27, 78.52, 78.07, 56.09, 55.39, 42.76, 42.42, 42.30, 42.22, 38.27, 38.18, 37.93, 33.87, 33.82, 33.66, 33.57, 28.73, 26.55, 21.66, 21.60, 20.53, 20.35, 20.16, 19.85, 18.76, 18.70. HRMS (ESI): *m/z* calculated for $C_{24}H_{31}FNO$ $[M+H]^+$: 368.2390, Found $[M+H]^+$: 368.2387.

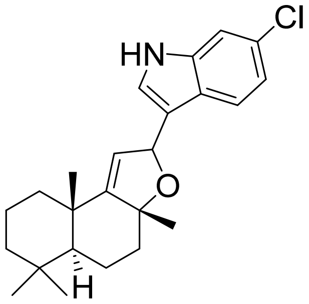

8p

6-chloro-3-((3aR,5aS,9aS)-3a,6,6,9a-tetramethyl-2,3a,4,5,5a,6,7,8,9,9a-decahydronaphtho[2,1-b]furan-2-yl)-1H-indole (8p): Method A: **4** (50 mg, 0.20 mmol), 6-chloroindole (24.2 mg, 0.16 mmol), $TiCl_4$ (0.16 mL, 0.16 mmol) and CH_2Cl_2 (10 mL) were used to afford the desired product **8p** as yellow oil (46.9 mg, 76% yield, Isomer ratio: 70:30). ^{1}H NMR (500 MHz, Methanol-d_4) δ 7.62 (dd, *J* = 8.5, 4.0 Hz, 1H), 7.35 (dd, *J* = 6.2, 1.9 Hz, 1H), 7.24 (s, 0.7H), 7.15 (s, 0.3H), 6.99

(ddd, J = 8.5, 4.4, 1.9 Hz, 1H), 5.99 (d, J = 1.2 Hz, 0.7H), 5.95 (d, J = 1.2Hz, 0.3H), 5.50 (d, J = 1.4Hz, 0.3H), 5.38 (d, J = 1.2Hz, 0.7H), 2.03 (ddt, J = 12.1, 5.9, 2.9 Hz, 1H), 1.87 – 1.70 (m, 4H), 1.65 – 1.58 (m, 1H), 1.57 – 1.54 (m, 1H), 1.52-1.43 (m, 4H), 1.34 – 1.18 (m, 5H), 1.17 – 1.02 (m, 1H), 0.97 – 0.90 (m, 6H). ^{13}C NMR (126 MHz, $CDCl_3$) δ 157.50, 156.65, 137.32, 137.14, 128.00, 124.76, 123.84, 123.32, 120.60, 120.50, 120.29, 118.53, 117.45, 117.12, 115.45, 111.37, 111.28, 87.84, 87.35, 78.46, 78.01, 56.07, 55.36, 42.74, 42.37, 42.29, 42.21, 38.27, 38.17, 37.94, 33.86, 33.81, 33.65, 33.56, 28.69, 26.58, 21.65, 21.60, 20.52, 20.33, 20.14, 19.83, 18.75, 18.69. HRMS (ESI): *m/z* calculated for $C_{24}H_{31}{}^{35}ClNO$ $[M+H]^+$: 384.2094, $C_{24}H_{31}{}^{37}ClNO$ $[M+H]^+$: 386.2065, Found $[M+H]^+$: 384.2090, 386.2075.

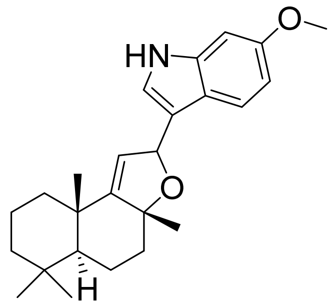

8q

6-methoxy-3-((3aR,5aS,9aS)-3a,6,6,9a-tetramethyl-2,3a,4,5,5a,6,7,8,9,9a-decahydronaphtho[2,1-b]furan-2-yl)-1H-indole (8q): Method A: **4** (50 mg, 0.20 mmol), 6-methoxyindole (23.5 mg, 0.16 mmol), $TiCl_4$ (0.16 mL, 0.16 mmol) and CH_2Cl_2 (10 mL) were used to afford the desired product **8q** as white solid (32.3 mg, 53% yield, Isomer ratio: 59:41). ^{1}H NMR (500 MHz, Methanol-d_4) δ 7.33 (dd, J = 8.6, 5.9 Hz, 1H), 6.91 (dd, J = 11.5, 2.3 Hz, 1H), 6.64 (dt, J = 8.6, 2.3 Hz, 1H), 6.28 (d, J = 0.8 Hz, 0.59H), 6.21 (d, J = 0.8Hz, 0.41H), 5.83 (d, J = 1.3 Hz, 0.59H), 5.74 (d, J = 1.3Hz, 0.41H), 5.42 (d, J= 1.4Hz, 0.41H), 5.29 (d, J = 1.3 Hz, 0.59H), 3.80 (d, J = 1.7 Hz, 3H), 2.07 – 2.00 (m, 1H), 1.84 – 1.72 (m, 4H), 1.59 – 1.45 (m, 6H), 1.32 – 0.99 (m, 6H), 0.97 – 0.90 (m, 6H). ^{13}C NMR (126 MHz, $CDCl_3$) δ 158.48, 158.06, 156.37, 139.03, 138.36, 136.87, 122.87, 121.22, 121.20, 115.94, 114.24, 109.78, 109.72, 99.62, 99.42, 94.68, 88.32, 88.03, 78.89, 78.65, 55.92, 55.80, 55.61, 43.50, 42.52, 42.23, 42.09, 38.29, 38.12, 38.00, 33.85, 33.51, 29.19, 26.62, 21.64, 21.58, 20.43, 20.33, 19.96, 19.86, 18.71, 18.65. HRMS (ESI): *m/z* calculated for $C_{25}H_{34}NO_2$ $[M+H]^+$: 380.2590, Found $[M+H]^+$: 380.2588.

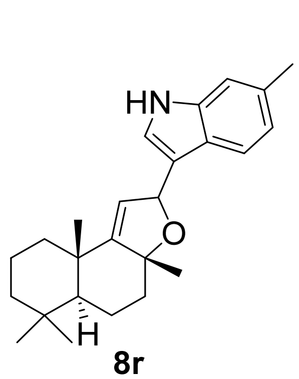

8r

6-methyl-3-((3aR,5aS,9aS)-3a,6,6,9a-tetramethyl-2,3a,4,5,5a,6,7,8,9,9a-decahydronaphtho[2,1-b]furan-2-yl)-1H-indole (8r): Method A: **4** (50 mg, 0.20 mmol), 6-methyl-1H-indole (21.0 mg,

0.16 mmol), $TiCl_4$ (0.16 mL, 0.16 mmol) and CH_2Cl_2 (10 mL) were used to afford the desired product **8r** as white solid (39.1 mg, 67% yield, Isomer ratio: 69:31). ^{1}H NMR (500 MHz, $CDCl_3$) δ 8.06 (s, 1H), 7.62 (dd, *J* = 8.3, 1.7 Hz, 1H), 7.09 – 7.05 (m, 1H), 7.04 (d, *J* = 2.4 Hz, 0.69H), 6.97 (d, J = 2.4Hz, 0.31H), 6.94 (ddd, *J* = 8.2, 4.6, 1.5 Hz, 1H), 6.02 (d, *J* = 1.2 Hz, 0.69H), 5.99 (d, J = 1.3Hz, 0.31H), 5.49 (d, J = 1.3Hz, 0.31H), 5.39 (d, *J* = 1.2 Hz, 0.69H), 2.44 (s, 3H), 2.08 (dt, *J* = 11.8, 2.8 Hz, 1H), 1.79 – 1.65 (m, 4H), 1.58 – 1.43 (m, 7H), 1.26 – 1.16 (m, 4H), 1.08 (ddd, *J* = 53.5, 12.3, 2.4 Hz, 1H), 0.94 – 0.89 (m, 6H). ^{13}C NMR (126 MHz, $CDCl_3$) δ 156.97, 156.17, 137.47, 137.26, 131.82, 124.00, 122.43, 121.91, 121.30, 119.48, 119.35, 118.44, 117.52, 117.39, 115.84, 111.33, 111.27, 87.56, 87.13, 78.84, 78.35, 56.06, 55.25, 42.81, 42.39, 42.31, 42.24, 38.21, 38.17, 37.89, 33.85, 33.79, 33.65, 33.56, 28.72, 26.65, 21.79, 21.65, 21.60, 20.54, 20.37, 20.16, 19.83, 18.77, 18.71. HRMS (ESI): *m*/*z* calculated for $C_{25}H_{34}NO$ $[M+H]^+$: 364.2640, Found $[M+H]^+$: 364.2636.

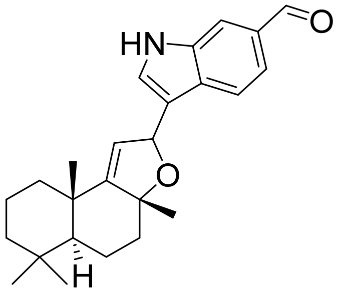

8s

3-((3aR,5aS,9aS)-3a,6,6,9a-tetramethyl-2,3a,4,5,5a,6,7,8,9,9a-decahydronaphtho[2,1-b]furan -2-yl)-1H-indole-6-carbaldehyde (8s): Method A: **4** (50 mg, 0.20 mmol), Indole-6-carboxaldehyde (23.2.0 mg, 0.16 mmol), $TiCl_4$ (0.16 mL, 0.16 mmol) and CH_2Cl_2 (10 mL) were used to afford the desired product **8s** as yellow oil (46.3 mg, 77% yield, Isomer ratio: 70:30). ^{1}H NMR (500 MHz, $CDCl_3$) δ 10.03 (s, 1H), 8.51 (s, 1H), 7.92 – 7.82 (m, 2H), 7.64 (ddd, *J* = 8.3, 2.5, 1.4 Hz, 1H), 7.42 (d, *J* = 2.5 Hz, 0.7H), 7.36 (d, J = 2.5Hz, 0.3H), 6.05 (d, *J* = 1.3 Hz, 0.7H), 6.02 (d, j = 1.3Hz, 0.3H), 5.48 (d, J = 1.3Hz, 0.3H), 5.37 (d, *J* = 1.2 Hz, 0.7H), 2.12 – 2.04 (m, 1H), 1.80 – 1.66 (m, 4H), 1.54 – 1.44 (m, 6H), 1.33 – 1.15 (m, 5H), 1.07 (ddd, *J* = 40.6, 12.3, 2.3 Hz, 1H), 0.97 – 0.87 (m, 6H). ^{13}C NMR (126 MHz, $CDCl_3$) δ 192.83, 157.72, 156.91, 136.48, 136.32, 131.27, 131.21, 131.10, 127.86, 127.34, 120.87, 120.75, 120.02, 119.89, 119.36, 118.28, 116.91, 115.24, 114.55, 114.38, 88.02, 87.55, 78.27, 77.82, 56.05, 55.39, 42.70, 42.42, 42.26, 42.19, 38.31, 38.28, 38.17, 37.97, 33.86, 33.81, 33.64, 33.54, 28.74, 26.59, 21.64, 21.58, 20.50, 20.32, 20.11,19.82, 18.73, 18.66. HRMS (ESI): *m*/*z* calculated for $C_{25}H_{30}NO_2$ $[M-H]^+$: 376.2277, Found $[M-H]^-$: 376.2289.

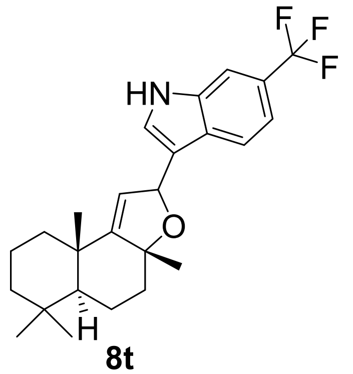

8t

3-((3aR,5aS,9aS)-3a,6,6,9a-tetramethyl-2,3a,4,5,5a,6,7,8,9,9a-decahydronaphtho[2,1-b]furan -2-yl)-6-(trifluoromethyl)-1H-indole (8t): Method A: **4** (50 mg, 0.20 mmol), 6-(trifluoromethyl)indole (29.6 mg, 0.16 mmol), $TiCl_4$ (0.16 mL, 0.16 mmol) and CH_2Cl_2 (10 mL) were used to afford the desired product **8t** as yellow oil (41.2 mg, 62% yield, Isomer ratio: 73:27). ^1H NMR (500 MHz, $CDCl_3$) δ 8.66 (d, J = 18.6 Hz, 1H), 7.81 (dd, J = 8.3, 3.6 Hz, 1H), 7.57 – 7.49 (m, 1H), 7.33 (td, J = 8.4, 1.6 Hz, 1H), 7.16 (d, J = 2.5 Hz, 0.73H), 7.03 (d, J = 2.5Hz, 0.27H), 6.03 (d, J = 1.2 Hz, 0.73H), 5.99 (d, J = 1.3Hz, 0.27H), 5.46 (d, J = 1.2Hz, 0.27H), 5.37 (d, J = 1.2 Hz, 0.73H), 2.11 – 2.05 (m, 1H), 1.80 – 1.65 (m, 4H), 1.61 – 1.41 (m, 7H), 1.27 – 1.15 (m, 4H), 1.08 (ddd, J = 43.0, 12.2, 2.3 Hz, 1H), 0.96 – 0.89 (m, 6H). ^{13}C NMR (126 MHz, $CDCl_3$) δ 157.72, 156.83, 135.83, 135.66, 128.52, 128.42, 126.39, 125.78, 125.28, 124.23, 124.06, 120.17, 120.05, 117.71, 117.01, 116.28, 115.34, 108.96, 87.98, 87.47, 78.42, 77.96, 56.10, 55.41, 42.74, 42.37, 42.29, 42.21, 38.33, 38.29, 38.19, 37.98, 33.88, 33.83, 33.65, 33.56, 28.67, 26.55, 21.66, 21.60, 20.53, 20.33, 20.15, 19.81, 18.75, 18.69. HRMS (ESI): m/z calculated for $C_{25}H_{31}F_3NO$ $[M+H]^+$: 418.2358, Found $[M+H]^+$: 418.2352.

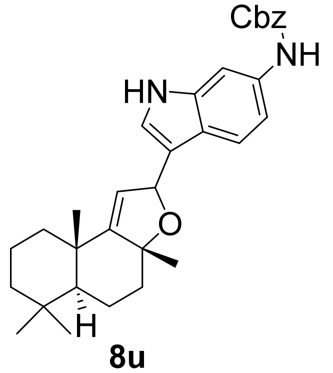

8u

benzyl (3-((3aR,5aS,9aS)-3a,6,6,9a-tetramethyl-2,3a,4,5,5a,6,7,8,9,9a-decahydronaphtho [2,1-b]furan-2-yl)-1H-indol-6-yl)carbamate (8u): Method A: **4** (50 mg, 0.20 mmol), benzyl (1H-indol-6-yl)carbamate (29.6 mg, 0.16 mmol), $TiCl_4$ (0.16 mL, 0.16 mmol) and CH_2Cl_2 (10 mL) were used to afford the desired product **8u** as yellow oil (62.4 mg, 78% yield, Isomer ratio: 69:31). ^1H NMR (500 MHz, Methanol-d_4) δ 7.67 (s, 1H), 7.57 (d, J = 8.5 Hz, 1H), 7.43 (d, J = 7.1 Hz, 2H), 7.39 – 7.34 (m, 2H), 7.33 – 7.28 (m, 1H), 7.15 (s, 0.69H), 7.06 (s, 0.31H), 6.93 (dt, J = 8.5, 2.5 Hz, 1H), 5.97 (d, J = 1.2 Hz, 0.69H), 5.94 (d, J = 1.3Hz, 0.31H), 5.49 (d, J = 1.5Hz, 0.31H), 5.37 (d, J = 1.4 Hz, 0.69H), 5.18 (s, 2H), 2.02 (dt, J = 11.9, 3.0 Hz, 1H), 1.86 – 1.70 (m, 4H), 1.65 – 1.58 (m, 1H), 1.57 – 1.53 (m, 1H), 1.51-1.44 (m, 4H), 1.33 – 1.01 (m, 6H), 0.98 – 0.89 (m, 6H).

^{13}C NMR (126 MHz, $CDCl_3$) δ 157.23, 156.34, 137.22, 137.06, 136.43, 132.61, 128.71, 128.40, 128.37, 123.39, 122.82, 119.99, 119.84, 118.16, 117.36, 117.04, 115.68, 87.65, 87.15, 78.65, 78.18, 66.96, 56.06, 55.28, 42.76, 42.32, 42.29, 42.20, 38.22, 38.15, 37.88, 33.84, 33.78, 33.64, 33.55, 28.65, 26.56, 21.65, 21.58, 20.52, 20.32, 20.15, 19.81, 18.75, 18.68, 2.00. HRMS (ESI): *m/z* calculated for $C_{32}H_{39}N_2O_3$ $[M+H]^+$: 499.2961, Found $[M+H]^+$: 499.2958.

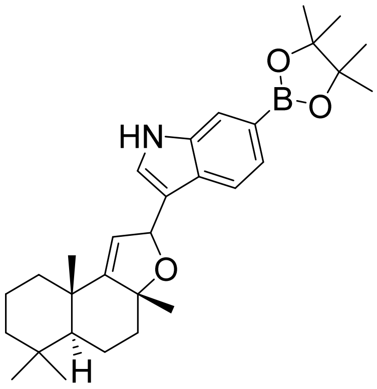

8v

6-(4,4,5,5-tetramethyl-1,3,2-dioxaborolan-2-yl)-3-((3aR,5aS,9aS)-3a,6,6,9a-tetramethyl-2,3a,4,5,5a,6,7,8,9,9a-decahydronaphtho[2,1-b]furan-2-yl)-1H-indole (8v): Method A: **4** (50 mg, 0.20 mmol), indole-6-boronic acid pinacol ester (38.8 mg, 0.16 mmol), $TiCl_4$ (0.16 mL, 0.16 mmol) and CH_2Cl_2 (10 mL) were used to afford the desired product **8v** as yellow oil (62.3 mg, 82% yield, Isomer ratio: 70:30). ^1H NMR (500 MHz, $CDCl_3$) δ 8.33 (d, *J* = 14.8 Hz, 1H), 7.83 (d, *J* = 1.6 Hz, 1H), 7.77 (dd, *J* = 8.1, 5.5 Hz, 1H), 7.55 (dd, *J* = 8.0, 1.1 Hz, 1H), 7.18 (d, *J* = 2.6 Hz, 0.7H), 7.12 (d, 2.5Hz, 0.3H), 6.02 (dd, *J* = 4.8, 1.5 Hz, 1H), 5.49 (d, J = 1.4Hz, 0.3H), 5.38 (d, *J* = 1.2 Hz, 1H), 2.06 (dt, *J* = 13.9, 3.8 Hz, 1H), 1.83 – 1.67 (m, 4H), 1.66 – 1.55 (m, 1H), 1.53 – 1.44 (m, 5H), 1.37 (d, *J* = 1.7 Hz, 12H), 1.27 – 1.01 (m, 6H), 0.96 – 0.87 (m, 6H). ^{13}C NMR (126 MHz, $CDCl_3$) δ 157.01, 156.25, 136.79, 136.53, 128.69, 125.40, 125.33, 124.68, 123.98, 119.35, 119.07, 118.91, 118.40, 117.69, 117.46, 115.74, 87.59, 87.21, 83.64, 78.67, 78.23, 56.04, 55.22, 42.81, 42.25, 38.23, 38.18, 37.91, 33.82, 33.77, 33.62, 33.55, 28.66, 26.71, 25.03, 25.00, 21.64, 21.58, 20.52, 20.36, 20.13, 19.76, 18.75, 18.69. HRMS (ESI): *m/z* calculated for $C_{30}H_{43}BNO_3$ $[M+H]^+$: 476.3336, Found $[M+H]^+$: 376.3335.

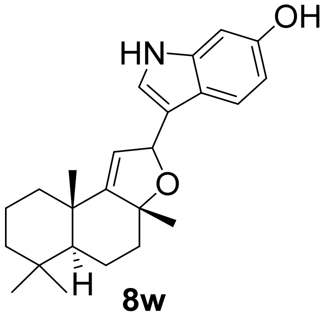

8w

3-((3aR,5aS,9aS)-3a,6,6,9a-tetramethyl-2,3a,4,5,5a,6,7,8,9,9a-decahydronaphtho[2,1-b]furan -2-yl)-1H-indol-6-ol (8w): Method A: **4** (50 mg, 0.20 mmol), 6-hydroxyindole (21.3 mg, 0.16 mmol), $TiCl_4$ (0.16 mL, 0.16 mmol) and CH_2Cl_2 (10 mL) were used to afford the desired product **8w** as yellow oil (28.2mg, 48% yield, Isomer ratio: 73:27). ^1H NMR (500 MHz, Methanol-d_4) δ

7.47 (d, *J* = 8.5 Hz, 1H), 7.03 (s, 0.73H), 6.95 (s, 0.27H), 6.76 (dd, *J* = 5.4, 2.2 Hz, 1H), 6.58 (ddd, *J* = 8.7, 6.9, 2.2 Hz, 1H), 5.93 (dd, *J* = 11.2, 1.6 Hz, 1H), 5.47 (d, J = 1.3Hz, 0.27H), 5.36 (d, *J* = 1.3 Hz, 0.73H), 2.03 – 1.98 (m, 1H), 1.84 – 1.71 (m, 4H), 1.63 – 1.44 (m, 7H), 1.29 – 1.01 (m, 5H), 0.97 – 0.90 (m, 6H). ^{13}C NMR (126 MHz, Methanol-d_4) δ 157.83, 157.00, 154.15, 139.74, 139.50, 123.48, 122.89, 121.74, 121.41, 120.84, 120.51, 118.84, 117.71, 117.04, 116.65, 110.18, 110.12, 97.78, 97.64, 88.84, 88.38, 80.08, 79.85, 75.82, 57.36, 56.58, 43.85, 43.33, 43.16, 39.37, 39.27, 39.25, 38.96, 34.65, 34.61, 34.01, 33.92, 30.78, 29.03, 26.88, 25.02, 21.93, 21.89, 21.39, 21.23, 20.65, 20.39, 19.65, 19.59. HRMS (ESI): *m/z* calculated for $C_{24}H_{32}NO_2$ [M+H]$^+$: 366.2433, Found [M+H]$^+$: 366.2433.

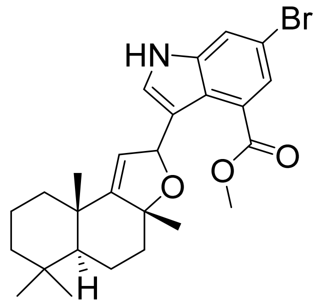

8x

methyl 6-bromo-3-((3aR,5aS,9aS)-3a,6,6,9a-tetramethyl-2,3a,4,5,5a,6,7,8,9,9a-decahydronaphtho[2,1-b]furan-2-yl)-1H-indole-4-carboxylate (8x): Method A: **4** (50 mg, 0.20 mmol), methyl 6-bromo-1H-indole-4-carboxylate (45.7 mg, 0.16 mmol), $TiCl_4$ (0.16 mL, 0.16 mmol) and CH_2Cl_2 (10 mL) were used to afford major isomer **8x** (11.2 mg, 14% yield) and minor isomer **8x'** (10.1 mg, 13% yield) both as transparent oil (Isomer ratio: 52:48). **8x:** ^{1}H NMR (500 MHz, $CDCl_3$) δ 7.99 (d, *J* = 1.7 Hz, 1H), 7.96 (d, *J* = 1.9 Hz, 1H), 7.32 (d, *J* = 3.3 Hz, 1H), 7.13 (dd, *J* = 3.2, 0.7 Hz, 1H), 6.84 (d, *J* = 1.1 Hz, 1H), 5.34 (d, *J* = 1.1 Hz, 1H), 3.97 (s, 3H), 2.02 (dt, *J* = 12.3, 3.1 Hz, 1H), 1.84 – 1.70 (m, 4H), 1.55 – 1.43 (m, 6H), 1.33 – 1.25 (m, 2H), 1.19 -1.16(m, 4H), 0.94 (d, *J* = 24.3 Hz, 6H). ^{13}C NMR (126 MHz, $CDCl_3$) δ 166.92, 163.26, 137.64, 128.40, 128.34, 126.32, 122.82, 118.11, 114.33, 112.58, 104.05, 89.33, 89.21, 55.62, 52.12, 42.07, 41.89, 38.64, 38.41, 34.04, 33.50, 27.65, 21.66, 20.34, 19.42, 18.61. HRMS (ESI): *m/z* calculated for $C_{26}H_{32}{}^{35}BrNNaO_3$ [M+Na]$^+$: 508.1463, $C_{26}H_{32}{}^{37}BrNNaO_3$ [M+Na]$^+$: 510.1443, Found [M+Na]$^+$: 508.1458, 510.1446. Specific Rotation: $[\alpha]^{20}$ = -47.72° (c =1.0 g/100 mL, in $CHCl_3$). **8x'** (mg): ^{1}H NMR (500 MHz, $CDCl_3$) δ 8.01 (d, *J* = 1.8 Hz, 1H), 7.93 (d, *J* = 1.7 Hz, 1H), 7.29 (d, *J* = 3.3 Hz, 1H), 7.09 (d, *J* = 3.3 Hz, 1H), 6.69 (d, *J* = 1.8 Hz, 1H), 5.42 (d, *J* = 1.8 Hz, 1H), 3.96 (s, 3H), 2.11 (dt, *J* = 11.9, 3.1 Hz, 1H), 1.90 – 1.71 (m, 4H), 1.56-1.52 (m, 1H), 1.49-1.41 (m, 2H), 1.33 (s, 3H), 1.26 – 1.18 (m, 5H), 0.99 (dd, *J* = 12.2, 2.4 Hz, 1H), 0.91 (d, *J* = 4.5 Hz, 6H). ^{13}C NMR (126 MHz, $CDCl_3$) δ 166.95, 164.05, 138.06, 128.13, 127.57, 126.43, 122.73, 117.84, 114.53, 110.66, 103.54, 88.46, 87.61, 55.76, 52.11, 42.79, 42.07, 38.62, 38.08, 33.94, 33.55, 27.75, 21.64, 20.15, 19.65, 18.62. HRMS (ESI): *m/z* calculated for $C_{26}H_{32}{}^{35}BrNNaO_3$ [M+Na]$^+$: 508.1463, $C_{26}H_{32}{}^{37}BrNNaO_3$ [M+Na]$^+$: 510.1443, Found [M+Na]$^+$: 508.1463, 510.1456. Specific Rotation: $[\alpha]^{20}$ = +92.97° (c = 1.0 g/100 mL, in $CHCl_3$).

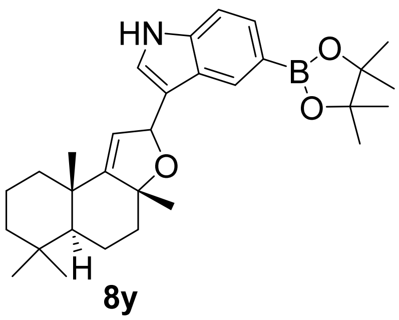

8y

5-(4,4,5,5-tetramethyl-1,3,2-dioxaborolan-2-yl)-3-((3aR,5aS,9aS)-3a,6,6,9a-tetramethyl-2,3a,4,5,5a,6,7,8,9,9a-decahydronaphtho[2,1-b]furan-2-yl)-1H-indole (8y): Method A: **4** (50 mg, 0.20 mmol), indole-5-boronic acid pinacol ester (38.8 mg, 0.16 mmol), $TiCl_4$ (0.16 mL, 0.16 mmol) and CH_2Cl_2 (10 mL) were used to afford the desired product **8y** as yellow oil (64.0 mg, 84% yield, Isomer ratio: 71:29). ^{1}H NMR (500 MHz, $CDCl_3$) δ 8.30 (s, 0.29), 8.23 (s, 0.71H), 8.09 (d, J = 11.6 Hz, 1H), 7.63 (ddd, J = 12.4, 8.2, 1.1 Hz, 1H), 7.33 (dd, J = 8.3, 2.3 Hz, 1H), 7.18 (d, J = 2.3 Hz, 0.71H), 7.12 (d, J = 2.2Hz, 0.29H), 6.07 (dd, J = 14.1, 1.6 Hz, 1H), 5.50 (d, J = 1.4Hz, 0.29H), 5.43 (d, J = 1.3 Hz, 0.71H), 2.07 (ddd, J = 12.3, 9.4, 3.2 Hz, 1H), 1.79 – 1.70 (m, 4H), 1.55 – 1.43 (m, 6H), 1.35 (d, J = 1.8 Hz, 12H), 1.31 – 1.24 (m, 2H), 1.22 – 1.01 (m, 4H), 0.96 – 0.88 (m, 6H). ^{13}C NMR (126 MHz, Methanol-d_4) δ 158.28, 140.93, 140.59, 128.99, 128.76, 128.59, 128.43, 127.11, 125.63, 124.58, 118.68, 118.42, 117.13, 116.95, 111.85, 111.75, 89.02, 88.42, 84.54, 84.50, 80.06, 79.75, 57.39, 56.31, 43.97, 43.37, 43.31, 43.11, 39.34, 39.26, 38.97, 34.74, 34.67, 34.30, 33.95, 28.72, 26.72, 25.44, 25.30, 25.25, 25.23, 21.99, 21.93, 21.44, 21.19, 20.74, 20.15, 19.69. HRMS (ESI): m/z calculated for $C_{30}H_{43}BNO_3$ $[M+H]^+$: 476.3336, Found $[M+H]^+$: 476.3334.

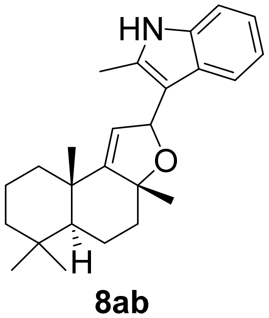

8ab

2-methyl-3-((3aR,5aS,9aS)-3a,6,6,9a-tetramethyl-2,3a,4,5,5a,6,7,8,9,9a-decahydronaphtho[2,1-b]furan-2-yl)-1H-indole (8ab): Method A: **4** (50 mg, 0.20 mmol), 2-methyl-1H-indole (21.0 mg, 0.16 mmol), $TiCl_4$ (0.16 mL, 0.16 mmol) and CH_2Cl_2 (10 mL) were used to afford the desired product **8ab** as white solid (50.2 mg, 86% yield, Isomer ratio: 78:22). ^{1}H NMR (500 MHz, $CDCl_3$) δ 8.00 (d, J = 17.9 Hz, 1H), 7.74 – 7.68 (m, 1H), 7.21 – 7.16 (m, 1H), 7.10 – 6.99 (m, 2H), 6.04 (d, J = 1.3 Hz, 0.78H), 6.00 (d, J = 1.3Hz, 0.22H), 5.46 (d, J = 1.3Hz, 0.22H), 5.37 (d, J = 1.3 Hz, 1H), 2.34 (d, J = 41.4 Hz, 3H), 2.04 (dt, J = 11.7, 2.8 Hz, 1H), 1.87 – 1.69 (m, 4H), 1.59 – 1.43 (m, 7H), 1.29 – 1.02 (m, 5H), 0.94 (d, J = 27.6 Hz, 6H). ^{13}C NMR (126 MHz, $CDCl_3$) δ 157.24, 135.64, 133.64, 127.79, 121.01, 120.94, 119.59, 119.44, 119.29, 117.75, 116.25, 111.11, 110.38, 86.62, 78.18, 77.03, 56.48, 55.04, 42.69, 42.39, 42.31, 41.68, 38.50, 38.29, 38.24, 37.94, 33.90, 33.81, 33.73, 33.57, 27.77, 26.29, 21.69, 21.62, 20.71, 20.33, 19.48, 18.80, 18.73, 11.98, 11.88. HRMS (ESI): m/z calculated for $C_{25}H_{34}NO$ $[M+H]^+$: 364.2640, Found $[M+H]^+$: 364.2626.

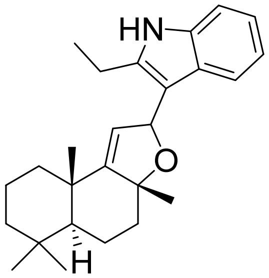

8ac

2-ethyl-3-((3aR,5aS,9aS)-3a,6,6,9a-tetramethyl-2,3a,4,5,5a,6,7,8,9,9a-decahydronaphtho[2,1-b]furan-2-yl)-1H-indole (8ac): Method A: **4** (50 mg, 0.20 mmol), 2-ethyl-1H-indole (23.2 mg, 0.16 mmol), $TiCl_4$ (0.16mL, 0.16 mmol) and CH_2Cl_2 (10 mL) were used to afford the desired product **8ac** as white solid (43.6 mg, 72% yield, Isomer ratio: 85:15). ^{1}H NMR (500 MHz, $CDCl_3$) δ 7.88 (d, *J* = 11.7 Hz, 1H), 7.72 (dd, *J* = 13.4, 7.9 Hz, 1H), 7.27 (s, 1H), 7.12 – 7.00 (m, 2H), 6.03 (d, *J* = 1.2 Hz, 0.85H), 6.00 (d, J = 1.2Hz, 0.15H), 5.45 (d, J = 1.3Hz, 0.15H), 5.36 (d, *J* = 1.2 Hz, 1H), 2.87 (qd, *J* = 7.5, 2.3 Hz, 2H), 2.09 – 1.99 (m, 1H), 1.88 – 1.68 (m, 4H), 1.56-1.43 (m, 6H), 1.30 (t, *J* = 7.6 Hz, 3H), 1.26 – 1.16 (m, 6H), 0.93 (d, *J* = 28.8 Hz, 6H). ^{13}C NMR (126 MHz, $CDCl_3$) δ 157.28, 139.34, 135.64, 127.81, 121.09, 119.90, 119.71, 119.33, 117.96, 110.47, 86.58, 57.26, 55.04, 43.50, 42.67, 42.32, 41.63, 39.23, 38.54, 38.30, 37.92, 33.81, 33.75, 33.59, 28.42, 26.21, 21.69, 20.74, 20.34, 19.52, 18.74, 14.72. HRMS (ESI): *m*/*z* calculated for $C_{26}H_{36}NO$ $[M+H]^+$: 378.2797, Found $[M+H]^+$: 378.2793.

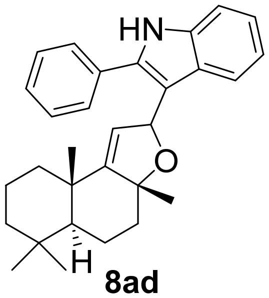

8ad

2-phenyl-3-((3aR,5aS,9aS)-3a,6,6,9a-tetramethyl-2,3a,4,5,5a,6,7,8,9,9a-decahydronaphtho[2,1-b]furan-2-yl)-1H-indole (8ad): Method A: **4** (50 mg, 0.20 mmol), 2-Phenylindole (30.9 mg, 0.16 mmol), $TiCl_4$ (0.16 mL, 0.16 mmol) and CH_2Cl_2 (10 mL) were used to afford the desired product **8ad** as white solid (56.0 mg, 82% yield, Isomer ratio: 82:18). ^{1}H NMR (500 MHz, $CDCl_3$) δ 8.32 – 8.18 (m, 1H), 7.90 (dd, *J* = 13.6, 8.0 Hz, 1H), 7.66 – 7.57 (m, 2H), 7.46 – 7.35 (m, 3H), 7.32 (d, *J* = 7.8 Hz, 1H), 7.22 – 7.07 (m, 2H), 6.06 (d, *J* = 1.3 Hz, 0.82H), 5.95 (d, J = 1.3Hz, 0.18H), 5.45 (d, J = 1.2Hz, 0.18H), 5.42 (s, 0.82H), 2.10 – 2.03 (m, 1H), 1.88 (td, *J* = 12.9, 4.3 Hz, 1H), 1.81 (ddd, *J* = 14.2, 9.2, 2.7 Hz, 1H), 1.71 (dd, *J* = 14.5, 3.5 Hz, 2H), 1.58 – 1.44 (m, 6H), 1.32 – 1.10 (m, 5H), 1.02 – 0.88 (m, 6H). ^{13}C NMR (126 MHz, $CDCl_3$) δ 157.26, 155.49, 137.53, 136.38, 132.58, 129.13, 128.98, 128.77, 128.12, 128.07, 122.26, 122.17, 121.03, 120.85, 119.79, 117.81, 116.43, 112.13, 111.06, 87.76, 86.68, 78.46, 56.50, 54.91, 42.66, 42.38, 42.30, 41.65, 38.53, 38.21, 37.93, 33.88, 33.81, 33.75, 33.55, 27.75, 26.13, 21.69, 21.61, 20.74, 20.34, 20.31, 19.45, 18.78, 18.71. HRMS (ESI): *m*/*z* calculated for $C_{30}H_{36}NO$ $[M+H]^+$: 426.2797, Found $[M+H]^+$: 426.2794.

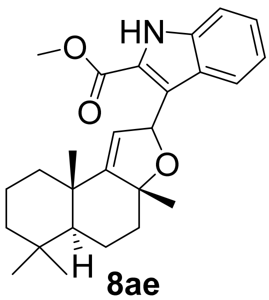

8ae

methyl 3-((3aR,5aS,9aS)-3a,6,6,9a-tetramethyl-2,3a,4,5,5a,6,7,8,9,9a-decahydronaphtho [2,1-b]furan-2-yl)-1H-indole-2-carboxylate (8ae): Method A: **4** (50 mg, 0.20 mmol), methyl indole-2-carboxylate (28.0 mg, 0.16 mmol), $TiCl_4$ (0.16 mL, 0.16 mmol) and CH_2Cl_2 (10 mL) were used to afford the desired product **8ae** as yellow oil (41.2 mg, 63% yield, Isomer ratio: 67:33). ^{1}H NMR (500 MHz, $CDCl_3$) δ 9.74 (d, *J* = 9.1 Hz, 1H), 7.98 (t, *J* = 8.2 Hz, 1H), 7.88 (dd, *J* = 7.6, 1.1 Hz, 1H), 7.29 (d, *J* = 2.4 Hz, 0.67H), 7.23 (d, J = 2.5Hz, 0.33H), 7.18 – 7.12 (m, 1H), 6.06 (d, *J* = 1.3 Hz, 0.67H), 6.03 (d, J = 1.3Hz, 0.33H), 5.49 (d, J = 1.3Hz, 0.33H), 5.38 (d, *J* = 1.3 Hz, 1H), 3.97 (d, *J* = 1.3 Hz, 3H), 2.15 – 2.03 (m, 1H), 1.82 – 1.69 (m, 4H), 1.64 (ddd, *J* = 24.3, 12.4, 3.2 Hz, 1H), 1.54 – 1.43 (m, 6H), 1.33 – 1.14 (m, 5H), 1.07 (ddd, *J* = 43.5, 12.2, 2.3 Hz, 1H), 0.95 – 0.87 (m, 6H). ^{13}C NMR (126 MHz, $CDCl_3$) δ 168.03, 157.55, 156.85, 136.86, 136.65, 127.65, 127.47, 125.61, 125.47, 124.49, 123.98, 123.36, 119.08, 118.96, 118.93, 117.91, 117.18, 115.46, 112.60, 112.55, 87.71, 87.26, 78.27, 77.91, 56.03, 55.41, 51.98, 42.75, 42.49, 42.28, 42.21, 38.27, 38.24, 38.18, 37.92, 33.85, 33.81, 33.64, 33.56, 28.80, 26.51, 21.65, 21.59, 20.50, 20.36, 20.13, 19.88, 18.75, 18.69. HRMS (ESI): *m/z* calculated for $C_{26}H_{34}NO_3$ $[M+H]^+$: 408.2539, Found $[M+H]^+$: 3408.2533.

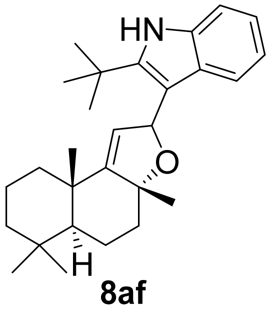

8af

2-(tert-butyl)-3-((3aR,5aS,9aS)-3a,6,6,9a-tetramethyl-2,3a,4,5,5a,6,7,8,9,9a-decahydronaphth o[2,1-b]furan-2-yl)-1H-indole (8af): Method A: **4** (50 mg, 0.20 mmol), 2-(tert-butyl)indole (27.7 mg, 0.16 mmol), $TiCl_4$ (0.16 mL, 0.16 mmol) and CH_2Cl_2 (10 mL) were used to afford the desired product **8af** as white solid (22.6 mg, 35% yield, Isomer ratio: 87:13). ^{1}H NMR (500 MHz, Methanol-*d*$_4$) δ 10.12 (s, 1H), 7.77 – 7.66 (m, 1H), 7.31 (d, *J* = 8.0 Hz, 1H), 7.00 (ddd, *J* = 8.2, 7.0, 1.2 Hz, 1H), 6.90 (ddd, *J* = 8.1, 6.9, 1.1 Hz, 1H), 6.42 (d, *J* = 1.3 Hz, 0.87H), 6.36 (d, J = 1.3Hz, 0.13H), 5.45 (d, J = 1.4Hz, 0.13H), 5.41 (d, *J* = 1.3 Hz, 0.87H), 2.00 (dt, *J* = 11.4, 2.5 Hz, 1H), 1.89 – 1.74 (m, 4H), 1.70 – 1.52 (m, 6H), 1.50 (s, 9H), 1.33 – 1.02 (m, 6H), 1.01 – 0.91 (m, 6H). ^{13}C NMR (126 MHz, Methanol-*d*$_4$) δ 157.57, 146.81, 136.71, 129.68, 121.52, 121.09, 119.57, 119.45, 111.99, 109.32, 87.68, 79.14, 56.12, 43.42, 42.21, 39.35, 39.01, 34.63, 34.08, 31.77, 26.27,

21.96, 21.19, 20.86, 19.60. HRMS (ESI): *m*/*z* calculated for $C_{28}H_{40}NO$ $[M+H]^+$: 406.3110, Found $[M+H]^+$: 406.3108.

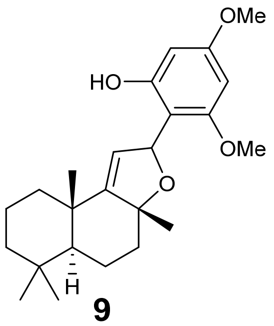

9

3,5-dimethoxy-2-((3aR,5aS,9aS)-3a,6,6,9a-tetramethyl-2,3a,4,5,5a,6,7,8,9,9a-decahydronaphtho[2,1-b]furan-2-yl)phenol (9): Method A: **4** (50 mg, 0.20 mmol), 3,5-dimethoxyphenol (24.7 mg, 0.16 mmol), $TiCl_4$ (0.16 mL, 0.16 mmol) and CH_2Cl_2 (10 mL) were used to afford the desired product **9** as transparent oil (49.8 mg, 81% yield, Isomer ratio: 87:13). ^{1}H NMR (500 MHz, $CDCl_3$) δ 9.96 (s, 0.13H), 9.86 (s, 0.87H), 6.14 (d, J = 1.3 Hz, 0.87H), 6.05 (d, J = 1.3Hz, 0.13H), 6.03 – 5.98 (m, 2H), 5.53(d, J = 1.3Hz, 0.13H), 5.30 (d, J = 1.3 Hz, 0.87H), 3.76 (d, J = 6.7 Hz, 6H), 2.21 – 2.02 (m, 1H), 1.79 – 1.60 (m, 4H), 1.59 – 1.34 (m, 7H), 1.29 – 0.92 (m, 5H), 0.88 – 0.78 (m, 6H). ^{13}C NMR (126 MHz, $CDCl_3$) δ 160.51, 157.75, 157.70, 155.82, 117.49, 116.51, 104.59, 94.17, 90.53, 88.70, 83.18, 81.52, 56.44, 55.56, 55.51, 55.34, 54.61, 42.29, 41.92, 41.49, 38.45, 37.87, 37.77, 37.74, 33.86, 33.68, 33.48, 33.32, 27.74, 25.63, 21.60, 21.47, 20.50, 20.12, 19.26, 18.58. HRMS (ESI): *m*/*z* calculated for $C_{24}H_{35}O_4$ $[M+H]^+$: 387.2535, Found $[M+H]^+$: 387.2528.

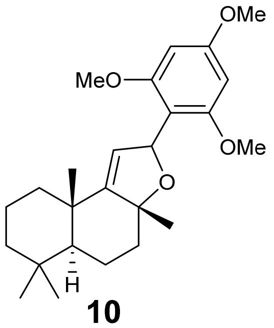

10

(3aR,5aS,9aS)-3a,6,6,9a-tetramethyl-2-(2,4,6-trimethoxyphenyl)-2,3a,4,5,5a,6,7,8,9,9a-decahydronaphtho[2,1-b]furan (10): Method A: **4** (50 mg, 0.20 mmol), 1,3,5-trimethoxybenzene (26.9 mg, 0.16 mmol), $TiCl_4$ (0.16 mL, 0.16 mmol) and CH_2Cl_2 (10 mL) were used to afford the desired product **10** as transparent oil (39.6 mg, 62% yield, Isomer ratio: 80:20). ^{1}H NMR (500 MHz, Methanol-d_4) δ 6.28 – 6.13 (m, 3H), 5.20 (d, J = 1.5Hz, 0.2H), 5.15 (d, *J* = 1.4 Hz, 0.8H), 3.79 (d, *J* = 1.6 Hz, 3H), 3.75 (d, *J* = 11.0 Hz, 6H), 1.95 – 1.83 (m, 2H), 1.82 – 1.68 (m, 3H), 1.57 – 1.48 (m, 3H), 1.45 (s, 3H), 1.41 – 1.21 (m, 2H), 1.19 – 0.96 (m, 4H), 0.96 – 0.87 (m, 6H). ^{13}C NMR (126 MHz, $CDCl_3$) δ 161.07, 161.02, 160.49, 160.43, 153.97, 152.60, 117.63, 116.41, 109.96, 109.78, 91.03, 90.95, 87.76, 87.11, 76.03, 75.31, 56.25, 55.64, 55.47, 55.39, 55.36, 55.24, 42.64, 42.58, 42.43, 40.97, 38.37, 38.21, 38.02, 37.72, 33.91, 33.78, 33.70, 33.52, 26.92, 21.53, 20.76,

20.44, 20.01, 19.85, 18.82, 18.77, 0.11. HRMS (ESI): *m/z* calculated for $C_{25}H_{37}O_4$ $[M+H]^+$: 401.2692, Found $[M+H]^+$: 401.2685.

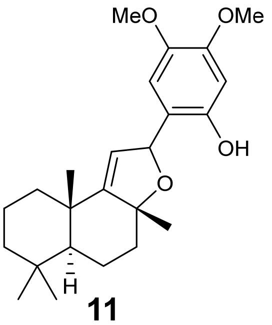

11

4,5-dimethoxy-2-((3aR,5aS,9aS)-3a,6,6,9a-tetramethyl-2,3a,4,5,5a,6,7,8,9,9a-decahydronaphtho[2,1-b]furan-2-yl)phenol (**11**): Method A: 4 (50 mg, 0.20 mmol), 3, 4-dimethoxyphenol (24.7 mg, 0.16 mmol), $TiCl_4$ (0.16 mL, 0.16 mmol) and CH_2Cl_2 (10 mL) were used to afford the desired product **11** as transparent oil (46.9 mg, 76% yield, Isomer ratio: 77:23). ^{1}H NMR (500 MHz, Methanol-d4) δ 6.87 (s, 0.23H), 6.82 (s, 0.77H), 6.44 (d, J = 1.7 Hz, 1H), 6.02 (d, J = 1.3 Hz, 0.77H), 5.94 (d, J = 1.3Hz, 0.23H), 5.41 (d, J = 1.3Hz, 0.23H), 5.25 (d, J = 1.3 Hz, 0.77H), 3.79 – 3.72 (m, 6H), 2.15 – 2.02 (m, 1H), 1.81-1.66 (m, 4H), 1.62 – 1.55 (m, 1H), 1.55 – 1.49 (m, 4H), 1.44 (td, J = 13.5, 4.0 Hz, 2H), 1.30 – 1.15 (m, 4H), 1.02 (ddd, J = 12.1, 5.4, 2.5 Hz, 1H), 0.90 (s, 6H). ^{13}C NMR (126 MHz, $CDCl_3$) δ 157.57, 150.08, 149.71, 149.49, 142.27, 142.15, 117.16, 114.88, 114.69, 111.01, 101.64, 101.53, 89.56, 88.86, 84.76, 83.42, 57.03, 56.85, 55.95, 55.72, 54.82, 42.16, 42.09, 42.04, 41.87, 38.23, 38.04, 37.88, 33.83, 33.73, 33.51, 33.34, 27.85, 25.61, 21.62, 21.54, 20.23, 20.12, 20.09, 19.70, 18.65, 18.57. HRMS (ESI): m/z calculated for $C_{24}H_{35}O_4$ [M+H]+: 387.2535, Found [M+H]+: 387.2534.

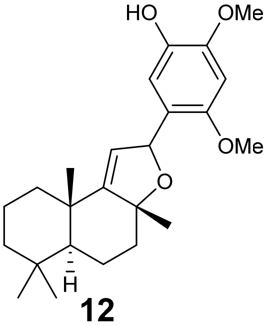

12

2,4-dimethoxy-6-((3aR,5aS,9aS)-3a,6,6,9a-tetramethyl-2,3a,4,5,5a,6,7,8,9,9a-decahydronaphtho[2,1-b]furan-2-yl)phenol (12): Method A: **4** (50 mg, 0.20 mmol), 2, 4-dimethoxyphenol (24.7 mg, 0.16 mmol), $TiCl_4$ (0.16 mL, 0.16 mmol) and CH_2Cl_2 (10 mL) were used to afford the desired product **12** as transparent oil (49.0 mg, 79% yield, Isomer ratio: 70:30). ^{1}H NMR (500 MHz, Methanol-*d*$_4$) δ 6.84 (s, 0.3H), 6.82 (d, *J* = 15.2 Hz, 1H), 6.60 (d, *J* = 1.3 Hz, 1H), 6.01 (d, *J* = 1.3 Hz, 0.7H), 5.91 (d, J = 1.3Hz, 0.3H), 5.31 (d, 1.2Hz, 0.3H), 5.16 (d, *J* = 1.2 Hz, 0.7H), 3.89 – 3.76 (m, 6H), 2.11 – 1.99 (m, 1H), 1.82 – 1.67 (m, 4H), 1.59 – 1.40 (m, 7H), 1.30 – 1.13 (m, 4H), 1.02 (ddd, *J* = 25.7, 11.9, 2.3 Hz, 1H), 0.90 (d, *J* = 3.6 Hz, 6H).^{13}C NMR (100 MHz, $CDCl_3$): HRMS (ESI): *m/z* calculated for $C_{24}H_{35}O_4$ $[M+H]^+$: 387.2535, Found $[M+H]^+$: 387.2534.

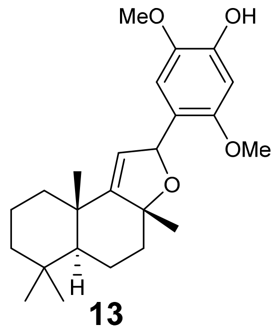

13

2,5-dimethoxy-4-((3aR,5aS,9aS)-3a,6,6,9a-tetramethyl-2,3a,4,5,5a,6,7,8,9,9a-decahydronaphtho[2,1-b]furan-2-yl)phenol (13): Method A: **4** (50 mg, 0.20 mmol), 2, 5-dimethoxyphenol (24.7 mg, 0.16 mmol), $TiCl_4$ (0.16 mL, 0.16 mmol) and CH_2Cl_2 (10 mL) were used to afford the desired product **13** as transparent oil (51.2 mg, 83% yield, Isomer ratio: 66:34). ^{1}H NMR (500 MHz, Methanol-d_4) δ 6.92 (s, 0.34H), 6.88 (s, 0.66H), 6.49 (d, J = 2.8 Hz, 1H), 5.99 (s, -0.66H), 5.91(d, J = 1.3Hz, 0.34H), 5.34 (d, J = 1.3Hz, 0.34H), 5.20 (s, 0.66H), 3.82 – 3.72 (m, 6H), 2.14 – 2.02 (m, 1H), 1.81-1.66 (m, 4H), 1.59 – 1.49 (m, 5H), 1.47 – 1.40 (m, 2H), 1.29-1.16 (m, 5H), 1.02-0.99 (m, 1H), 0.90 (s, 6H). ^{13}C NMR (126 MHz, $CDCl_3$) δ 155.83, 155.15, 150.85, 150.80, 145.24, 145.18, 140.50, 140.26, 122.81, 122.55, 117.67, 116.31, 110.18, 110.05, 99.10, 99.03, 87.88, 87.50, 80.08, 78.92, 56.76, 56.66, 56.09, 56.04, 56.00, 55.38, 43.25, 42.71, 42.27, 42.11, 38.10, 38.02, 38.00, 37.74, 33.79, 33.71, 33.52, 33.50, 29.19, 26.90, 21.56, 21.53, 20.55, 20.37, 19.98, 19.70, 18.70, 18.63. HRMS (ESI): m/z calculated for $C_{24}H_{35}O_4$ $[M+H]^+$: 387.2535, Found $[M+H]^+$: 387.2528.

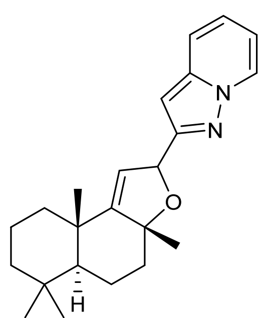

14

2-((3aR,5aS,9aS)-3a,6,6,9a-tetramethyl-2,3a,4,5,5a,6,7,8,9,9a-decahydronaphtho[2,1-b]furan-2-yl)pyrazolo[1,5-a]pyridine (14): Method A: **4** (50 mg, 0.20 mmol), pyrazolo[1,5-a]pyridine (18.9 mg, 0.16 mmol), $TiCl_4$ (0.16 mL, 0.16 mmol) and CH_2Cl_2 (10 mL) were used to afford the desired product **14** as transparent oil (46.0 mg, 82% yield, Isomer ratio: 66:34). ^{1}H NMR (500 MHz, $CDCl_3$) δ 8.44 (ddt, J = 7.1, 2.0, 1.1 Hz, 1H), 7.90 (s, 0.66H), 7.85 (s, 0.34H), 7.67 – 7.59 (m, 1H), 7.09 (ddt, J = 9.1, 6.7, 1.3 Hz, 1H), 6.74 (td, J = 6.9, 1.4 Hz, 1H), 6.04 (d, J = 1.3 Hz, 0.66H), 5.97 (d, J = 1.3Hz, 0.34H), 5.41 (d, J = 1.3Hz, 0.34H), 5.30 (d, J = 1.3 Hz, 0.66H), 2.06 (ddt, J = 15.7, 12.0, 3.2 Hz, 1H), 1.80-1.70 m, 3H), 1.69 – 1.60 (m, 1H), 1.60 – 1.53 (m, 1H), 1.52 – 1.41 (m, 5H), 1.36 – 1.12 (m, 5H), 1.04 (ddd, J = 34.0, 12.2, 2.4 Hz, 1H), 0.95 – 0.87 (m, 6H). ^{13}C NMR (126 MHz, $CDCl_3$) δ 158.17, 157.40, 141.22, 140.93, 138.24, 128.93, 128.84, 123.11,

117.48, 117.44, 116.63, 115.02, 113.36, 112.60, 111.88, 87.77, 87.50, 76.49, 76.15, 55.97, 55.70, 42.75, 42.71, 42.26, 42.19, 38.34, 38.25, 38.19, 37.98, 33.85, 33.62, 33.56, 28.86, 26.52, 21.66, 21.60, 20.45, 20.35, 20.06, 19.97, 18.74, 18.70. HRMS (ESI): *m/z* calculated for $C_{23}H_{31}N_2O$ $[M+H]^+$: 351.2436, Found $[M+H]^+$: 351.2433.

3. ^{1}H NMR and ^{13}C NMR spectrum of intermediates and final compounds

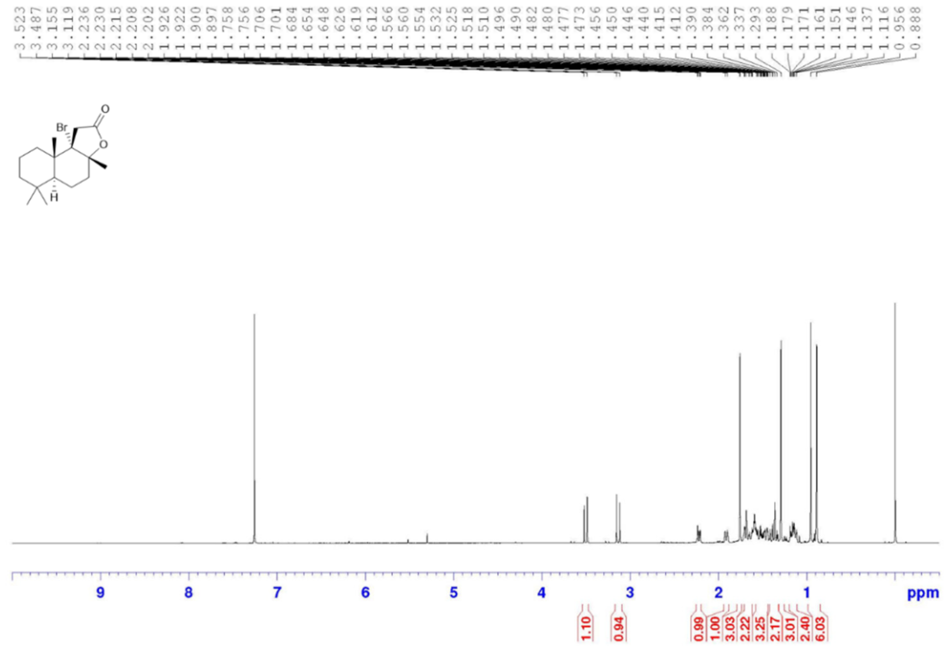

^{1}H NMR of Compound **2**

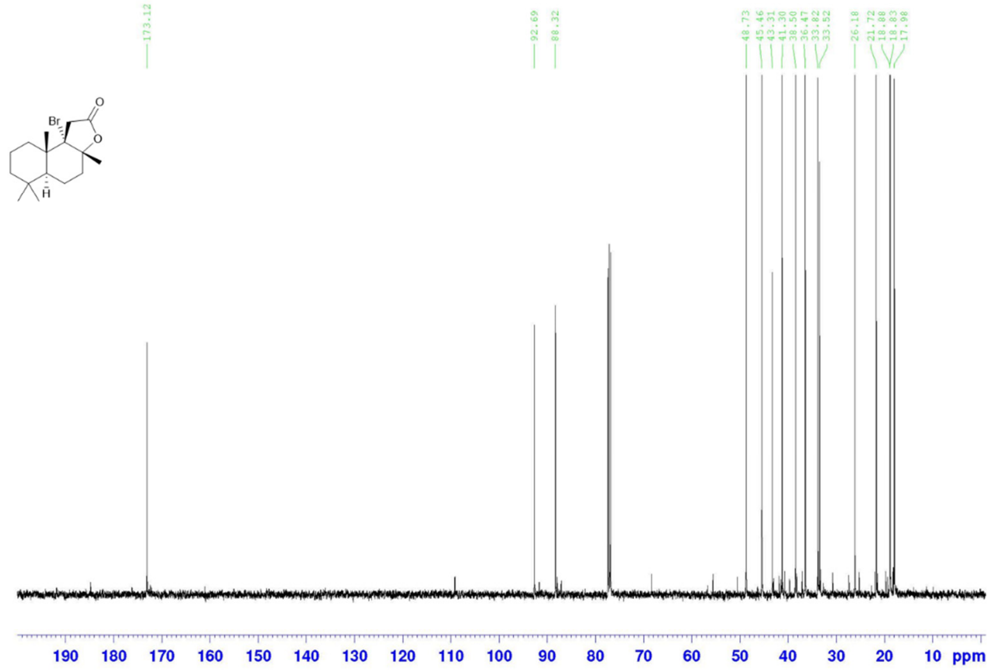

^{13}C NMR of Compound **2**

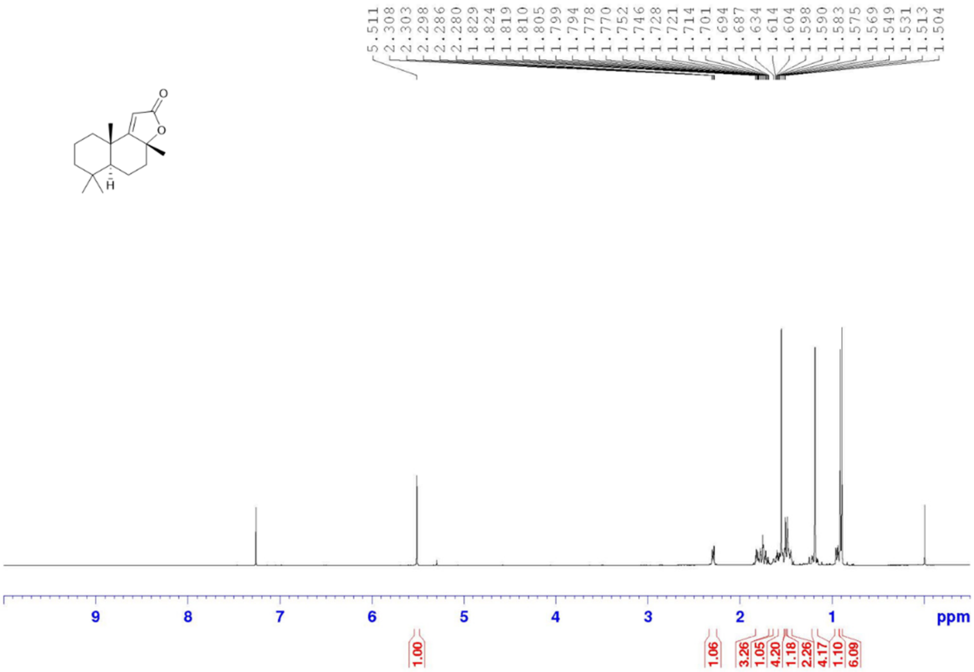

^{1}H NMR of Compound **3**

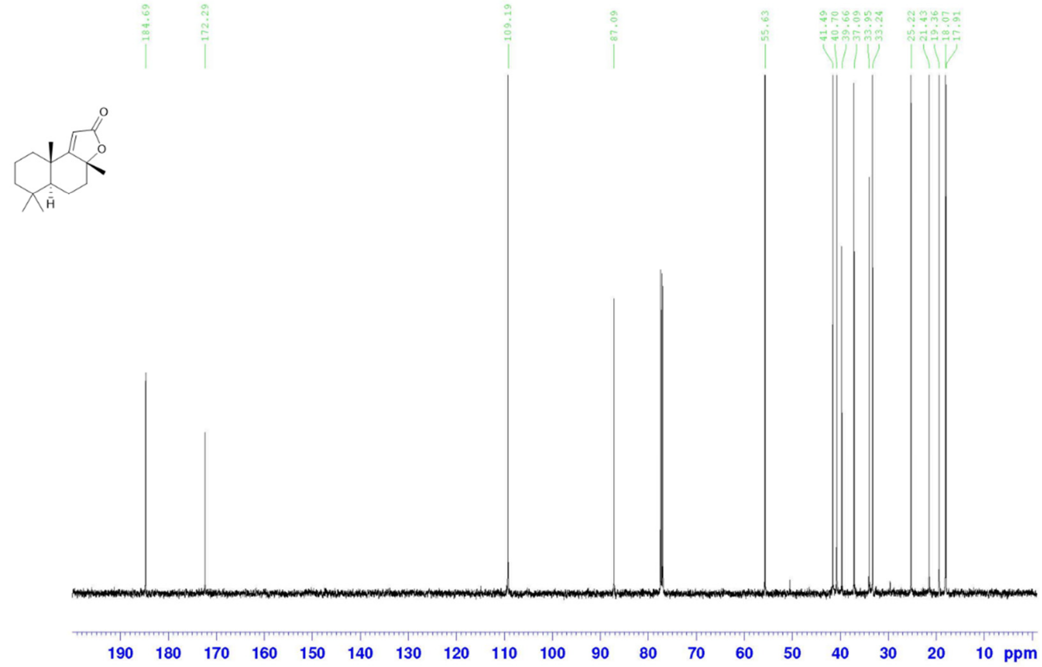

^{13}C NMR of Compound **3**

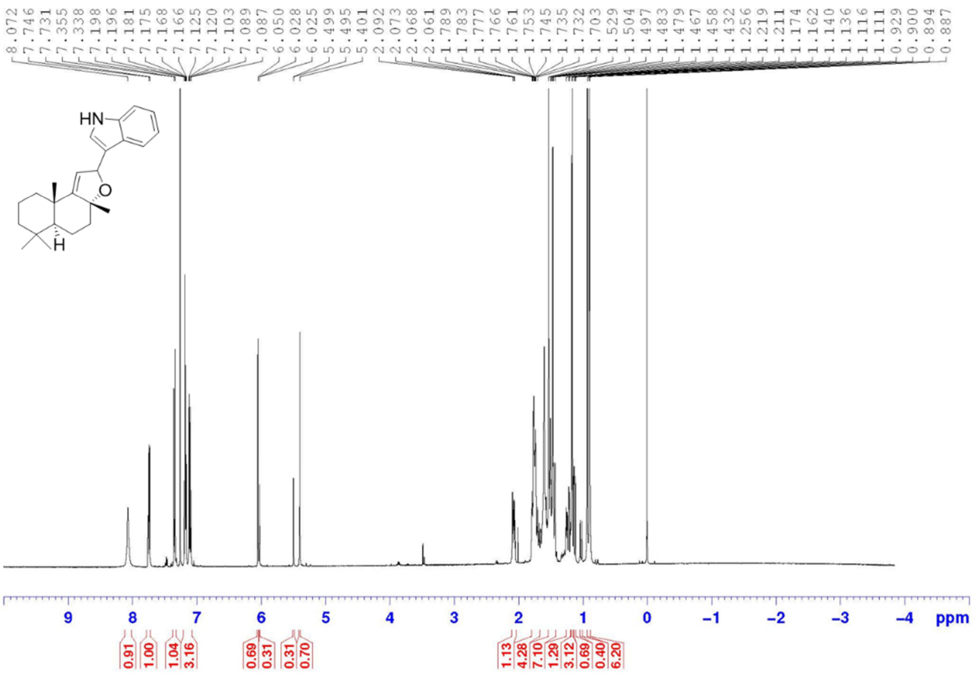

^{1}H NMR of Compound **6**

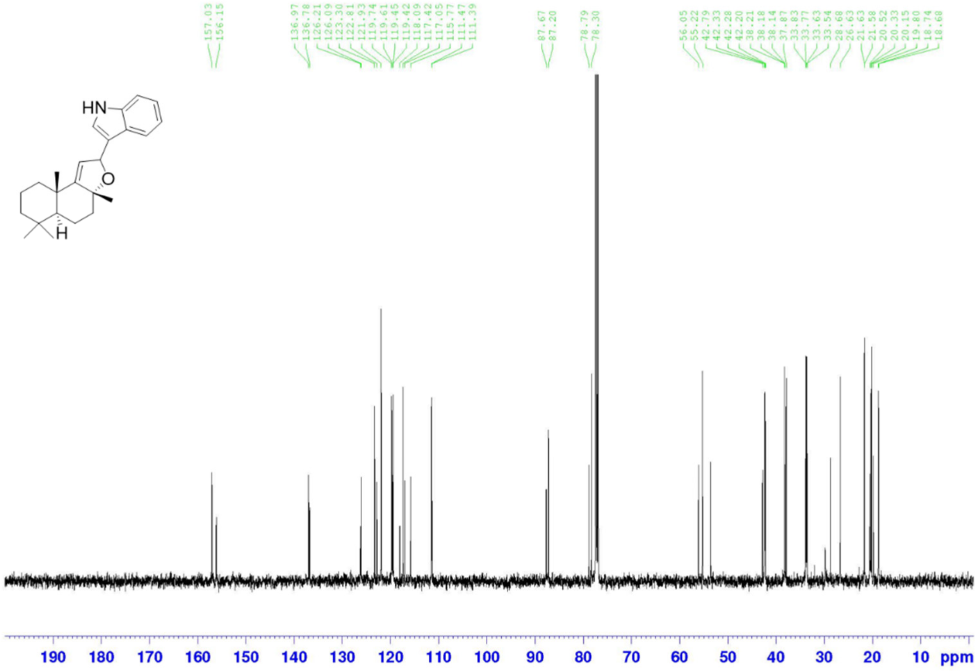

^{13}C NMR of Compound **6**

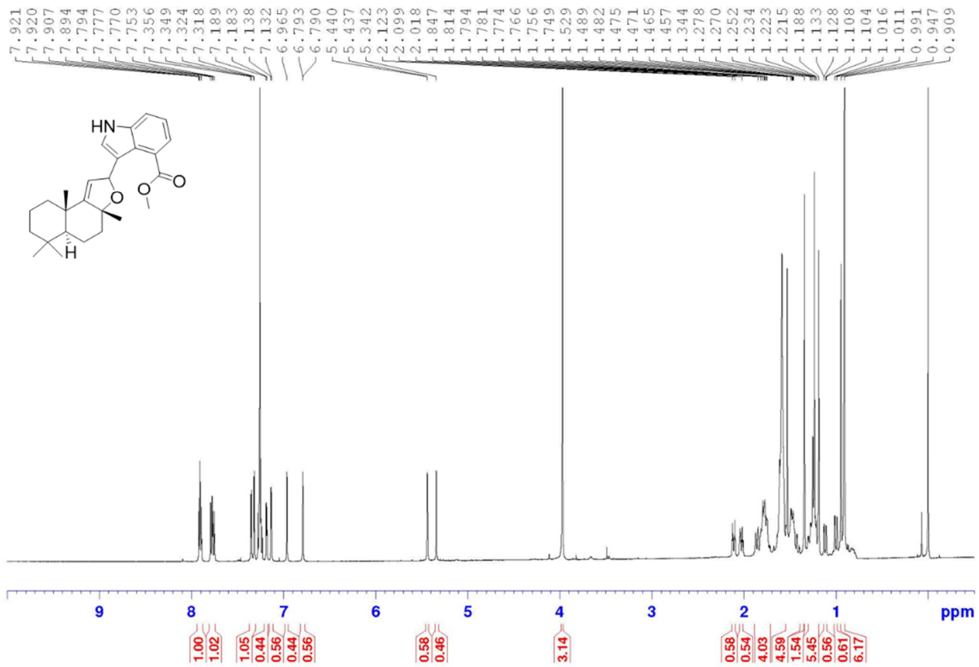

^{1}H NMR of Compound **8a**

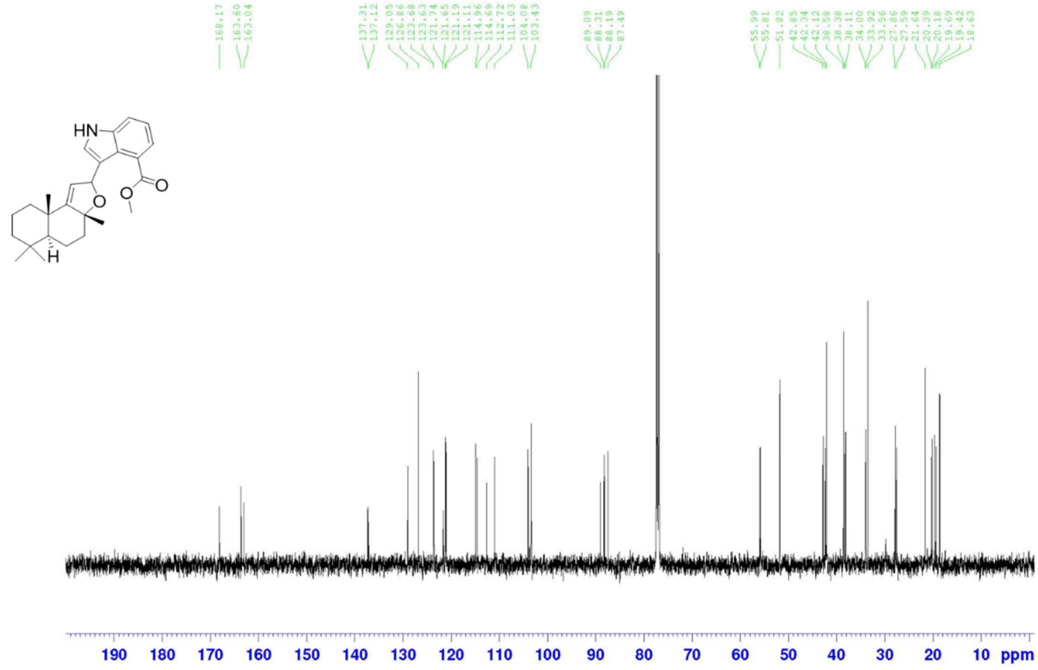

^{13}C NMR of Compound **8a**

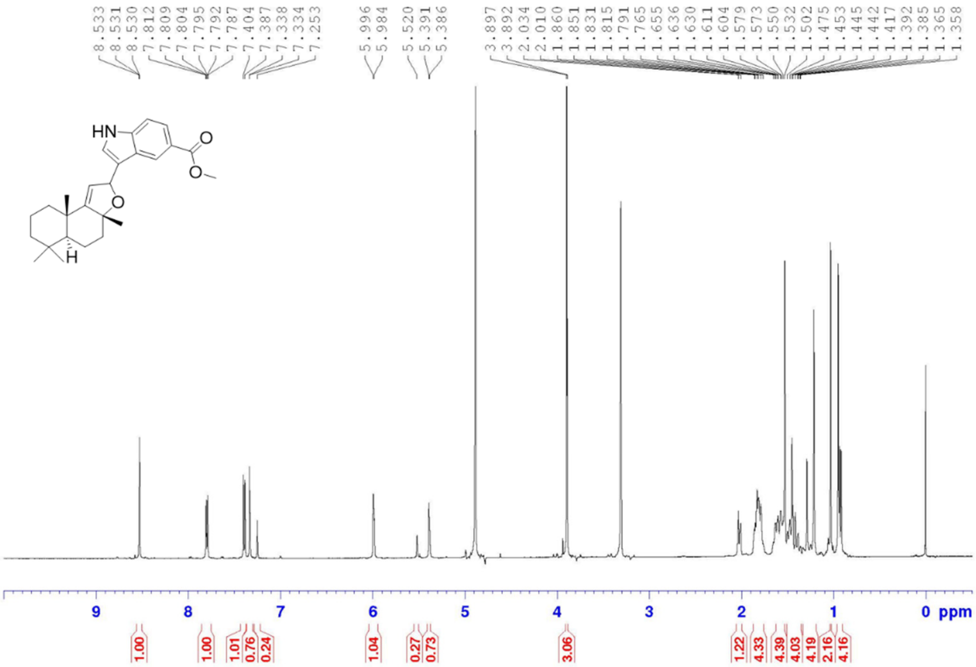

^{1}H NMR of Compound **8b**

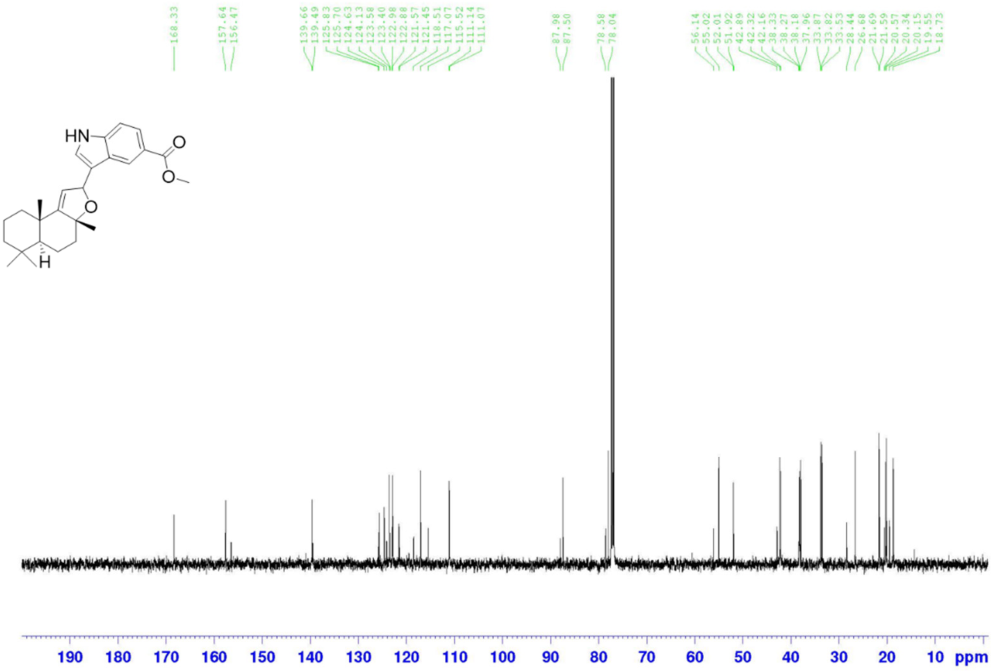

^{13}C NMR of Compound **8b**

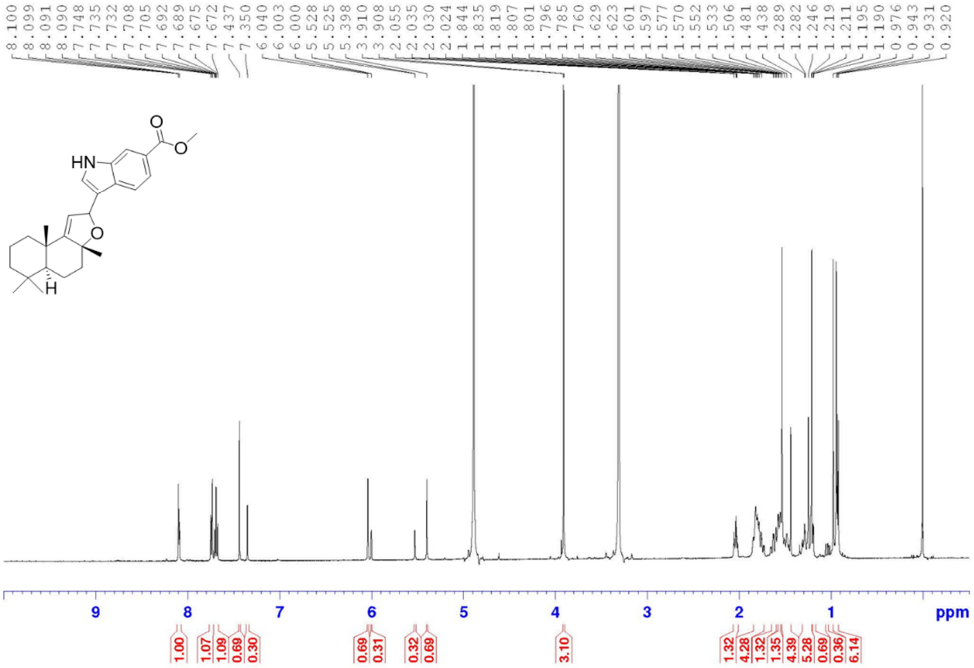

^{1}H NMR of Compound **8c**

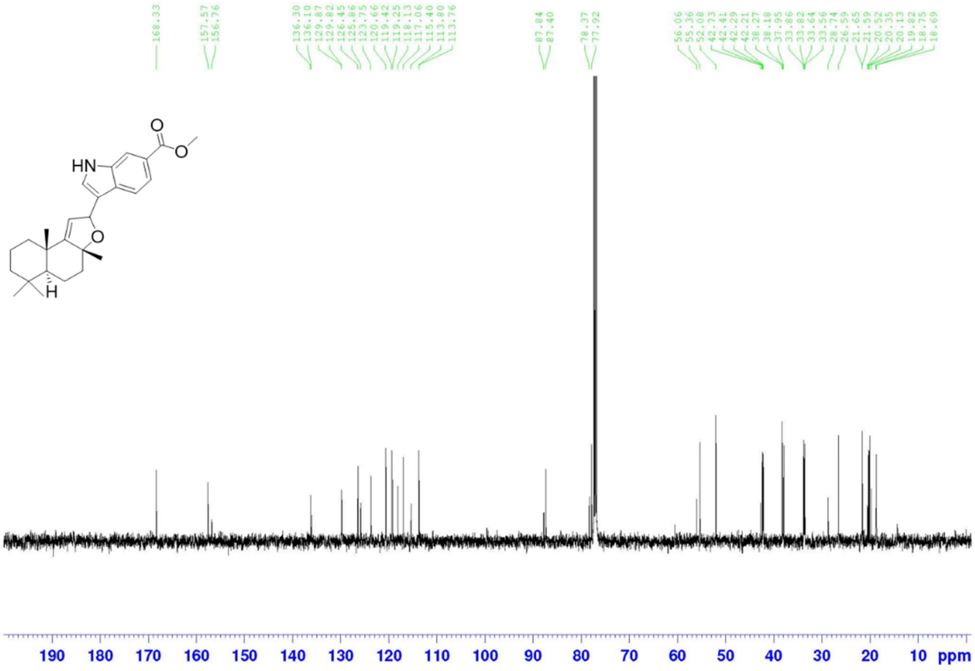

^{13}C NMR of Compound **8c**

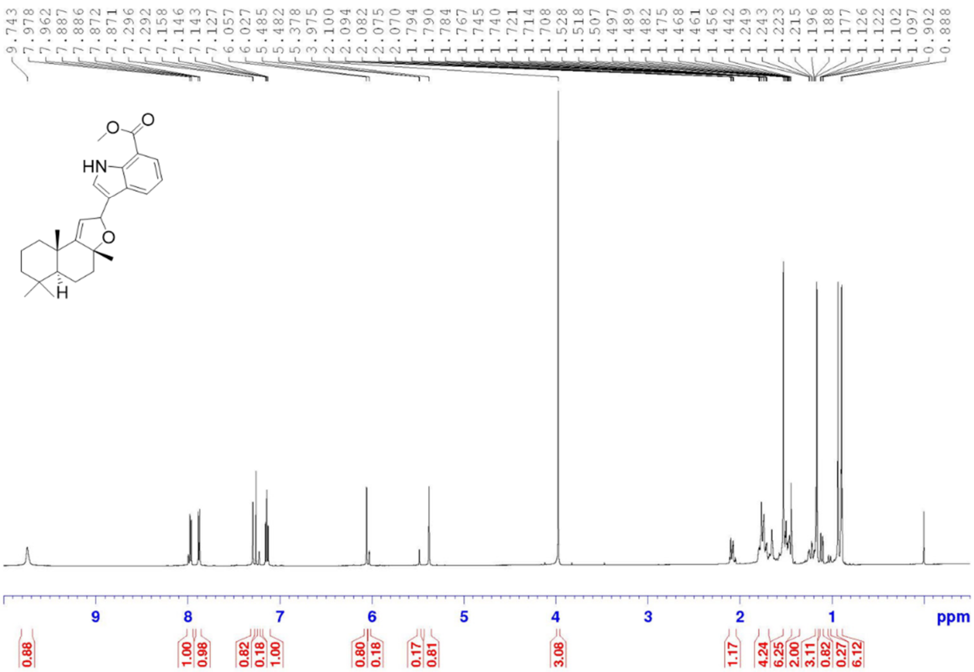

^{1}H NMR of Compound **8d**

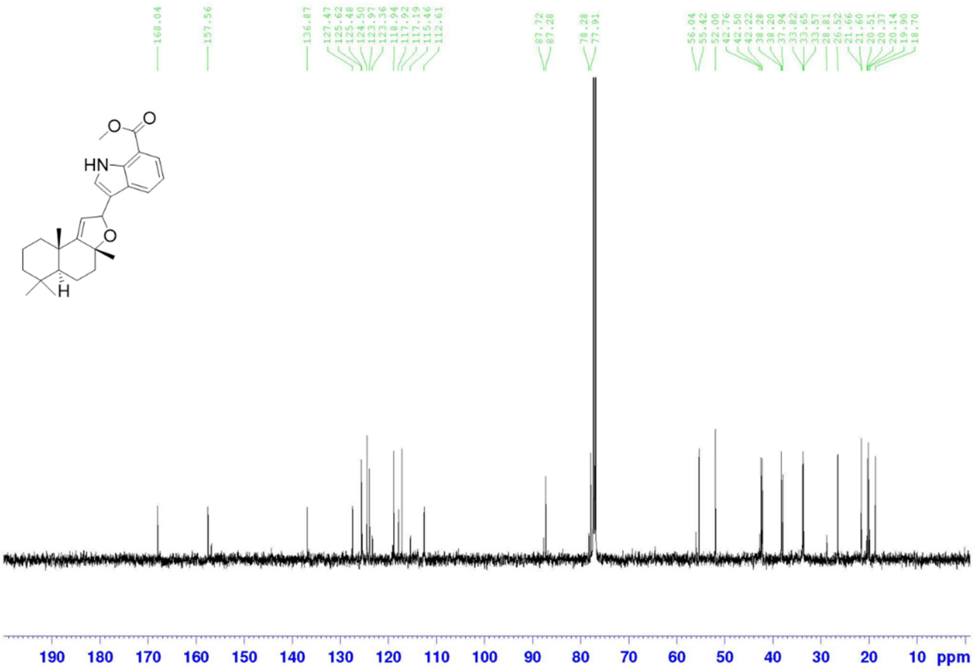

^{13}C NMR of Compound **8d**

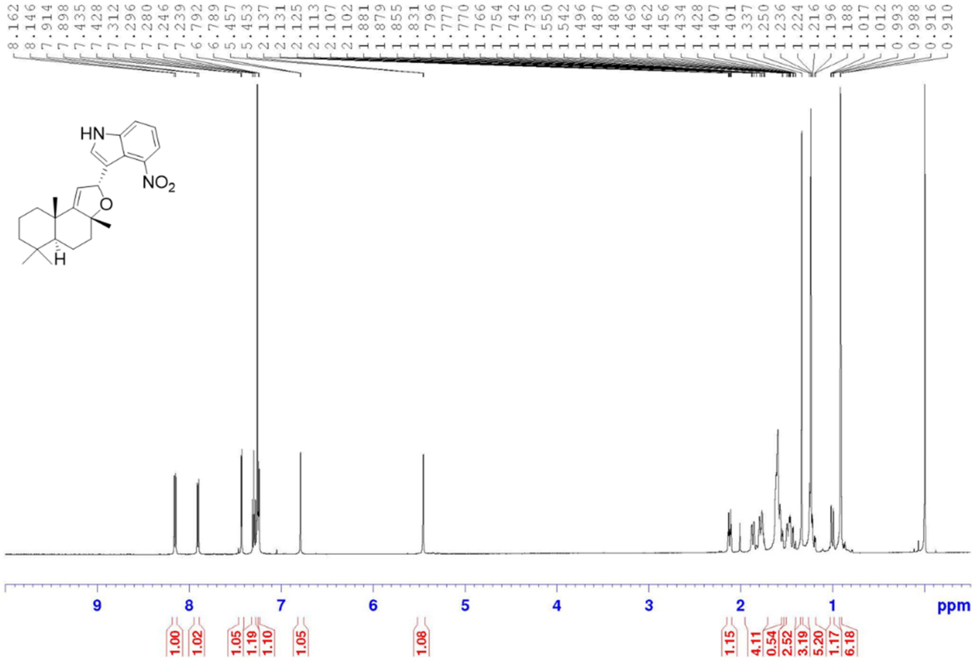

^{1}H NMR of Compound **8e**

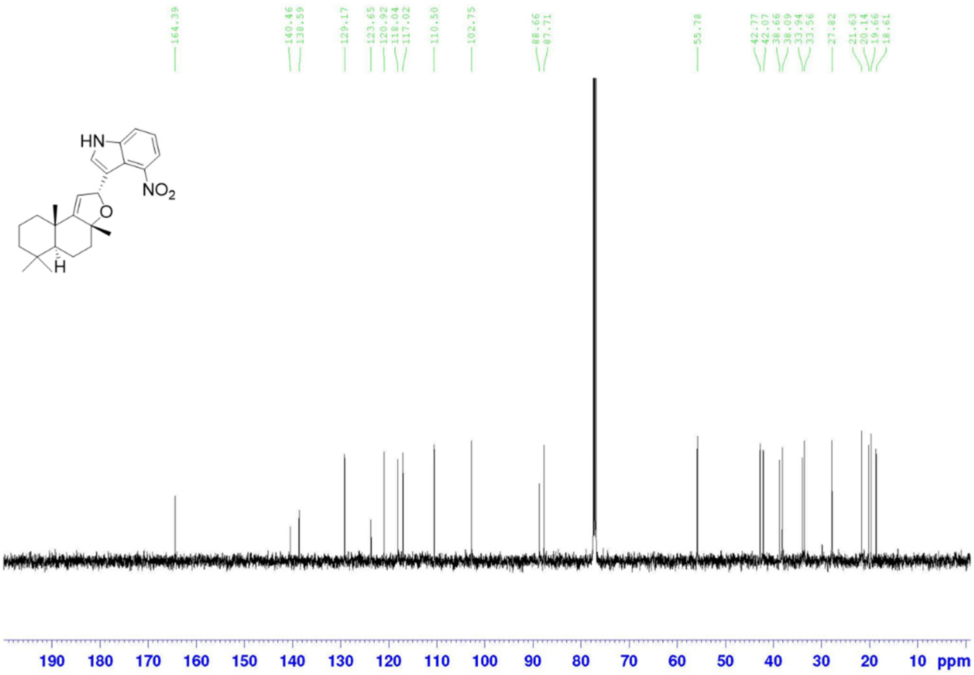

^{13}C NMR of Compound **8e**

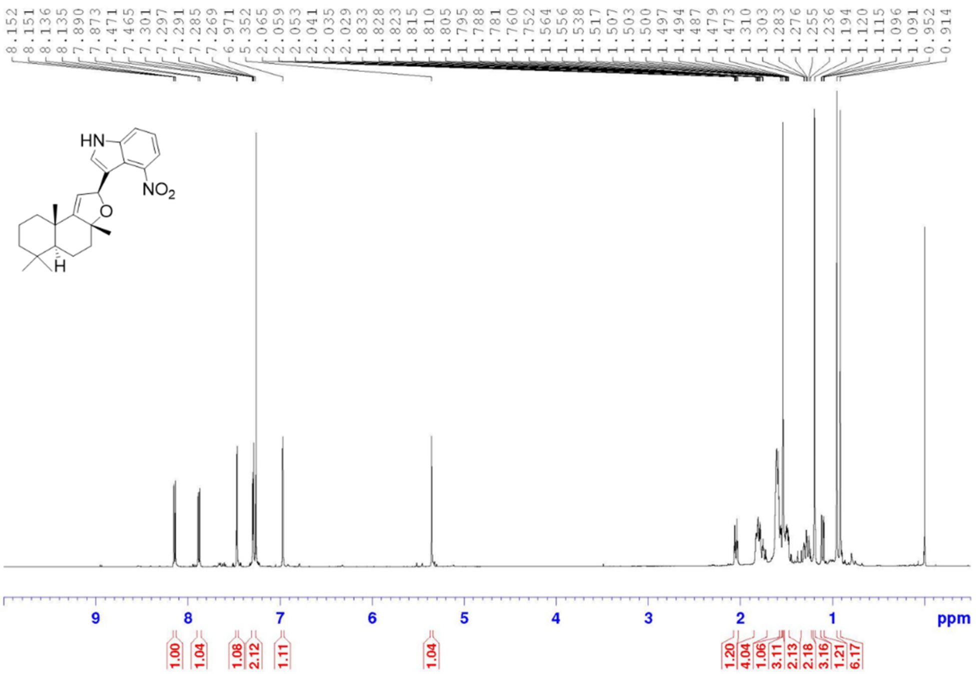

^{1}H NMR of Compound **8e'**

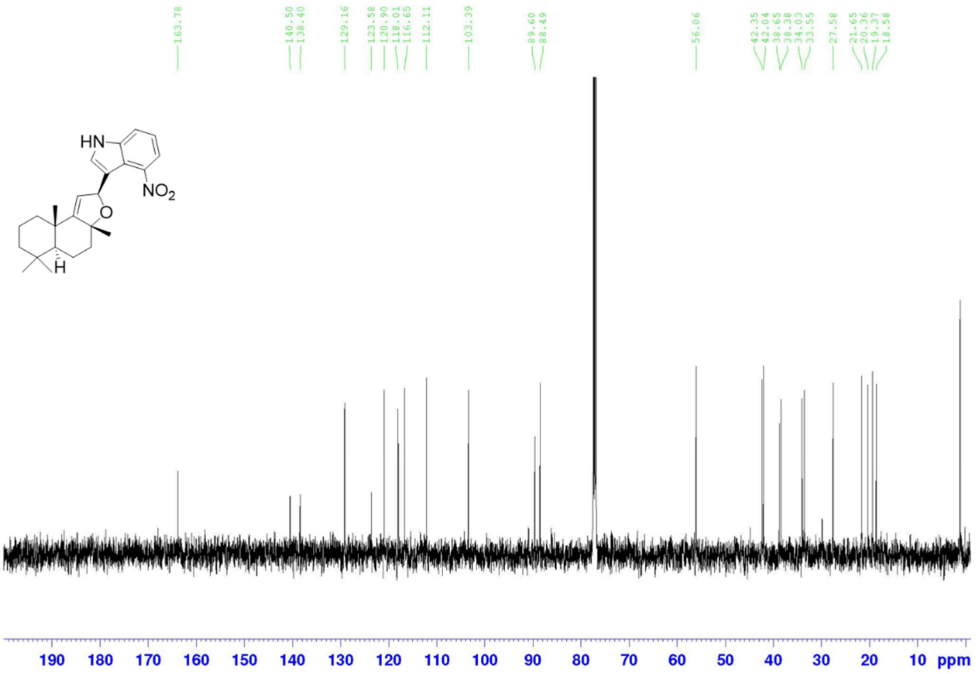

^{13}C NMR of Compound **8e'**

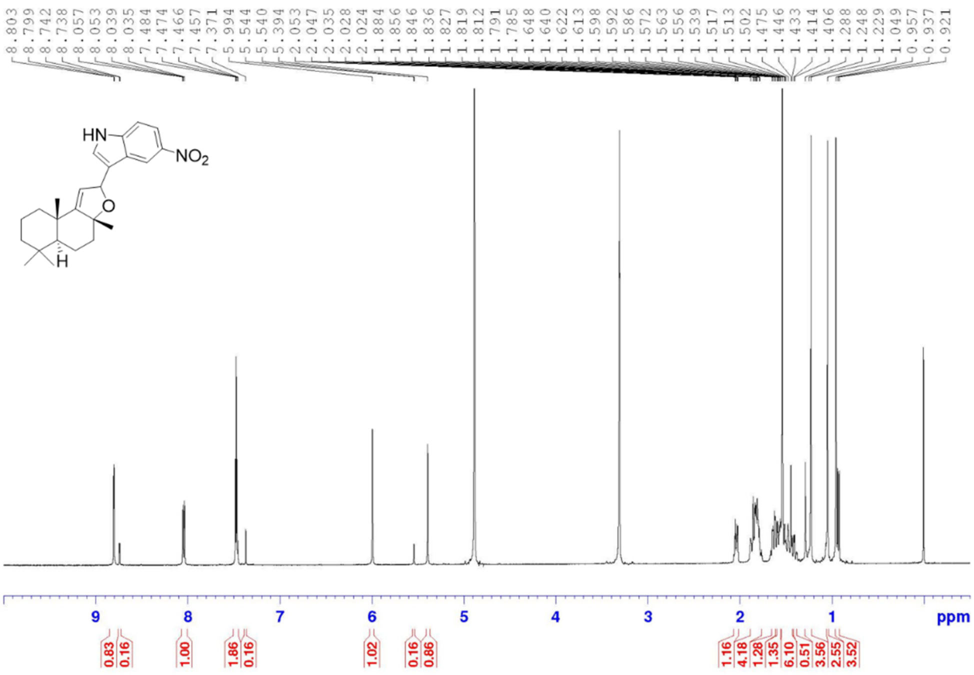

^{1}H NMR of Compound **8f**

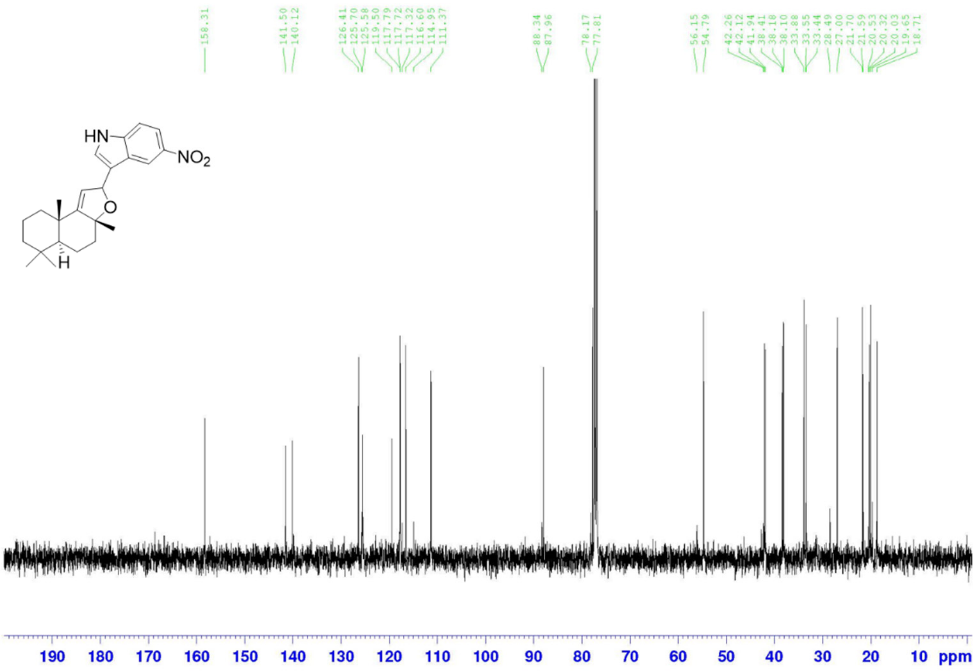

^{13}C NMR of Compound **8f**

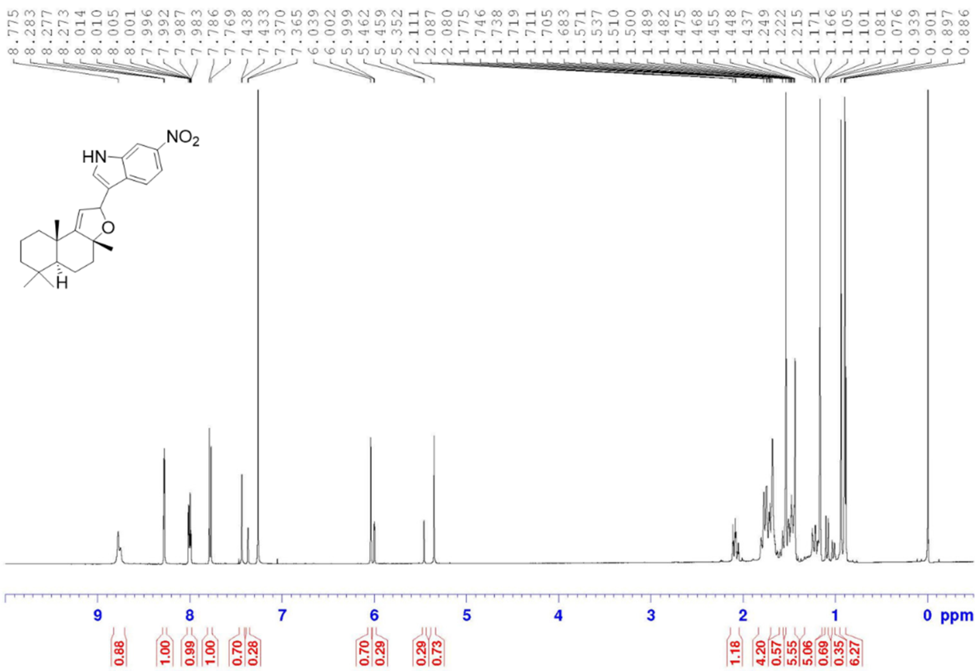

^{1}H NMR of Compound **8g**

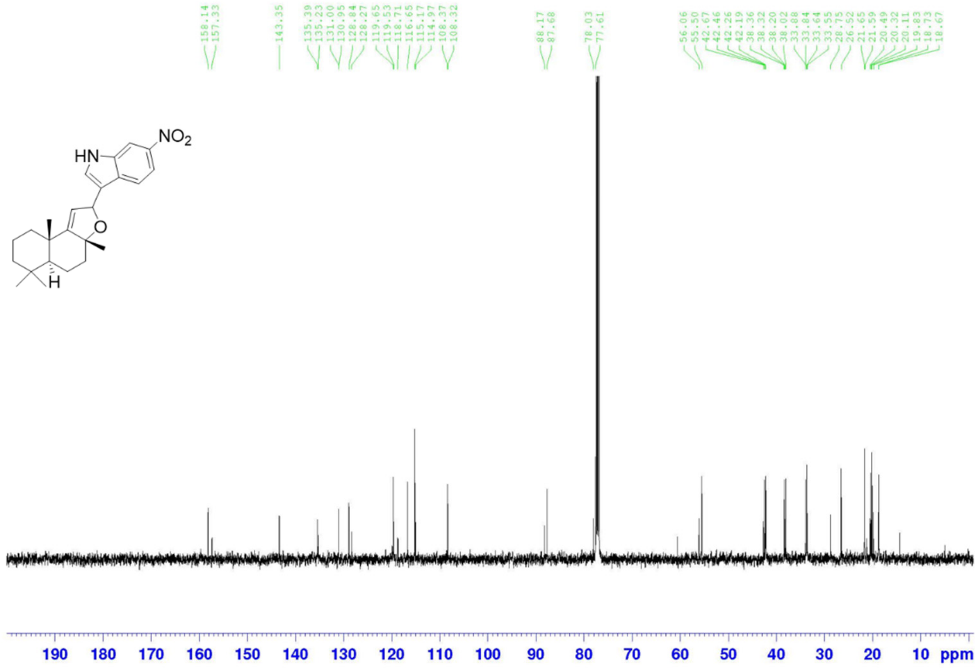

^{13}C NMR of Compound **8g**

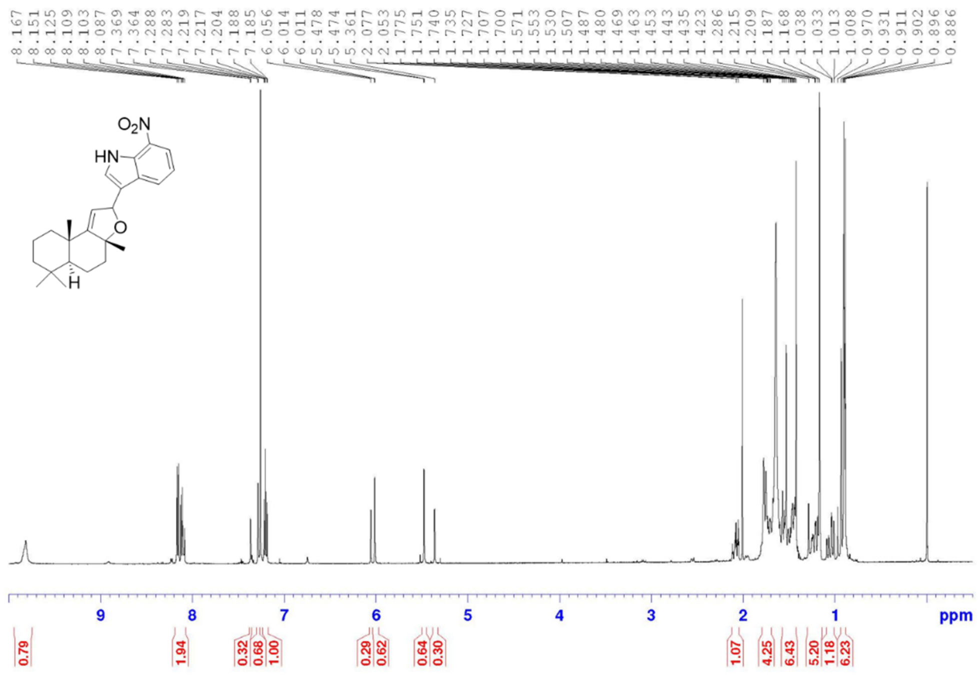

^{1}H NMR of Compound **8h**

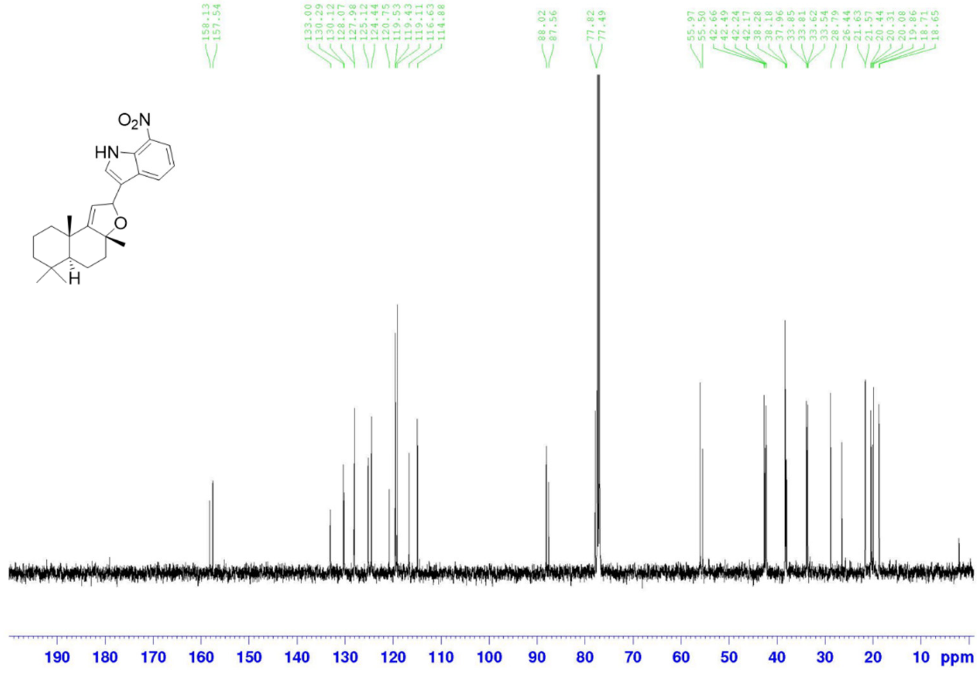

^{13}C NMR of Compound **8h**

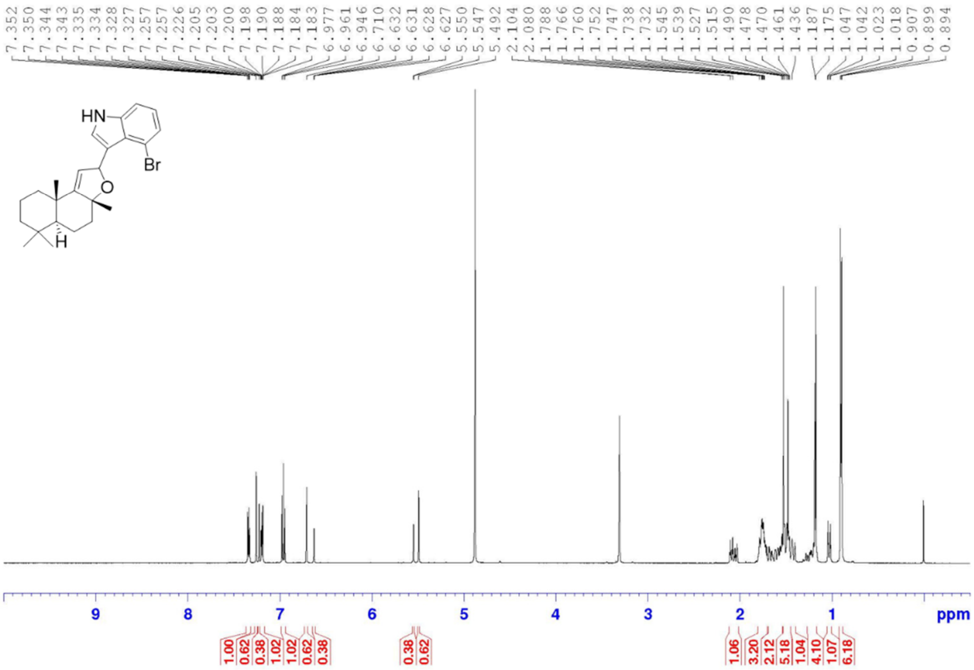

^{1}H NMR of Compound **8i**

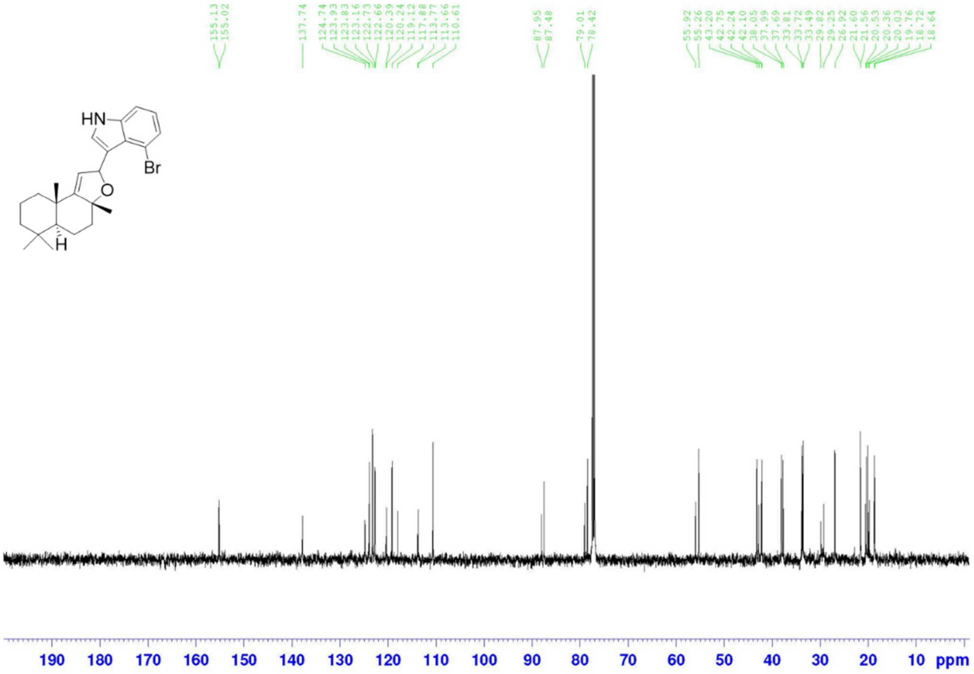

^{13}C NMR of Compound **8i**

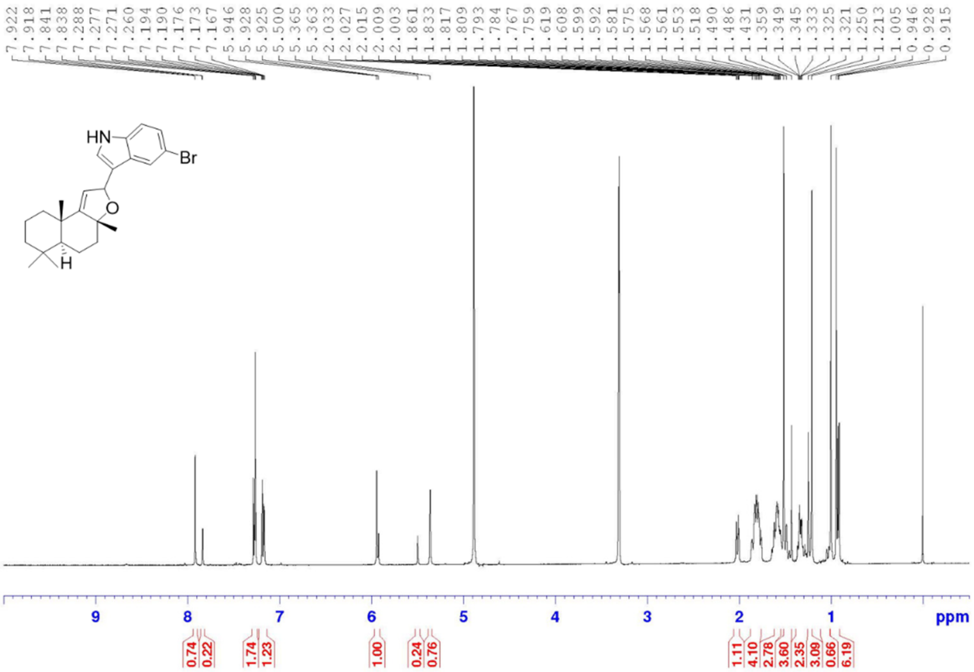

^{1}H NMR of Compound **8j**

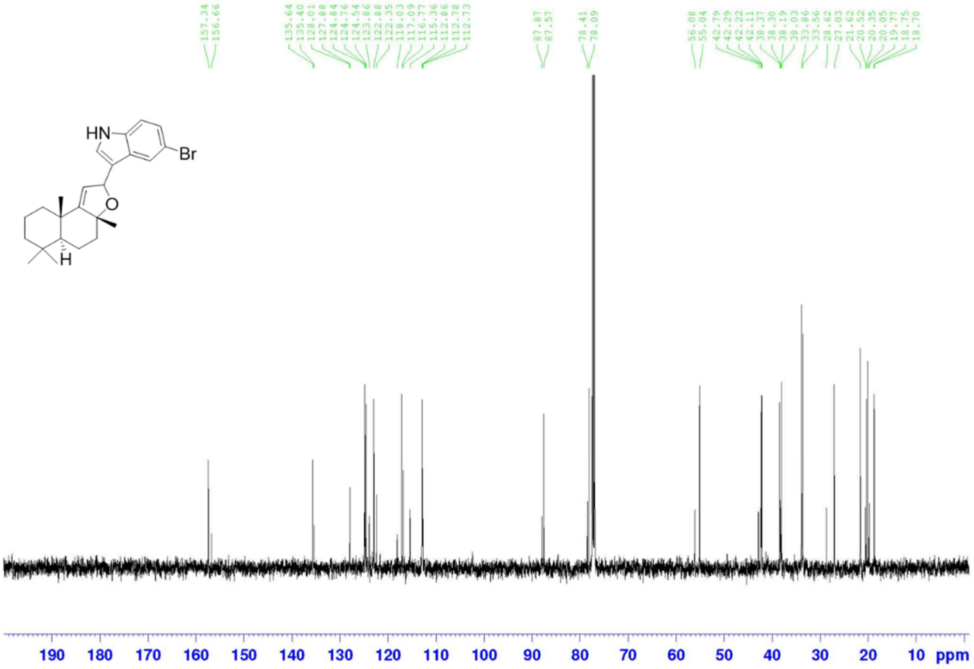

^{13}C NMR of Compound **8j**

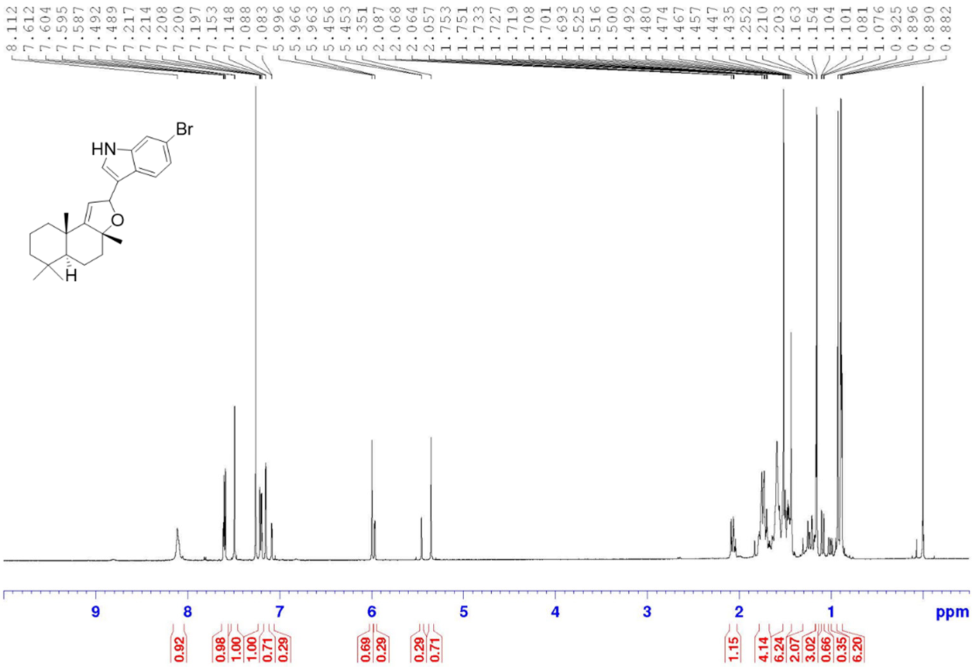

^{1}H NMR of Compound **8k**

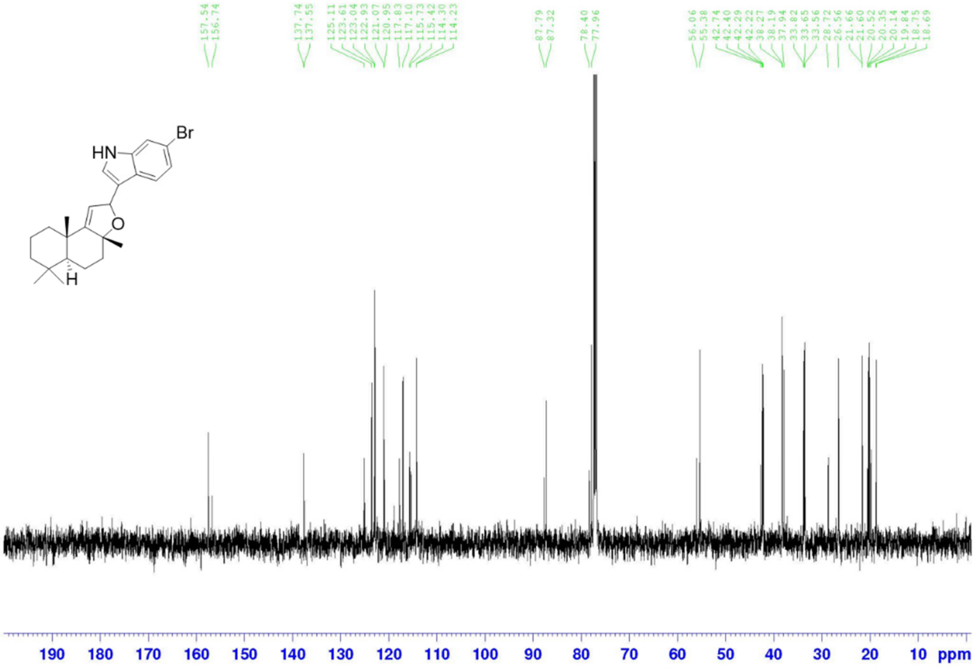

^{13}C NMR of Compound **8k**

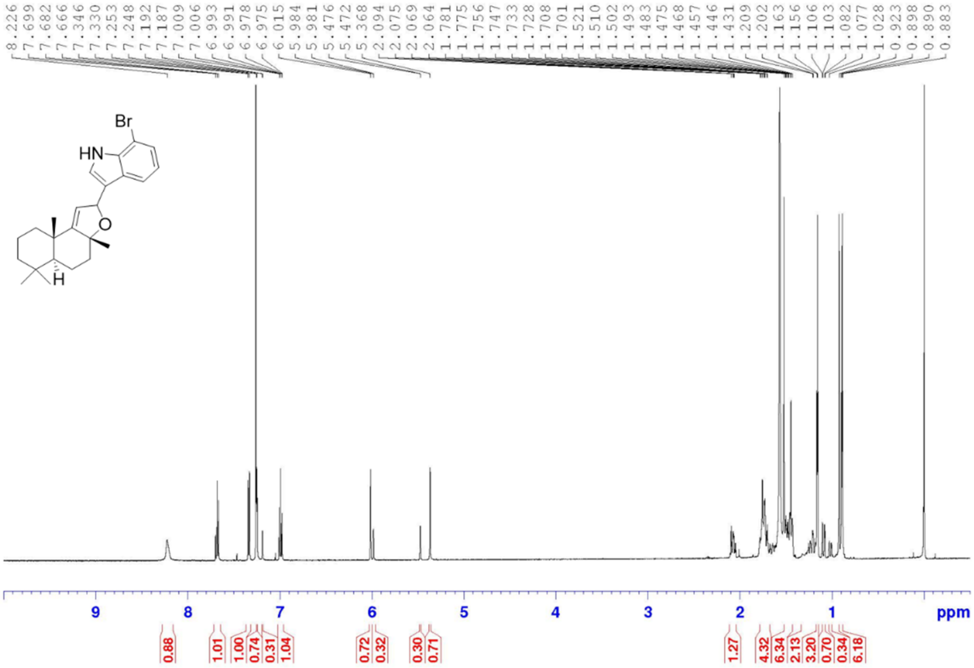

^{1}H NMR of Compound **8l**

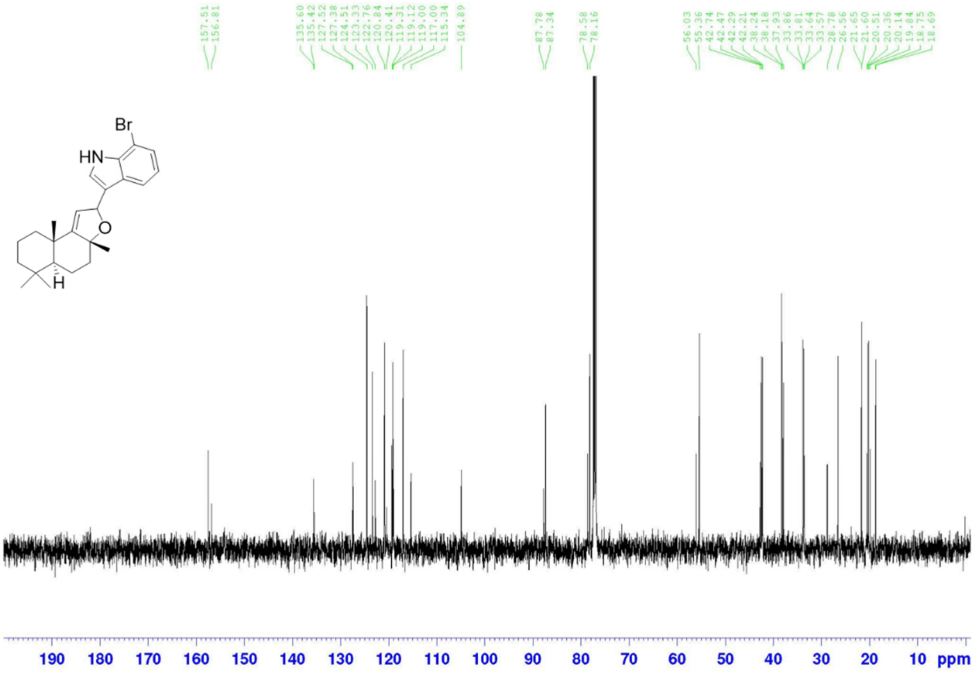

^{13}C NMR of Compound **8l**

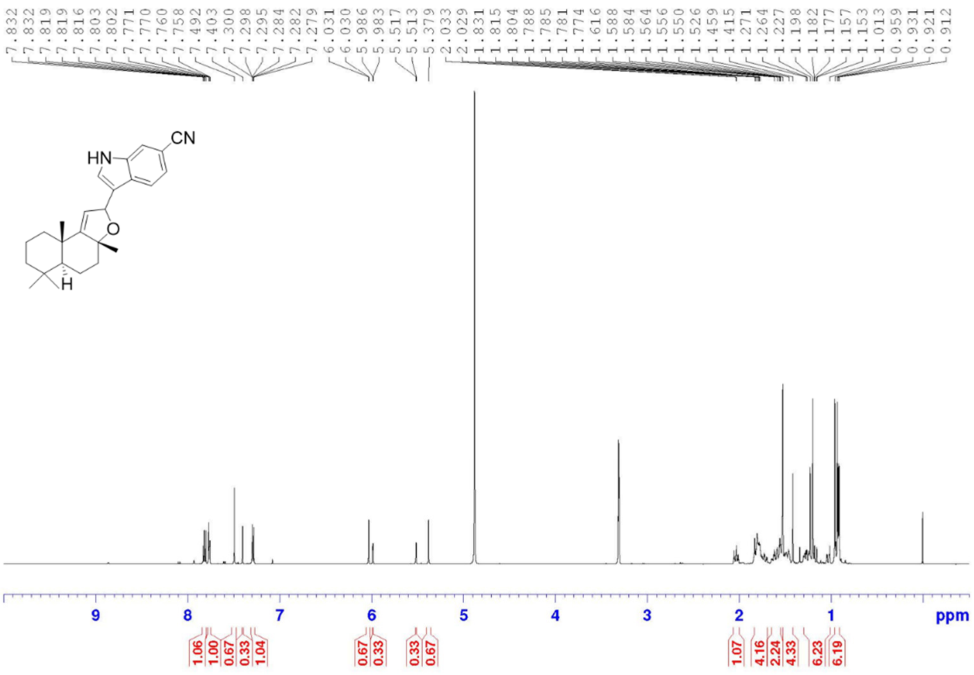

^{1}H NMR of Compound **8m**

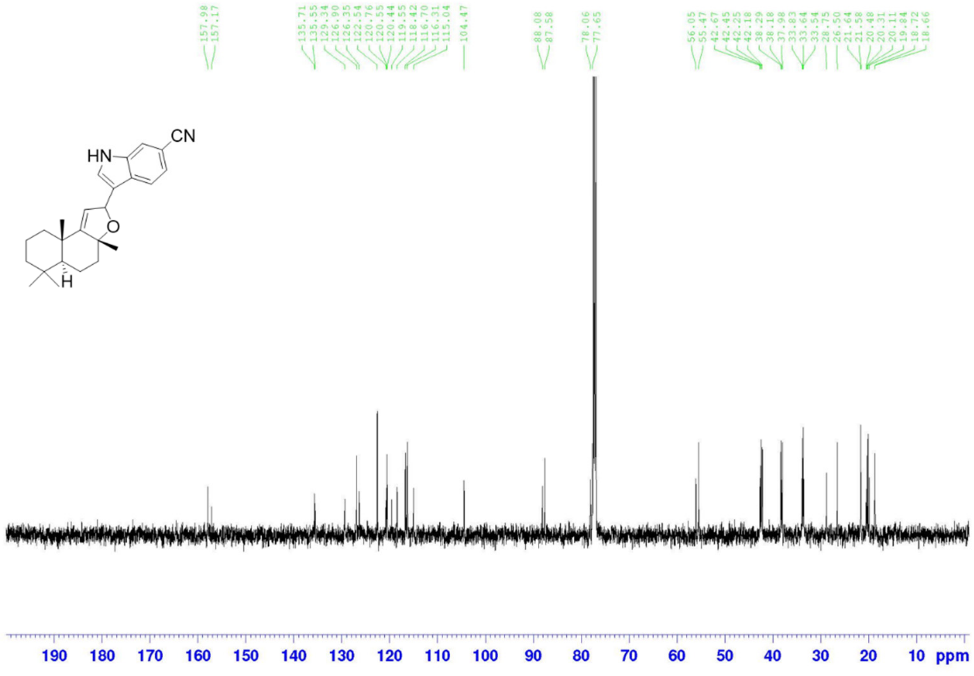

^{13}C NMR of Compound **8m**

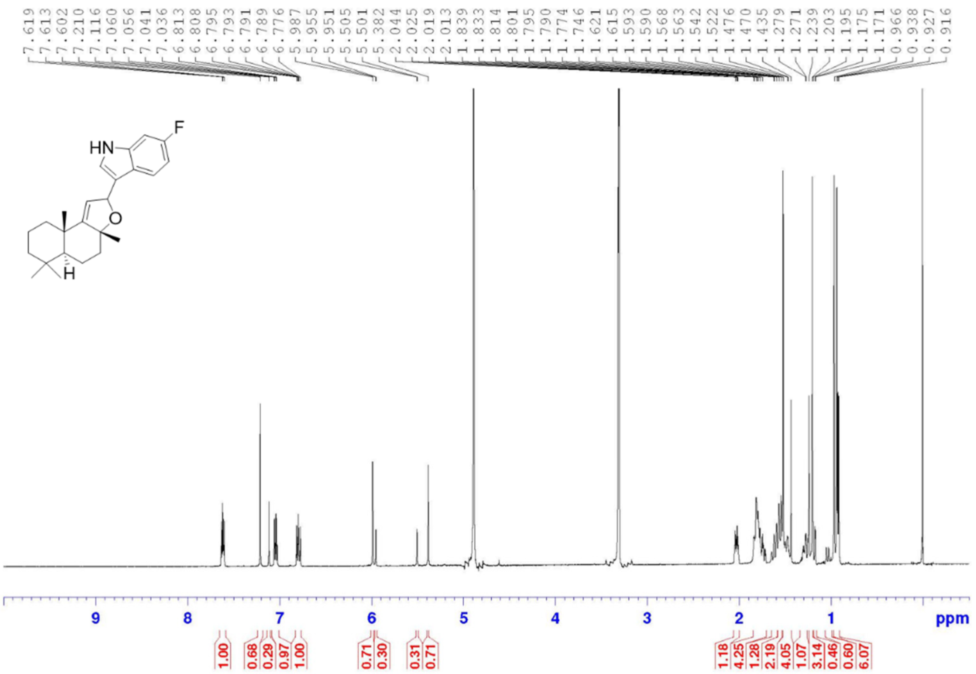

^{1}H NMR of Compound **8n**

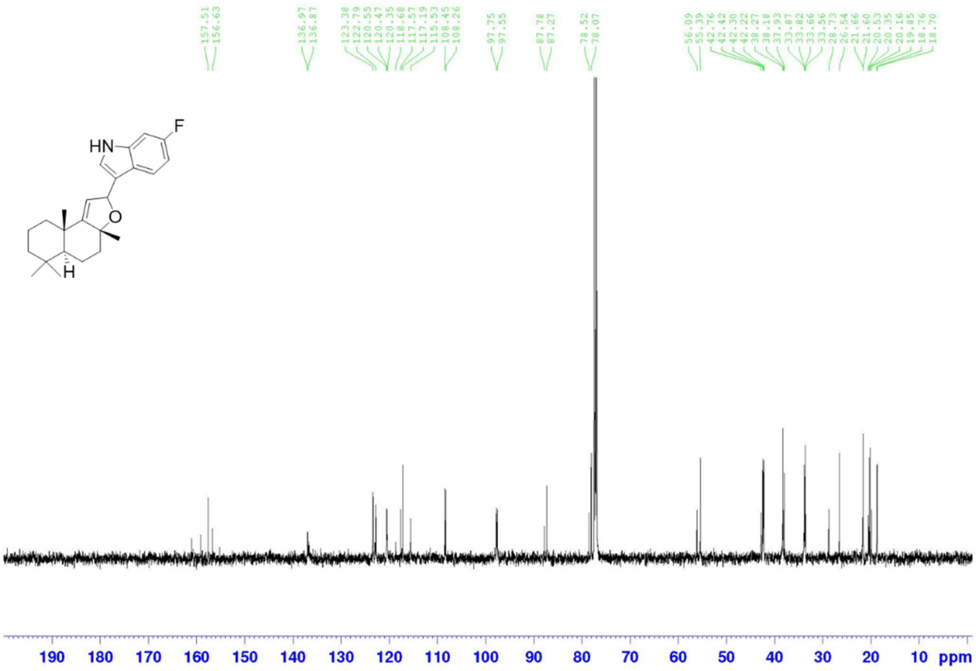

^{13}C NMR of Compound **8n**

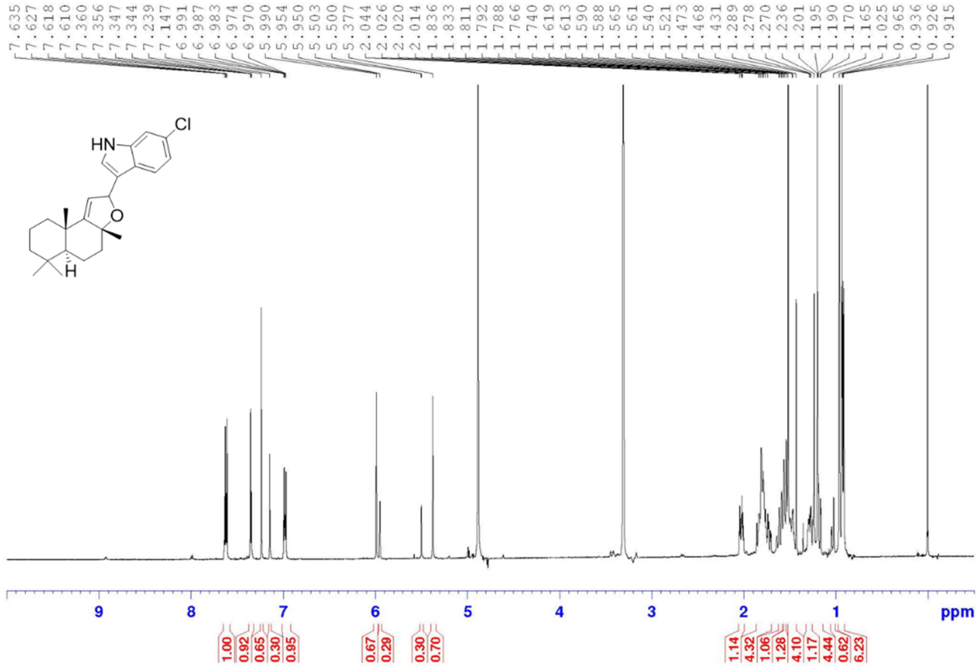

^{1}H NMR of Compound **8p**

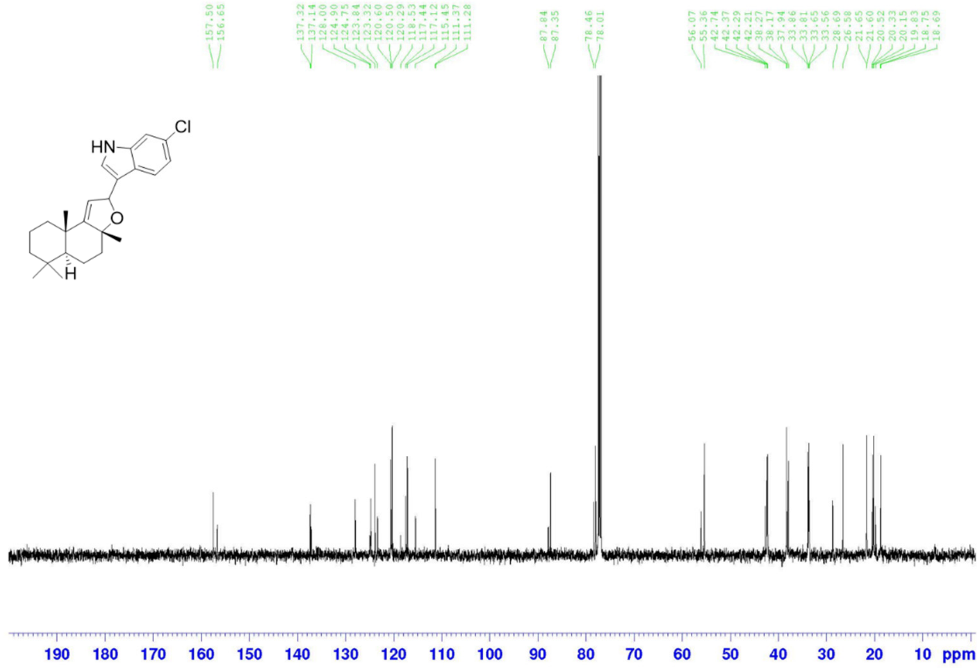

^{13}C NMR of Compound **8p**

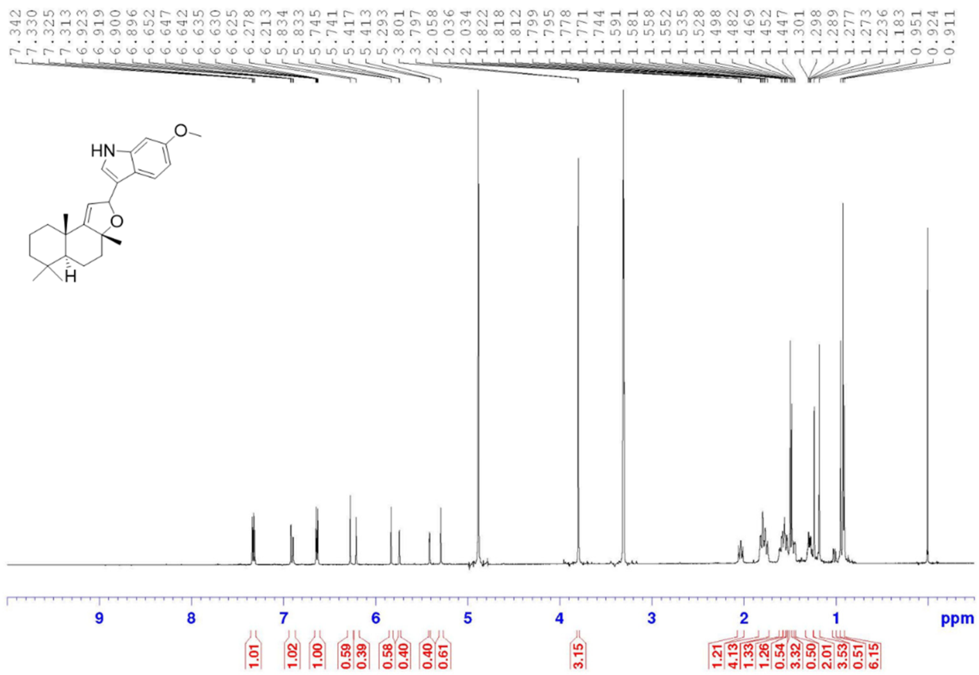

^{1}H NMR of Compound **8q**

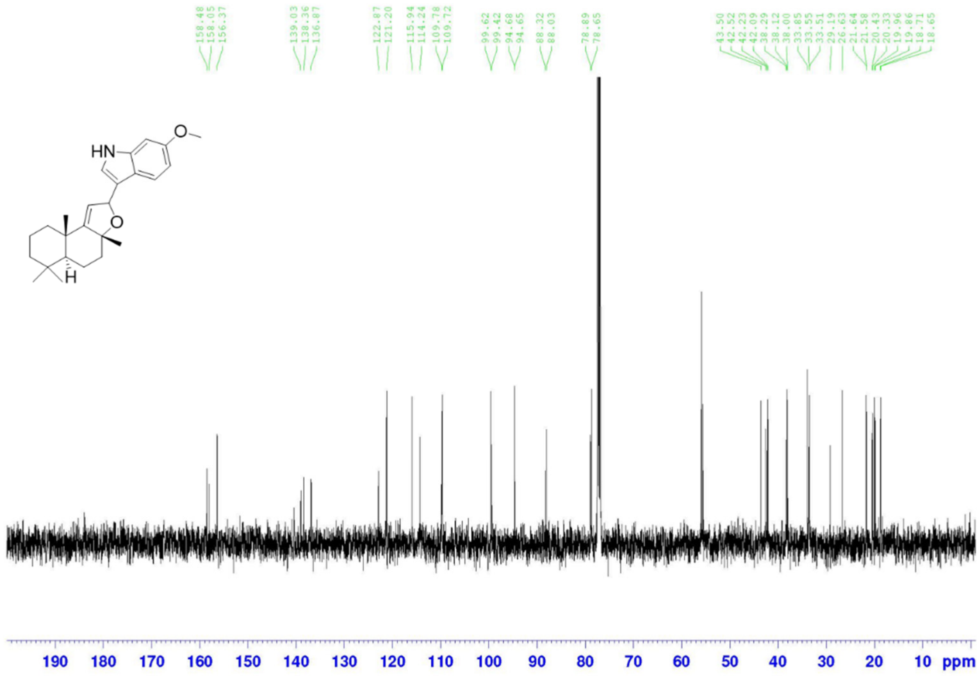

^{13}C NMR of Compound **8q**

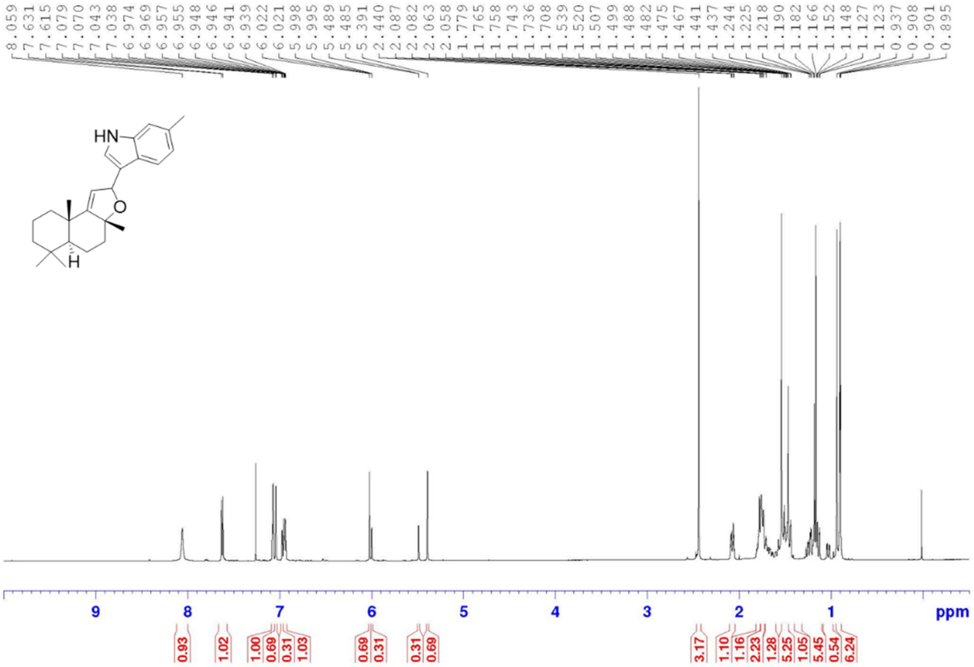

^{1}H NMR of Compound **8r**

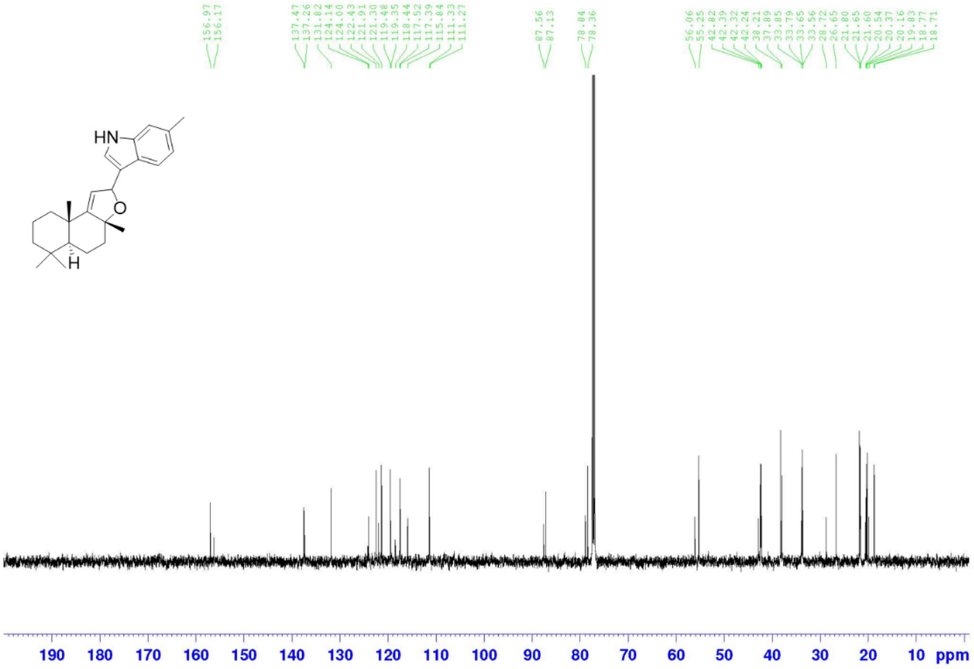

^{13}C NMR of Compound **8r**

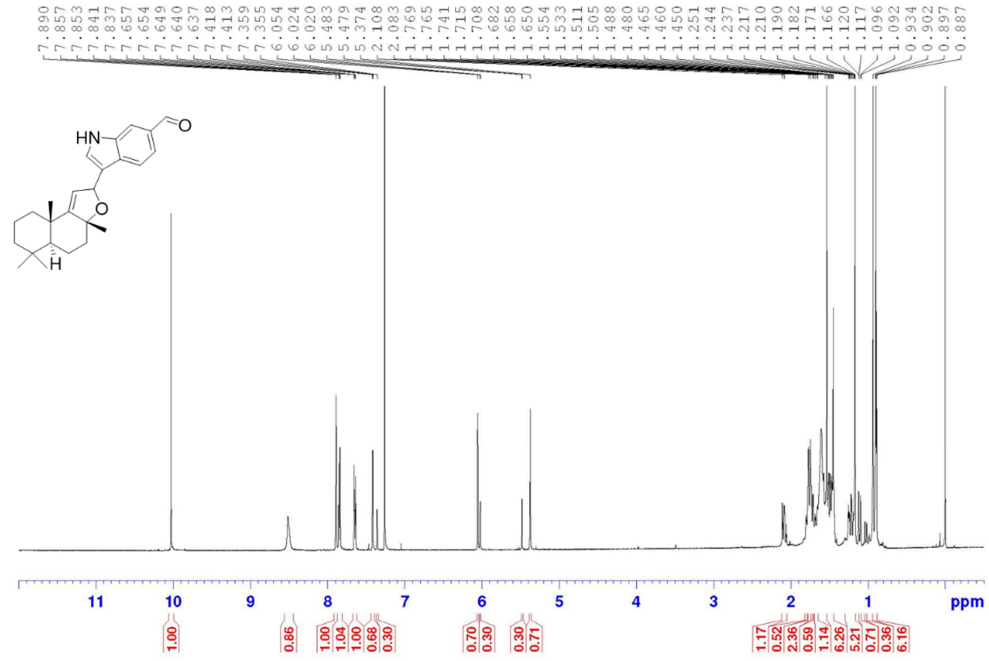

^{1}H NMR of Compound **8s**

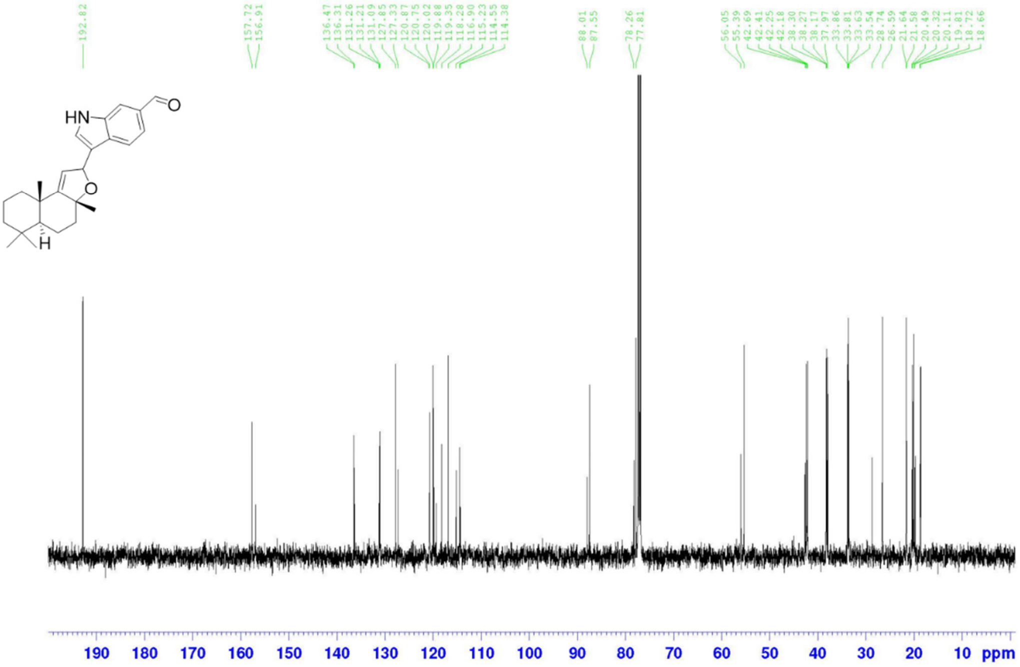

^{13}C NMR of Compound **8s**

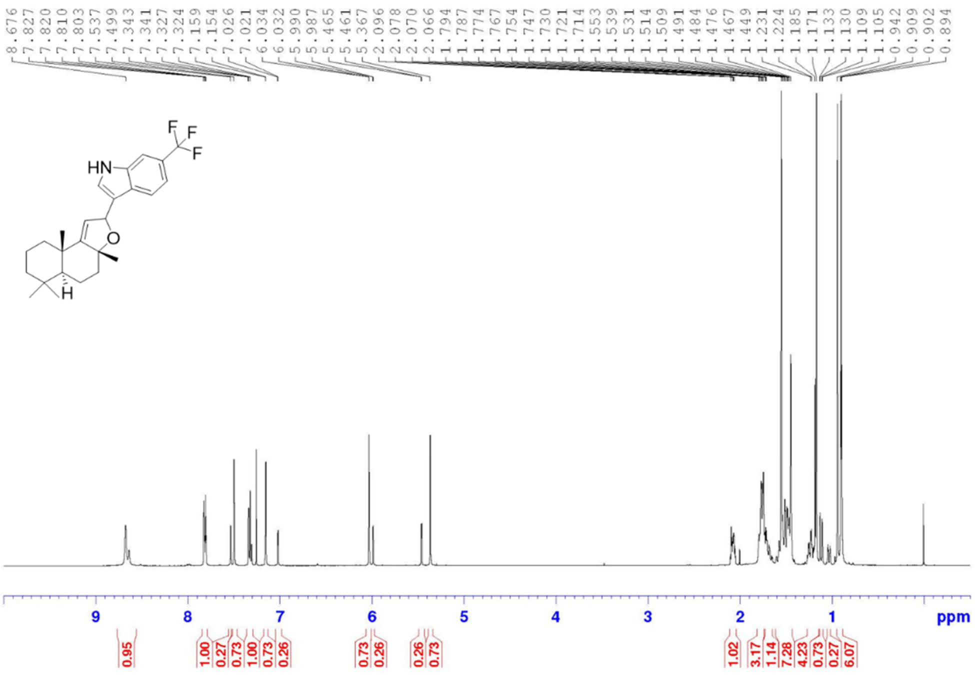

^{1}H NMR of Compound **8t**

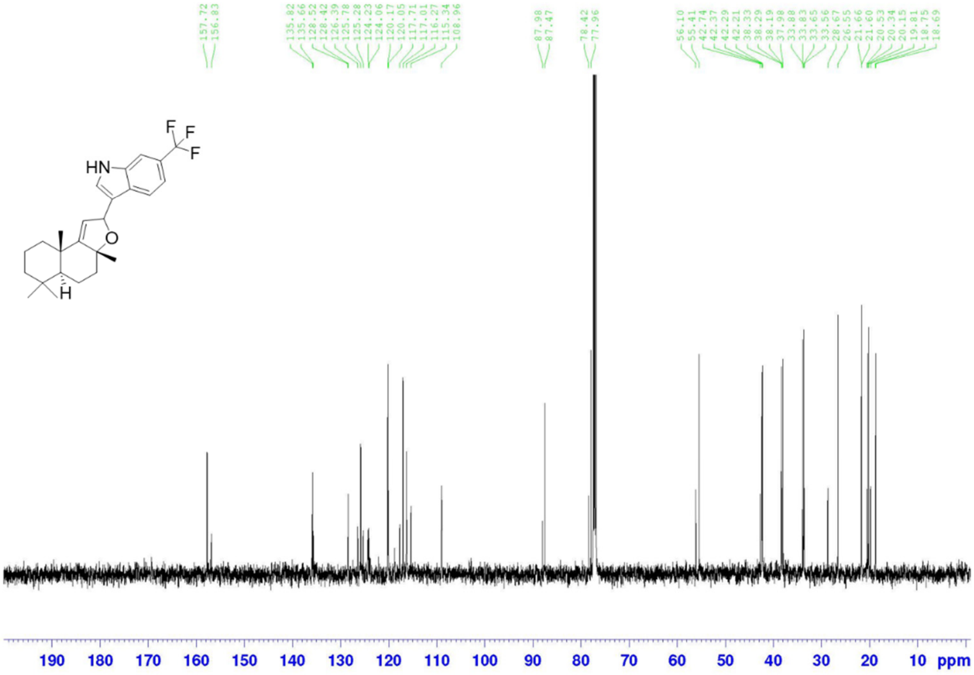

^{13}C NMR of Compound **8t**

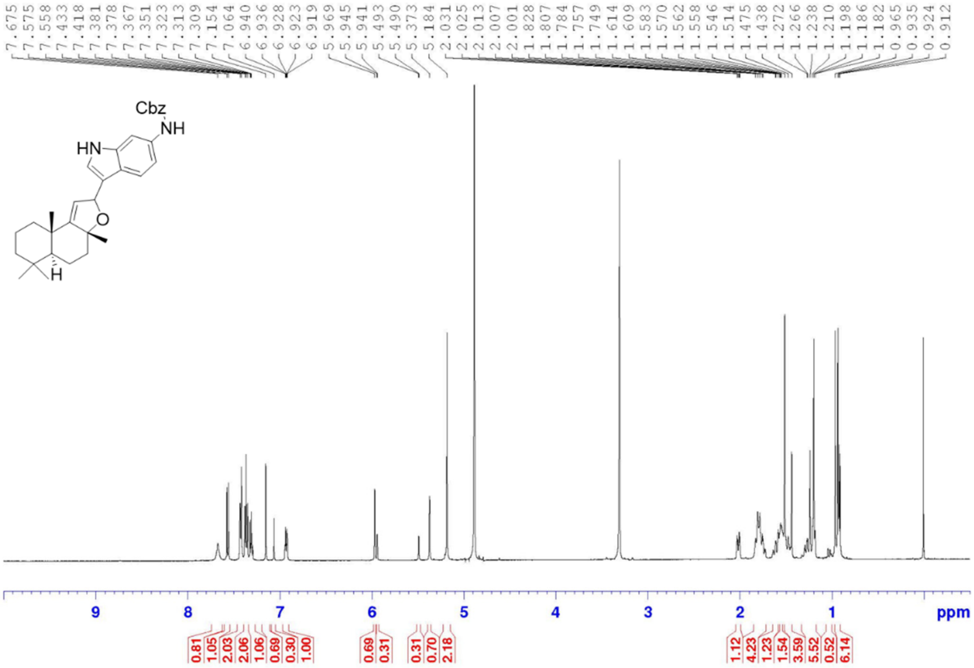

^{1}H NMR of Compound **8u**

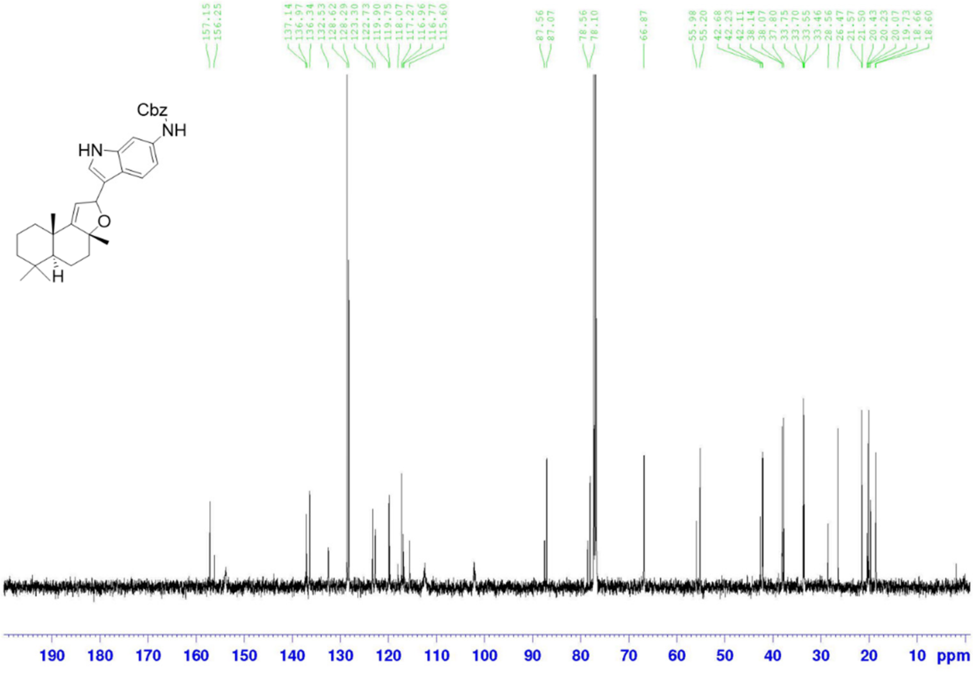

^{13}C NMR of Compound **8u**

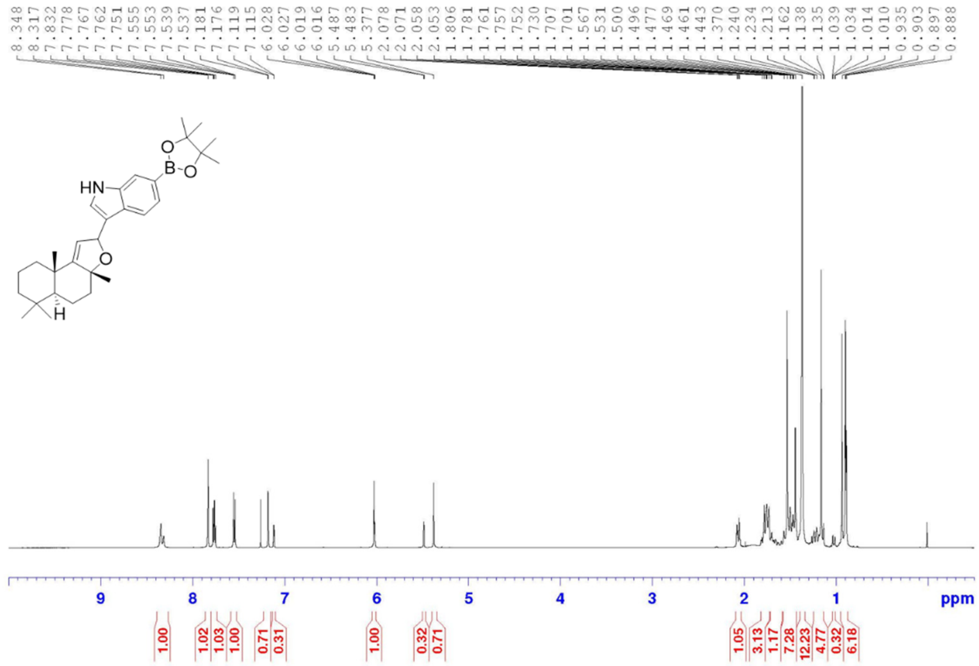

^{1}H NMR of Compound **8v**

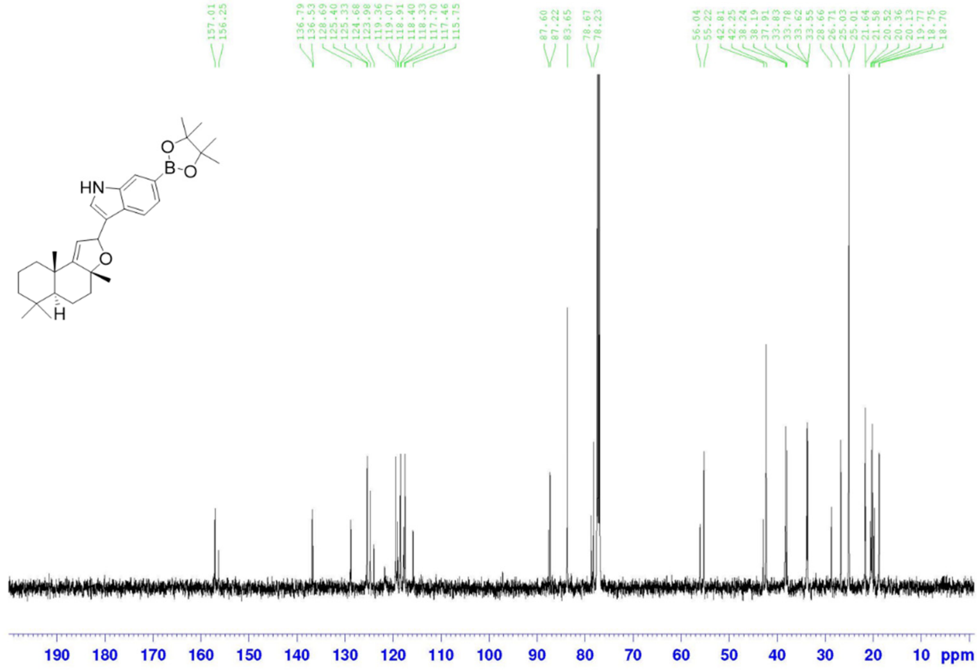

^{13}C NMR of Compound **8v**

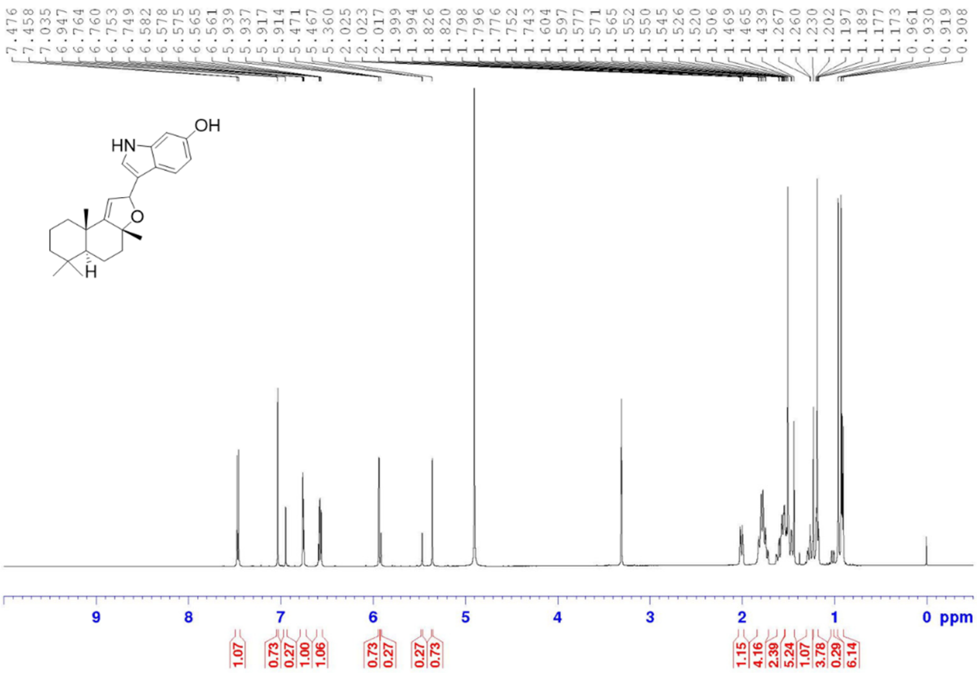

^{1}H NMR of Compound **8w**

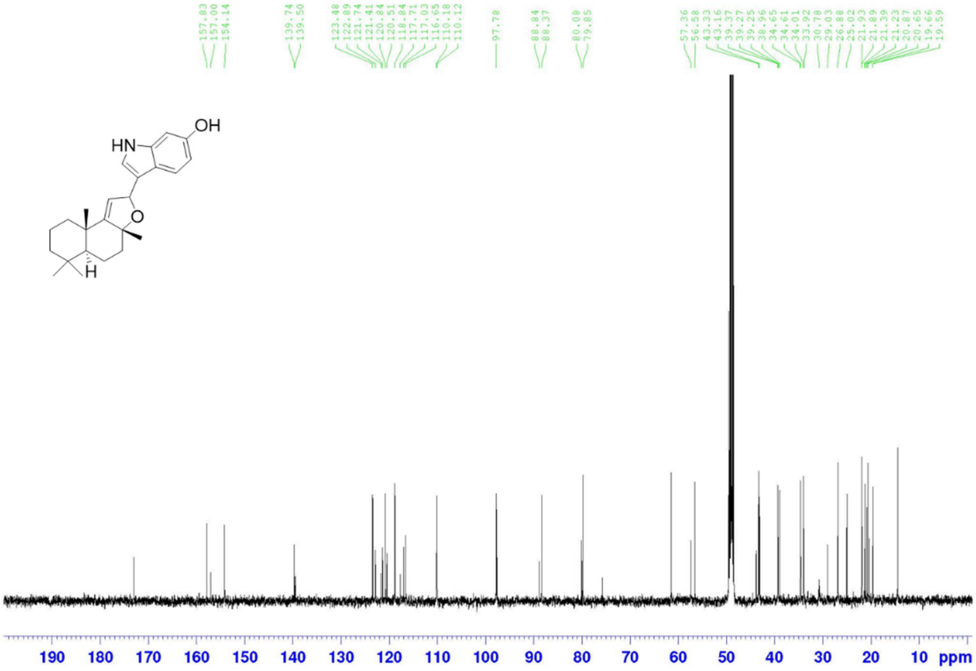

^{13}C NMR of Compound **8w**

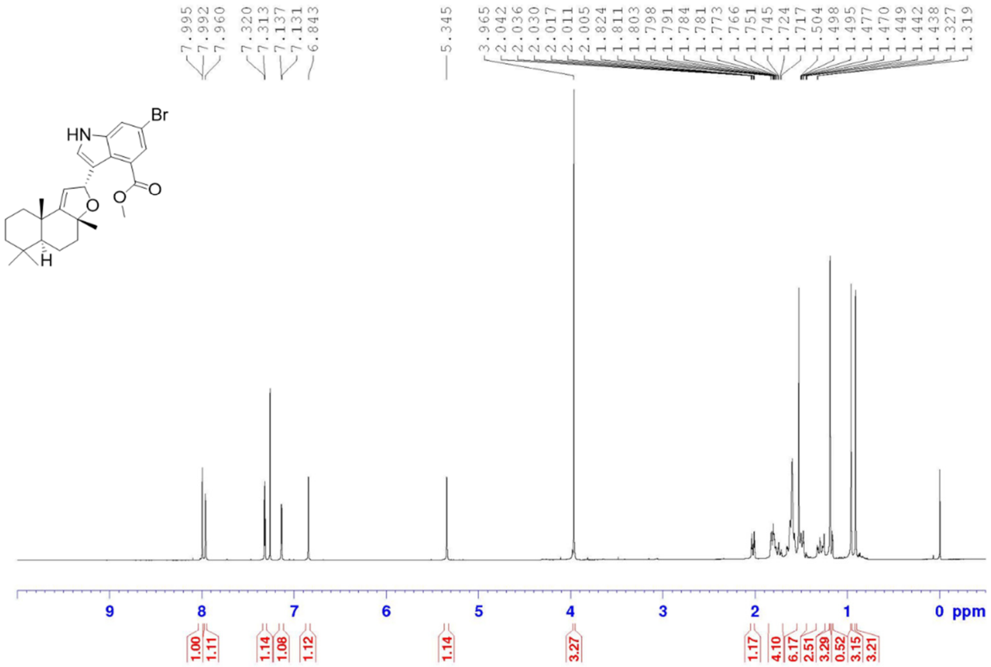

^{1}H NMR of Compound **8x**

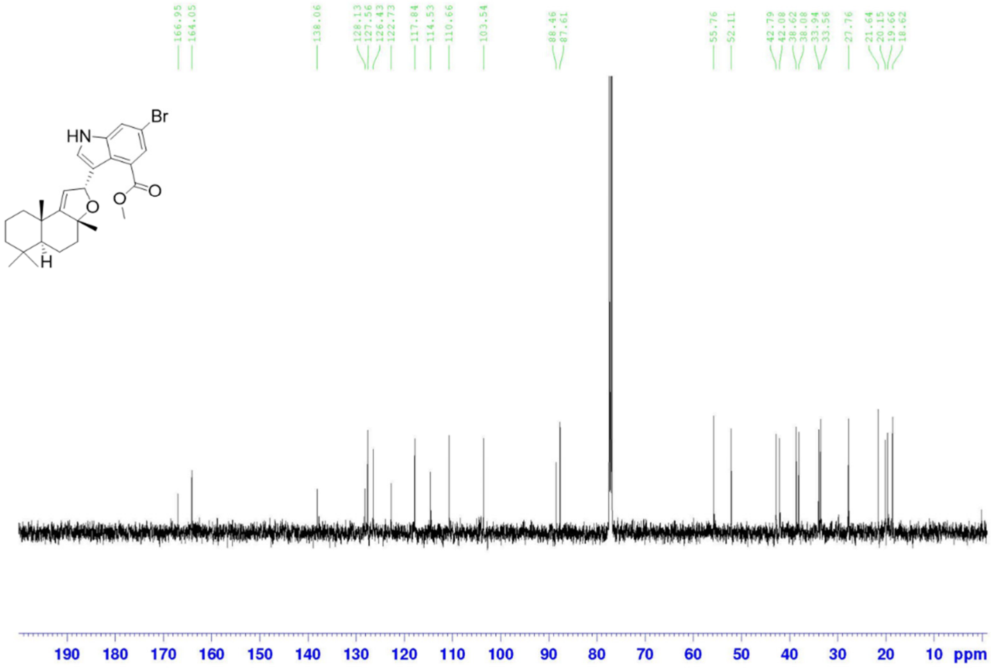

^{13}C NMR of Compound **8x**

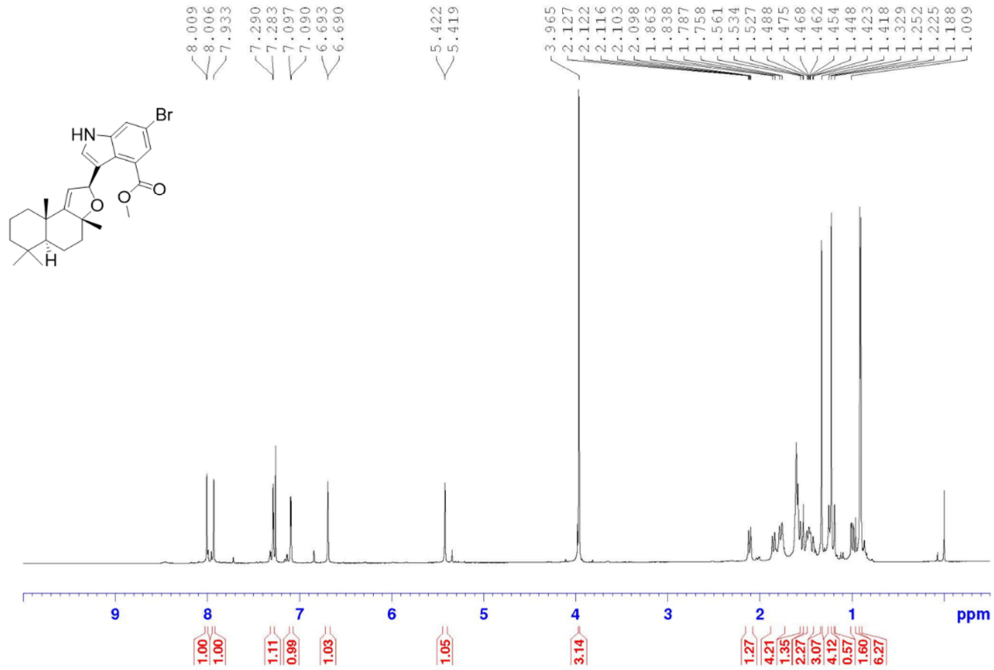

^{1}H NMR of Compound **8x'**

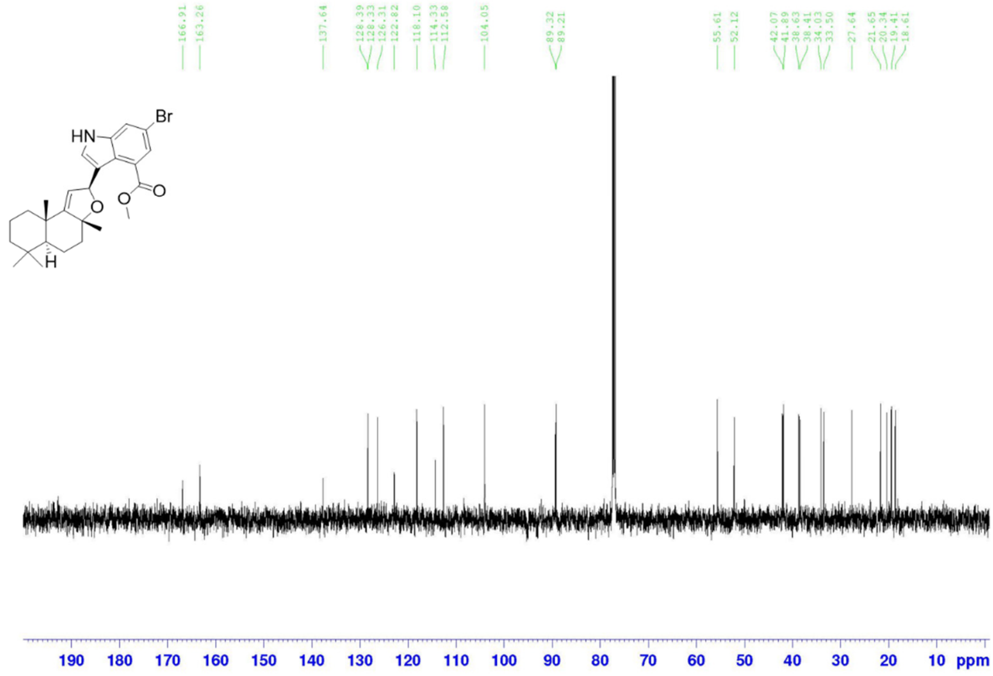

^{13}C NMR of Compound **8x'**

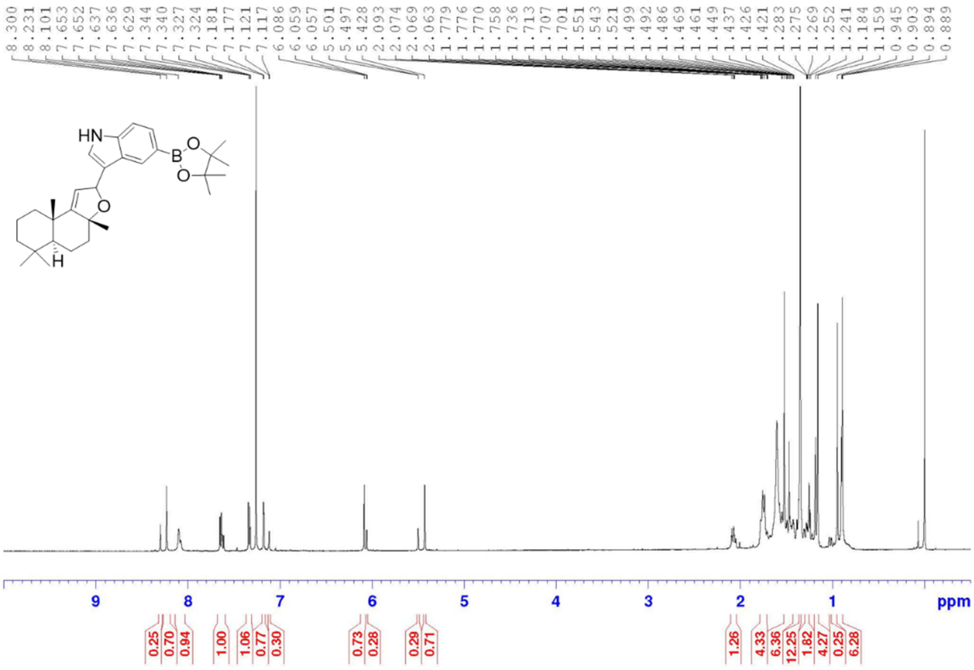

^{1}H NMR of Compound **8y**

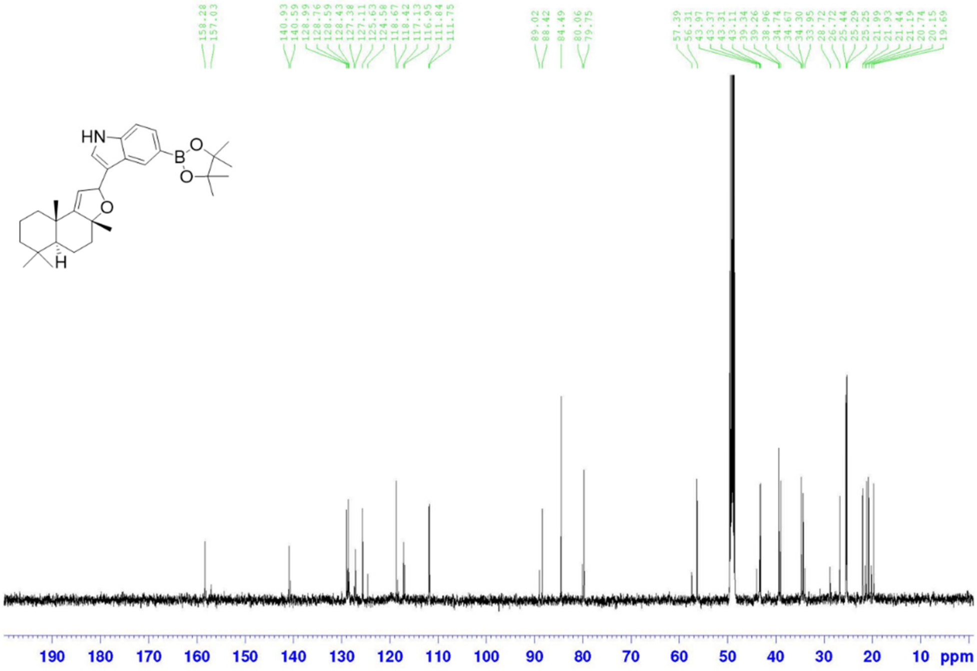

^{13}C NMR of Compound **8y**

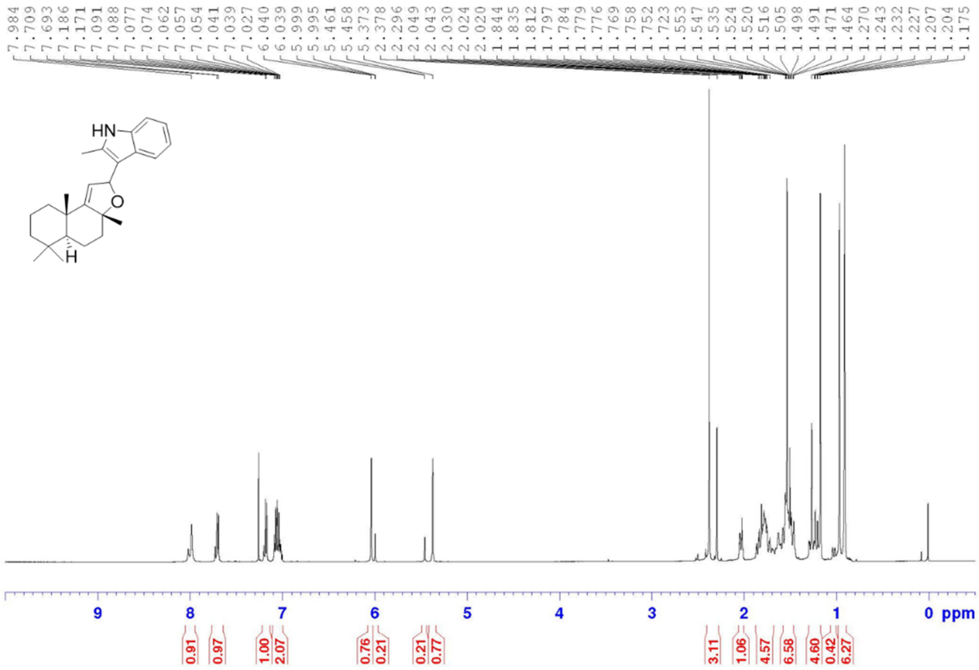

^{1}H NMR of Compound **8ab**

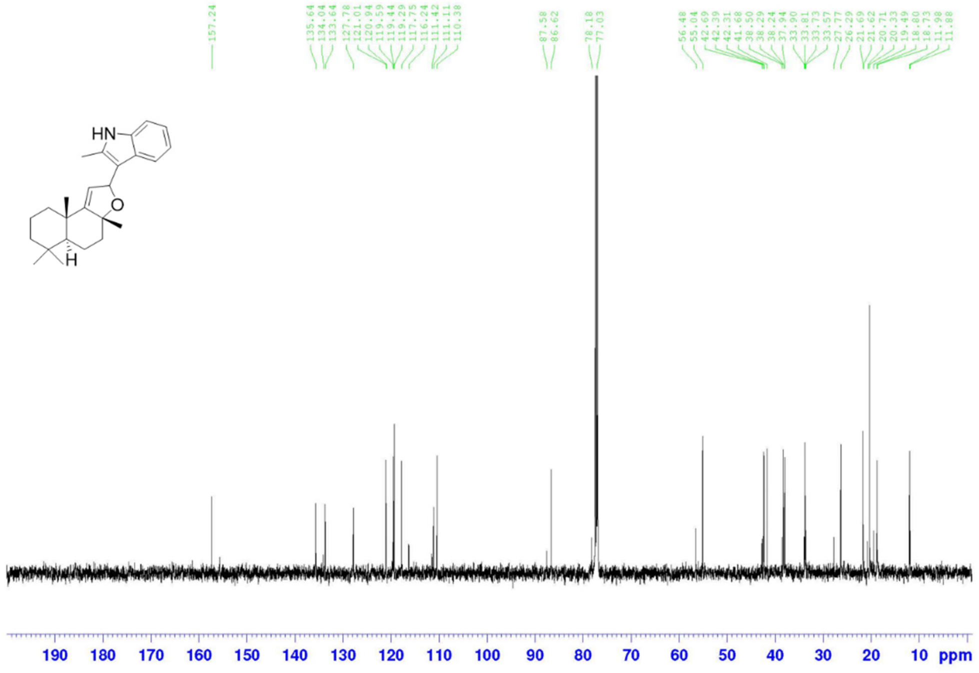

^{13}C NMR of Compound **8ab**

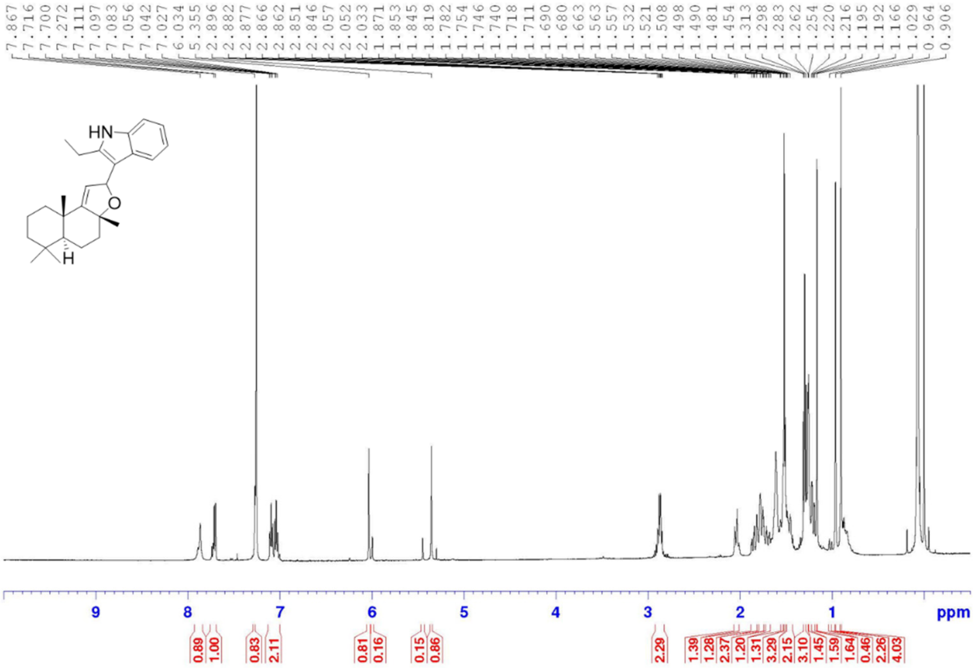

^{1}H NMR of Compound **8ac**

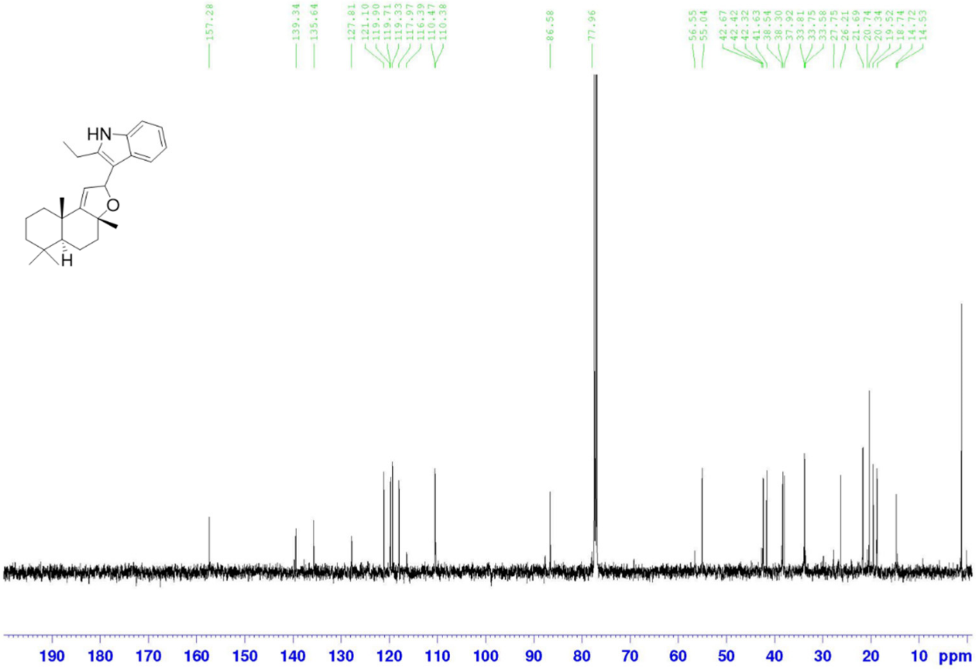

^{13}C NMR of Compound **8ac**

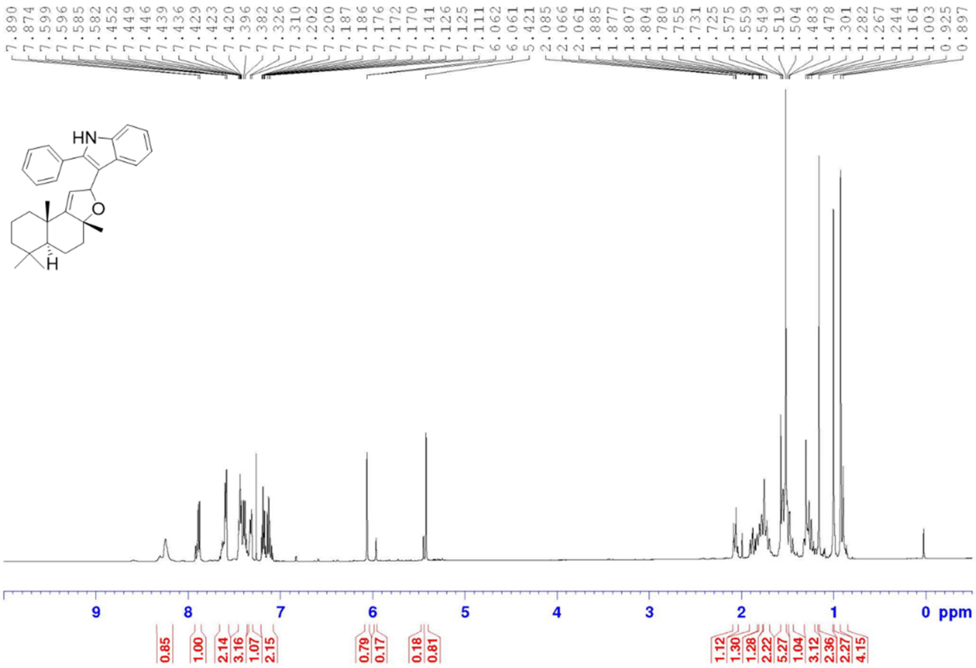

^{1}H NMR of Compound **8ad**

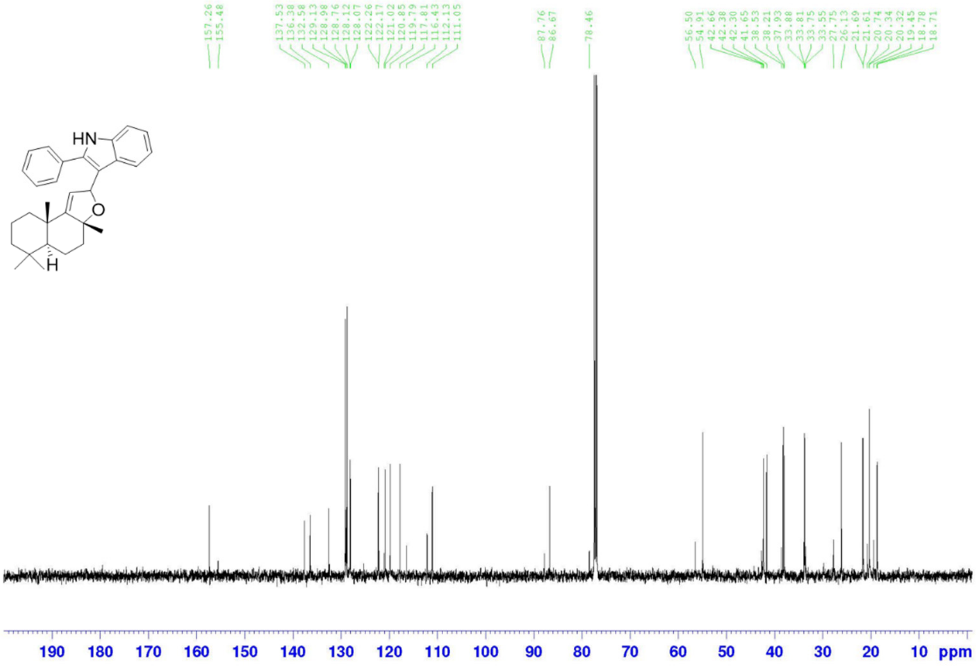

^{13}C NMR of Compound **8ad**

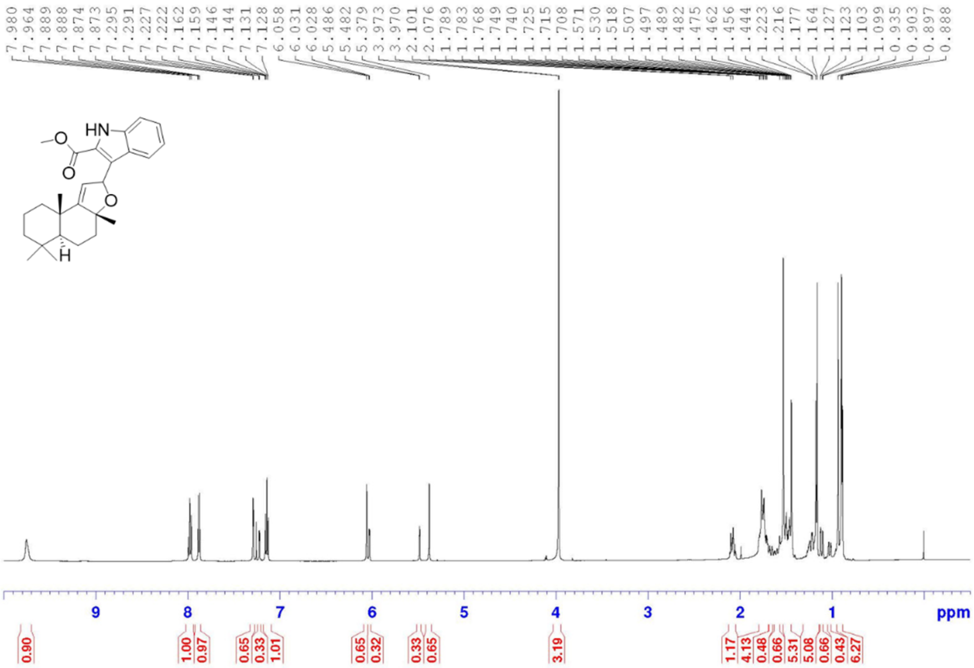

^{1}H NMR of Compound **8ae**

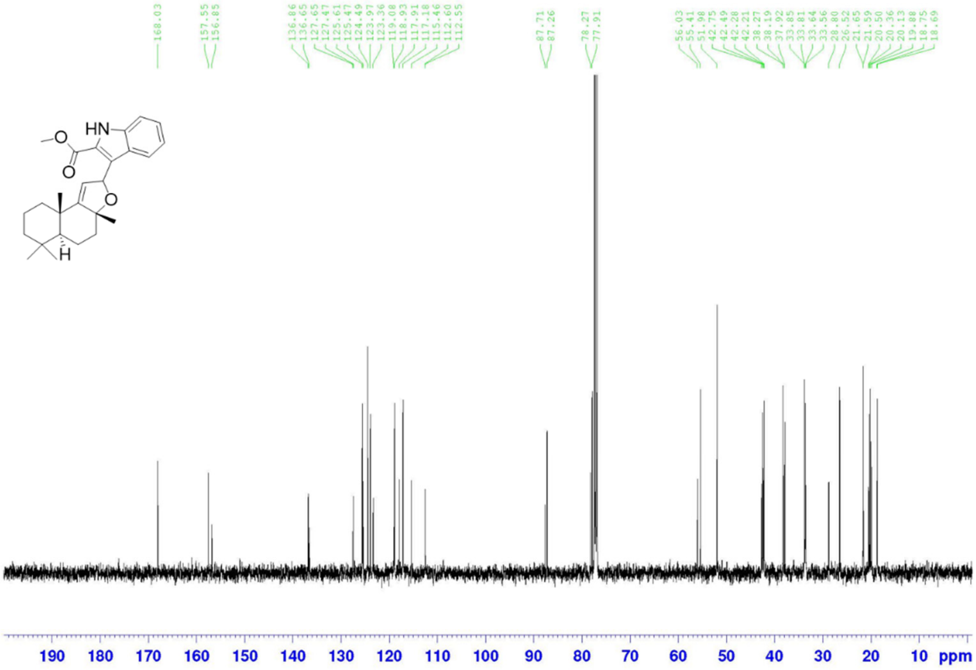

^{13}C NMR of Compound **8ae**

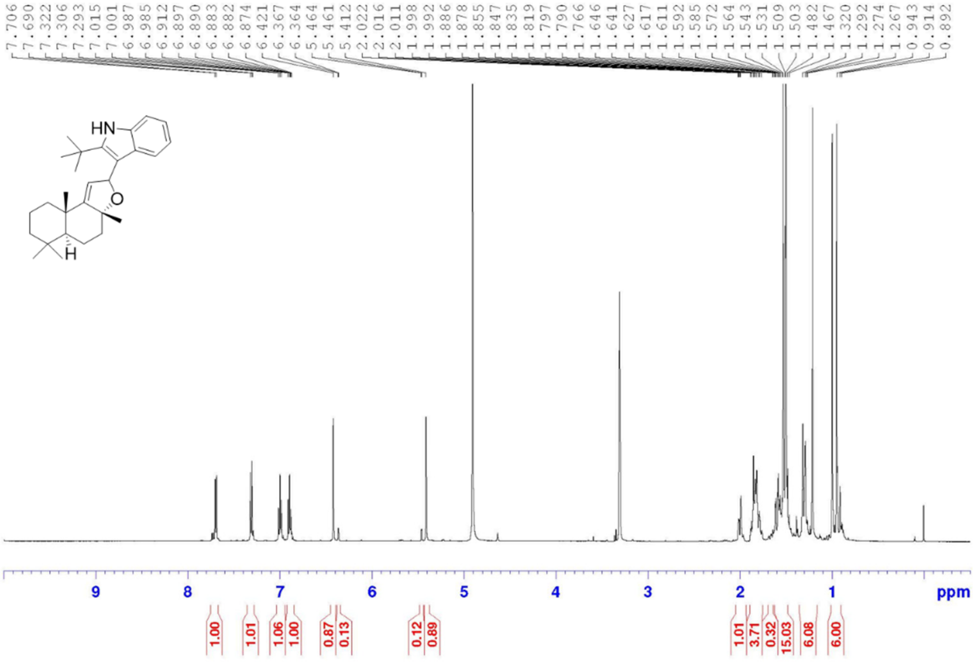

^{1}H NMR of Compound **8af**

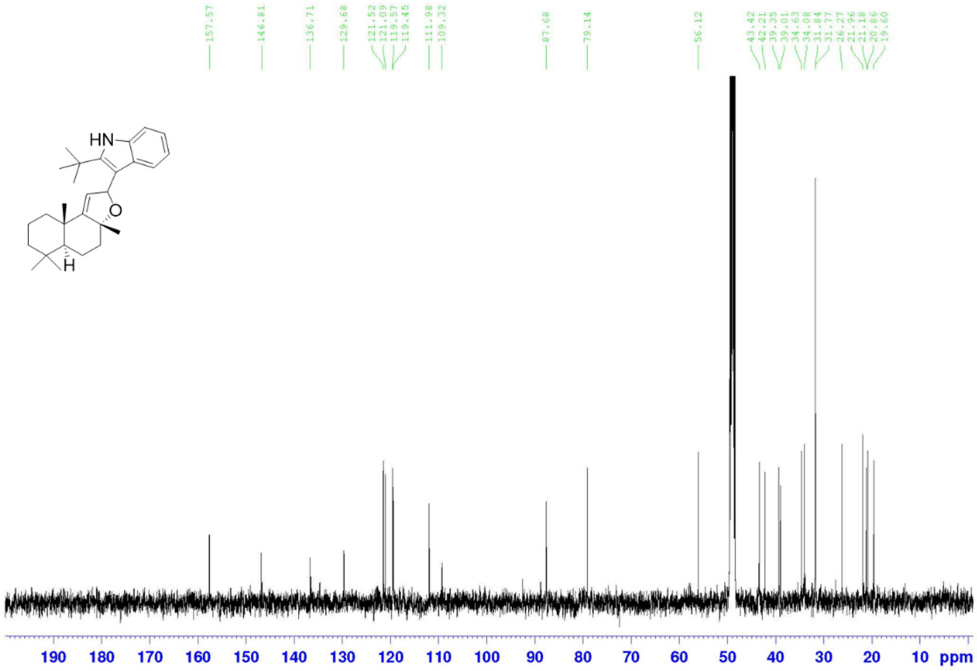

^{13}C NMR of Compound **8af**

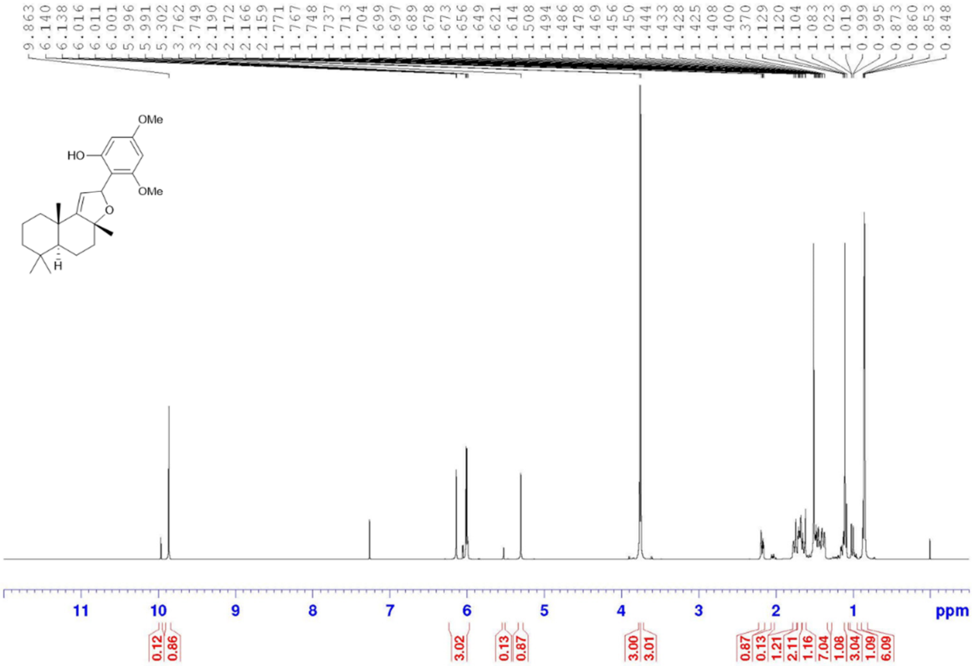

^{1}H NMR of Compound **9**

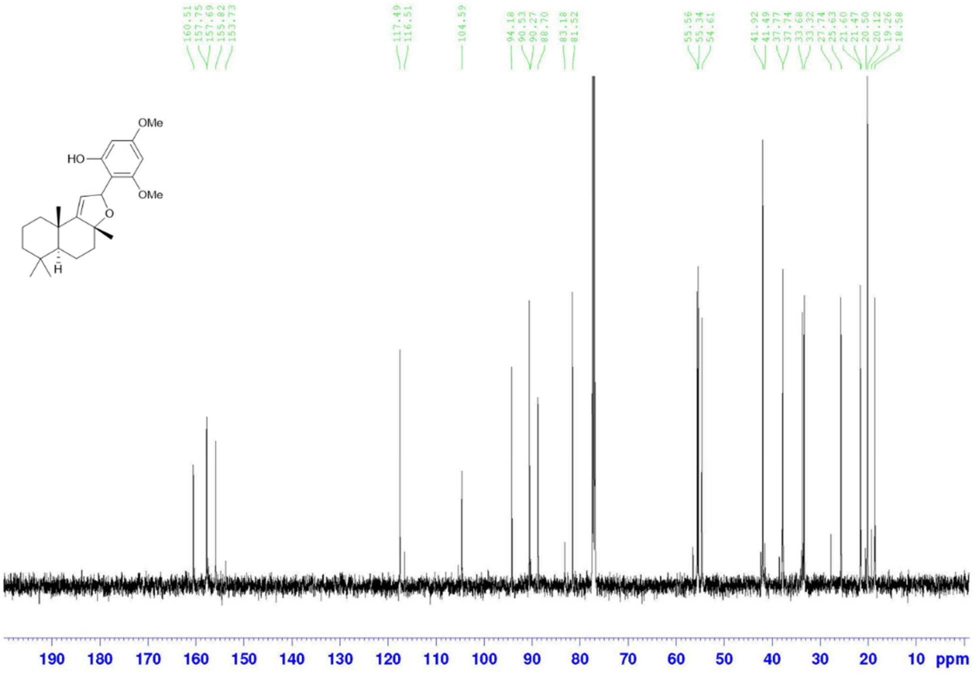

^{13}C NMR of Compound **9**

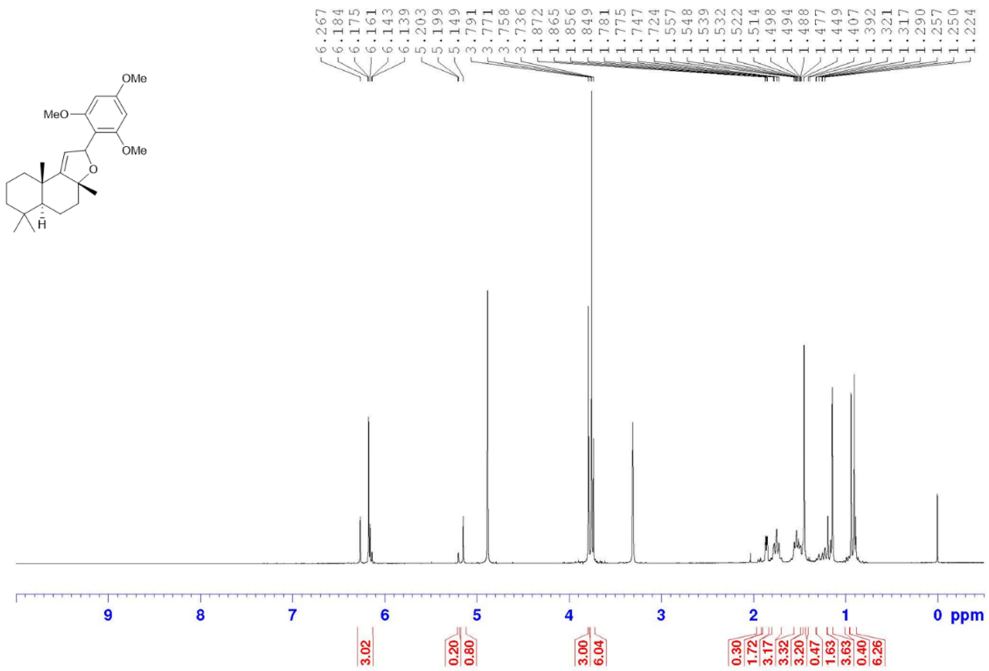

^{1}H NMR of Compound **10**

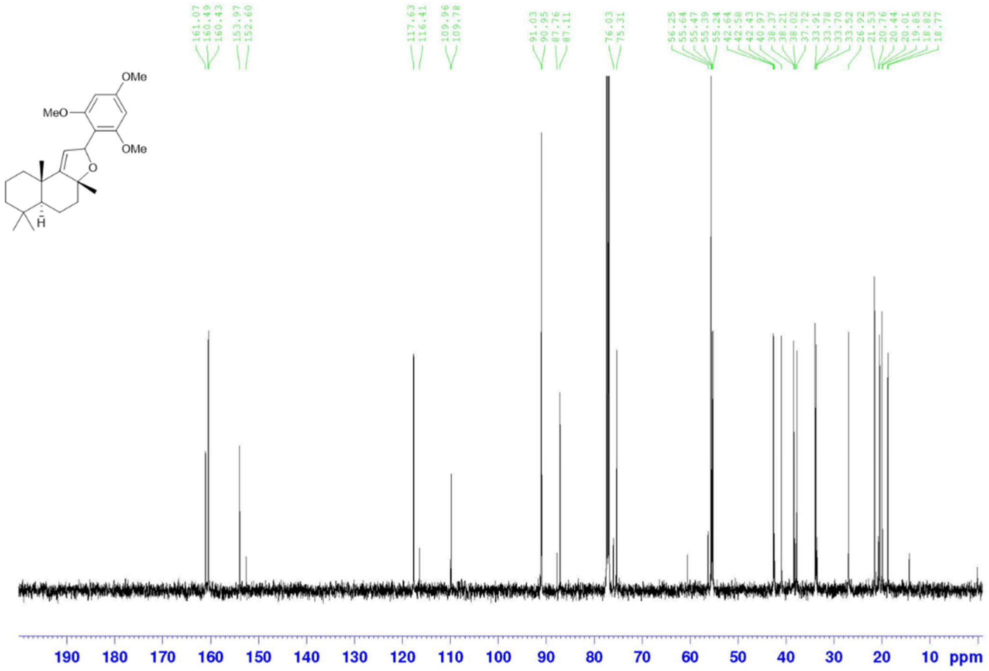

^{13}C NMR of Compound **10**

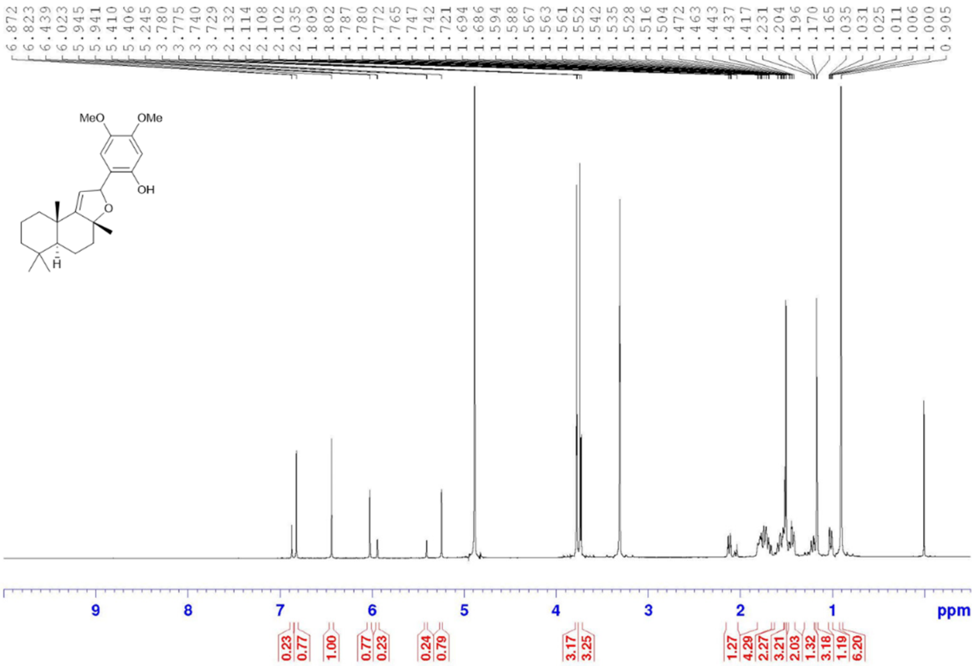

^{1}H NMR of Compound **11**

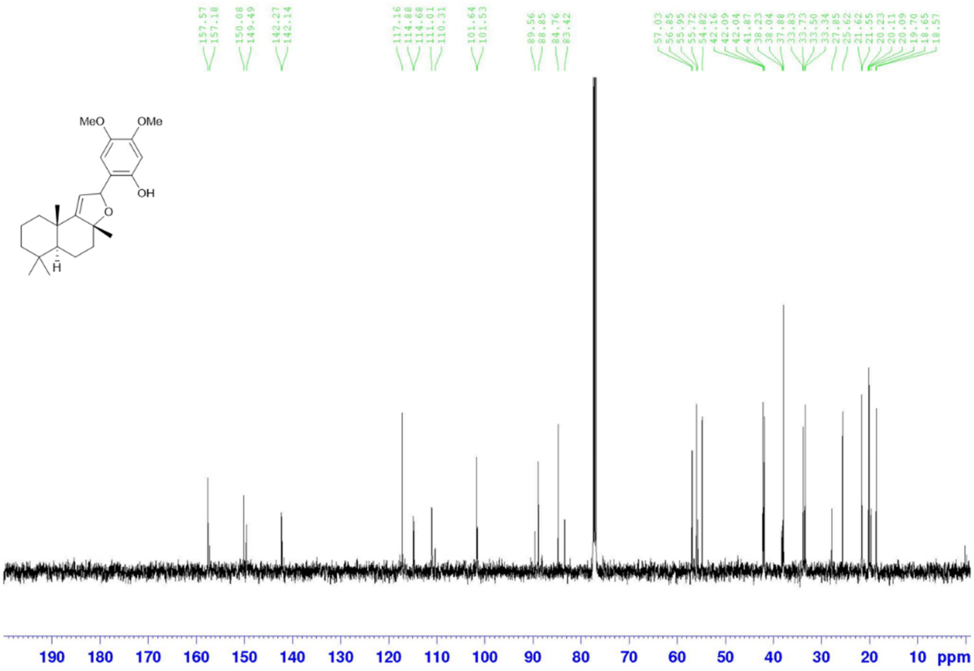

^{13}C NMR of Compound **11**

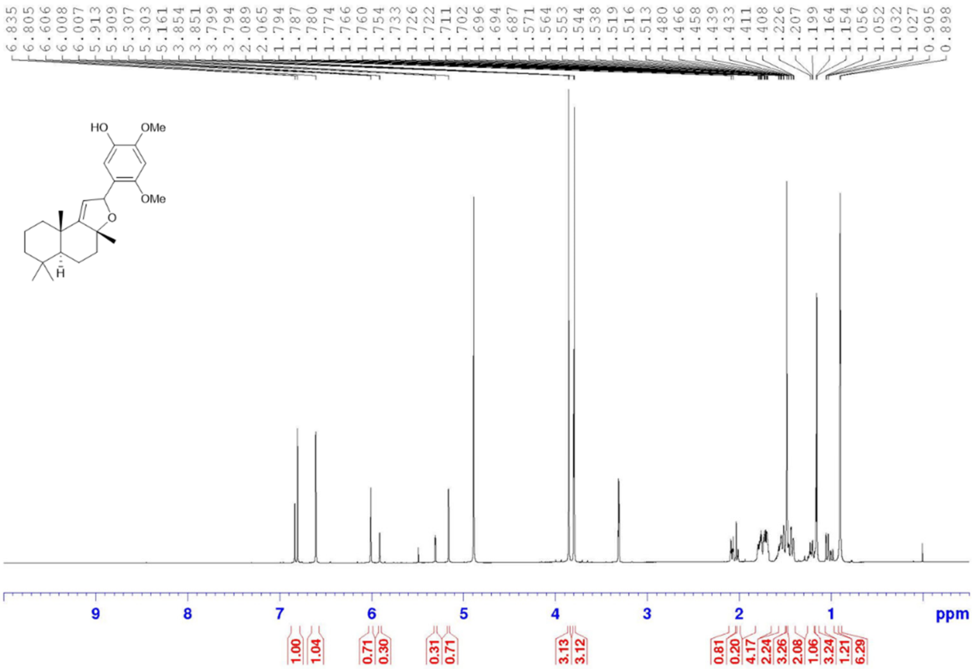

^{1}H NMR of Compound **12**

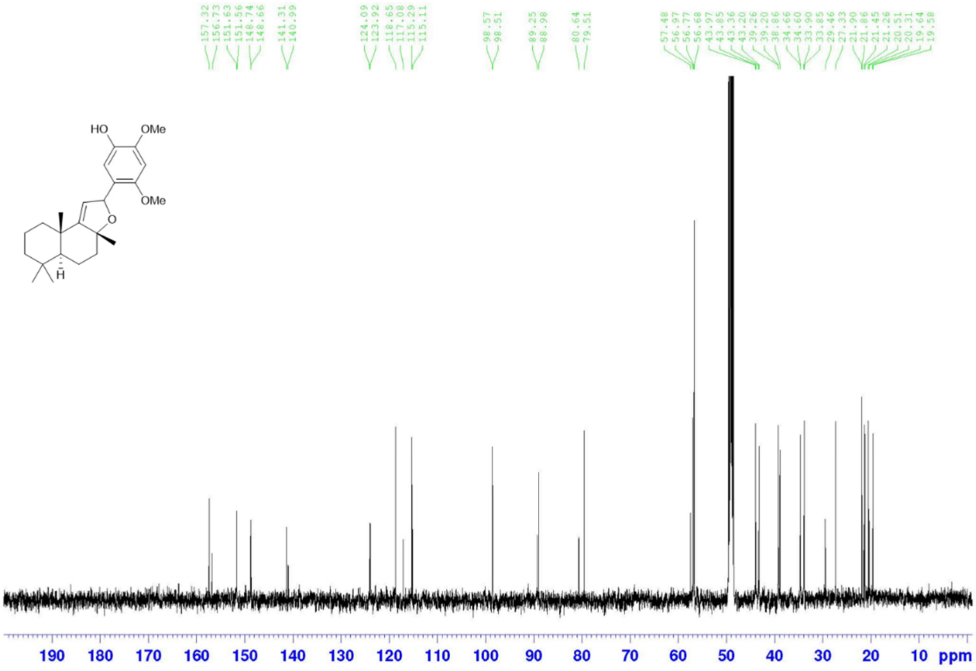

^{13}C NMR of Compound **12**

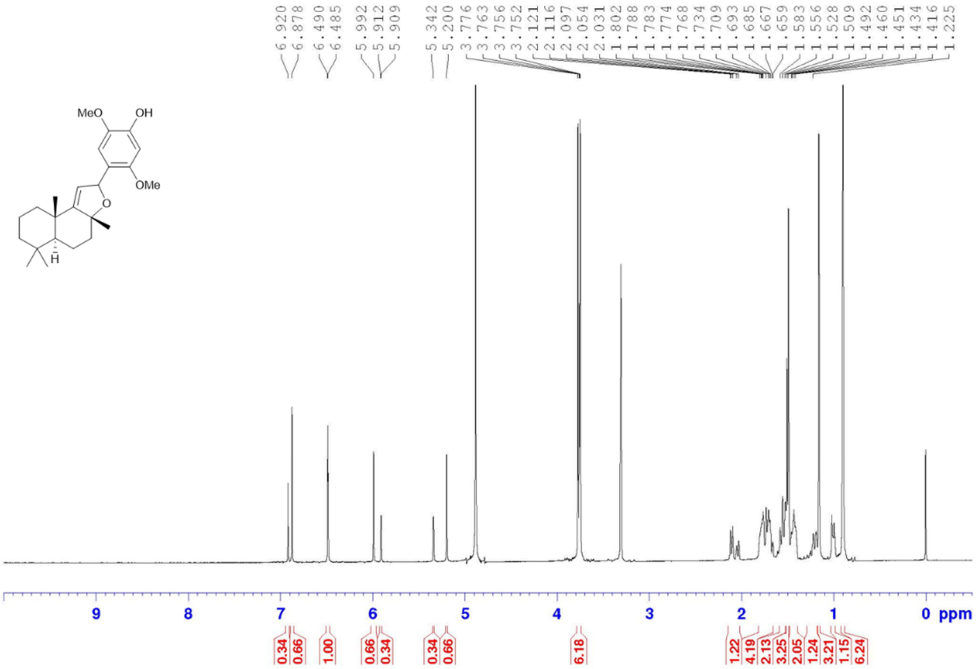

^{1}H NMR of Compound **13**

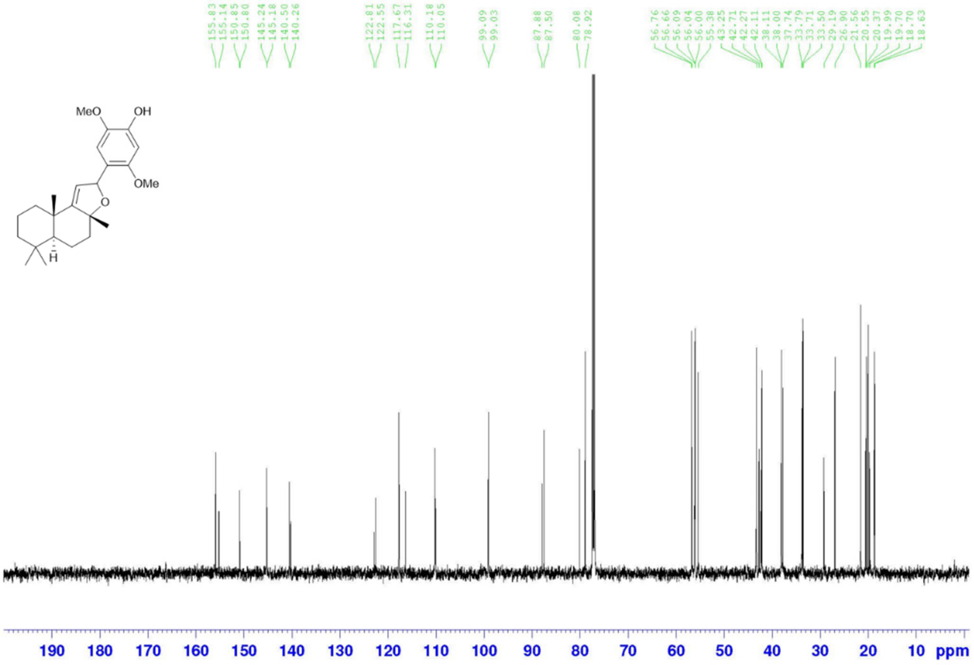

^{13}C NMR of Compound **13**

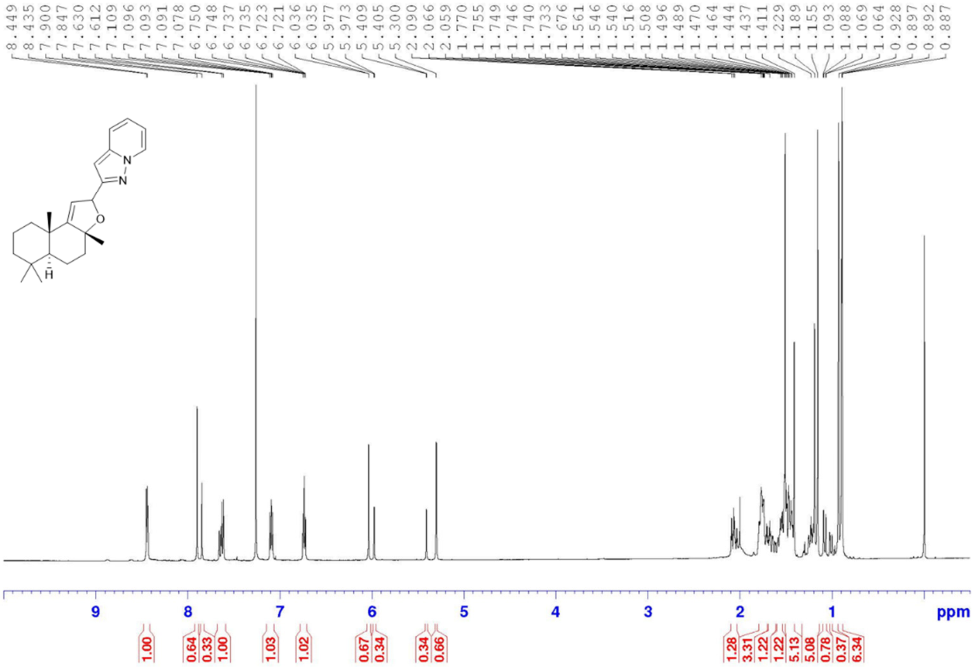

^{1}H NMR of Compound **14**

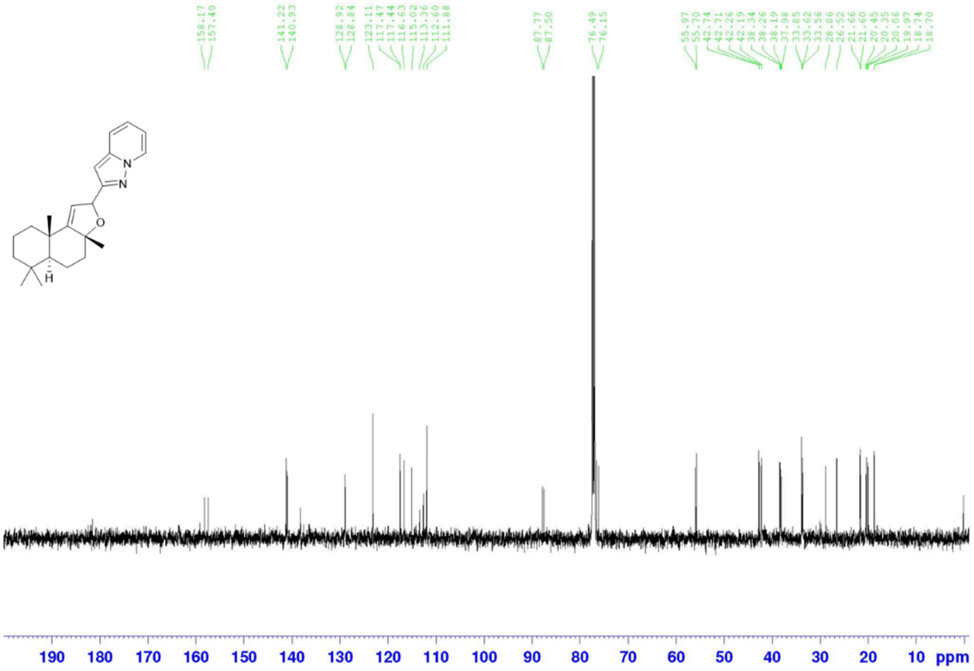

^{13}C NMR of Compound **14**

4. Original HRMS spectra of final compounds

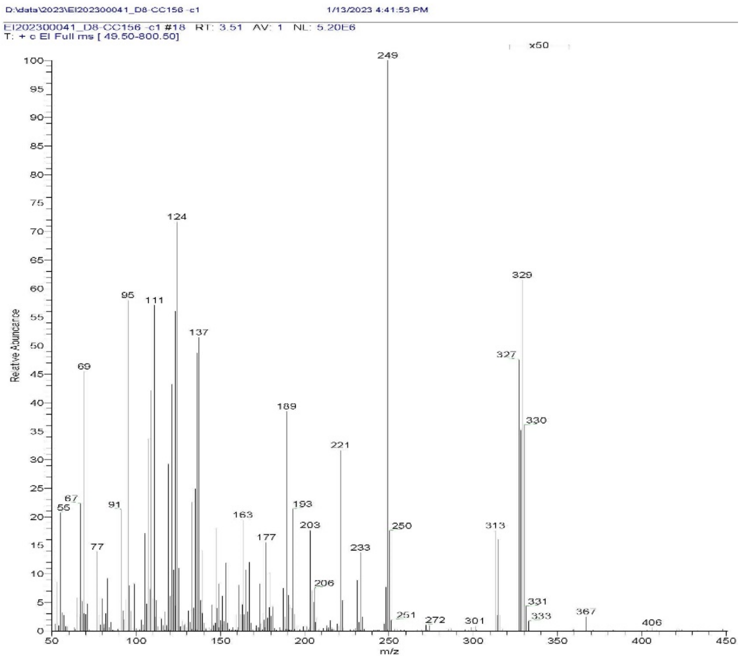

D:\data\2023\EI202300041_D8-CC156 -c1 1/13/2023 4:41:53 PM

EI202300041_D8-CC156 -c1#23 RT: 4.54
T: + c EI Full ms [49.50-800.50]
m/z= 48-803

m/z	Intensity	Relative	Theo. Mass	Delta (mmu)	RDB equiv.	Composition
192.1486	55273.0	0.85	192.1509	-2.28	4.0	$C_{13}H_{20}O_{1}$
193.1223	1446179.0	22.23	193.1223	-0.01	4.5	$C_{12}H_{17}O_{2}$
194.1259	189459.0	2.91	194.1301	-4.24	4.0	$C_{12}H_{18}O_{2}$
203.1803	1090244.0	16.76	203.1794	0.88	4.5	$C_{15}H_{23}$
203.9787	491208.0	7.55	203.9780	0.68	3.0	$C_{7}H_{9}O_{2}{}^{79}Br_{1}$
204.1843	193431.0	2.97	204.1873	-2.91	4.0	$C_{15}H_{24}$
205.1228	281918.0	4.33	205.1223	0.52	5.5	$C_{13}H_{17}O_{2}$
205.1597	283469.0	4.36	205.1587	1.01	4.5	$C_{14}H_{21}O_{1}$
205.9771	492106.0	7.56	205.9726	4.52	7.0	$C_{10}H_{7}{}^{79}Br_{1}$
206.1276	60551.0	0.93	206.1301	-2.53	5.0	$C_{13}H_{18}O_{2}$
206.1653	75185.0	1.16	206.1665	-1.25	4.0	$C_{14}H_{22}O_{1}$
207.1383	133614.0	2.05	207.1380	0.34	4.5	$C_{13}H_{19}O_{2}$
207.1754	63488.0	0.98	207.1743	1.08	3.5	$C_{14}H_{23}O_{1}$
213.1621	77116.0	1.19	213.1638	-1.65	6.5	$C_{16}H_{21}$
214.0334	62455.0	0.96	214.0352	-1.77	3.0	$C_{10}H_{15}{}^{79}Br_{1}$
215.1426	104862.0	1.61	215.1430	-0.44	6.5	$C_{15}H_{19}O_{1}$
219.1377	79537.0	1.22	219.1380	-0.27	5.5	$C_{14}H_{19}O_{2}$
220.1820	61693.0	0.95	220.1822	-0.20	4.0	$C_{15}H_{24}O_{1}$
221.1895	1955857.0	30.06	221.1900	-0.51	3.5	$C_{15}H_{25}O_{1}$
222.1932	317063.0	4.87	222.1978	-4.62	3.0	$C_{15}H_{26}O_{1}$
231.1749	545693.0	8.39	231.1743	0.58	5.5	$C_{16}H_{23}O_{1}$
233.1533	900512.0	13.84	233.1536	-0.26	5.5	$C_{15}H_{21}O_{2}$
234.1568	146834.0	2.26	234.1614	-4.64	5.0	$C_{15}H_{22}O_{2}$
247.1693	88296.0	1.36	247.1693	0.01	5.5	$C_{16}H_{23}O_{2}$
248.1769	483292.0	7.43	248.1771	-0.17	5.0	$C_{16}H_{24}O_{2}$
249.1840	6505997.0	100.00	249.1849	-0.90	4.5	$C_{16}H_{25}O_{2}$
272.0412	63379.0	0.97	272.0406	0.60	4.0	$C_{12}H_{17}O_{2}{}^{79}Br_{1}$
274.0395	64821.0	1.00	274.0352	4.30	8.0	$C_{15}H_{15}{}^{79}Br_{1}$
299.1005	76654.0	1.18	299.1005	0.02	3.5	$C_{15}H_{24}O_{1}{}^{79}Br_{1}$
313.0787	1109884.0	17.06	313.0798	-1.11	4.5	$C_{15}H_{22}O_{2}{}^{79}Br_{1}$
327.0976	76382.0	1.17	327.0954	2.18	4.5	$C_{16}H_{24}O_{2}{}^{79}Br_{1}$
328.1033	72356.0	1.11	328.1032	0.08	4.0	$C_{16}H_{25}O_{2}{}^{79}Br_{1}$

2

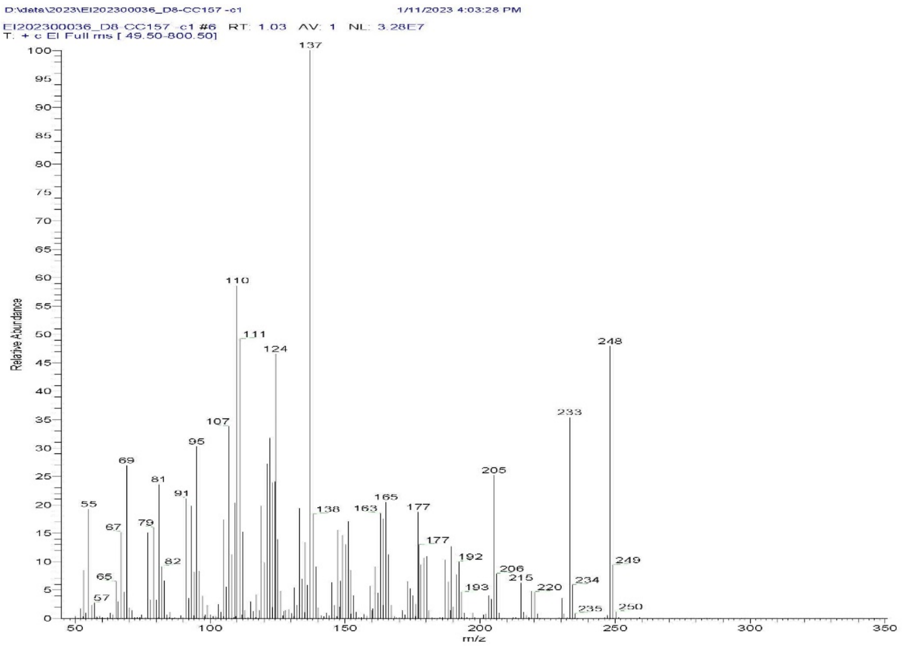

D:\data\2023\EI202300036_D8-CC157 -c1 1/11/2023 4:03:28 PM

EI202300036_D8-CC157 -c1#7 RT: 1.24
T: + c EI Full ms [49.50-800.50]
m/z= 48-803

m/z	Intensity	Relative	Theo. Mass	Delta (mmu)	RDB equiv.	Composition
69.0694	7841720.0	23.54	69.0699	-0.43	1.5	C_5H_9
79.0178	5724953.0	17.19	79.0178	-0.07	4.5	$C_5H_3O_1$
81.0309	7632278.0	22.91	81.0335	-2.62	3.5	$C_5H_5O_1$
93.0699	7022084.0	21.08	93.0699	-0.01	3.5	C_7H_9
95.0882	10181120.0	30.56	95.0855	2.69	2.5	C_7H_{11}
107.0114	11855872.0	35.59	107.0128	-1.35	5.5	$C_6H_3O_2$
119.0859	7105937.0	21.33	119.0855	0.41	4.5	C_9H_{11}
133.1014	7173873.0	21.54	133.1012	0.23	4.5	$C_{10}H_{13}$
135.0802	4855815.0	14.58	135.0804	-0.24	4.5	$C_9H_{11}O_1$
135.1163	4151128.0	12.46	135.1168	-0.50	3.5	$C_{10}H_{15}$
137.0597	3366226.0	10.10	137.0597	0.04	4.5	$C_8H_9O_2$
137.1323	33312512.0	100.00	137.1325	-0.18	2.5	$C_{10}H_{17}$
138.0675	6449632.0	19.36	138.0675	-0.06	4.0	$C_8H_{10}O_2$
147.1166	5863642.0	17.60	147.1168	-0.26	4.5	$C_{11}H_{15}$
149.0955	5249209.0	15.76	149.0961	-0.58	4.5	$C_{10}H_{13}O_1$
150.1033	4584152.0	13.76	150.1039	-0.61	4.0	$C_{10}H_{14}O_1$
151.0747	6188499.0	18.58	151.0754	-0.67	4.5	$C_9H_{11}O_2$
161.1316	3497804.0	10.50	161.1325	-0.85	4.5	$C_{12}H_{17}$
163.0744	6643075.0	19.94	163.0754	-0.92	5.5	$C_{10}H_{11}O_2$
164.0822	6372780.0	19.13	164.0832	-0.96	5.0	$C_{10}H_{12}O_2$
165.0904	7375849.0	22.14	165.0910	-0.65	4.5	$C_{10}H_{13}O_2$
166.0982	4039433.0	12.13	166.0988	-0.66	4.0	$C_{10}H_{14}O_2$
177.0912	6759255.0	20.29	177.0910	0.21	5.5	$C_{11}H_{13}O_2$
177.1275	3918111.0	11.76	177.1274	0.07	4.5	$C_{12}H_{17}O_1$
177.1637	4858987.0	14.59	177.1638	-0.11	3.5	$C_{13}H_{21}$
178.0975	3575778.0	10.73	178.0988	-1.31	5.0	$C_{11}H_{14}O_2$
179.1059	3889613.0	11.68	179.1067	-0.77	4.5	$C_{11}H_{15}O_2$
180.1134	4005274.0	12.02	180.1145	-1.05	4.0	$C_{11}H_{16}O_2$
187.1476	3977842.0	11.94	187.1481	-0.53	5.5	$C_{14}H_{19}$
189.1631	5132837.0	15.41	189.1638	-0.72	4.5	$C_{14}H_{21}$
192.1135	3937775.0	11.82	192.1145	-1.02	5.0	$C_{12}H_{16}O_2$
205.1220	5151380.0	15.46	205.1223	-0.27	5.5	$C_{13}H_{17}O_2$
205.1584	9623552.0	28.89	205.1587	-0.29	4.5	$C_{14}H_{21}O_1$
233.1531	13518080.0	40.58	233.1536	-0.49	5.5	$C_{15}H_{21}O_2$
248.1758	21569280.0	64.75	248.1771	-1.25	5.0	$C_{16}H_{24}O_2$

3

Qualitative Analysis Report

**Data Filename**	ESIH202205829.d	**Sample Name**	D8-CD099
**Sample ID**		**Position**	P1-D6
**Instrument Name**	Agilent G6520 Q-TOF	**Acq Method**	20160322_MS_ESIH_POS_1min.m
**Acquired Time**	12/21/2022 16:59:17	**IRM Calibration Status**	Success
**DA Method**	small molecular data analysis method.m	**Comment**	ESIH by zhuzhenyun

User Spectra

**Fragmentor Voltage**	**Collision Energy**	**Ionization Mode**
170	0	ESI

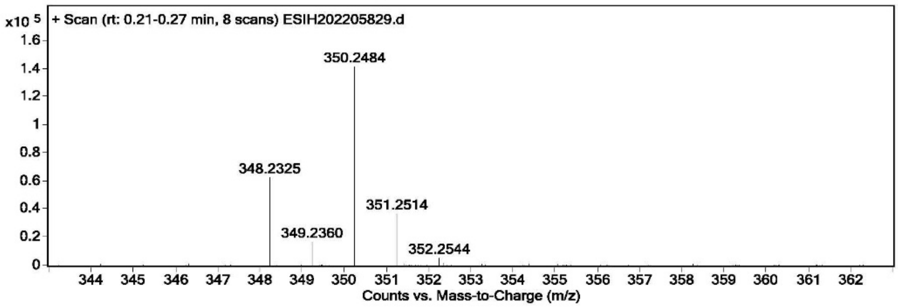

Formula Calculator Results

m/z	Calc m/z	Diff (mDa)	Diff (ppm)	Ion Formula	Ion
350.2484	350.2478	-0.59	-1.7	C24 H32 N O	(M+H)+

--- End Of Report ---

Agilent Technologies | Page 1 of 1 | Printed at: 17:19 on: 12/21/2022

6

Qualitative Analysis Report

**Data Filename**	ESIH202300067.d	**Sample Name**	D8-CD127
**Sample ID**		**Position**	P1-A5
**Instrument Name**	Agilent G6520 Q-TOF	**Acq Method**	20160322_MS_ESIH_POS_1min.m
**Acquired Time**	1/4/2023 10:41:59	**IRM Calibration Status**	Success
**DA Method**	small molecular data analysis method.m	**Comment**	ESIH by fangsu

User Spectra

**Fragmentor Voltage**	**Collision Energy**	**Ionization Mode**
170	0	ESI

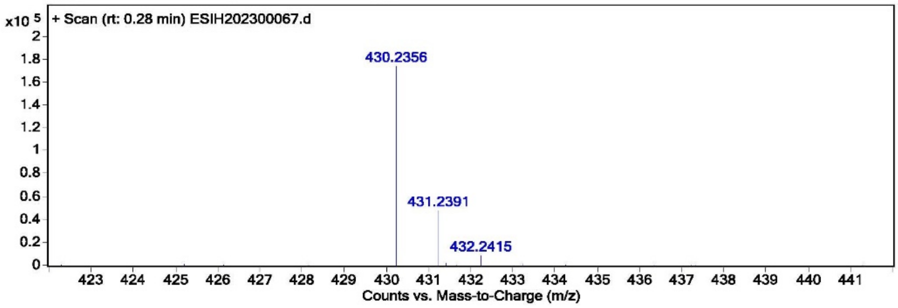

Formula Calculator Results

m/z	Calc m/z	Diff (mDa)	Diff (ppm)	Ion Formula	Ion
430.2356	430.2353	-0.34	-0.79	C26 H33 N Na O3	(M+Na)+

--- End Of Report ---

Agilent Technologies | Page 1 of 1 | Printed at: 10:43 on: 1/4/2023

8a

Qualitative Analysis Report

**Data Filename**	ESIH202300063.d	**Sample Name**	D8-CD129
**Sample ID**		**Position**	P1-A1
**Instrument Name**	Agilent G6520 Q-TOF	**Acq Method**	20160322_MS_ESIH_POS_1min.m
**Acquired Time**	1/4/2023 10:36:53	**IRM Calibration Status**	Success
**DA Method**	small molecular data analysis method.m	**Comment**	ESIH by fangsu

User Spectra

Fragmentor Voltage	Collision Energy	Ionization Mode
170	0	ESI

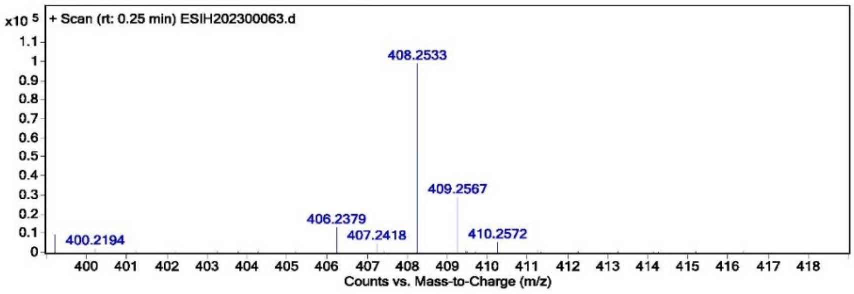

Formula Calculator Results

m/z	Calc m/z	Diff (mDa)	Diff (ppm)	Ion Formula	Ion
408.2533	408.2533	0.01	0.02	C26 H34 N O3	(M+H)+

--- End Of Report ---

8b

Qualitative Analysis Report

**Data Filename**	ESIH202300006.d	**Sample Name**	D8-CD130
**Sample ID**		**Position**	P1-A6
**Instrument Name**	Agilent G6520 Q-TOF	**Acq Method**	20160322_MS_ESIH_POS_1min.m
**Acquired Time**	1/3/2023 13:23:50	**IRM Calibration Status**	Success
**DA Method**	small molecular data analysis method.m	**Comment**	ESIH by fangsu

User Spectra

Fragmentor Voltage	Collision Energy	Ionization Mode
170	0	ESI

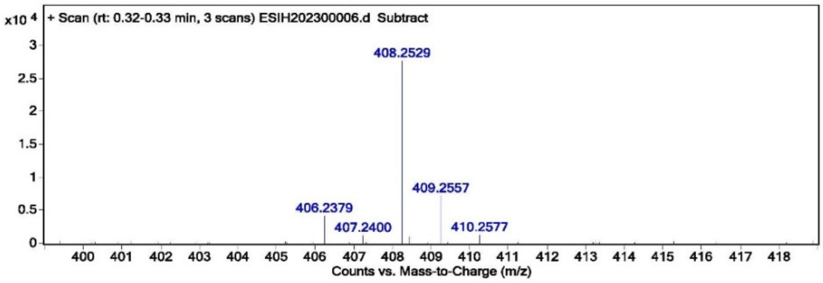

Formula Calculator Results

m/z	Calc m/z	Diff (mDa)	Diff (ppm)	Ion Formula	Ion
408.2529	408.2533	0.42	1.02	C26 H34 N O3	(M+H)+

--- End Of Report ---

8c

Qualitative Analysis Report

**Data Filename**	ESIH202300068.d	**Sample Name**	D8-CD146
**Sample ID**		**Position**	P1-A6
**Instrument Name**	Agilent G6520 Q-TOF	**Acq Method**	20160322_MS_ESIH_POS_1min.m
**Acquired Time**	1/4/2023 10:43:16	**IRM Calibration Status**	Success
**DA Method**	small molecular data analysis method.m	**Comment**	ESIH by fangsu

User Spectra

Fragmentor Voltage	Collision Energy	Ionization Mode
170	0	ESI

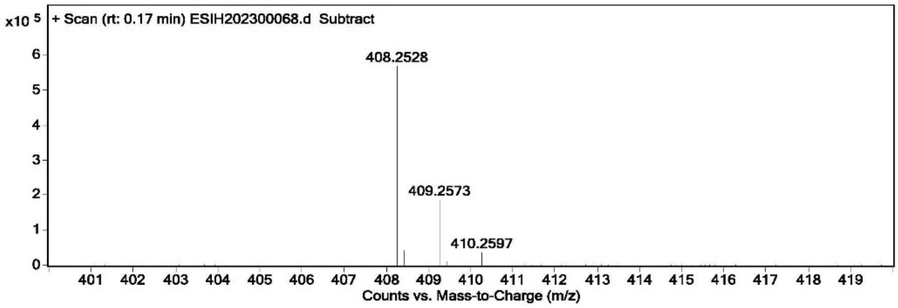

Formula Calculator Results

m/z	Calc m/z	Diff (mDa)	Diff (ppm)	Ion Formula	Ion
408.2528	408.2533	0.54	1.32	C26 H34 N O3	(M+H)+

--- End Of Report ---

8d

Qualitative Analysis Report

**Data Filename**	ESIH202300071.d	**Sample Name**	D8-CD153
**Sample ID**		**Position**	P1-A9
**Instrument Name**	Agilent G6520 Q-TOF	**Acq Method**	20160322_MS_ESIH_POS_1min.m
**Acquired Time**	1/4/2023 10:47:05	**IRM Calibration Status**	Success
**DA Method**	small molecular data analysis method.m	**Comment**	ESIH by fangsu

User Spectra

Fragmentor Voltage	Collision Energy	Ionization Mode
170	0	ESI

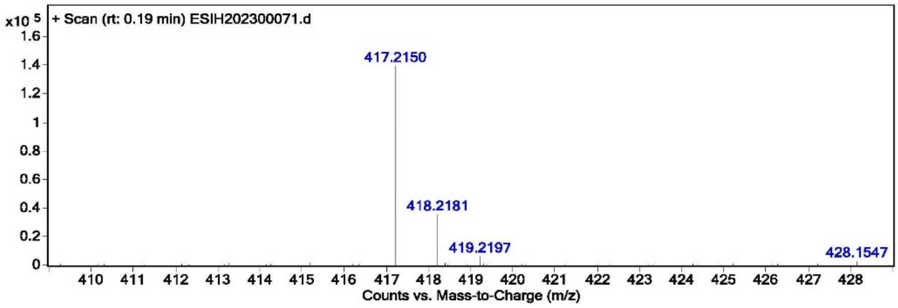

Formula Calculator Results

m/z	Calc m/z	Diff (mDa)	Diff (ppm)	Ion Formula	Ion
417.215	417.2149	-0.15	-0.36	C24 H30 N2 Na O3	(M+Na)+

--- End Of Report ---

8e

Qualitative Analysis Report

**Data Filename**	ESIH202300394.d	**Sample Name**	D8-CD153-B
**Sample ID**		**Position**	P1-B9
**Instrument Name**	Agilent G6520 Q-TOF	**Acq Method**	20160322_MS_ESIH_POS_1min.m
**Acquired Time**	1/11/2023 13:38:14	**IRM Calibration Status**	Success
**DA Method**	small molecular data analysis method.m	**Comment**	ESIH by fangsu

User Spectra

Fragmentor Voltage	Collision Energy	Ionization Mode
170	0	ESI

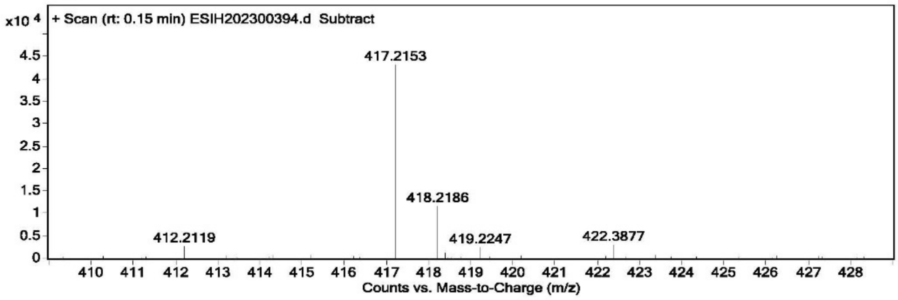

Formula Calculator Results

m/z	Calc m/z	Diff (mDa)	Diff (ppm)	Ion Formula	Ion
417.2153	417.2149	-0.47	-1.12	C24 H30 N2 Na O3	(M+Na)+

--- End Of Report ---

8e'

Qualitative Analysis Report

**Data Filename**	ESIH202300069.d	**Sample Name**	D8-CD149
**Sample ID**		**Position**	P1-A7
**Instrument Name**	Agilent G6520 Q-TOF	**Acq Method**	20160322_MS_ESIH_POS_1min.m
**Acquired Time**	1/4/2023 10:44:33	**IRM Calibration Status**	Success
**DA Method**	small molecular data analysis method.m	**Comment**	ESIH by fangsu

User Spectra

Fragmentor Voltage	Collision Energy	Ionization Mode
170	0	ESI

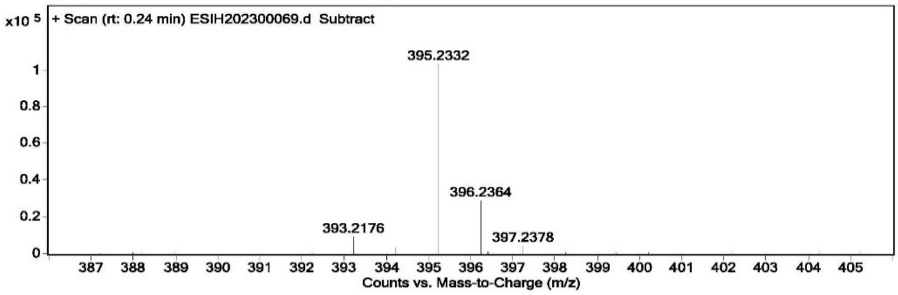

Formula Calculator Results

m/z	Calc m/z	Diff (mDa)	Diff (ppm)	Ion Formula	Ion
395.2332	395.2329	-0.26	-0.66	C24 H31 N2 O3	(M+H)+

--- End Of Report ---

8f

Qualitative Analysis Report

**Data Filename**	ESIH202205831.d	**Sample Name**	D8-CD140
**Sample ID**		**Position**	P1-D8
**Instrument Name**	Agilent G6520 Q-TOF	**Acq Method**	20160322_MS_ESIH_POS_1min.m
**Acquired Time**	12/21/2022 17:01:51	**IRM Calibration Status**	Success
**DA Method**	small molecular data analysis method.m	**Comment**	ESIH by zhuzhenyun

User Spectra

Fragmentor Voltage	Collision Energy	Ionization Mode
170	0	ESI

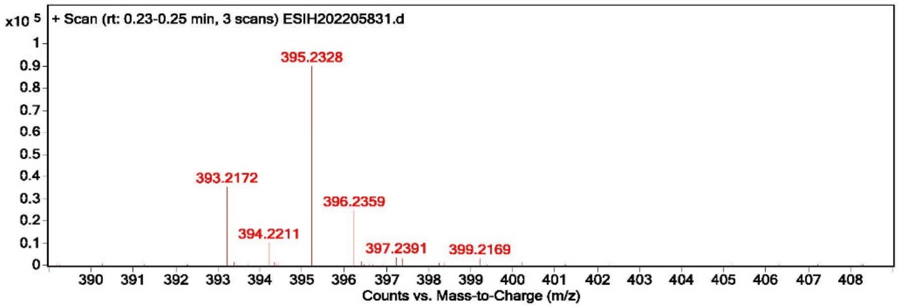

Formula Calculator Results

m/z	Calc m/z	Diff (mDa)	Diff (ppm)	Ion Formula	Ion
395.2328	395.2329	0.12	0.31	C24 H31 N2 O3	(M+H)+

--- End Of Report ---

Agilent Technologies Page 1 of 1 Printed at: 17:20 on: 12/21/2022

8g

Qualitative Analysis Report

**Data Filename**	ESIH202300003.d	**Sample Name**	D8-CD107
**Sample ID**		**Position**	P1-A3
**Instrument Name**	Agilent G6520 Q-TOF	**Acq Method**	20160322_MS_ESIH_POS_1min.m
**Acquired Time**	1/3/2023 13:20:00	**IRM Calibration Status**	Success
**DA Method**	small molecular data analysis method.m	**Comment**	ESIH by fangsu

User Spectra

Fragmentor Voltage	Collision Energy	Ionization Mode
170	0	ESI

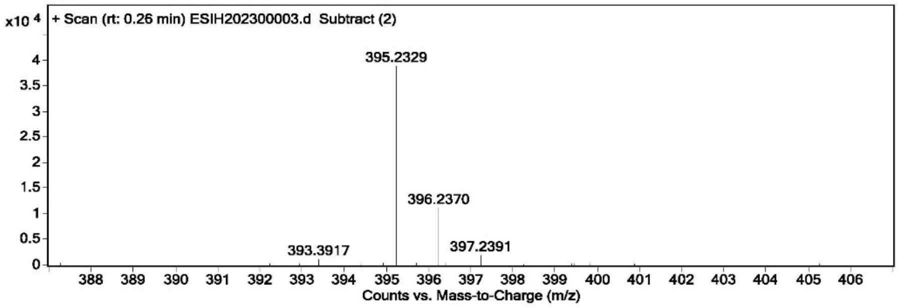

Formula Calculator Results

m/z	Calc m/z	Diff (mDa)	Diff (ppm)	Ion Formula	Ion
395.2329	395.2329	0.06	0.15	C24 H31 N2 O3	(M+H)+

--- End Of Report ---

Agilent Technologies Page 1 of 1 Printed at: 13:37 on: 1/3/2023

8h

Qualitative Analysis Report

**Data Filename**	ESIH202300070.d	**Sample Name**	D8-CD151
**Sample ID**		**Position**	P1-A8
**Instrument Name**	Agilent G6520 Q-TOF	**Acq Method**	20160322_MS_ESIH_POS_1min.m
**Acquired Time**	1/4/2023 10:45:51	**IRM Calibration Status**	Success
**DA Method**	small molecular data analysis method.m	**Comment**	ESIH by fangsu

User Spectra

Fragmentor Voltage	Collision Energy	Ionization Mode
170	0	ESI

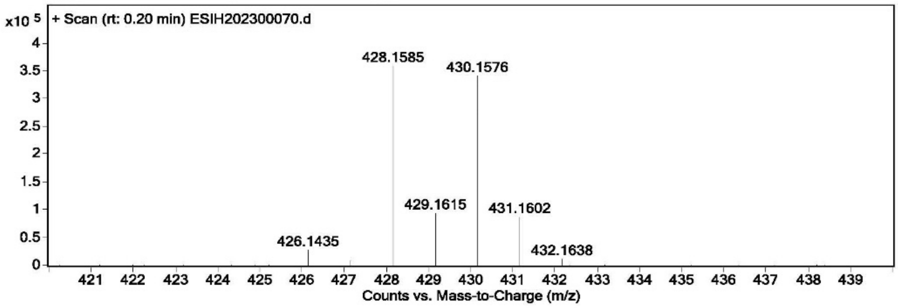

Formula Calculator Results

m/z	Calc m/z	Diff (mDa)	Diff (ppm)	Ion Formula	Ion
428.1585	428.1584	-0.14	-0.32	C24 H31 Br N O	(M+H)+

--- End Of Report ---

Agilent Technologies Page 1 of 1 Printed at: 10:46 on: 1/4/2023

8i

Qualitative Analysis Report

**Data Filename**	ESIH202300065.d	**Sample Name**	D8-CD115
**Sample ID**		**Position**	P1-A3
**Instrument Name**	Agilent G6520 Q-TOF	**Acq Method**	20160322_MS_ESIH_POS_1min.m
**Acquired Time**	1/4/2023 10:39:26	**IRM Calibration Status**	Success
**DA Method**	small molecular data analysis method.m	**Comment**	ESIH by fangsu

User Spectra

Fragmentor Voltage	Collision Energy	Ionization Mode
170	0	ESI

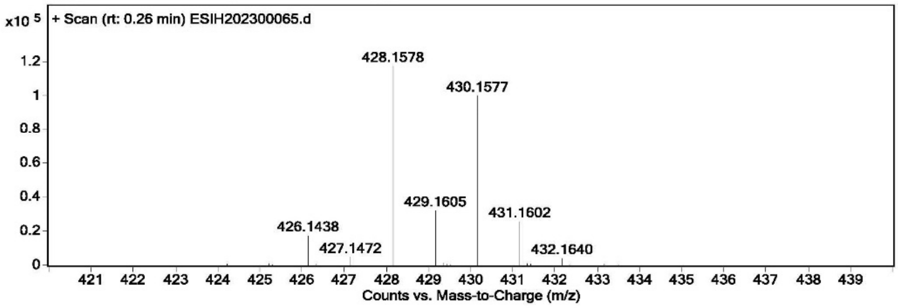

Formula Calculator Results

m/z	Calc m/z	Diff (mDa)	Diff (ppm)	Ion Formula	Ion
428.1578	428.1584	0.51	1.19	C24 H31 Br N O	(M+H)+

--- End Of Report ---

Agilent Technologies Page 1 of 1 Printed at: 10:41 on: 1/4/2023

8j

Qualitative Analysis Report

**Data Filename**	ESIH202300002.d	**Sample Name**	D8-CD105
**Sample ID**		**Position**	P1-A2
**Instrument Name**	Agilent G6520 Q-TOF	**Acq Method**	20160322_MS_ESIH_POS_1min.m
**Acquired Time**	1/3/2023 13:18:44	**IRM Calibration Status**	Success
**DA Method**	small molecular data analysis method.m	**Comment**	ESIH by fangsu

User Spectra

Fragmentor Voltage	Collision Energy	Ionization Mode
170	0	ESI

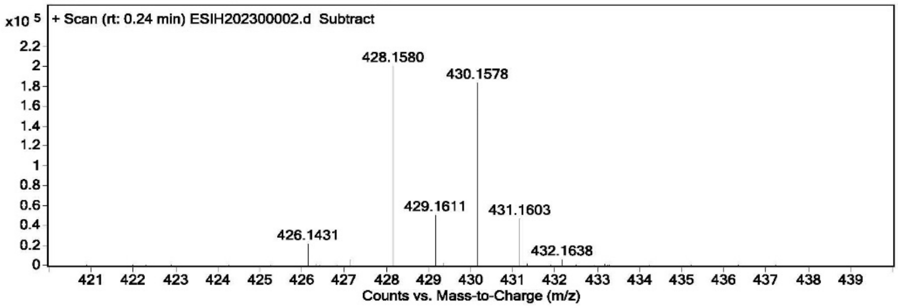

Formula Calculator Results

m/z	Calc m/z	Diff (mDa)	Diff (ppm)	Ion Formula	Ion
428.158	428.1584	0.36	0.85	C24 H31 Br N O	(M+H)+

--- End Of Report ---

8k

Qualitative Analysis Report

**Data Filename**	ESIH202206031.d	**Sample Name**	D8-CD114
**Sample ID**		**Position**	P1-A9
**Instrument Name**	Agilent G6520 Q-TOF	**Acq Method**	20160322_MS_ESIH_POS_1min.m
**Acquired Time**	12/29/2022 16:28:55	**IRM Calibration Status**	Success
**DA Method**	small molecular data analysis method.m	**Comment**	ESIH by fangsu

User Spectra

Fragmentor Voltage	Collision Energy	Ionization Mode
170	0	ESI

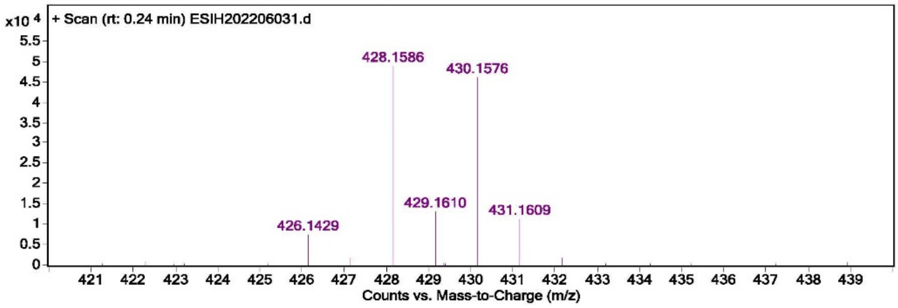

Formula Calculator Results

m/z	Calc m/z	Diff (mDa)	Diff (ppm)	Ion Formula	Ion
428.1586	428.1584	-0.25	-0.58	C24 H31 Br N O	(M+H)+

--- End Of Report ---

8l

Qualitative Analysis Report

**Data Filename**	ESIH202300001.d	**Sample Name**	D8-CD103
**Sample ID**		**Position**	P1-A1
**Instrument Name**	Agilent G6520 Q-TOF	**Acq Method**	20160322_MS_ESIH_POS_1min.m
**Acquired Time**	1/3/2023 13:17:23	**IRM Calibration Status**	Success
**DA Method**	small molecular data analysis method.m	**Comment**	ESIH by fangsu

User Spectra

Fragmentor Voltage	Collision Energy	Ionization Mode
170	0	ESI

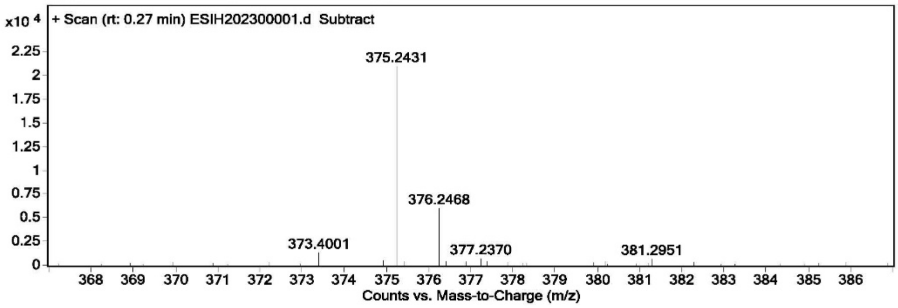

Formula Calculator Results

m/z	Calc m/z	Diff (mDa)	Diff (ppm)	Ion Formula	Ion
375.2431	375.2431	0	0	C25 H31 N2 O	(M+H)+

--- End Of Report ---

Agilent Technologies Page 1 of 1 Printed at: 13:35 on: 1/3/2023

8m

Qualitative Analysis Report

**Data Filename**	ESIH202300005.d	**Sample Name**	D8-CD112
**Sample ID**		**Position**	P1-A5
**Instrument Name**	Agilent G6520 Q-TOF	**Acq Method**	20160322_MS_ESIH_POS_1min.m
**Acquired Time**	1/3/2023 13:22:32	**IRM Calibration Status**	Success
**DA Method**	small molecular data analysis method.m	**Comment**	ESIH by fangsu

User Spectra

Fragmentor Voltage	Collision Energy	Ionization Mode
170	0	ESI

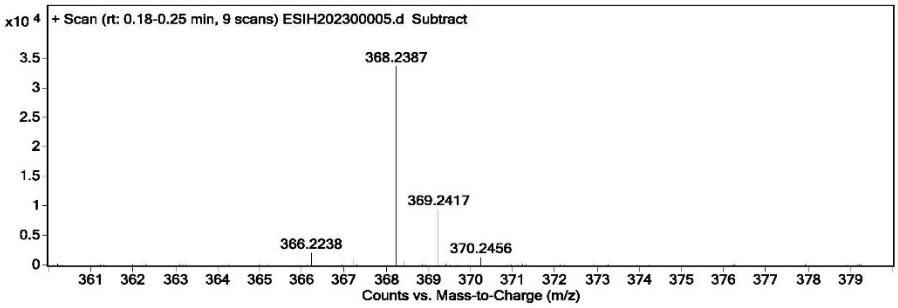

Formula Calculator Results

m/z	Calc m/z	Diff (mDa)	Diff (ppm)	Ion Formula	Ion
368.2387	368.2384	-0.3	-0.82	C24 H31 F N O	(M+H)+

--- End Of Report ---

Agilent Technologies Page 1 of 1 Printed at: 13:39 on: 1/3/2023

8n

Qualitative Analysis Report

**Data Filename**	ESIH202300004.d	**Sample Name**	D8-CD111
**Sample ID**		**Position**	P1-A4
**Instrument Name**	Agilent G6520 Q-TOF	**Acq Method**	20160322_MS_ESIH_POS_1min.m
**Acquired Time**	1/3/2023 13:21:15	**IRM Calibration Status**	Success
**DA Method**	small molecular data analysis method.m	**Comment**	ESIH by fangsu

User Spectra

Fragmentor Voltage	Collision Energy	Ionization Mode
170	0	ESI

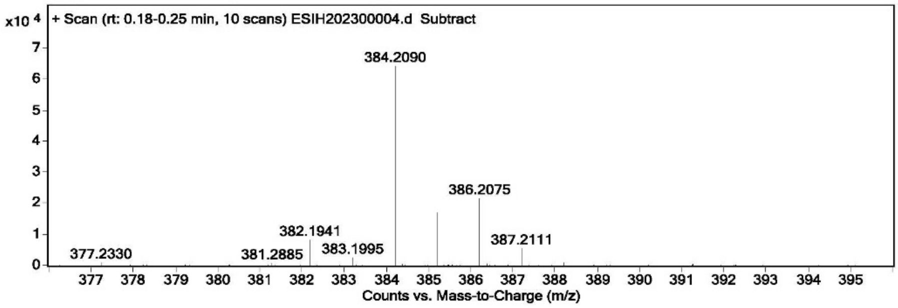

Formula Calculator Results

m/z	Calc m/z	Diff (mDa)	Diff (ppm)	Ion Formula	Ion
384.209	384.2089	-0.14	-0.35	C24 H31 Cl N O	(M+H)+

--- End Of Report ---

Agilent Technologies Page 1 of 1 Printed at: 13:38 on: 1/3/2023

8p

Qualitative Analysis Report

**Data Filename**	ESIH202300007.d	**Sample Name**	D8-CD137
**Sample ID**		**Position**	P1-A7
**Instrument Name**	Agilent G6520 Q-TOF	**Acq Method**	20160322_MS_ESIH_POS_1min.m
**Acquired Time**	1/3/2023 13:25:07	**IRM Calibration Status**	Success
**DA Method**	small molecular data analysis method.m	**Comment**	ESIH by fangsu

User Spectra

Fragmentor Voltage	Collision Energy	Ionization Mode
170	0	ESI

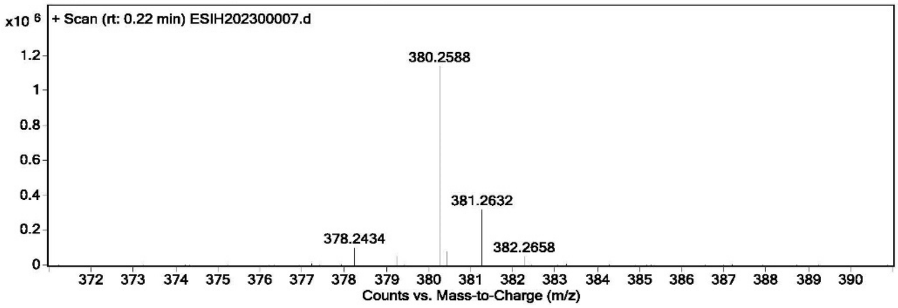

Formula Calculator Results

m/z	Calc m/z	Diff (mDa)	Diff (ppm)	Ion Formula	Ion
380.2588	380.2584	-0.42	-1.11	C25 H34 N O2	(M+H)+

--- End Of Report ---

Agilent Technologies Page 1 of 1 Printed at: 13:41 on: 1/3/2023

8q

Qualitative Analysis Report

**Data Filename**	ESIH202300011.d	**Sample Name**	D8-CD154
**Sample ID**		**Position**	P1-B2
**Instrument Name**	Agilent G6520 Q-TOF	**Acq Method**	20160322_MS_ESIH_POS_1min.m
**Acquired Time**	1/3/2023 13:30:14	**IRM Calibration Status**	Success
**DA Method**	small molecular data analysis method.m	**Comment**	ESIH by fangsu

User Spectra

Fragmentor Voltage	Collision Energy	Ionization Mode
170	0	ESI

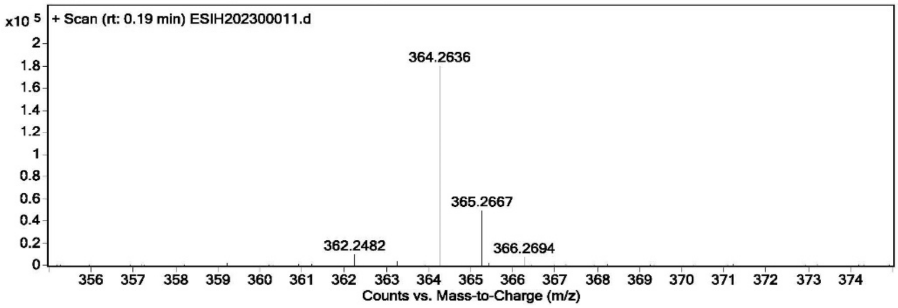

Formula Calculator Results

m/z	Calc m/z	Diff (mDa)	Diff (ppm)	Ion Formula	Ion
364.2636	364.2635	-0.12	-0.33	C25 H34 N O	(M+H)+

--- End Of Report ---

8r

Qualitative Analysis Report

**Data Filename**	N_ESIH202300010.d	**Sample Name**	D8-CD147
**Sample ID**		**Position**	P1-B1
**Instrument Name**	Agilent G6520 Q-TOF	**Acq Method**	20160324_MS_ESIH_NEG_1min.m
**Acquired Time**	1/3/2023 14:01:06	**IRM Calibration Status**	Success
**DA Method**	small molecular data analysis method.m	**Comment**	ESIH by fangsu

User Spectra

Fragmentor Voltage	Collision Energy	Ionization Mode
170	0	ESI

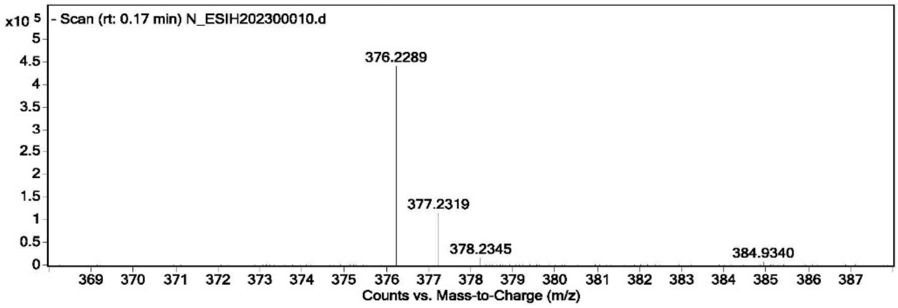

Formula Calculator Results

m/z	Calc m/z	Diff (mDa)	Diff (ppm)	Ion Formula	Ion
376.2289	376.2282	-0.67	-1.77	C25 H30 N O2	(M-H)-

--- End Of Report ---

8s

Qualitative Analysis Report

**Data Filename**	ESIH202300014.d	**Sample Name**	D8-CE030
**Sample ID**		**Position**	P1-B5
**Instrument Name**	Agilent G6520 Q-TOF	**Acq Method**	20160322_MS_ESIH_POS_1min.m
**Acquired Time**	1/3/2023 13:34:05	**IRM Calibration Status**	Success
**DA Method**	small molecular data analysis method.m	**Comment**	ESIH by fangsu

User Spectra

Fragmentor Voltage	Collision Energy	Ionization Mode
170	0	ESI

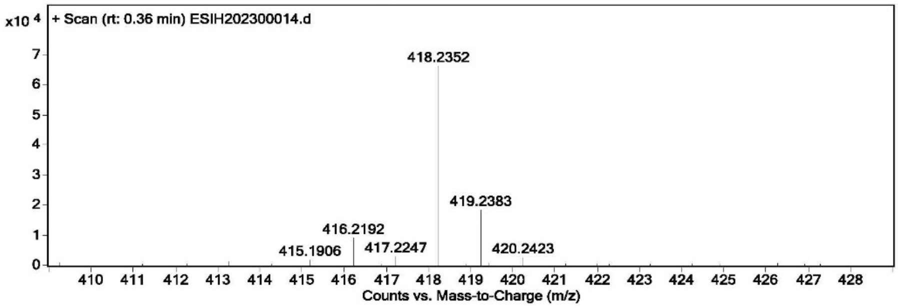

Formula Calculator Results

m/z	Calc m/z	Diff (mDa)	Diff (ppm)	Ion Formula	Ion
418.2352	418.2352	0.05	0.11	C25 H31 F3 N O	(M+H)+

--- End Of Report ---

8t

Qualitative Analysis Report

**Data Filename**	ESIH202300012.d	**Sample Name**	D8-CE016
**Sample ID**		**Position**	P1-B3
**Instrument Name**	Agilent G6520 Q-TOF	**Acq Method**	20160322_MS_ESIH_POS_1min.m
**Acquired Time**	1/3/2023 13:31:31	**IRM Calibration Status**	Success
**DA Method**	small molecular data analysis method.m	**Comment**	ESIH by fangsu

User Spectra

Fragmentor Voltage	Collision Energy	Ionization Mode
170	0	ESI

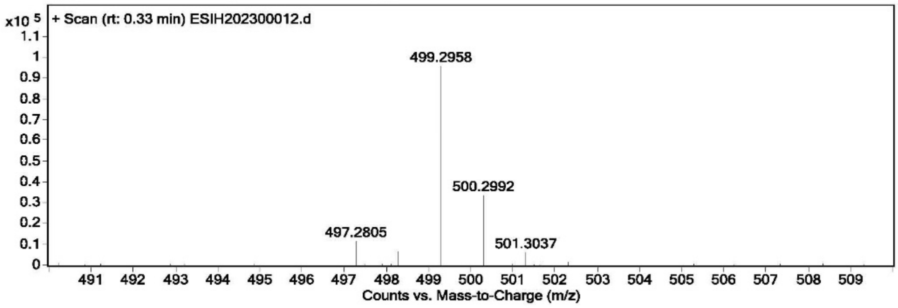

Formula Calculator Results

m/z	Calc m/z	Diff (mDa)	Diff (ppm)	Ion Formula	Ion
499.2958	499.2955	-0.29	-0.57	C32 H39 N2 O3	(M+H)+

--- End Of Report ---

8u

Qualitative Analysis Report

**Data Filename**	ESIH202300013.d	**Sample Name**	D8-CE028
**Sample ID**		**Position**	P1-B4
**Instrument Name**	Agilent G6520 Q-TOF	**Acq Method**	20160322_MS_ESIH_POS_1min.m
**Acquired Time**	1/3/2023 13:32:48	**IRM Calibration Status**	Success
**DA Method**	small molecular data analysis method.m	**Comment**	ESIH by fangsu

User Spectra

Fragmentor Voltage	Collision Energy	Ionization Mode
170	0	ESI

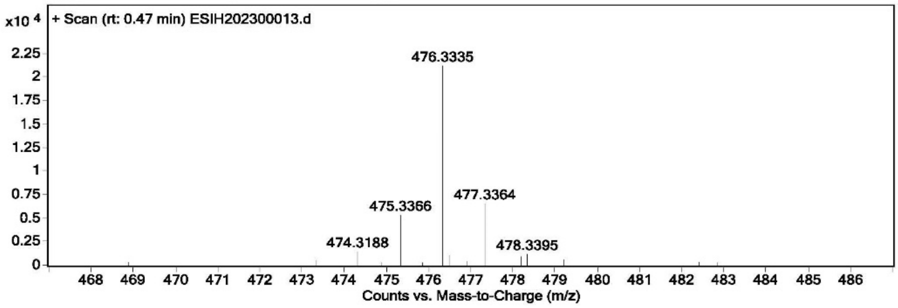

Formula Calculator Results

m/z	Calc m/z	Diff (mDa)	Diff (ppm)	Ion Formula	Ion
476.3335	476.3331	-0.47	-0.99	C30 H43 [11B] N O3	(M+H)+

--- End Of Report ---

Agilent Technologies Page 1 of 1 Printed at: 13:48 on: 1/3/2023

8v

Qualitative Analysis Report

**Data Filename**	ESIH202300015.d	**Sample Name**	D8-CE036
**Sample ID**		**Position**	P1-B6
**Instrument Name**	Agilent G6520 Q-TOF	**Acq Method**	20160322_MS_ESIH_POS_1min.m
**Acquired Time**	1/3/2023 13:35:22	**IRM Calibration Status**	Success
**DA Method**	small molecular data analysis method.m	**Comment**	ESIH by fangsu

User Spectra

Fragmentor Voltage	Collision Energy	Ionization Mode
170	0	ESI

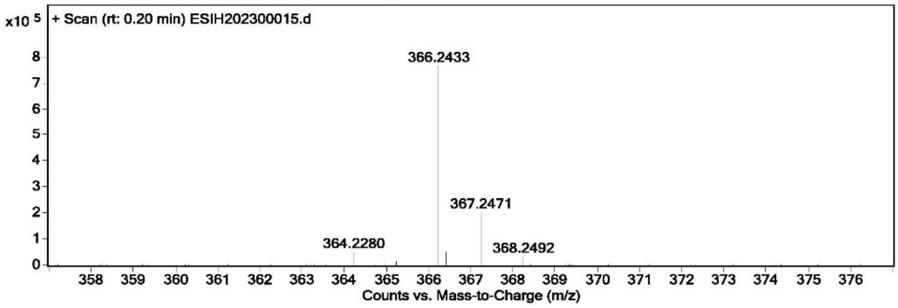

Formula Calculator Results

m/z	Calc m/z	Diff (mDa)	Diff (ppm)	Ion Formula	Ion
366.2433	366.2428	-0.51	-1.4	C24 H32 N O2	(M+H)+

--- End Of Report ---

Agilent Technologies Page 1 of 1 Printed at: 13:49 on: 1/3/2023

8w

Qualitative Analysis Report

**Data Filename**	ESIH202206033.d	**Sample Name**	D8-CD117
**Sample ID**		**Position**	P1-B2
**Instrument Name**	Agilent G6520 Q-TOF	**Acq Method**	20160322_MS_ESIH_POS_1min.m
**Acquired Time**	12/29/2022 16:31:30	**IRM Calibration Status**	Success
**DA Method**	small molecular data analysis method.m	**Comment**	ESIH by fangsu

User Spectra

Fragmentor Voltage	Collision Energy	Ionization Mode
170	0	ESI

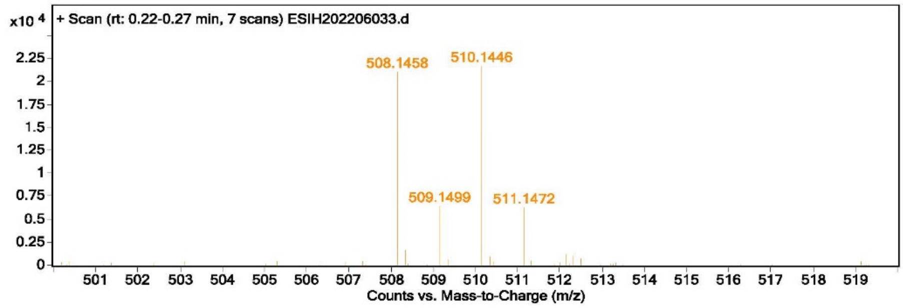

Formula Calculator Results

m/z	Calc m/z	Diff (mDa)	Diff (ppm)	Ion Formula	Ion
508.1458	508.1458	-0.07	-0.13	C26 H32 Br N Na O3	(M+Na)+

--- End Of Report ---

8x

Qualitative Analysis Report

**Data Filename**	ESIH202300078.d	**Sample Name**	D8-CD117-B
**Sample ID**		**Position**	P1-B7
**Instrument Name**	Agilent G6520 Q-TOF	**Acq Method**	20160322_MS_ESIH_POS_1min.m
**Acquired Time**	1/4/2023 10:56:07	**IRM Calibration Status**	Success
**DA Method**	small molecular data analysis method.m	**Comment**	ESIH by fangsu

User Spectra

Fragmentor Voltage	Collision Energy	Ionization Mode
170	0	ESI

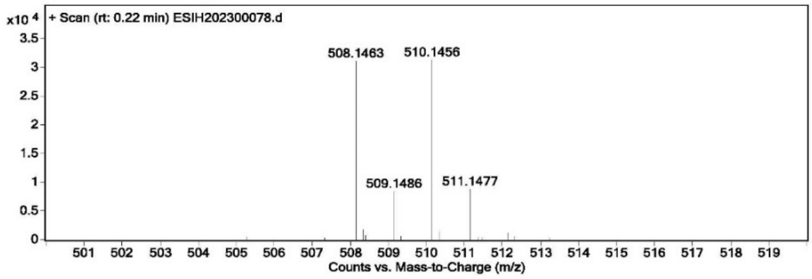

Formula Calculator Results

m/z	Calc m/z	Diff (mDa)	Diff (ppm)	Ion Formula	Ion
508.1463	508.1458	-0.54	-1.06	C26 H32 Br N Na O3	(M+Na)+

--- End Of Report ---

8x’

Qualitative Analysis Report

**Data Filename**	ESIH202300066.d	**Sample Name**	D8-CD124
**Sample ID**		**Position**	P1-A4
**Instrument Name**	Agilent G6520 Q-TOF	**Acq Method**	20160322_MS_ESIH_POS_1min.m
**Acquired Time**	1/4/2023 10:40:42	**IRM Calibration Status**	Success
**DA Method**	small molecular data analysis method.m	**Comment**	ESIH by fangsu

User Spectra

Fragmentor Voltage	Collision Energy	Ionization Mode
170	0	ESI

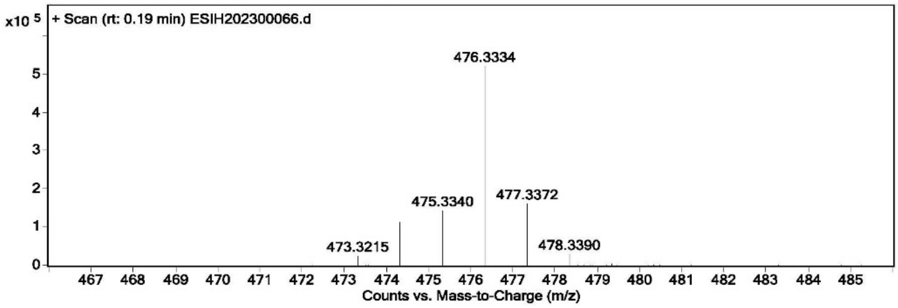

Formula Calculator Results

m/z	Calc m/z	Diff (mDa)	Diff (ppm)	Ion Formula	Ion
476.3334	476.3331	-0.38	-0.8	C30 H43 [11B] N O3	(M+H)+

--- End Of Report ---

8y

Qualitative Analysis Report

**Data Filename**	ESIH202205830.d	**Sample Name**	D8-CD109
**Sample ID**		**Position**	P1-D7
**Instrument Name**	Agilent G6520 Q-TOF	**Acq Method**	20160322_MS_ESIH_POS_1min.m
**Acquired Time**	12/21/2022 17:00:34	**IRM Calibration Status**	Success
**DA Method**	small molecular data analysis method.m	**Comment**	ESIH by zhuzhenyun

User Spectra

Fragmentor Voltage	Collision Energy	Ionization Mode
170	0	ESI

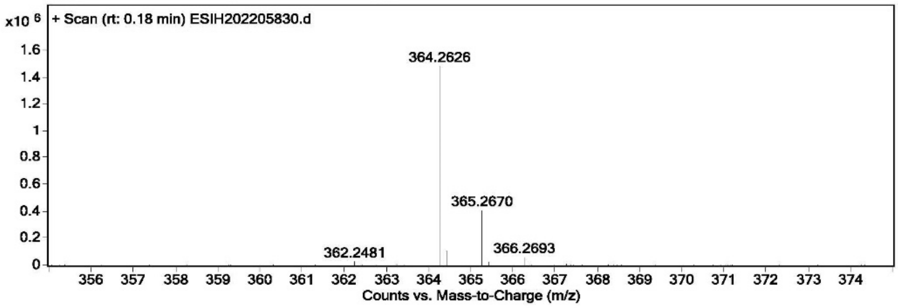

Formula Calculator Results

m/z	Calc m/z	Diff (mDa)	Diff (ppm)	Ion Formula	Ion
364.2626	364.2635	0.87	2.4	C25 H34 N O	(M+H)+

--- End Of Report ---

8ab

Qualitative Analysis Report

**Data Filename**	ESIH202300008.d	**Sample Name**	D8-CD138
**Sample ID**		**Position**	P1-A8
**Instrument Name**	Agilent G6520 Q-TOF	**Acq Method**	20160322_MS_ESIH_POS_1min.m
**Acquired Time**	1/3/2023 13:26:24	**IRM Calibration Status**	Success
**DA Method**	small molecular data analysis method.m	**Comment**	ESIH by fangsu

User Spectra

Fragmentor Voltage	Collision Energy	Ionization Mode
170	0	ESI

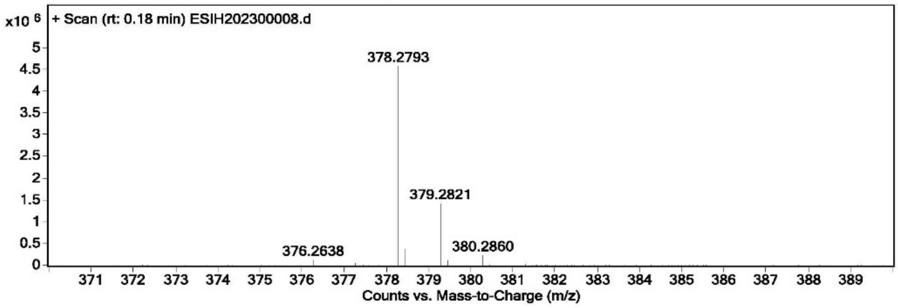

Formula Calculator Results

m/z	Calc m/z	Diff (mDa)	Diff (ppm)	Ion Formula	Ion
378.2793	378.2791	-0.13	-0.35	C26 H36 N O	(M+H)+

--- End Of Report ---

Agilent Technologies Page 1 of 1 Printed at: 13:41 on: 1/3/2023

8ac

Qualitative Analysis Report

**Data Filename**	ESIH202300009.d	**Sample Name**	D8-CD144
**Sample ID**		**Position**	P1-A9
**Instrument Name**	Agilent G6520 Q-TOF	**Acq Method**	20160322_MS_ESIH_POS_1min.m
**Acquired Time**	1/3/2023 13:27:39	**IRM Calibration Status**	Success
**DA Method**	small molecular data analysis method.m	**Comment**	ESIH by fangsu

User Spectra

Fragmentor Voltage	Collision Energy	Ionization Mode
170	0	ESI

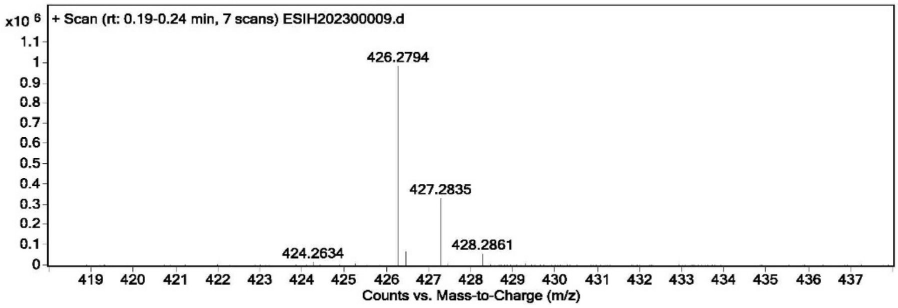

Formula Calculator Results

m/z	Calc m/z	Diff (mDa)	Diff (ppm)	Ion Formula	Ion
426.2794	426.2791	-0.31	-0.72	C30 H36 N O	(M+H)+

--- End Of Report ---

Agilent Technologies Page 1 of 1 Printed at: 13:42 on: 1/3/2023

8ad

Qualitative Analysis Report

**Data Filename**	ESIH202300064.d	**Sample Name**	D8-CD143
**Sample ID**		**Position**	P1-A2
**Instrument Name**	Agilent G6520 Q-TOF	**Acq Method**	20160322_MS_ESIH_POS_1min.m
**Acquired Time**	1/4/2023 10:38:09	**IRM Calibration Status**	Success
**DA Method**	small molecular data analysis method.m	**Comment**	ESIH by fangsu

User Spectra

Fragmentor Voltage	Collision Energy	Ionization Mode
170	0	ESI

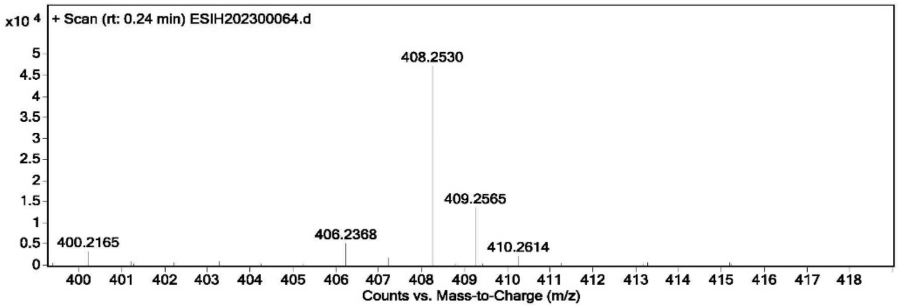

Formula Calculator Results

m/z	Calc m/z	Diff (mDa)	Diff (ppm)	Ion Formula	Ion
408.253	408.2533	0.34	0.83	C26 H34 N O3	(M+H)+

--- End Of Report ---

8ae

Qualitative Analysis Report

**Data Filename**	ESIH202300077.d	**Sample Name**	D8-CD156
**Sample ID**		**Position**	P1-B6
**Instrument Name**	Agilent G6520 Q-TOF	**Acq Method**	20160322_MS_ESIH_POS_1min.m
**Acquired Time**	1/4/2023 10:54:51	**IRM Calibration Status**	Success
**DA Method**	small molecular data analysis method.m	**Comment**	ESIH by fangsu

User Spectra

Fragmentor Voltage	Collision Energy	Ionization Mode
170	0	ESI

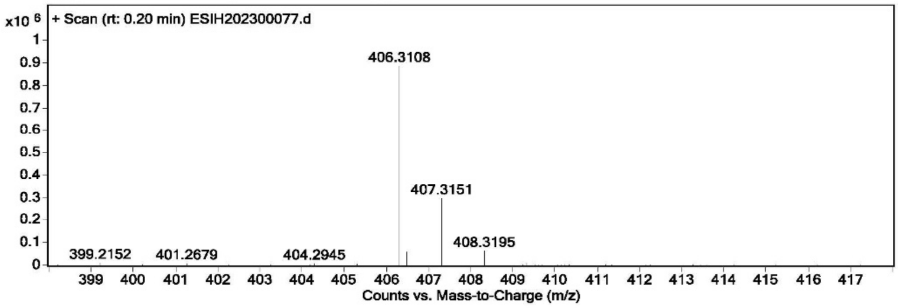

Formula Calculator Results

m/z	Calc m/z	Diff (mDa)	Diff (ppm)	Ion Formula	Ion
406.3108	406.3104	-0.35	-0.86	C28 H40 N O	(M+H)+

--- End Of Report ---

8af

Qualitative Analysis Report

**Data Filename**	ESIH202300072.d	**Sample Name**	D8-CE032
**Sample ID**		**Position**	P1-B1
**Instrument Name**	Agilent G6520 Q-TOF	**Acq Method**	20160322_MS_ESIH_POS_1min.m
**Acquired Time**	1/4/2023 10:48:22	**IRM Calibration Status**	Success
**DA Method**	small molecular data analysis method.m	**Comment**	ESIH by fangsu

User Spectra

Fragmentor Voltage	Collision Energy	Ionization Mode
170	0	ESI

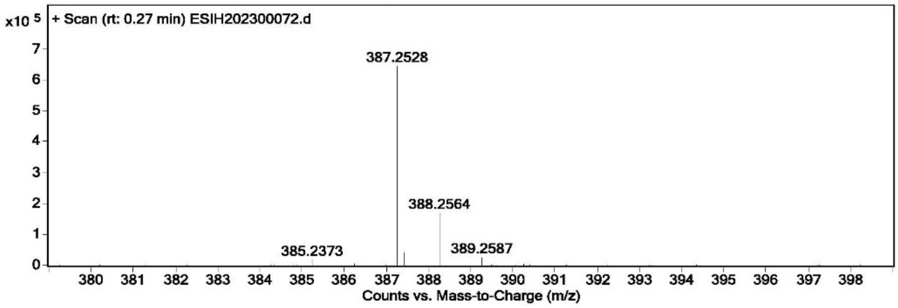

Formula Calculator Results

m/z	Calc m/z	Diff (mDa)	Diff (ppm)	Ion Formula	Ion
387.2528	387.253	0.16	0.41	C24 H35 O4	(M+H)+

--- End Of Report ---

Agilent Technologies Page 1 of 1 Printed at: 10:50 on: 1/4/2023

9

Qualitative Analysis Report

**Data Filename**	ESIH202300073.d	**Sample Name**	D8-CE045
**Sample ID**		**Position**	P1-B2
**Instrument Name**	Agilent G6520 Q-TOF	**Acq Method**	20160322_MS_ESIH_POS_1min.m
**Acquired Time**	1/4/2023 10:49:45	**IRM Calibration Status**	Success
**DA Method**	small molecular data analysis method.m	**Comment**	ESIH by fangsu

User Spectra

Fragmentor Voltage	Collision Energy	Ionization Mode
170	0	ESI

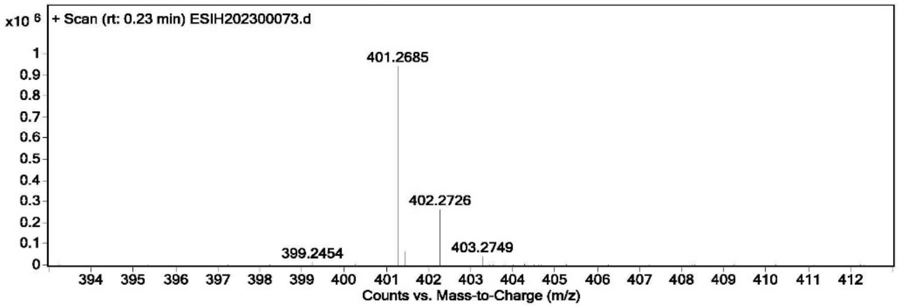

Formula Calculator Results

m/z	Calc m/z	Diff (mDa)	Diff (ppm)	Ion Formula	Ion
401.2685	401.2686	0.13	0.32	C25 H37 O4	(M+H)+

--- End Of Report ---

Agilent Technologies Page 1 of 1 Printed at: 10:51 on: 1/4/2023

10

Qualitative Analysis Report

**Data Filename**	ESIH202300076.d	**Sample Name**	D8-CE069
**Sample ID**		**Position**	P1-B5
**Instrument Name**	Agilent G6520 Q-TOF	**Acq Method**	20160322_MS_ESIH_POS_1min.m
**Acquired Time**	1/4/2023 10:53:35	**IRM Calibration Status**	Success
**DA Method**	small molecular data analysis method.m	**Comment**	ESIH by fangsu

User Spectra

**Fragmentor Voltage**	**Collision Energy**	**Ionization Mode**
170	0	ESI

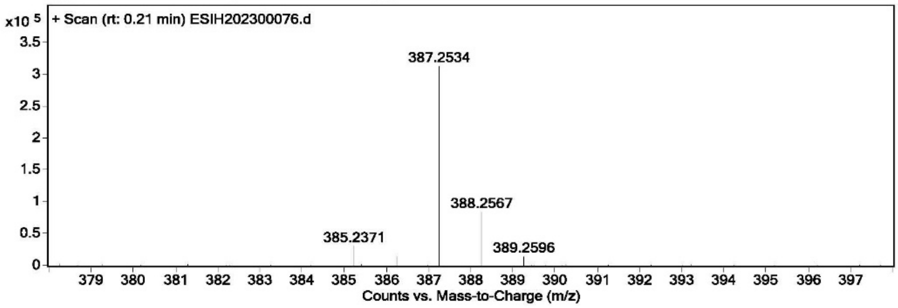

Formula Calculator Results

m/z	Calc m/z	Diff (mDa)	Diff (ppm)	Ion Formula	Ion
387.2534	387.253	-0.42	-1.08	C24 H35 O4	(M+H)+

--- End Of Report ---

Agilent Technologies Page 1 of 1 Printed at: 10:56 on: 1/4/2023

11

Qualitative Analysis Report

**Data Filename**	ESIH202300075.d	**Sample Name**	D8-CE068
**Sample ID**		**Position**	P1-B4
**Instrument Name**	Agilent G6520 Q-TOF	**Acq Method**	20160322_MS_ESIH_POS_1min.m
**Acquired Time**	1/4/2023 10:52:18	**IRM Calibration Status**	Success
**DA Method**	small molecular data analysis method.m	**Comment**	ESIH by fangsu

User Spectra

**Fragmentor Voltage**	**Collision Energy**	**Ionization Mode**
170	0	ESI

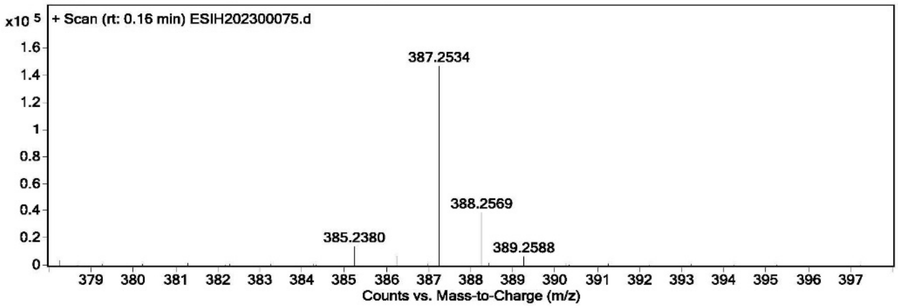

Formula Calculator Results

m/z	Calc m/z	Diff (mDa)	Diff (ppm)	Ion Formula	Ion
387.2534	387.253	-0.44	-1.14	C24 H35 O4	(M+H)+

--- End Of Report ---

Agilent Technologies Page 1 of 1 Printed at: 10:55 on: 1/4/2023

12

Qualitative Analysis Report

**Data Filename**	ESIH202300074.d	**Sample Name**	D8-CE067
**Sample ID**		**Position**	P1-B3
**Instrument Name**	Agilent G6520 Q-TOF	**Acq Method**	20160322_MS_ESIH_POS_1min.m
**Acquired Time**	1/4/2023 10:51:01	**IRM Calibration Status**	Success
**DA Method**	small molecular data analysis method.m	**Comment**	ESIH by fangsu

User Spectra

Fragmentor Voltage	Collision Energy	Ionization Mode
170	0	ESI

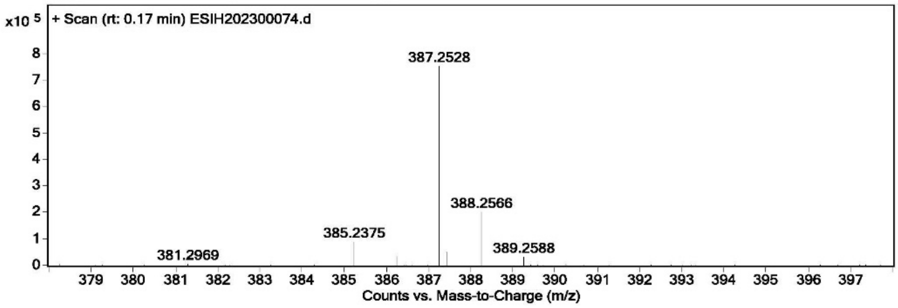

Formula Calculator Results

m/z	Calc m/z	Diff (mDa)	Diff (ppm)	Ion Formula	Ion
387.2528	387.253	0.17	0.44	C24 H35 O4	(M+H)+

--- End Of Report ---

Agilent Technologies   Page 1 of 1   Printed at: 10:52 on: 1/4/2023

13

Qualitative Analysis Report

**Data Filename**	ESIH202206032.d	**Sample Name**	D8-CE051
**Sample ID**		**Position**	P1-B1
**Instrument Name**	Agilent G6520 Q-TOF	**Acq Method**	20160322_MS_ESIH_POS_1min.m
**Acquired Time**	12/29/2022 16:30:13	**IRM Calibration Status**	Success
**DA Method**	small molecular data analysis method.m	**Comment**	ESIH by fangsu

User Spectra

Fragmentor Voltage	Collision Energy	Ionization Mode
170	0	ESI

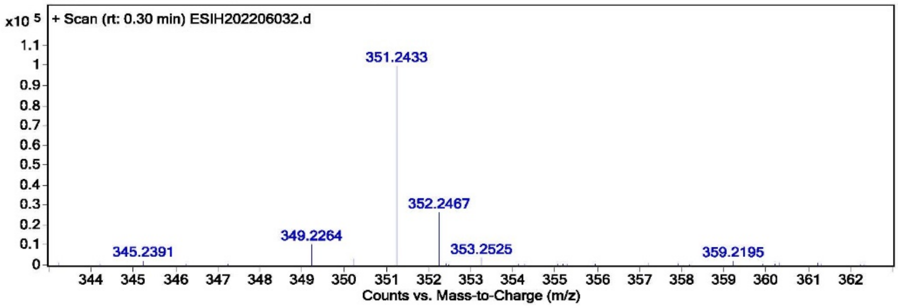

Formula Calculator Results

m/z	Calc m/z	Diff (mDa)	Diff (ppm)	Ion Formula	Ion
351.2433	351.2431	-0.24	-0.68	C23 H31 N2 O	(M+H)+

--- End Of Report ---

Agilent Technologies   Page 1 of 1   Printed at: 16:55 on: 12/29/2022

14

5. UPLC purity reports of final compounds

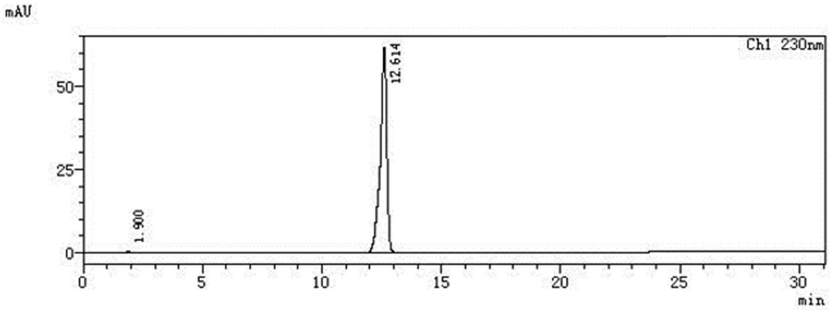

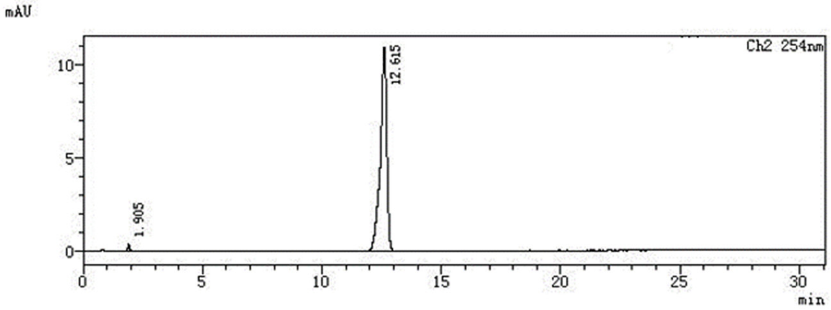

	Retention Time (Min)	Area	Peak Height	%Area
1	1.900	3178	563	0.270
2	12.614	1175810	61603	99.730
sum		1178989	62166	100.000

	Retention Time (Min)	Area	Peak Height	%Area
1	1.905	2007	356	0.967
2	12.615	205609	10878	99.033
sum		207616	11234	100.000

6 @ 230 nm & 254 nm

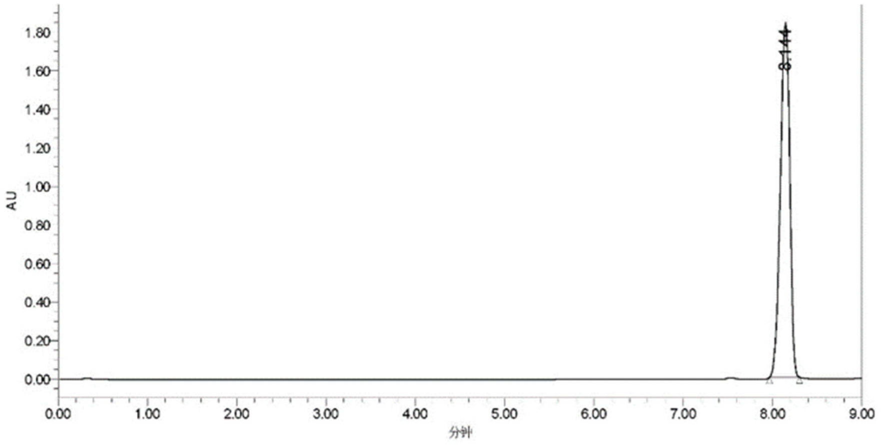

		Retention Time (Min)	Area	Peak Height	%Area
1		8.144	13517588	1850156	100.00
Sum					100.0

8a @230 nm

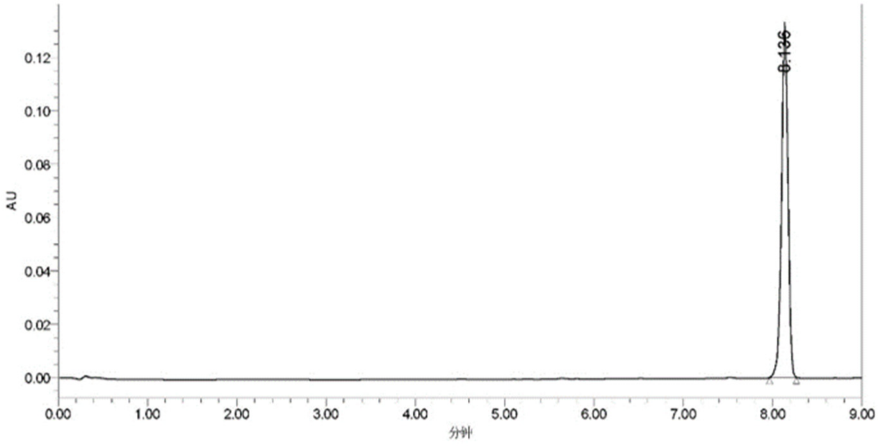

		Retention Time (Min)	Area	Peak Height	%Area
1		8.136	670602	131595	100.00
Sum					100.0

8a @254 nm

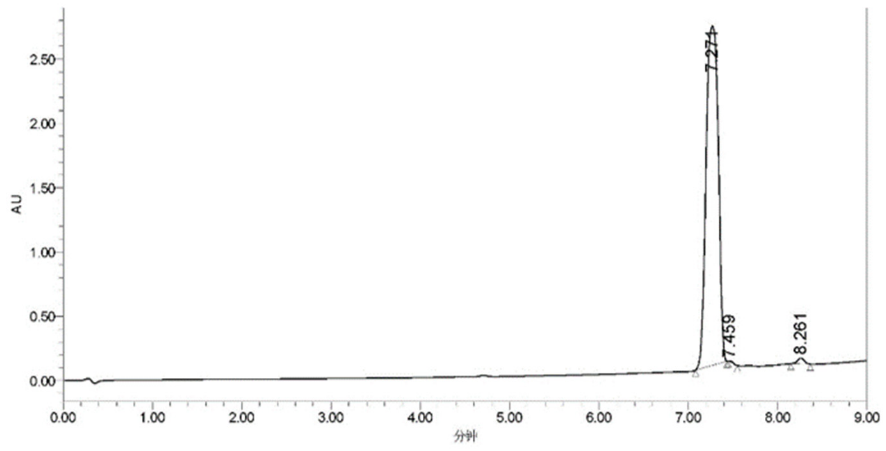

		Retention Time (Min)	Area	Peak Height	%Area
1		7.271	25133382	2647718	99.00
2		7.459	23308	4030	0.09
3		8.261	230978	45531	0.91
Sum					100.0

8b @230 nm

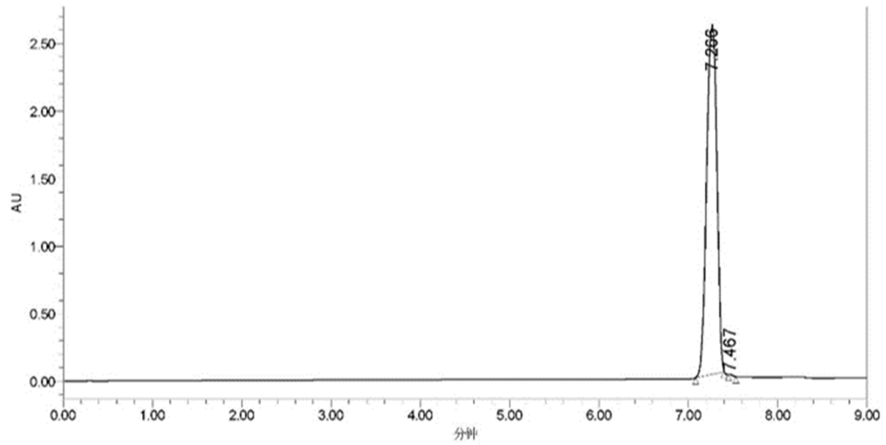

		Retention Time (Min)	Area	Peak Height	%Area
1		7.266	20728348	2594123	99.97
2		7.467	6210	2482	0.03
Sum					100.0

8b @254 nm

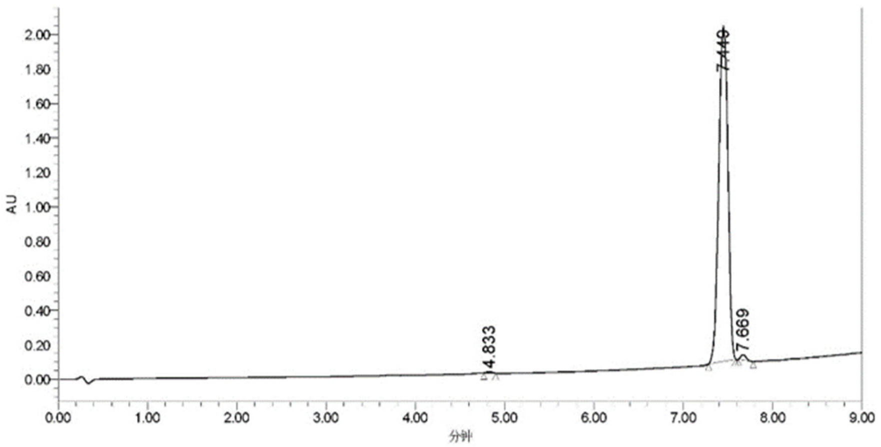

		Retention Time (Min)	Area	Peak Height	%Area
1		4.833	35322	8239	0.25
2		7.449	13885871	1950909	98.88
3		7.669	122129	29263	0.87
Sum					100.0

8c @230 nm

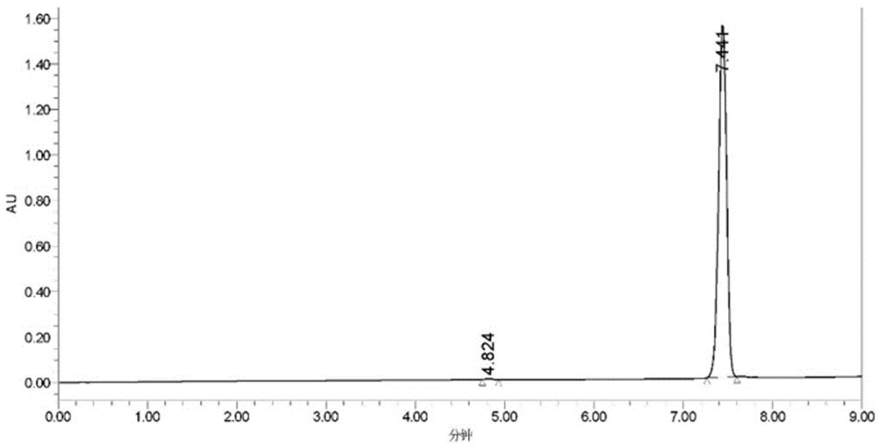

		Retention Time (Min)	Area	Peak Height	%Area
1		4.824	18329	3988	0.19
2		7.441	9692769	1556190	99.81
Sum					100.0

8c @254 nm

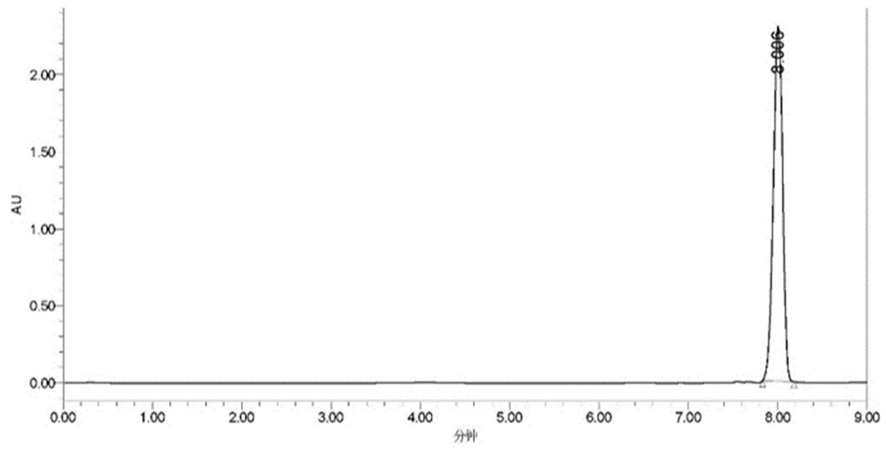

		Retention Time (Min)	Area	Peak Height	%Area
1		8.006	16664123	2317255	100.00
Sum					100.0

8d @230 nm

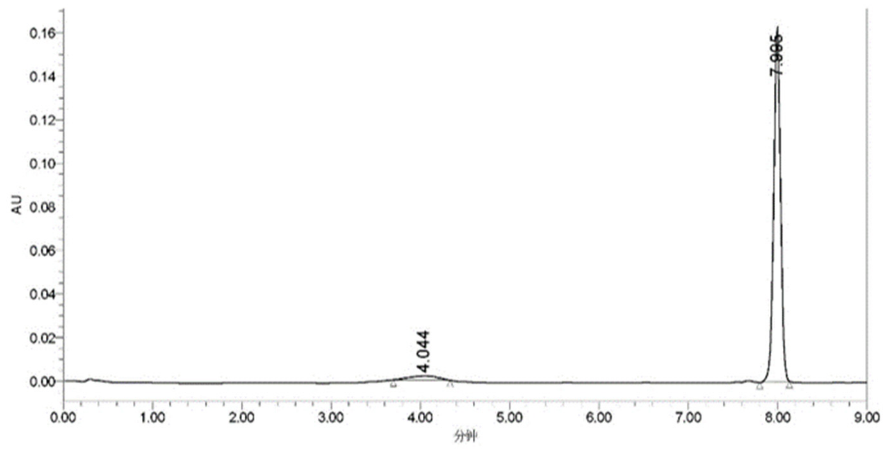

		Retention Time (Min)	Area	Peak Height	%Area
1		4.044	42009	1898	4.61
2		7.995	868508	162466	95.39
Sum					100.0

8d @254 nm

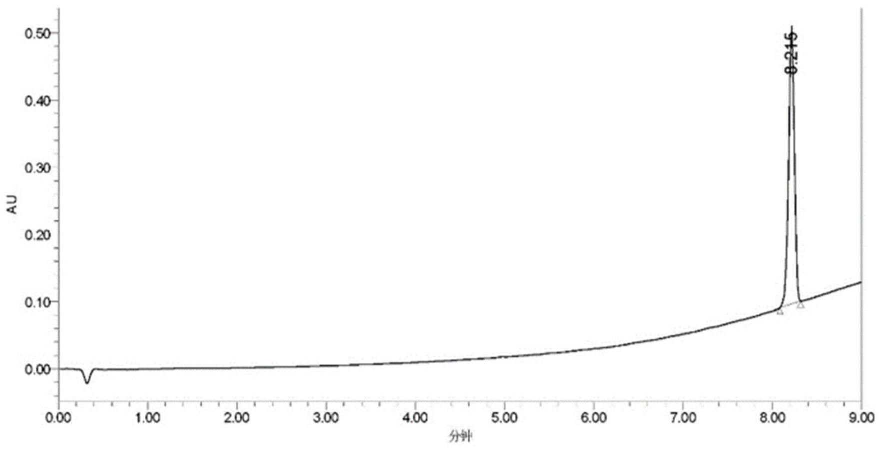

		Retention Time (Min)	Area	Peak Height	%Area
1		8.215	1857093	406539	100.00
Sum					100.0

8e @230 nm

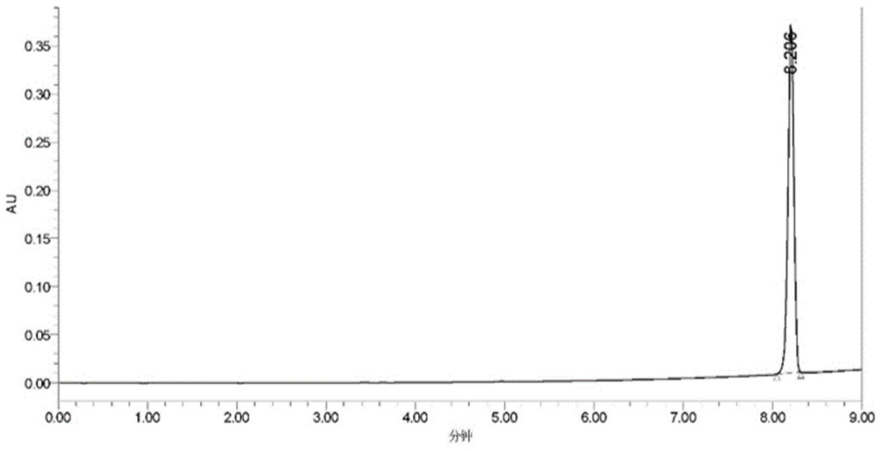

		Retention Time (Min)	Area	Peak Height	%Area
1		8.206	1675962	360092	100.00
Sum					100.0

8e @254 nm

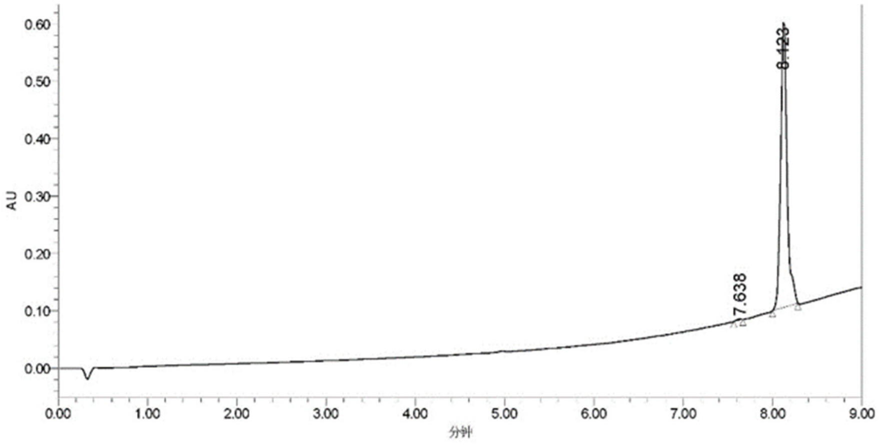

		Retention Time (Min)	Area	Peak Height	%Area
1		7.638	6176	1425	0.25
2		8.123	2485967	496974	99.75
Sum					100.0

8e' @230 nm

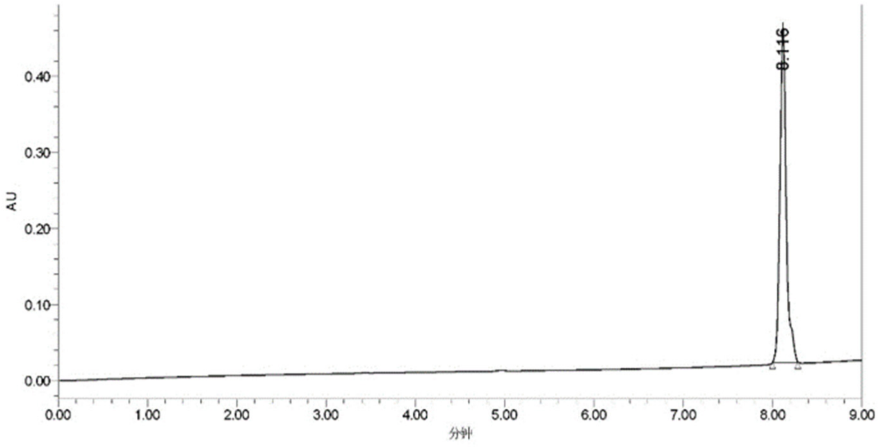

		Retention Time (Min)	Area	Peak Height	%Area
1		8.116	2177366	438310	100.00
Sum					100.0

8e' @254 nm

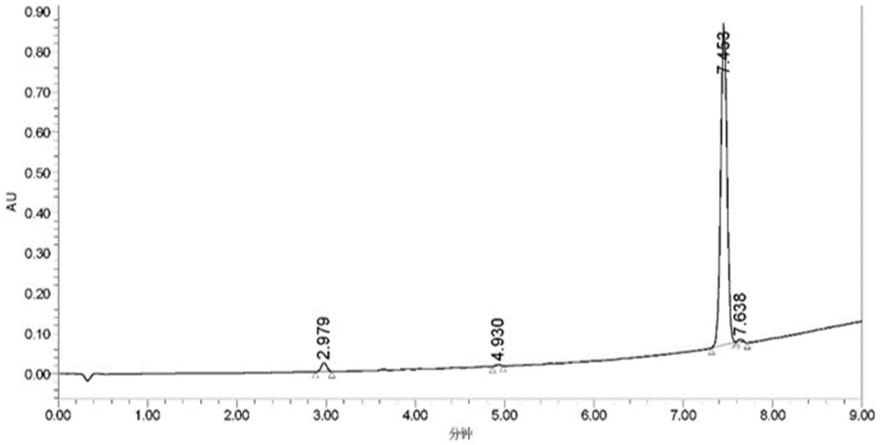

		Retention Time (Min)	Area	Peak Height	%Area
1		2.979	94990	21900	2.34
2		4.930	16665	4411	0.41
3		7.453	3922710	791069	96.72
4		7.638	21569	6357	0.53
Sum					100.0

8f @230 nm

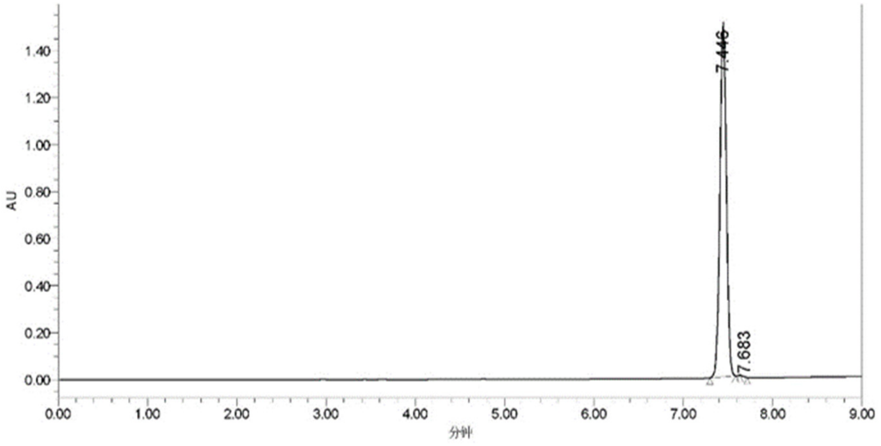

		Retention Time (Min)	Area	Peak Height	%Area
1		7.446	7555475	1494276	99.94
2		7.683	4538	-1810	0.06
Sum					100.0

8f @254 nm

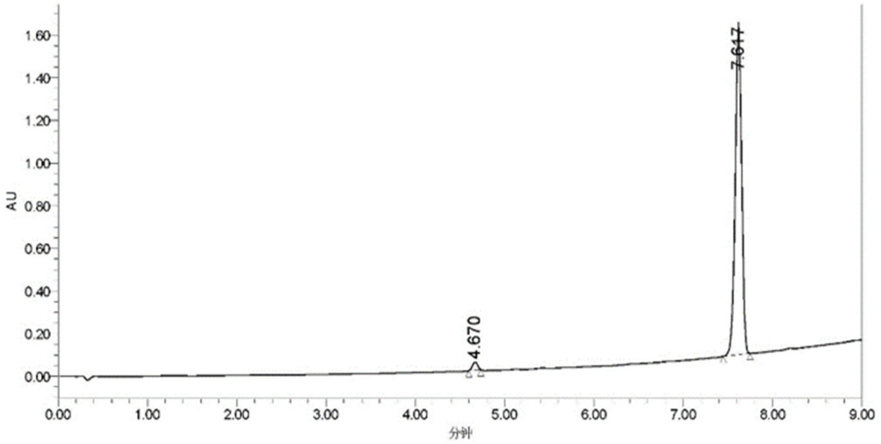

		Retention Time (Min)	Area	Peak Height	%Area
1		4.670	153312	37476	1.94
2		7.617	7752207	1536798	98.06
Sum					100.0

8g @230 nm

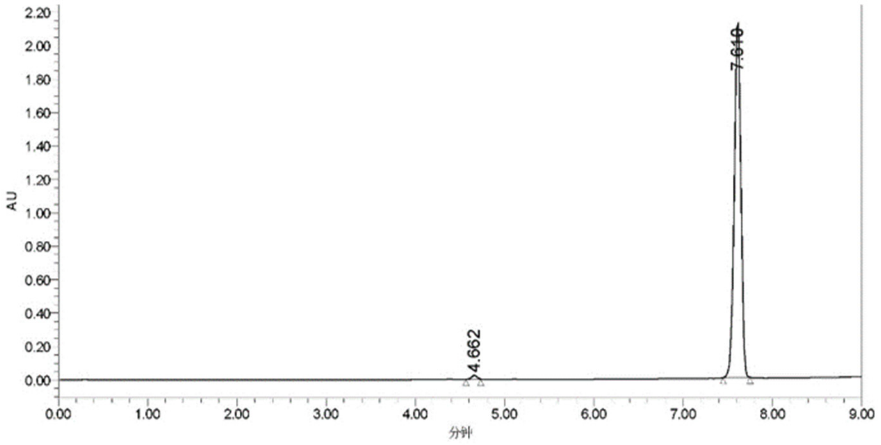

		Retention Time (Min)	Area	Peak Height	%Area
1		4.662	101634	23332	0.91
2		7.610	11063573	2127915	99.09
Sum					100.0

8g @254 nm

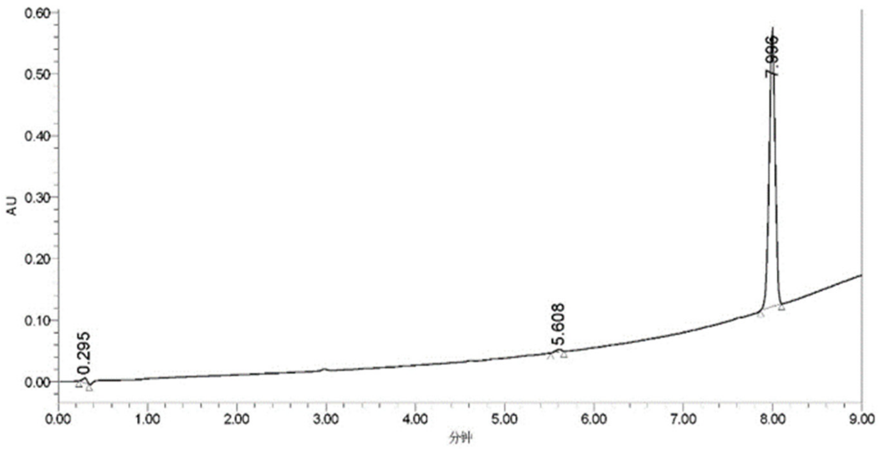

		Retention Time (Min)	Area	Peak Height	%Area
1		0.295	28697	7538	1.34
2		5.608	19060	4319	0.89
3		7.996	2095333	448464	97.77
Sum					100.0

8h @230 nm

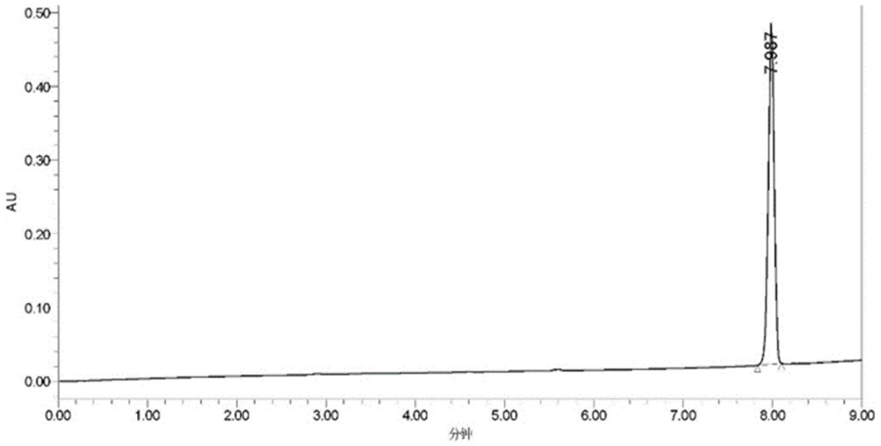

		Retention Time (Min)	Area	Peak Height	%Area
1		7.987	2148181	456784	100.00
Sum					100.0

8h @254 nm

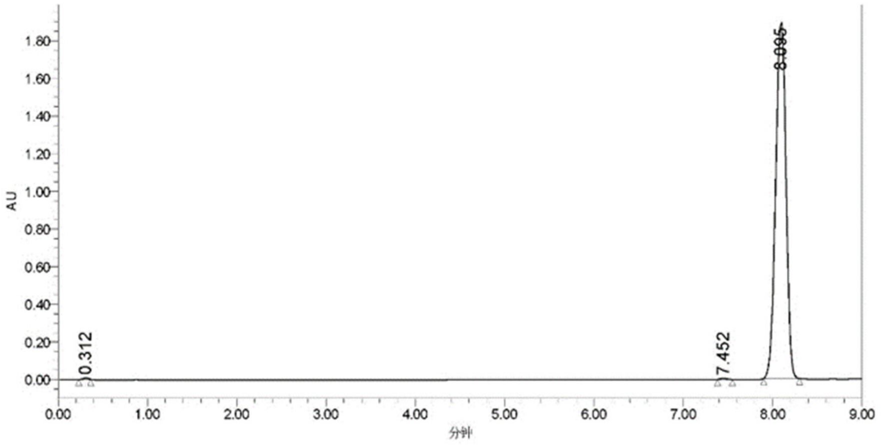

		Retention Time (Min)	Area	Peak Height	%Area
1		0.312	33476	7988	0.22
2		7.452	26113	5471	0.17
3		8.095	15163366	1900237	99.61
Sum					100.0

8i @230 nm

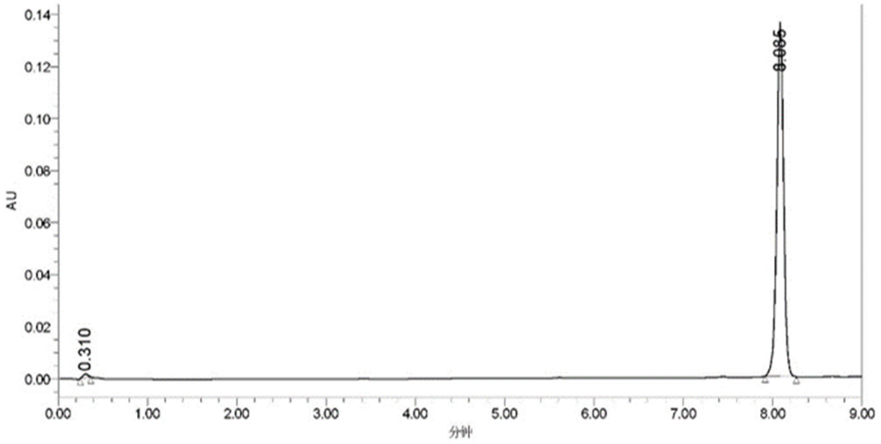

		Retention Time (Min)	Area	Peak Height	%Area
1		0.310	5630	1364	0.78
2		8.085	716664	134501	99.22
Sum					100.0

8i @254 nm

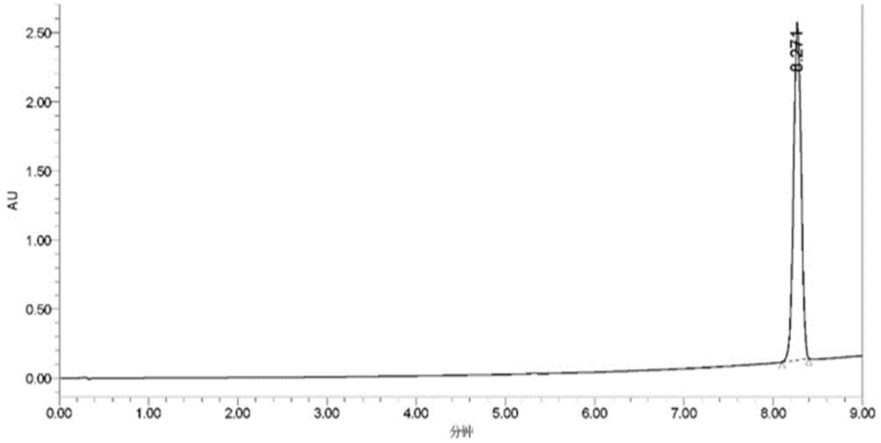

		Retention Time (Min)	Area	Peak Height	%Area
1		8.271	14275825	2438632	100.00
Sum					100.0

8j @230 nm

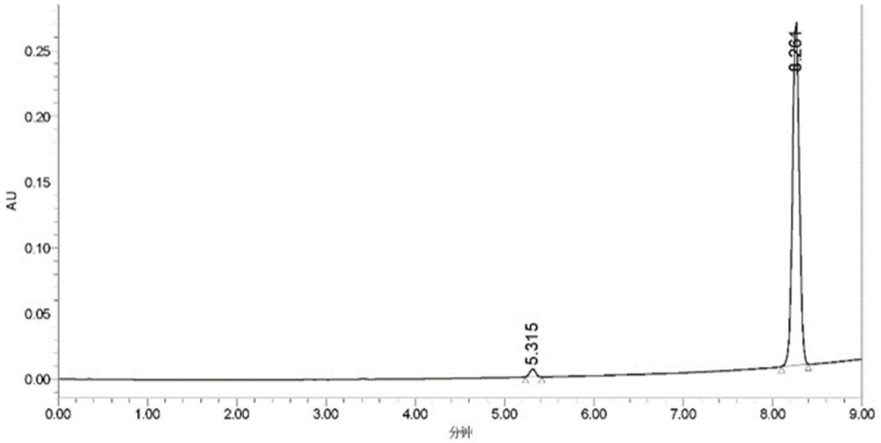

		Retention Time (Min)	Area	Peak Height	%Area
1		5.315	26612	5958	1.90
2		8.261	1374108	260258	98.10
Sum					100.0

8j @254 nm

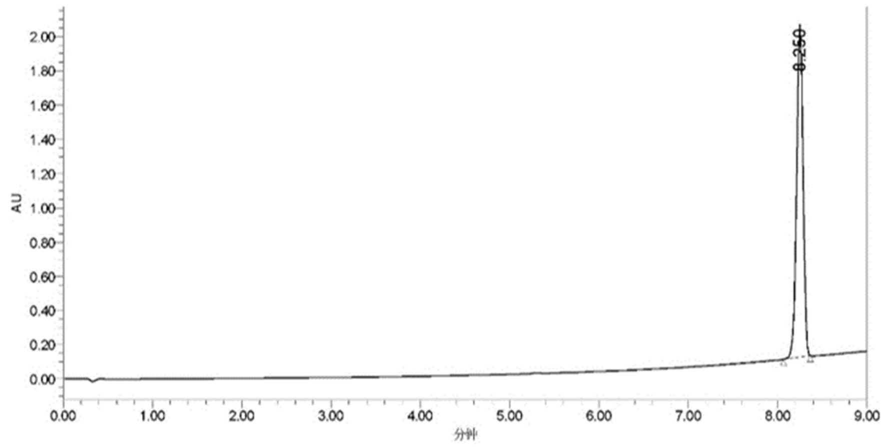

		Retention Time (Min)	Area	Peak Height	%Area
1		8.250	9677612	1914959	100.00
Sum					100.0

8k @230 nm

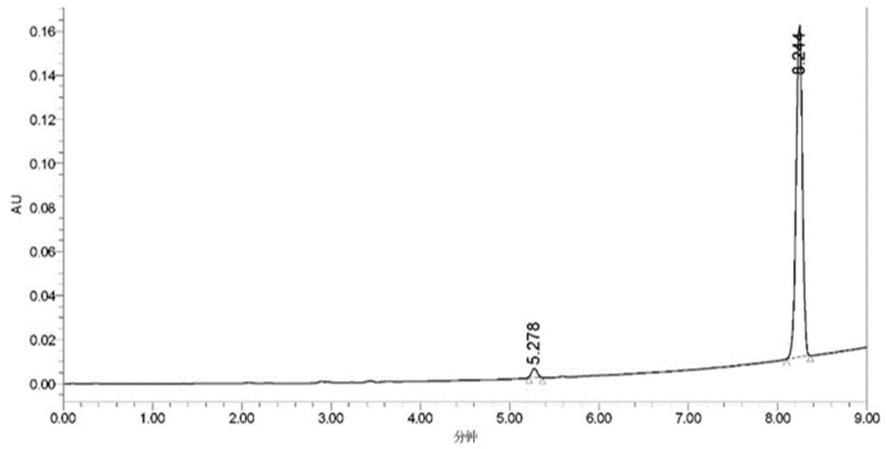

		Retention Time (Min)	Area	Peak Height	%Area
1		5.278	16720	3949	2.23
2		8.244	732674	150358	97.77
Sum					100.0

8k @254 nm

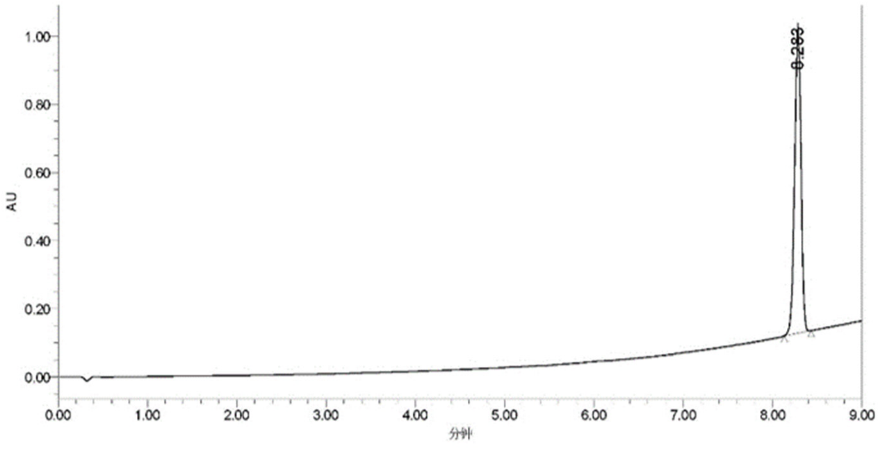

		Retention Time (Min)	Area	Peak Height	%Area
1		8.283	4394914	896119	100.00
Sum					100.0

8l @230 nm

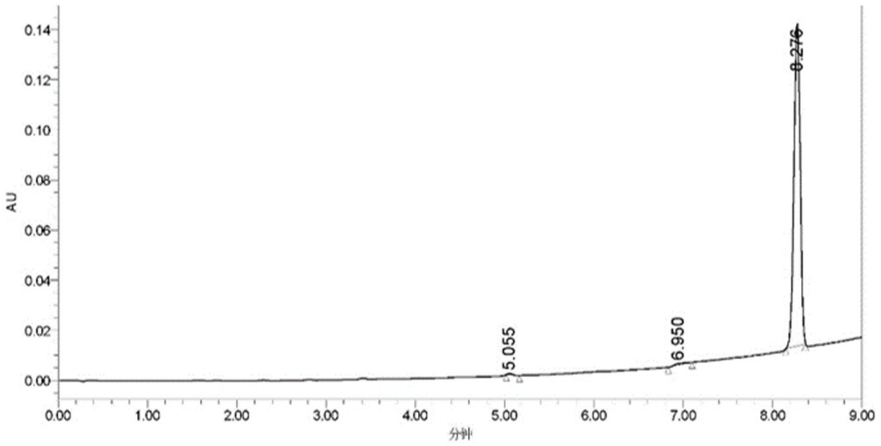

		Retention Time (Min)	Area	Peak Height	%Area
1		5.055	1958	559	0.31
2		6.950	4893	630	0.79
3		8.276	616157	129156	98.90
Sum					100.0

8l @254 nm

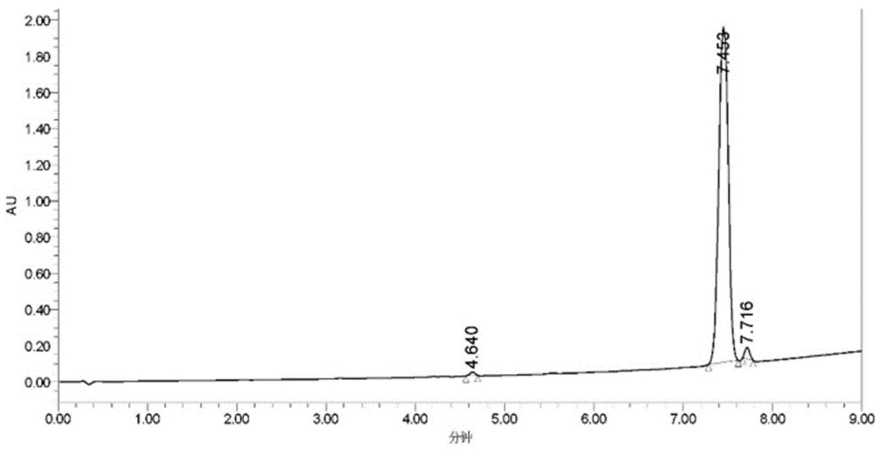

		Retention Time (Min)	Area	Peak Height	%Area
1		4.640	79863	19759	0.56
2		7.453	13857102	1860809	97.77
3		7.716	236879	62615	1.67
Sum					100.0

8m @ 230 nm

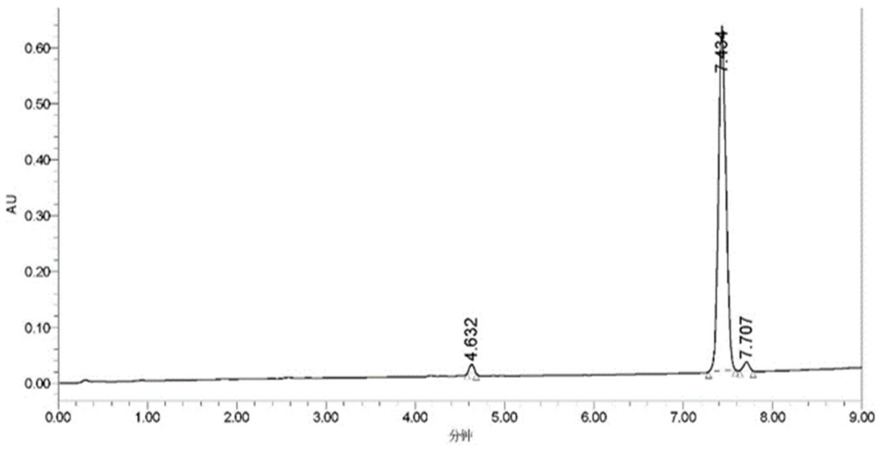

		Retention Time (Min)	Area	Peak Height	%Area
1		4.632	56442	16381	1.50
2		7.434	3635720	611563	96.67
3		7.707	68619	15619	1.82
Sum					100.0

8m @254 nm

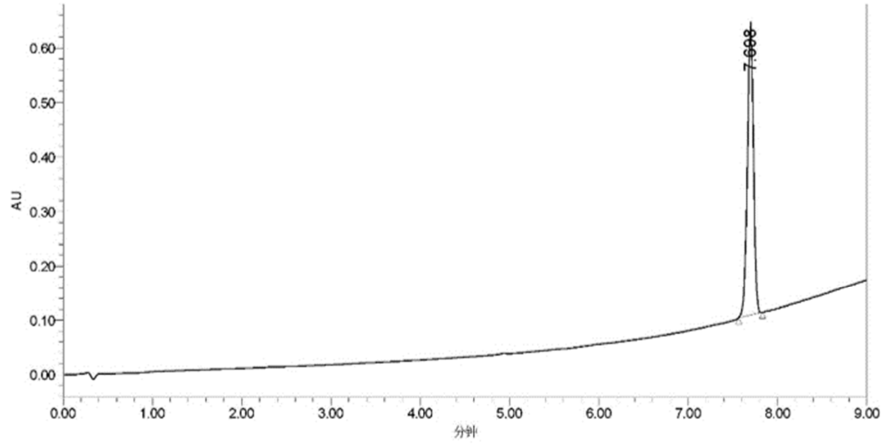

		Retention Time (Min)	Area	Peak Height	%Area
1		7.698	2587324	530259	100.00
Sum					100.0

8n @230 nm

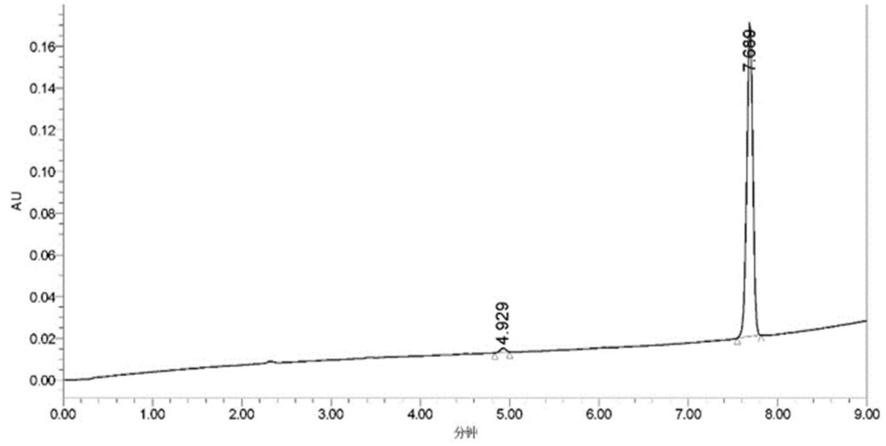

		Retention Time (Min)	Area	Peak Height	%Area
1		4.929	8888	2016	1.22
2		7.689	721247	149876	98.78
Sum					100.0

8n @254 nm

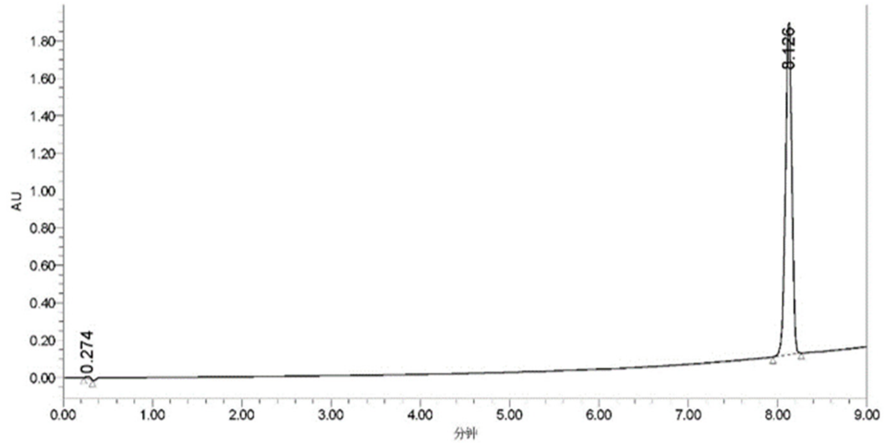

		Retention Time (Min)	Area	Peak Height	%Area
1		0.274	40309	10891	0.45
2		8.126	8893775	1782955	99.55
Sum					100.0

8p @230 nm

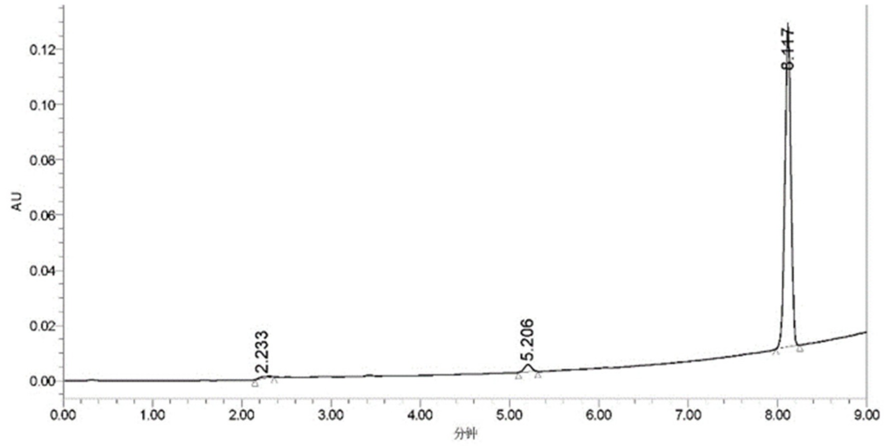

		Retention Time (Min)	Area	Peak Height	%Area
1		2.233	5741	684	1.02
2		5.206	14876	2669	2.64
3		8.117	543831	115465	96.35
Sum					100.0

8p @254 nm

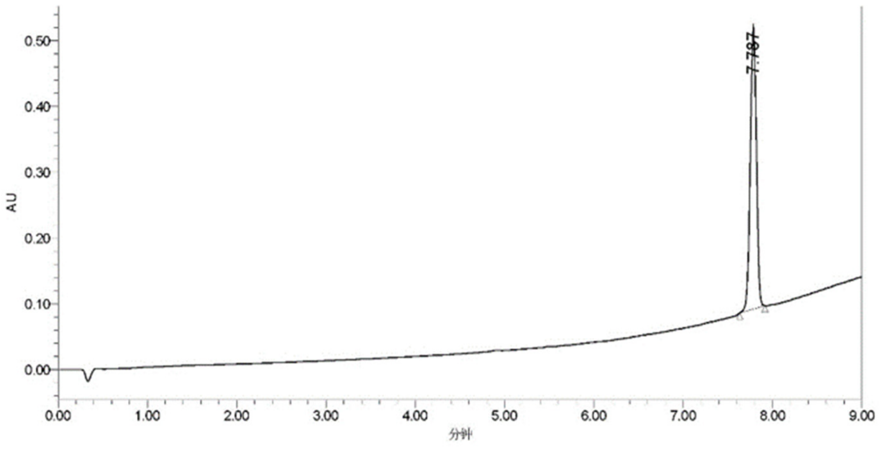

		Retention Time (Min)	Area	Peak Height	%Area
1		7.787	2019234	428278	100.00
Sum					100.0

8q @230 nm

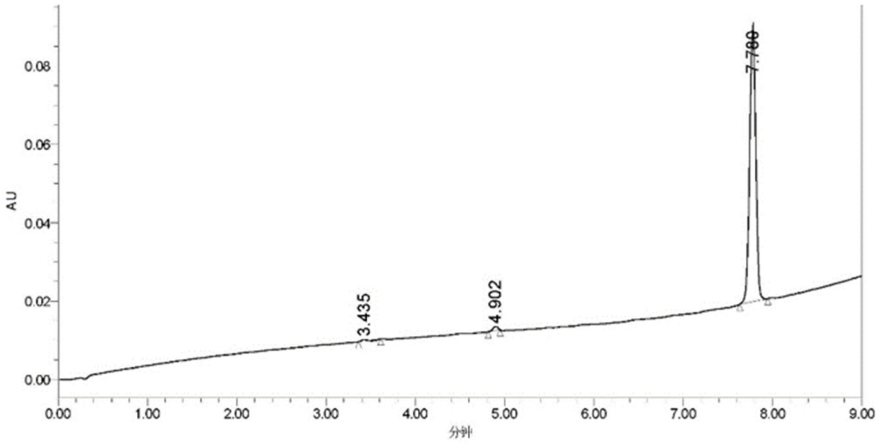

		Retention Time (Min)	Area	Peak Height	%Area
1		3.435	2320	334	0.69
2		4.902	4092	1055	1.21
3		7.780	332184	70302	98.11
Sum					100.0

8q @254 nm

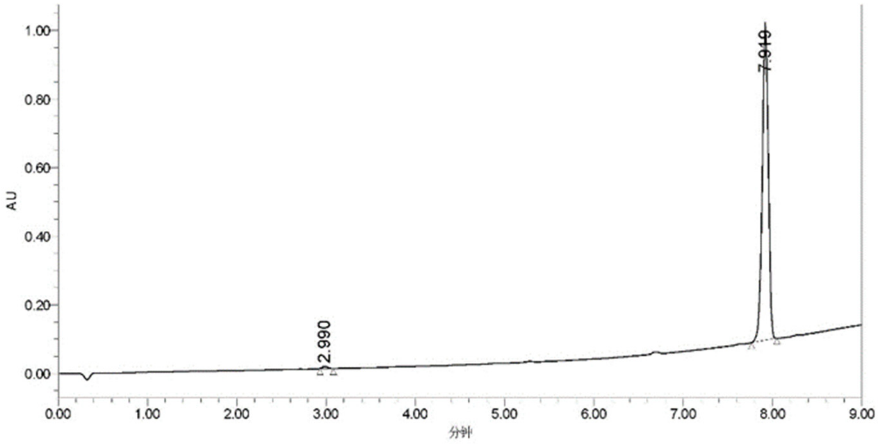

		Retention Time (Min)	Area	Peak Height	%Area
1		2.990	25183	6488	0.57
2		7.919	4376667	914093	99.43
Sum					100.0

8r @230 nm

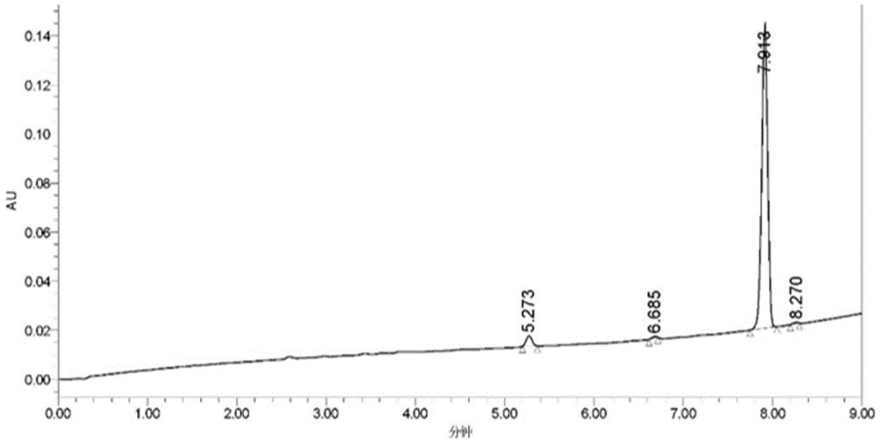

		Retention Time (Min)	Area	Peak Height	%Area
1		5.273	19123	4260	3.20
2		6.685	2450	724	0.41
3		7.913	574393	123219	96.13
4		8.270	1572	427	0.26
Sum					100.0

8r @254 nm

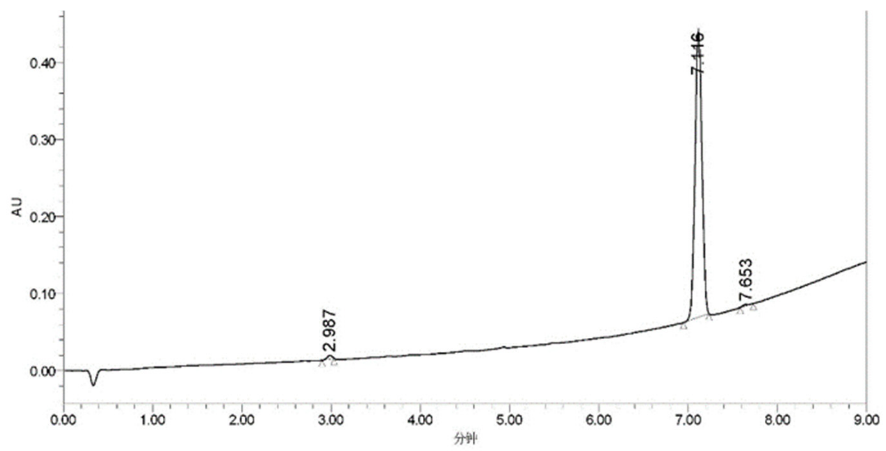

		Retention Time (Min)	Area	Peak Height	%Area
1		2.987	18652	5153	0.93
2		7.116	1979638	371106	98.73
3		7.653	6896	1498	0.34
Sum					100.0

8s @230 nm

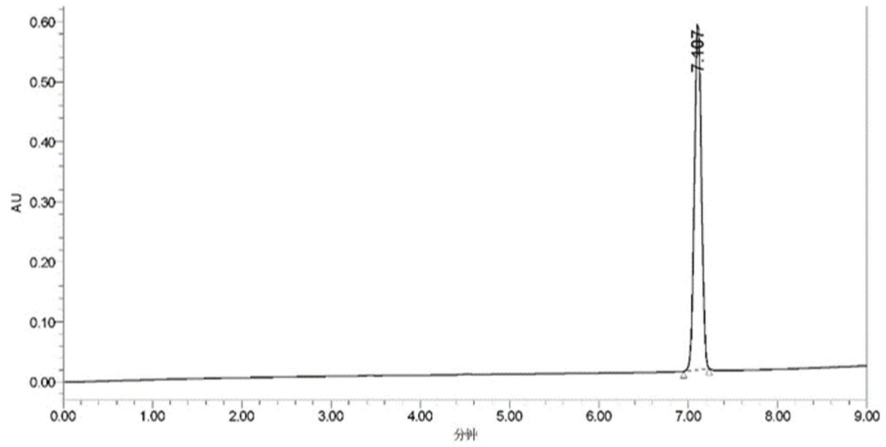

		Retention Time (Min)	Area	Peak Height	%Area
1		7.107	3108200	578801	100.00
Sum					100.0

8s @254 nm

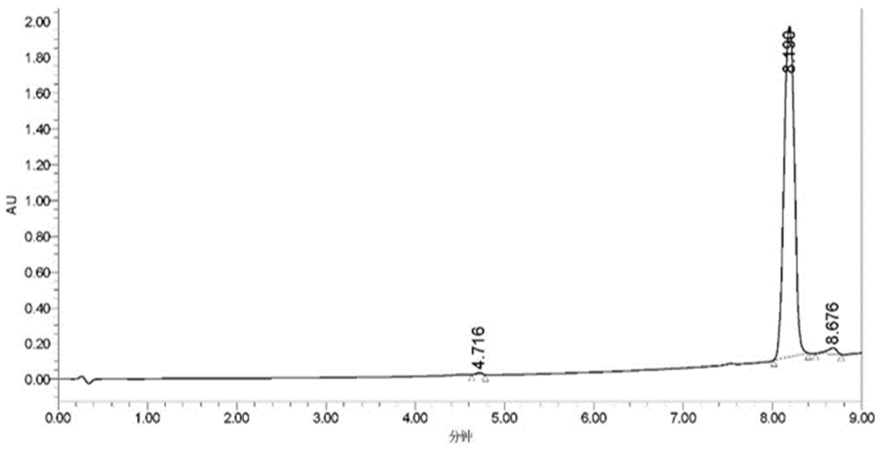

		Retention Time (Min)	Area	Peak Height	%Area
1		4.716	48259	10737	0.31
2		8.190	15108555	1860735	97.98
3		8.676	262925	34746	1.71
Sum					100.0

8t @230 nm

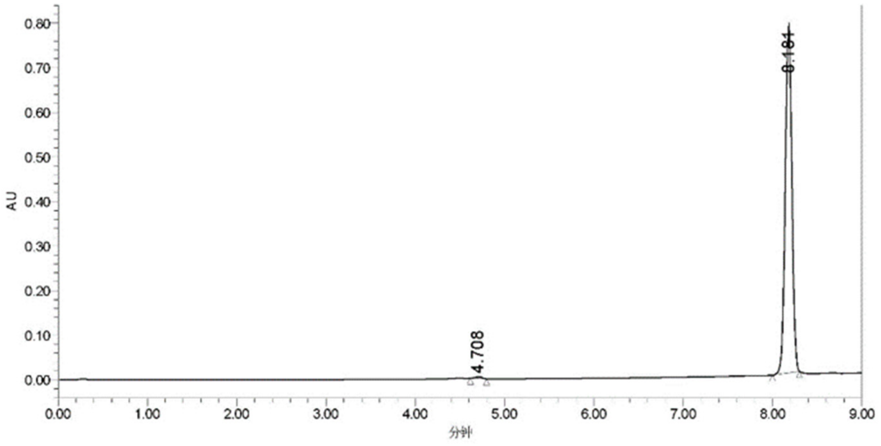

		Retention Time (Min)	Area	Peak Height	%Area
1		4.708	20276	4212	0.50
2		8.181	4031042	777141	99.50
Sum					100.0

8t @254 nm

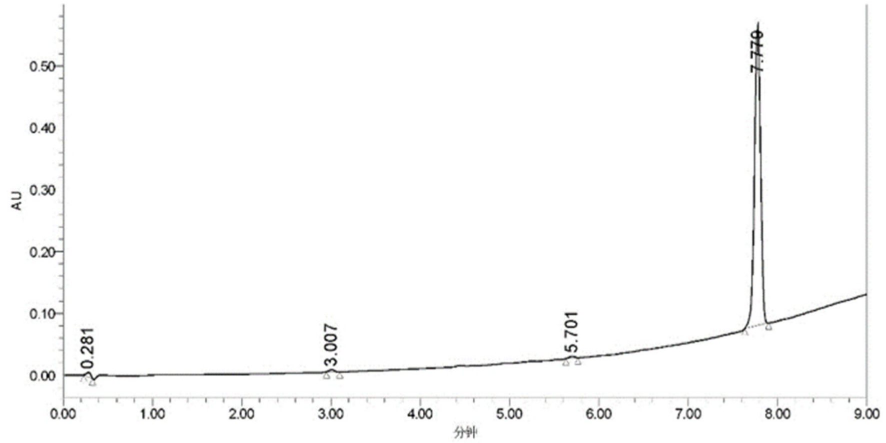

		Retention Time (Min)	Area	Peak Height	%Area
1		0.281	25269	7117	1.08
2		3.007	13699	3508	0.59
3		5.701	13003	3113	0.56
4		7.779	2281215	485595	97.77
Sum					100.0

8u @230 nm

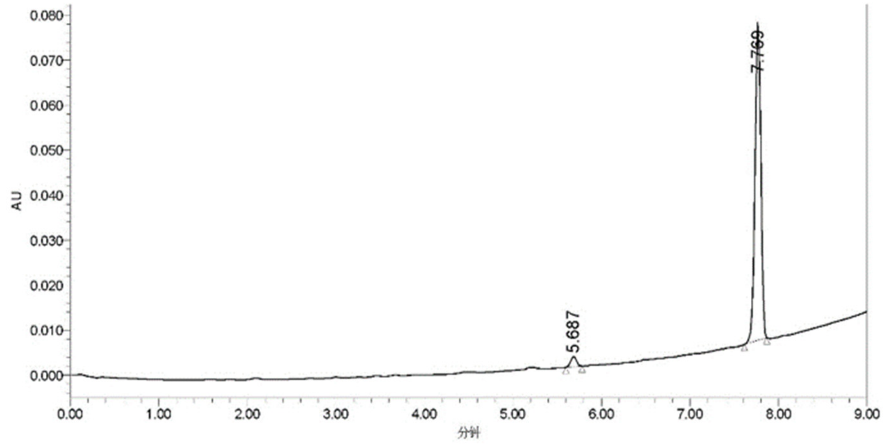

		Retention Time (Min)	Area	Peak Height	%Area
1		5.687	10358	2251	3.10
2		7.769	323637	69631	96.90
Sum					100.0

8u @254 nm

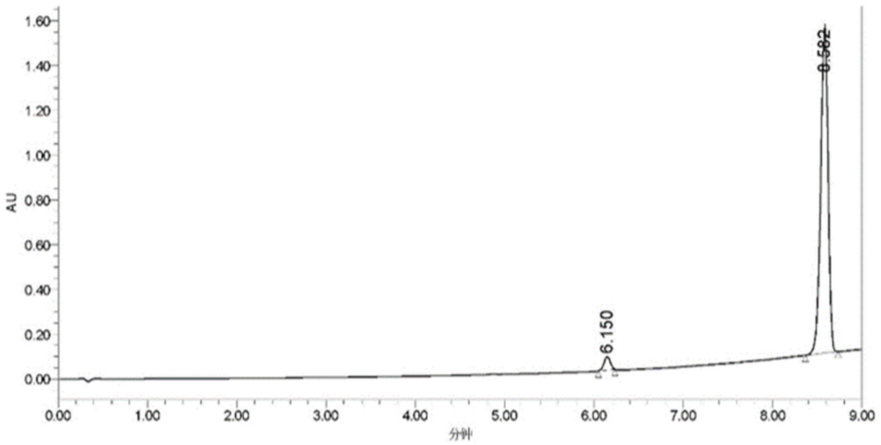

		Retention Time (Min)	Area	Peak Height	%Area
1		6.150	290201	59348	3.35
2		8.582	8373266	1456211	96.65
Sum					100.0

8v @230 nm

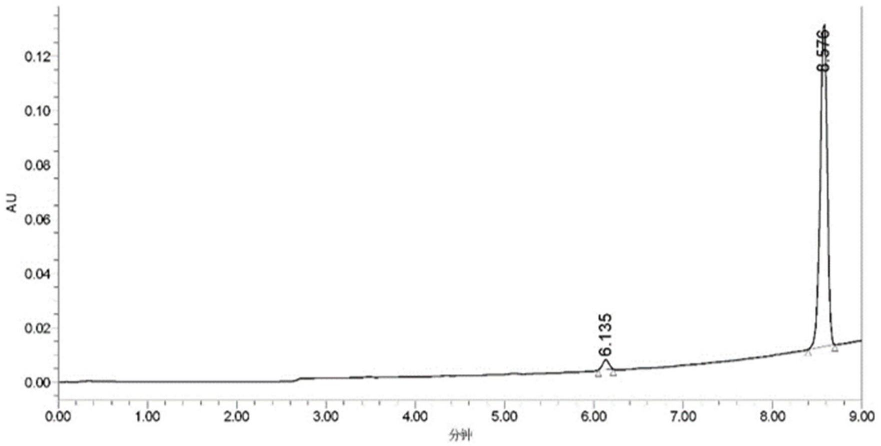

		Retention Time (Min)	Area	Peak Height	%Area
1		6.135	18220	3687	2.74
2		8.576	647484	119307	97.26
Sum					100.0

8v @254 nm

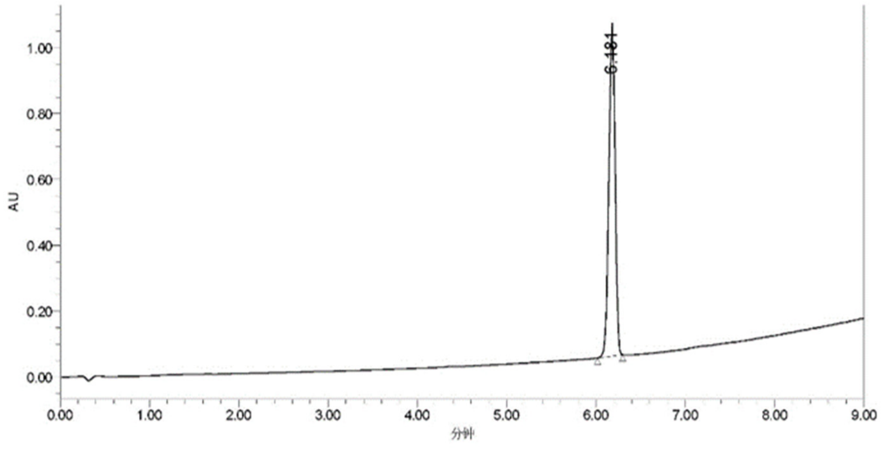

		Retention Time (Min)	Area	Peak Height	%Area
1		6.181	4827709	997879	100.00
Sum					100.0

8w @230 nm

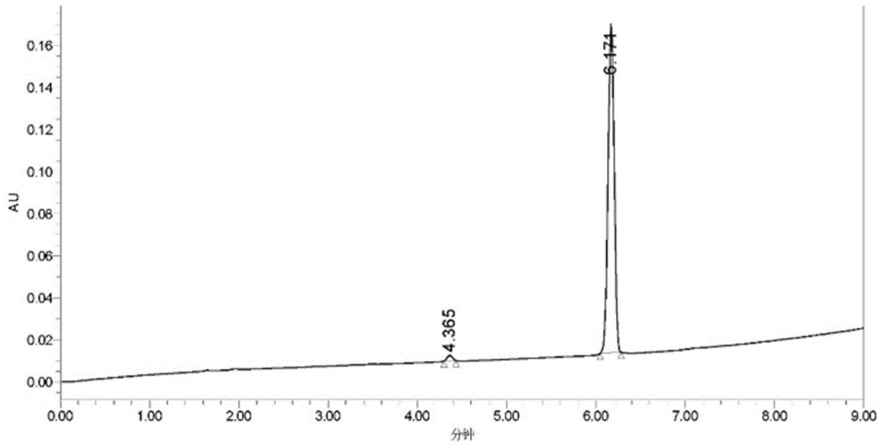

		Retention Time (Min)	Area	Peak Height	%Area
1		4.365	10781	2655	1.44
2		6.171	739405	155361	98.56
Sum					100.0

8w @254 nm

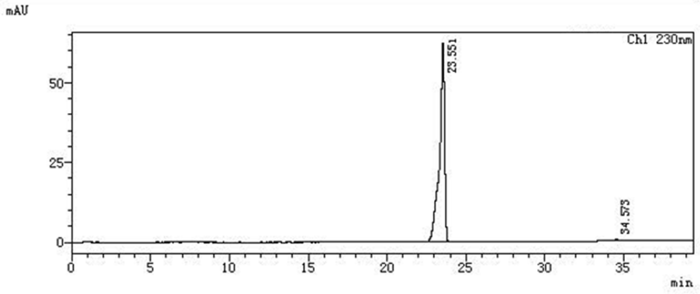

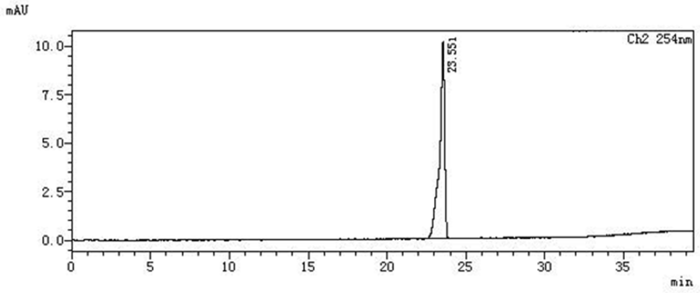

	Retention Time (Min)	Area	Peak Height	%Area
1	23.551	1394550	62226	99.687
2	34.573	4385	251	0.313
sum		1398936	62477	100.000

	Retention Time (Min)	Area	Peak Height	%Area
1	23.551	227530	10116	100.000
sum		227530	10116	100.000

8x @ 230 nm & 254 nm

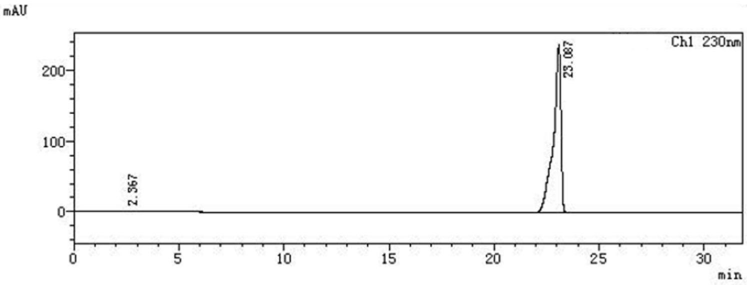

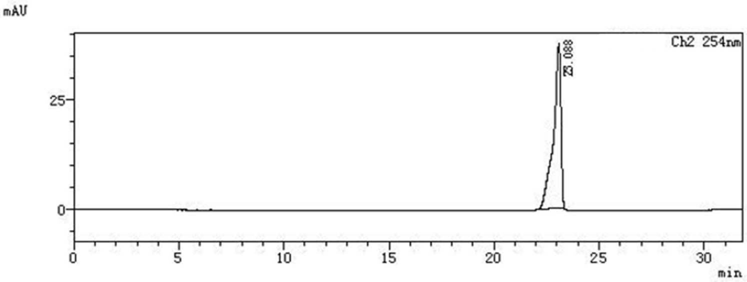

	Retention Time (Min)	Area	Peak Height	%Area
1	2.367	828	156	0.014
2	23.087	5896083	238196	99.986
sum		5896911	238351	100.000

	Retention Time (Min)	Area	Peak Height	%Area
1	23.088	923576	37589	100.000
sum		923576	37589	100.000

8x' @ 230 nm & 254 nm

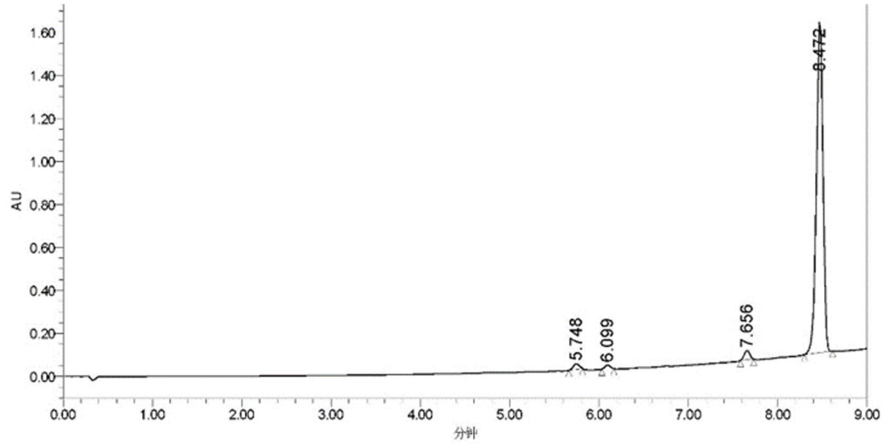

		Retention Time (Min)	Area	Peak Height	%Area
1		5.748	120733	25381	1.45
2		6.099	74262	18218	0.89
3		7.656	192204	44353	2.31
4		8.472	7924302	1531733	95.34
Sum					100.0

8y @230 nm

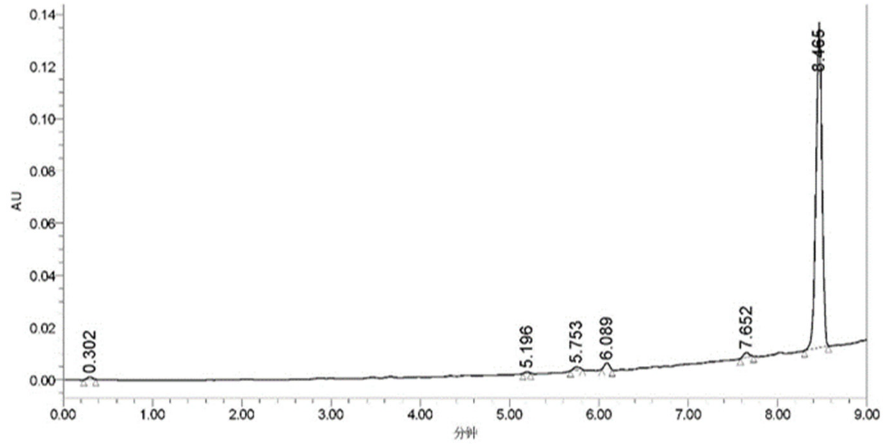

		Retention Time (Min)	Area	Peak Height	%Area
1		0.302	4365	1080	0.72
2		5.196	1814	591	0.30
3		5.753	5808	1224	0.95
4		6.089	9535	2479	1.56
5		7.652	7792	1832	1.28
6		8.465	580427	122481	95.19
Sum					100.0

8y @254 nm

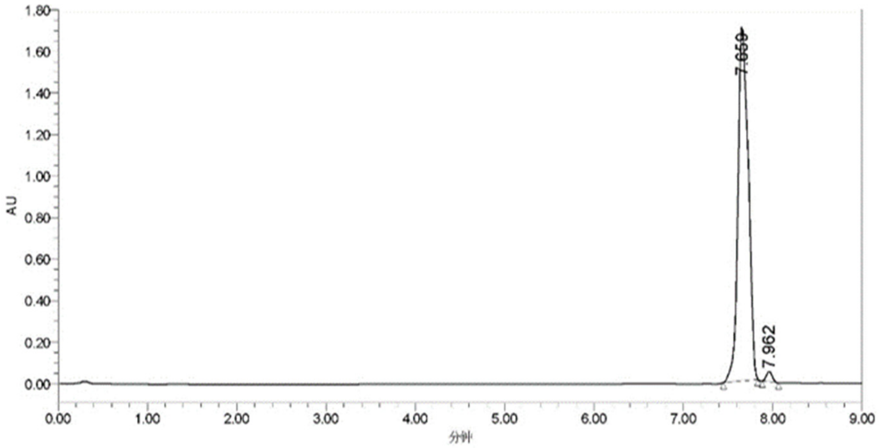

		Retention Time (Min)	Area	Peak Height	%Area
1		7.659	13794786	1717847	98.39
2		7.962	225797	49018	1.61
Sum					100.0

8ab @230 nm

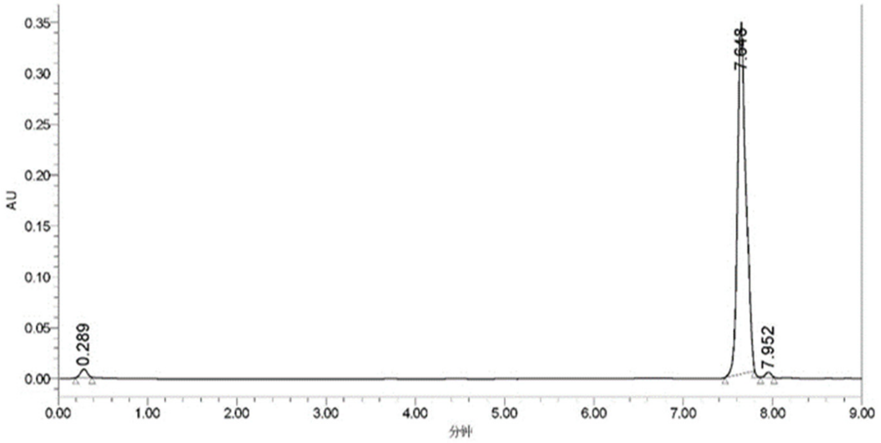

		Retention Time (Min)	Area	Peak Height	%Area
1		0.289	41426	8156	1.77
2		7.648	2278634	342272	97.36
3		7.952	20411	4722	0.87
Sum					100.0

8ab @254 nm

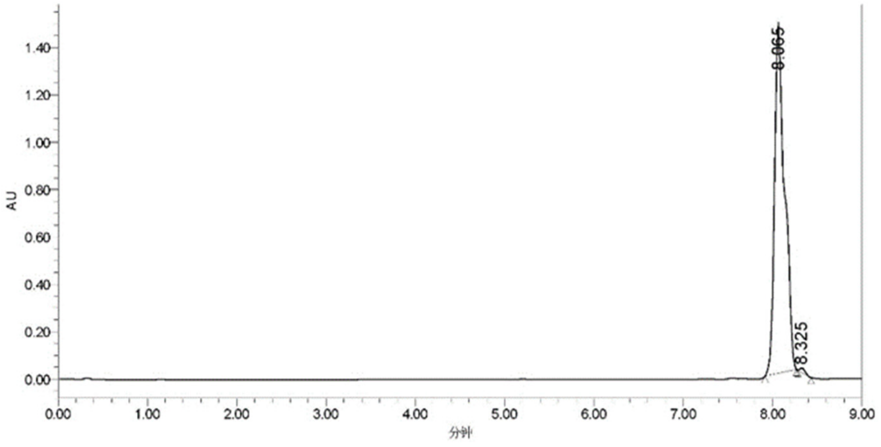

		Retention Time (Min)	Area	Peak Height	%Area
1		8.065	11479958	1467099	99.39
2		8.325	70934	17648	0.61
Sum					100.0

8ac @230 nm

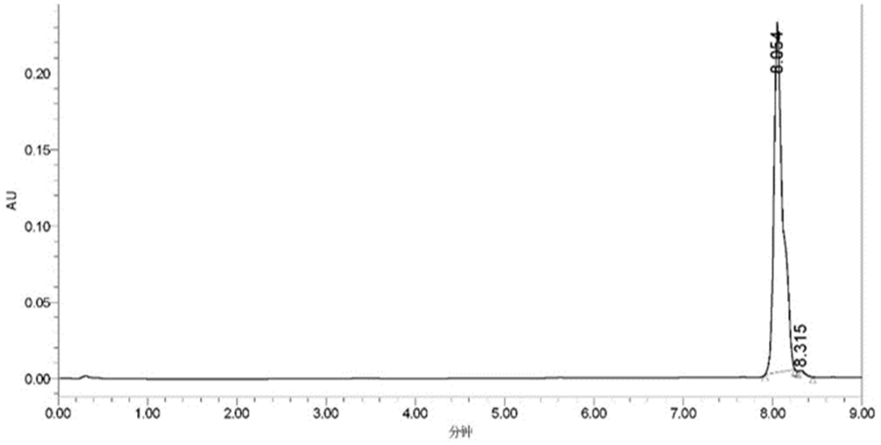

		Retention Time (Min)	Area	Peak Height	%Area
1		8.054	1568607	227623	99.62
2		8.315	6042	1285	0.38
Sum					100.0

8ac @254 nm

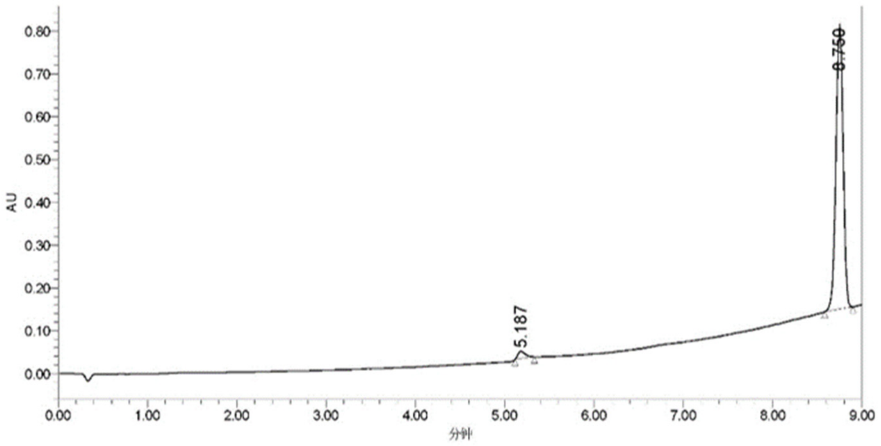

		Retention Time (Min)	Area	Peak Height	%Area
1		5.187	100580	17098	2.67
2		8.750	3660328	659564	97.33
Sum					100.0

8ad @230 nm

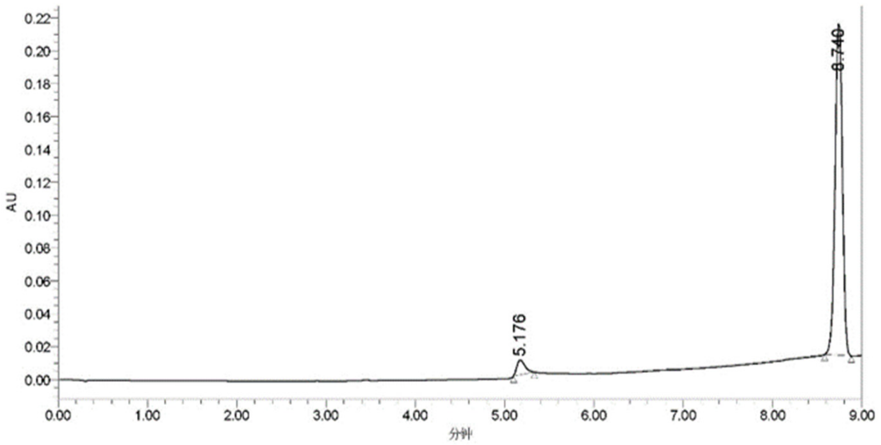

		Retention Time (Min)	Area	Peak Height	%Area
1		5.176	57159	9181	4.82
2		8.740	1127704	202679	95.18
Sum					100.0

8ad @254 nm

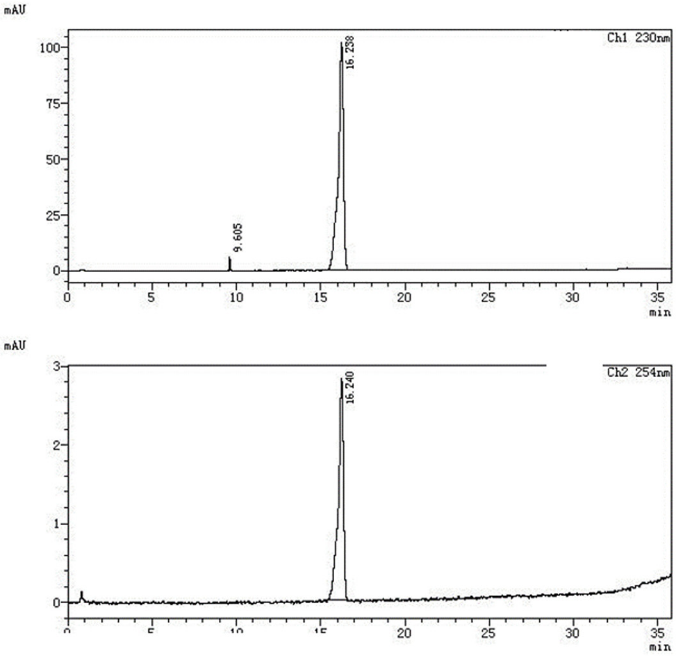

	Retention Time (Min)	Area	Peak Height	%Area
1	9.605	17884	6055	0.814
2	16.238	2178856	102165	99.186
sum		2196740	108220	100.000

	Retention Time (Min)	Area	Peak Height	%Area
1	16.240	59924	2820	100.000
sum		59924	2820	100.000

8ae @230 nm &254 nm

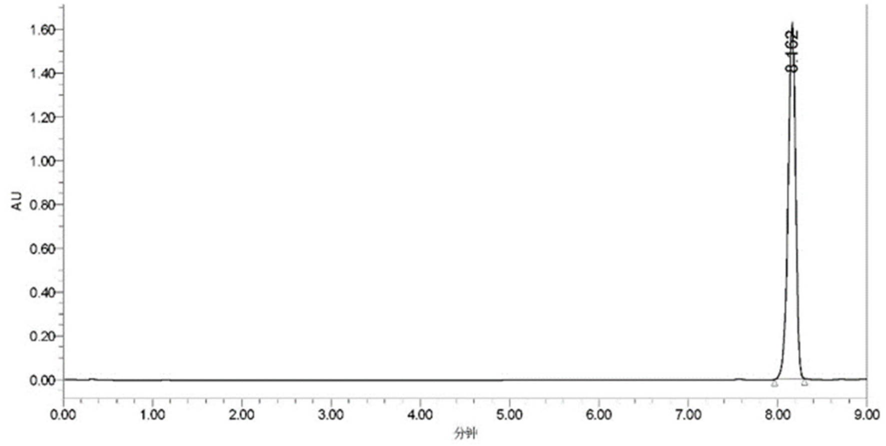

		Retention Time (Min)	Area	Peak Height	%Area
1		8.162	9551466	1622088	100.00
Sum					100.0

8af @230 nm

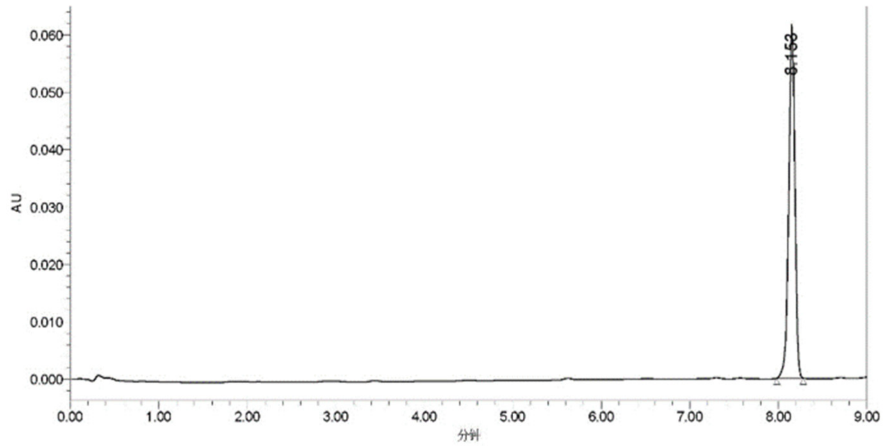

		Retention Time (Min)	Area	Peak Height	%Area
1		8.153	300110	60985	100.00
Sum					100.0

8af @254 nm

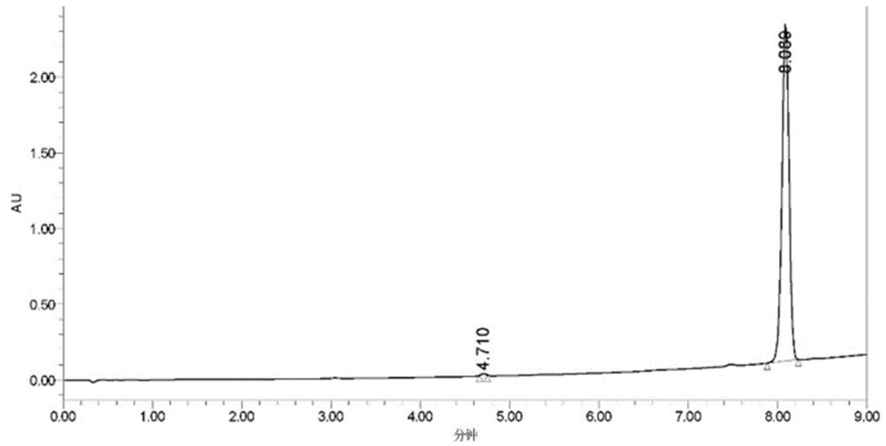

		Retention Time (Min)	Area	Peak Height	%Area
1		4.710	32978	10648	0.27
2		8.089	12407322	2223535	99.73
Sum					100.0

9 @230 nm

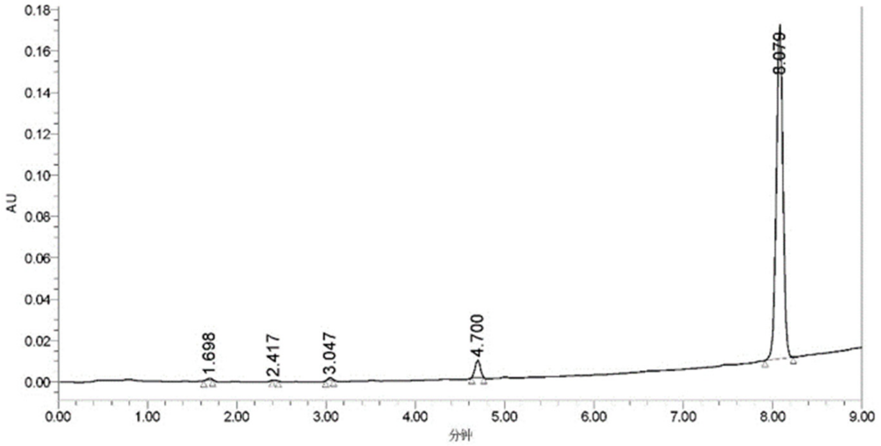

		Retention Time (Min)	Area	Peak Height	%Area
1		1.698	2828	851	0.33
2		2.417	625	199	0.07
3		3.047	3252	1057	0.38
4		4.700	33118	8185	3.83
5		8.079	825477	160236	95.40
Sum					100.0

9 @254 nm

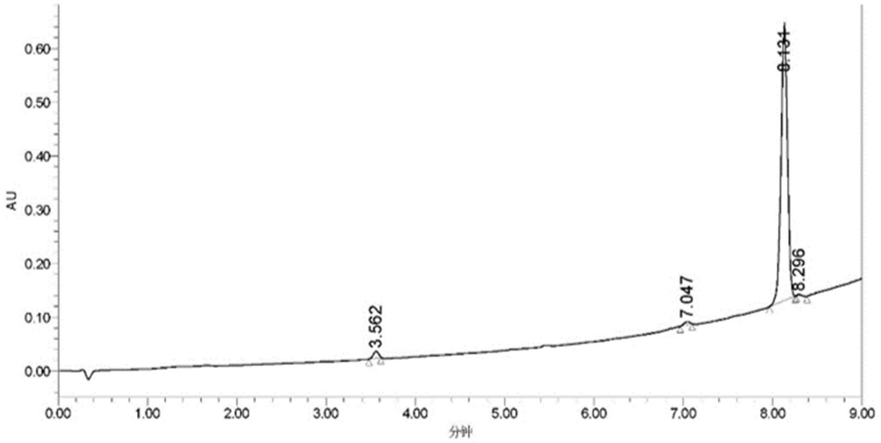

		Retention Time (Min)	Area	Peak Height	%Area
1		3.562	50327	12364	1.90
2		7.047	26328	5952	0.99
3		8.131	2568877	510481	96.84
4		8.296	7194	2167	0.27
Sum					100.0

10 @230 nm

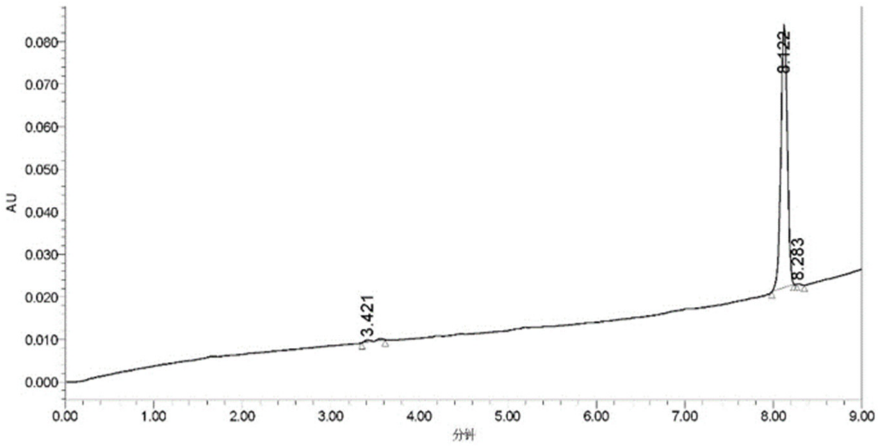

		Retention Time (Min)	Area	Peak Height	%Area
1		3.421	3792	506	1.20
2		8.122	310680	61632	98.68
3		8.283	369	151	0.12
Sum					100.0

10 @254 nm

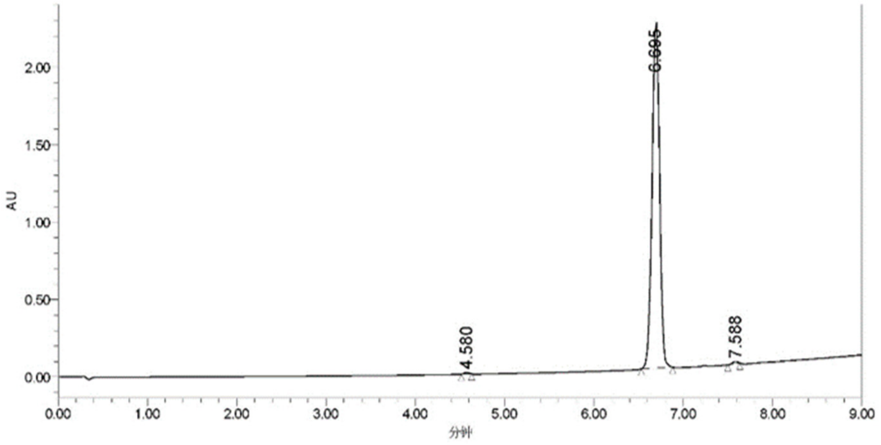

		Retention Time (Min)	Area	Peak Height	%Area
1		4.580	34148	8979	0.26
2		6.695	13089598	2231012	99.30
3		7.588	57518	14558	0.44
Sum					100.0

11 @230 nm

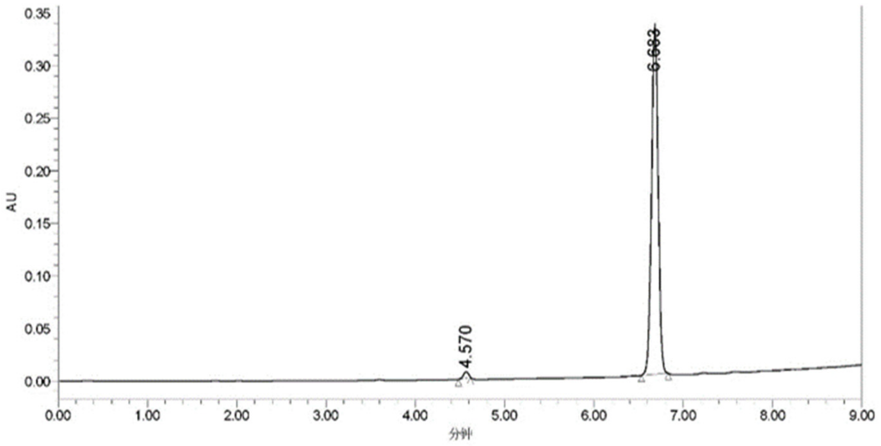

		Retention Time (Min)	Area	Peak Height	%Area
1		4.570	22510	6032	1.30
2		6.683	1703130	328971	98.70
Sum					100.0

11 @254 nm

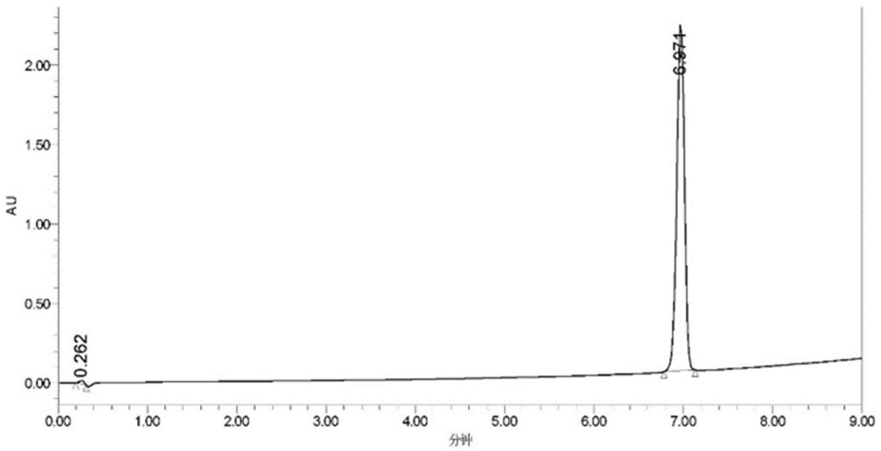

		Retention Time (Min)	Area	Peak Height	%Area
1		0.262	83118	20965	0.63
2		6.971	13097811	2170721	99.37
Sum					100.0

12 @230 nm

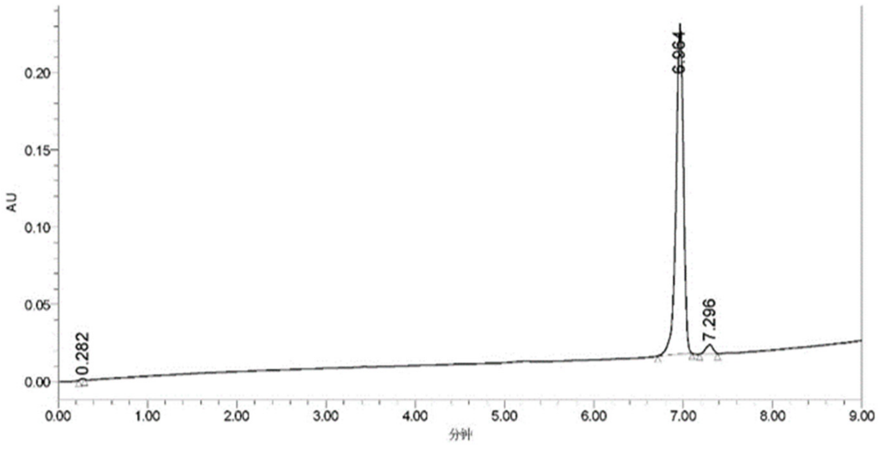

		Retention Time (Min)	Area	Peak Height	%Area
1		0.282	1247	416	0.10
2		6.964	1230635	212221	97.30
3		7.296	32913	5884	2.60
Sum					100.0

12 @254 nm

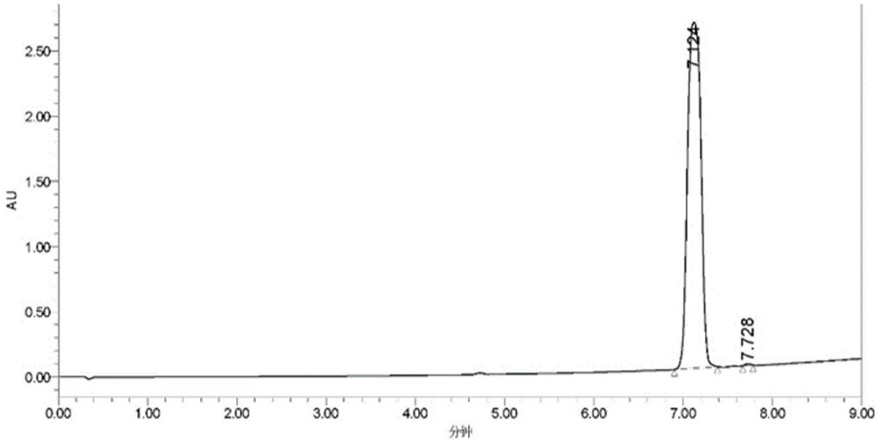

		Retention Time (Min)	Area	Peak Height	%Area
1		7.124	27421363	2663961	99.82
2		7.728	48483	12010	0.18
Sum					100.0

13 @230 nm

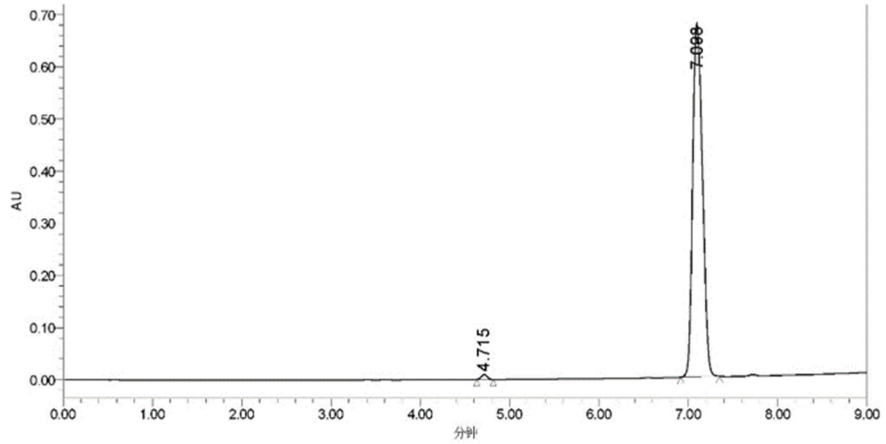

		Retention Time (Min)	Area	Peak Height	%Area
1		4.715	39589	8995	0.77
2		7.096	5104291	680579	99.23
Sum					100.0

13 @254 nm

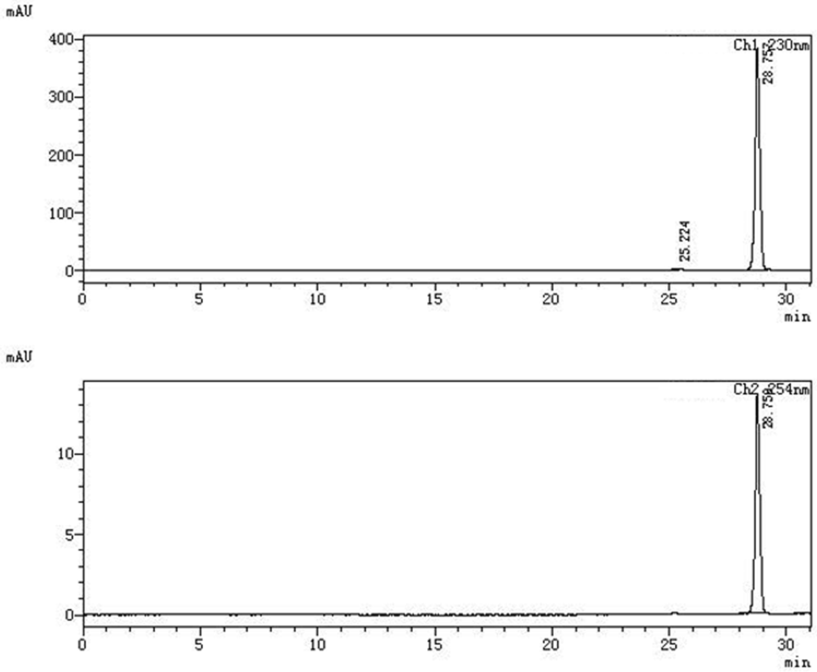

	Retention Time (Min)	Area	Peak Height	%Area
1	25.224	21417	2177	0.407
2	28.757	5237841	384494	99.593
sum		5259258	386671	100.000

	Retention Time (Min)	Area	Peak Height	%Area
1	28.759	181650	13657	100.000
sum		181650	13657	100.000

14 @ 230 nm & 254 nm
